# Conserving Large Old Trees in Guangxi, South China: Diversity, Distribution, and Preservation Strategies

**DOI:** 10.1002/ece3.73043

**Published:** 2026-02-12

**Authors:** Jiayi Yan, Jianyong Lin, Aihua Wang, Yadong Qie, Cong Hu, Zhonghua Zhang, Gang Hu

**Affiliations:** ^1^ Key Laboratory of Environment Change and Resources Use in Beibu Gulf, Ministry of Education Nanning Normal University Nanning China; ^2^ Guangxi Key Laboratory of Earth Surface Processes and Intelligent Simulation Nanning Normal University Nanning China; ^3^ Key Laboratory of Central South Fast‐Growing Timber Cultivation of Forestry Ministry of China Guangxi Forestry Research Institute Nanning China

**Keywords:** Guangxi, large old trees, preservation strategies, spatial distribution, species diversity

## Abstract

Large old trees (LOTs) are important living monuments with historical significance, landscape esthetics, and ecological functions. Understanding LOT diversity, spatial distribution, and conservation status is critical for implementing effective targeted protection measures. However, although there are numerous LOTs across Chinese provinces, a comprehensive analysis of LOTs' diversity and distribution of LOTs in Guangxi Province is lacking. Using publicly available government data and field surveys, we assessed the taxonomy, species, spatial distribution, and conservation status of LOTs aged > 500 years in Guangxi Province. We identified 2630 LOTs belonging to 149 species, 105 genera, and 48 families. Members of the *Ficus* (Moraceae), *Camphora* (Lauraceae), and *Castanopsis* (Fagaceae) genera were the most common, with *Ficus virens*, *Camphora officinarum*, and *Ficus microcarpa* representing the predominant species. A latitudinal gradient in the richness and diversity of LOTs was observed across 14 prefecture‐level cities, with a progressive decline toward lower latitudes. This pattern likely reflects the influence of climatic conditions, historical land use, and urbanization intensity. Comparative health assessments demonstrated superior vitality in villages and farmlands (85.47%), scenic spots (90.3%), and wooded areas and plant nurseries (89.42%) compared to residential districts (78.94%). We recommend establishing an integrated ecological‐cultural management strategy for LOT conservation in Guangxi, strengthening in situ protection of underrepresented LOTs, prioritizing the management of culturally unaffiliated taxa, and implementing habitat‐specific strategies to ensure optimal conservation outcomes.

## Graphical abstract

This study comprehensively analyzes the diversity and distribution of over 500 year old large old trees (LOTs) in Guangxi, China, identifying 2630 LOTs from 149 species with a notable latitudinal gradient in richness. Tree vitality was found to be highest in rural and natural habitats like villages and scenic spots, compared to residential areas. The research recommends integrated ecological–cultural management strategies and targeted in situ protection to enhance LOT conservation.

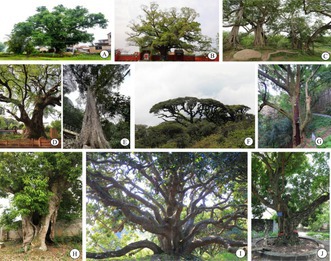

## 1 Introduction

As living monuments are shaped by centuries of biogeographical processes, large old trees (LOTs) constitute a non‐renewable natural capital of exceptional ecological and sociocultural significance (Nolan et al. 2020; Piovesan et al. 2022; Yang et al. 2024). LOTs serve as keystone ecological entities, provide essential habitats for diverse flora and fauna (Lindenmayer 2017), and contribute substantially to the conservation of genetic diversity, nutrient cycling, and long‐term carbon sequestration (Blicharska and Mikusiński 2014; Jin et al. 2024). Moreover, LOTs function as living archives of human history, encapsulating cultural narratives and reflecting the evolution of human society (Huang et al. 2021; Tian et al. 2024). Thus, their preservation is imperative not only for maintaining biodiversity but also for safeguarding intangible cultural heritage.

The accelerated, global decline in LOTs, driven by intensified human activities and land‐use changes, and increasing climatic extremes, has resulted in the degradation of ecosystem integrity and biodiversity (Lindenmayer et al. 2014). This alarming trend has made LOT conservation a critical priority at the intersection of ecological science, cultural preservation, and environmental policies (Le Roux et al. 2014). Worldwide, LOT conservation exhibits distinct regional characteristics shaped by cultural traditions, ecosystem dynamics, and socioeconomic development levels. These factors collectively influence the formulation and implementation of protection strategies across regions. Through cross‐cultural studies, Blicharska and Mikusiński (2014) demonstrated that incorporating the sociocultural values of LOTs, including their esthetic, religious, and historical significance, into conservation policies could enhance the synergy between ecological and cultural preservation efforts. Human activities pose substantial threats to LOTs through direct impacts (e.g., deforestation and land clearing) and indirect disturbances (e.g., landscape fragmentation and urban development). Lindenmayer and Laurance (2017) note that climate change compounds these anthropogenic pressures and creates significant challenges for conservation. Zapponi et al. (2017) analysis of LOT inventories detected a significant connection between tree preservation and cultural landscapes, suggesting the need for integrated conservation approaches that address both the biological and cultural dimensions in Italy. However, the current study has notable limitations. Many countries and regions lack comprehensive data on the composition and distribution of LOTs, and comparative studies across nations or regions remain particularly scarce. These gaps in research constrain the development and implementation of effective conservation strategies at various scales.

China's vast territory includes a significant repository of LOTs, comprising 1140 species across 108 families and 419 genera (Liu, Yang, and Lindenmayer 2019; Huang, Jin, et al. 2020). Chinese scholars have conducted extensive research on LOTs and have addressed diverse aspects, including dendrochronological assessments (Xie et al. 2011; Xu and Zheng 2013), spatial distribution patterns (Bao et al. 2009; Fu 2013; Huang et al. 2015), phytosanitary status and disease management (Liu and Xu 2013; Wang et al. 2016), cultural–historical associations (Qian et al. 2014; Huang, Tian, et al. 2020), and tourism valuation (Yang 2011; Hu et al. 2016). However, current studies have focused predominantly on LOT distribution patterns at metropolitan, county, or site‐specific scales (Jim and Zhang 2013; Xie et al. 2022), with limited exploration of macroscale spatial characteristics (Liu, Lindenmayer, et al. 2019; Huang, Jin, et al. 2020). For example, Liu et al. (2020) studied the regional distribution patterns of one species ( *Ginkgo biloba* ), whereas Huang, Tian, et al. (2020) investigated species diversity within a specific locality (Wuchuan County, Guizhou Province). These geographically or taxonomically constrained approaches present challenges for the development of comprehensive conservation strategies and integrated management plans for LOT resources in China.

Guangxi, a forested region in southern China, hosts an exceptional biodiversity and is one of China's most ecologically significant provinces. However, despite traditional conservation practices and community efforts to preserve numerous LOTs (Huang 2005), expanded urbanization threatens them with human activities and construction products, and natural climatic and biological events. Existing studies on LOTs in Guangxi are largely confined to case analyses at the city or county levels (Zhao et al. 2016). However, knowledge of the species composition and distribution of LOTs in Guangxi is critical for LOT protection, maintenance of regional biodiversity, and guaranteeing ecological integrity in this region. LOTs over 500 years of age are highly related to the local traditional culture, threatened by environmental factors (e.g., natural events and pests) and human activities (e.g., urban development and pollution), and exhibit significant rarity and implicit conservation urgency (Liu, Yang, and Lindenmayer 2019; Liu et al. 2022; Piovesan et al. 2022; Vandekerkhove et al. 2018). Here, we focused on LOTs over 500 years of age in Guangxi. We analyzed their taxonomic and species diversity, and spatial distribution, and assessed their conservation status by integrating publicly available government data with field surveys. Our objectives were (1) to understand the overall family, genus, and species composition of LOTs across Guangxi and examine the spatial distribution and diversity characteristics of these taxa across 14 cities, and (2) to analyze the habitats and health conditions of LOTs, determine the factors influencing their growth and conservation, and provide solutions for protection and management strategies.

## 2 Materials and Methods

### 2.1 Study Area

The study area is the Guangxi Zhuang Autonomous Region (20°54′ N‐26°23′ N, 104°26′ E‐112°03′ E) in southern China, which covers a total area of 237,600 km^2^. Administratively, Guangxi comprises 14 prefecture‐level cities, with a population of approximately 50.47 million (2023). The region has a characteristic basin topography dominated by mountainous hills, with the general terrain sloping from the northwest (maximum elevation: 2141.5 m) to the southeast. Climatically, Guangxi falls within the subtropical monsoon zone, featuring warm temperatures, abundant precipitation (annual range: 723.9–2983.8 mm), and sufficient solar radiation (mean annual temperature: 17.6°C–23.8°C; annual sunshine duration: 1231–2209 h). The soil types are primarily red, yellow, and limestone‐derived soils. As a global biodiversity hotspot, Guangxi harbors 8739 documented species of wild vascular plants (Wei et al. 2023), representing 262 families and 1793 genera. Given this remarkable floristic diversity, Guangxi accounts for 33.46% of China's total wild vascular plant species and ranks third in terms of plant species richness.

### 2.2 Data Collection

Field surveys with publicly available government data were combined to analyze LOTs in 14 prefecture‐level cities in Guangxi (Nanning, Liuzhou, Guilin, Wuzhou, Beihai, Fangchenggang, Qinzhou, Guigang, Yulin, Baise, Hezhou, Hechi, Laibin, and Chongzuo). We referred to the list of LOTs published by the People's Government of the Guangxi Zhuang Autonomous Region (http://www.gxzf.gov.cn/zfwj/zzqrmzfwj_34845/t1509641.shtml), with basic data on LOTs over 500 years of age, such as geographical location, tree age, height, diameter at breast height (DBH), and crown size. In addition, to obtain more accurate tree measurement data, we conducted field surveys between 2023 and 2024 to record the diameter at breast height (DBH), height, and crown width of all LOTs, with particular attention paid to documenting the habitat type and health status of each individual. Accurate determination of LOTs age is crucial; however, as these trees are legally protected, destructive sampling for precise age estimation is not permissible (Briffa 2000; Christopoulou et al. 2022). Therefore, in this study, the ages of some trees were determined based on historical records and interviews with the local elders (Xie et al. 2026). Furthermore, we retrieved published age‐DBH equations for common tree species (e.g., Ficus spp. and *Ginkgo biloba* ) from the literature (Huang, Jin, et al. 2020; Dey et al. 2017) and applied them to estimate the ages of the corresponding LOTs. For species lacking specific equations, the estimates were made using equations derived from closely related species within the same genus or family. The data were supplemented with planting records, historical photographs, records of local development (such as the founding year of the villages), and oral histories provided by residents.

Through field investigations, we recorded the habitat and growth conditions of the LOTs. On the basis of their growth status, the trees were categorized into three groups: “good,” “moderate,” and “poor.” Trees classified as “good” were characterized by vigorous vitality, full canopy structure, a dead branch rate of less than 10% in the crown, absence of main trunk damage or cavities, and minimal signs of human or pest disturbances. The “moderate” category referred to trees with average growth, a dead branch rate of 10%–30% in the crown, minor trunk damage or small cavities, and slight signs of disturbances. Trees categorized as “poor” exhibited slow growth, a dead branch rate exceeding 30% in the crown, large wounds or severe cavities in the main trunk, or significant damage from human activities or pests (Wang et al. 2016).

Tree species were classified into four groups according to size: dominant species (≥ 100 individuals), common species (10–100 individuals), rare species (2–9 individuals), and solitary species (1 individual). Four habitat types were identified based on the tree location: villages and farmlands, wooded areas, plant nurseries, scenic spots, and residential districts. Villages and farmlands are characterized by low density and are dominated by agricultural and natural landscapes, including villages, farmlands, and natural environments. Scenic areas are open spaces designed for public recreation, leisure, and social activities, and encompass scenic areas, parks, and forest parks designed to provide leisure spaces and protect natural ecosystems. Wooded areas and plant nurseries, which typically feature extensive forest vegetation and focus on sustainable forest management and ecological conservation, have been designated for forestry management and timber production. Residential districts include residential neighborhoods and apartments, and are marked by high building density, significant population density, and proximity to shops and entertainment facilities.

### 2.3 Data Analysis

Species diversity characteristics of LOTs in 14 prefecture‐level cities in Guangxi were compared. The species diversity indicators were as follows:

(a) Shannon–Wiener index (Shannon and Weaver 1998) (*H*)

$$H=-\sum \mathrm{pi}\left(\ln\mathrm{pi}\right)$$

where *pi* is the individual proportion of the i‐th species.

(b) Margalef Index (*D*)

$$D=\left(S-1\right)/\mathrm{lnN}$$

where *S* is the number of species and *N* is the total number of individuals.

(c) Pielou's evenness index (Pielou 1966) (*J*)

$$J=\frac{H}{\mathrm{lnS}}$$

where *H* is the Shannon–Wiener index, and S is the total number of species.

To analyze the similarity in LOT composition among different prefecture‐level cities, the Jaccard similarity index (McKinney 2006; Qian et al. 2016) (*C*_*j*_) was employed for a comparative assessment. The Jaccard similarity index formula is as follows:

$$C_{j}=j/\left(a+b-j\right)$$

where *a* is the number of species in the first city, *b* is the number of species in the second city, and *j* is the number of species shared between the two cities.

The importance value (IV) of a species was calculated from its relative frequency (RF), relative abundance (RA), and relative dominance (RD). The calculation formula for IV is as follows:

$$\mathrm{IV}=\left(\mathrm{RF}+\mathrm{RA}+\mathrm{RD}\right)\times 100/3$$

Among them, RF = the frequency of a certain species appearing in 14 prefecture‐level cities/the sum of the frequencies of all species; RA = the number of individuals of a species/the total number of individuals of all species; and RD = the basal area at breast height of a species/the sum of the basal areas at breast height of all species.

To further investigate the relationship between LOT diversity, tree structural attributes, and environmental factors across the 14 prefecture‐level cities, we performed Spearman's correlation analysis (Legendre and Legendre 2012). Tree diversity and structural attributes was evaluated using seven indices: Shannon‐Wiener index (*H*), Margalef index (*D*), number of individuals (NI), Pielou's evenness index (*J*), average tree height (ATH), mean diameter at breast height (MD), and mean crown width (MCW). Five environmental variables were considered: population density (PD), urbanization rate (UR), mean annual temperature (MAT), mean annual precipitation (MAP), and latitude (*L*). These variables were obtained from the Guangxi Statistical Bureau (http://tjj.gxzf.gov.cn/tjsj/tjnj/). R 4.3.3 was used to conduct data analysis.

## 3 Results

### 3.1 Taxonomic Diversity and Structure of LOTs

**FIGURE 1:**
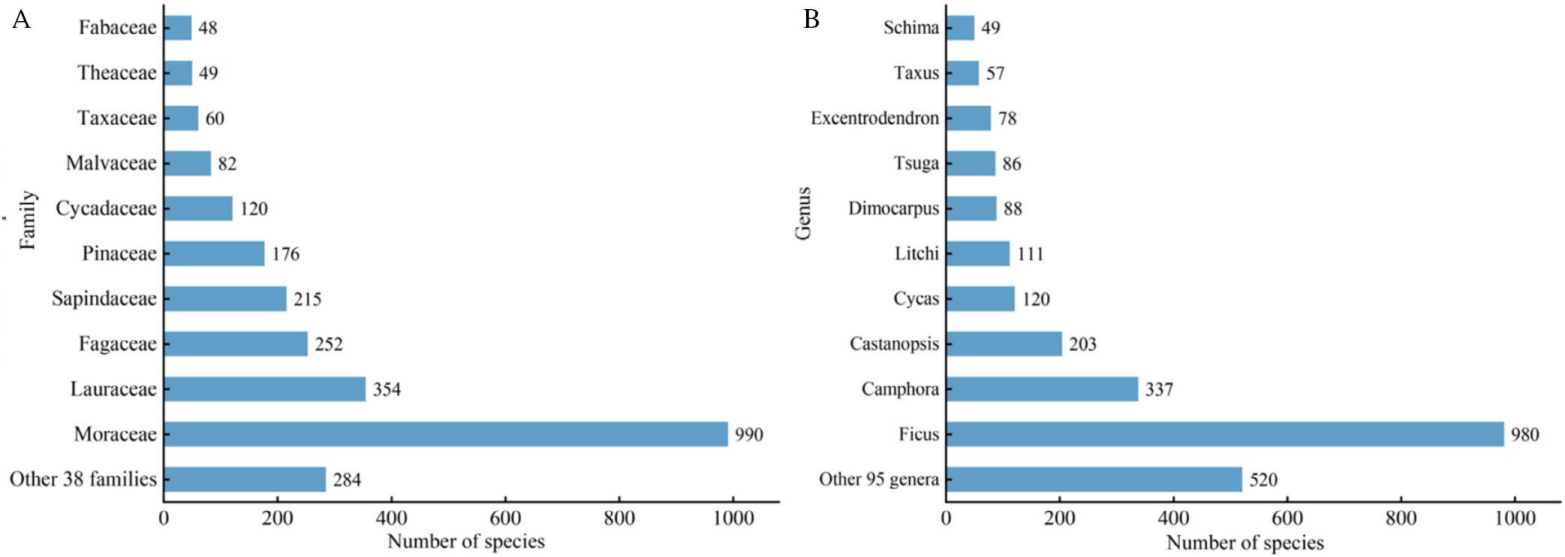
Distribution of large old trees by family and genus in Guangxi.

**FIGURE 2:**
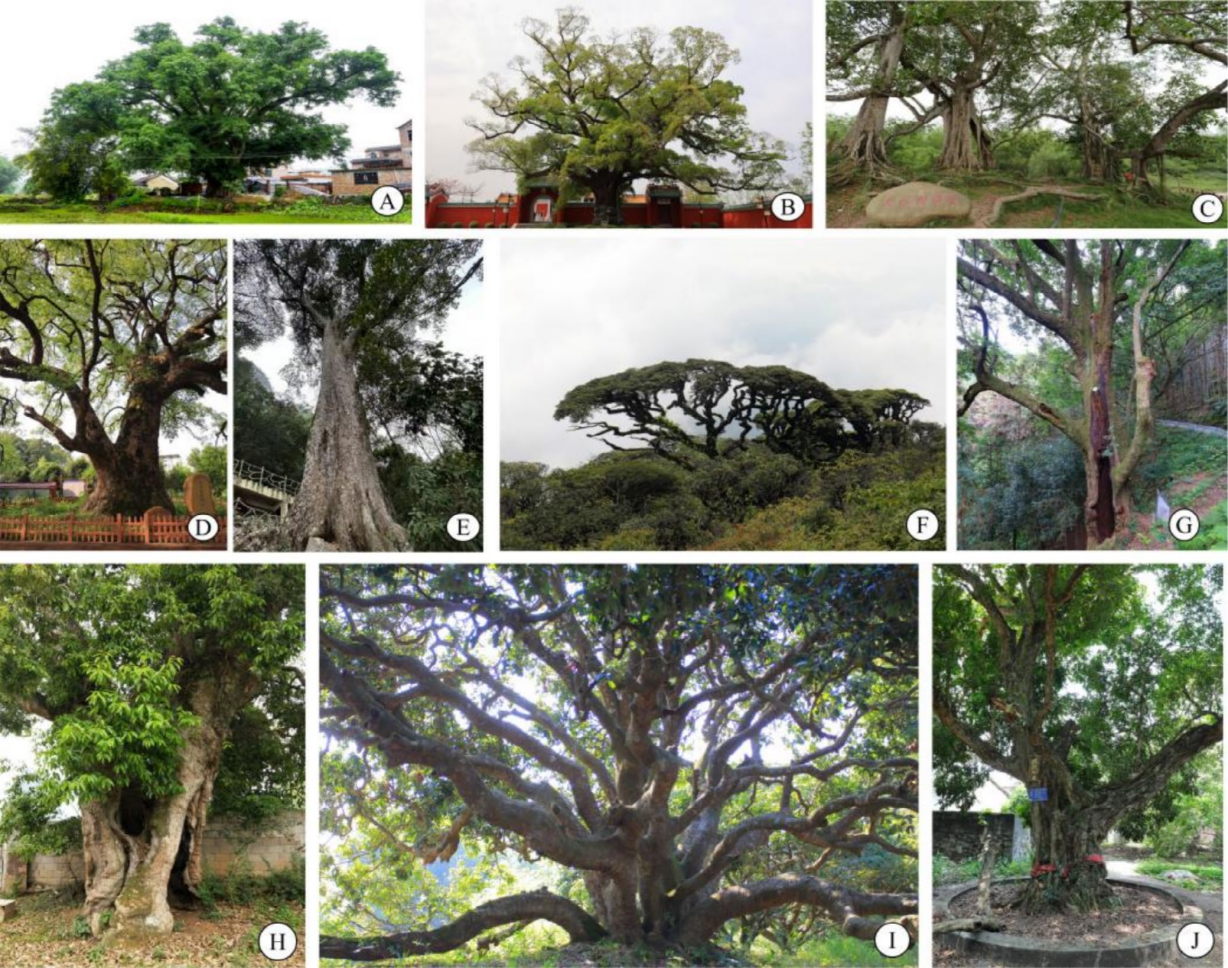
Photographs of common large old trees in Guangxi, South China. (A) *Ficus virens* (Moraceae). (B) *Ficus concinna* (Moraceae). (C) *Ficus altissima* (Moraceae). (D) *Camphora officinarum* (Lauraceae). (E) *Excentrodendron tonkinense* (Malvaceae). (F) *Tsuga chinensis* (Pinaceae). (G) *Castanopsis hystrix* (Fagaceae). (H) *Castanopsis sclerophylla* (Fagaceae). (I) *Litchi chinensis* (Sapindaceae). (J) *Dimocarpus longan* (Sapindaceae). The photographs were obtained by Jiayi Yan, Jianyong Lin, and Gang Hu.

There are 2630 LOT individuals aged over 500 years in Guangxi; these LOTs belong to 149 species, 105 genera, and 48 families. In terms of family composition, the dominant family was Moraceae (37.64%), followed by Lauraceae (13.46%) and Fagaceae (9.58%), whereas the proportion of the remaining families was relatively low, ranging from 0.03% to 8.17% (Figure 1A). At the genus level, *Ficus* (Moraceae), *Camphora* (Lauraceae), and *Castanopsis* (Fagaceae) were predominant (Figure 1B). On the basis of the analysis of relative abundance (RA) (Table S1), *Ficus virens* (421 trees), *Camphora officinarum* (337 trees), *Ficus microcarpa* (211 trees), *Ficus concinna* (210 trees), *Ficus altissima* (128 trees), and *Litchi chinensis* (111 trees) were the dominant species (Figure 2), with a total RA of approximately 53.95%. Twenty‐two common species (10–100 individuals) account for 25.48% of all species. The IV analysis (Table 1) showed that *Ficus virens* ranked first, with an IV of 41.67, which was significantly greater than the values of *Camphora officinarum* (35.0), *Ficus microcarpa* (33.33), and *Ficus concinna* (27.0). The IVs of the remaining 133 species ranged from 1 to 10 (Table S1).

**TABLE 1:** Ten large old trees are arranged in descending order of their importance value index (*IV*).

Species	Family	NI	RF	RA	RD	IV
*Ficus virens*	Moraceae	421	0.79	0.16	0.30	41.67
*Camphora officinarum*	Lauraceae	337	0.79	0.13	0.13	35.00
*Ficus microcarpa*	Moraceae	211	0.79	0.08	0.13	33.33
*Ficus concinna*	Moraceae	210	0.57	0.08	0.16	27.00
*Ficus altissima*	Moraceae	128	0.5	0.05	0.09	21.33
*Dimocarpus longan*	Sapindaceae	88	0.57	0.03	0.01	20.33
*Castanopsis hystrix*	Fagaceae	74	0.57	0.03	0.01	20.33
*Dacrycarpus imbricatus*	Podocarpaceae	24	0.57	0.01	0.01	19.67
*Schima superba*	Theaceae	46	0.5	0.02	0.01	17.67
*Erythrophleum fordii*	Fabaceae	24	0.5	0.01	0.01	17.33

Abbreviations: NI, number of individuals; RF, relative frequency; RA, relative abundance; RD, relative dominance.

**FIGURE 3:**
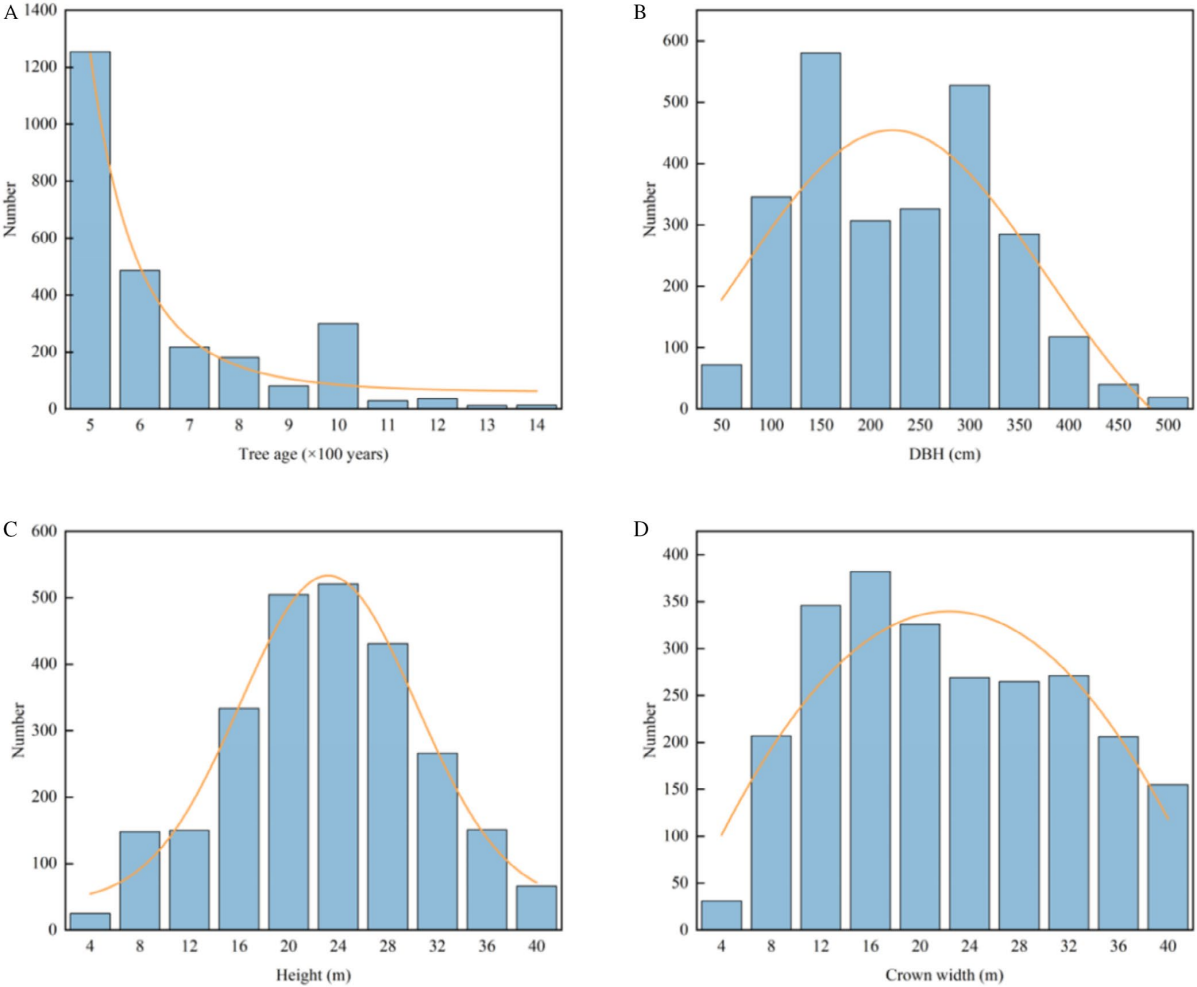
Distribution of large old trees in Guangxi by tree age, DBH, height, and crown width.

The average age of LOTs (≥ 500 years) in Guangxi was 675.84 ± 217.32 years (Figure 3A). The age distribution shows great variation, with a minimum age of 500 years (with 693 trees in this category) and a maximum age of 2300 years, which is the age of the unique tree *Excentrodendron tonkinense* located in the Nonggang National Nature Reserve of Guangxi. The age distribution pattern revealed that the 500–600 years age group represented the highest proportion, with 1254 trees accounting for 47.68% of the total. This group was followed by the 600–700 years old group (486 trees, 18.48%) and the 1000–1100 years old group (300 trees, 11.41%). With the exception of the 1000–1100 years old group, there was a significant negative correlation between tree age and the number of trees, indicating an inverted J‐shaped age distribution.

The average diameter at breast height (DBH) of LOTs over 500 years old is 203.83 ± 99.13 cm (Figure 3B). The largest recorded DBH was 722 cm, which belonged to *Ficus virens* in Gaoguo Village, Gaolong Township, Tianlin County, Baise City. The smallest was 20.7 cm from *Cathaya argyrophylla* in Guangxi Huaping National Nature Reserve, Longsheng County, Guilin City. The DBH distribution indicated that the group with a DBH of 100–150 cm had the largest number of trees (580 trees), accounting for 22.05% of all trees. This group was followed by the 250–300 cm group (528 trees, 20.08%), the 50–100 cm group (346 trees, 13.16%), and the 200–250 cm group (326 trees, 12.40%). There were comparatively fewer trees in the groups with DBH exceeding 400 cm, with fewer than 50 trees in each group, accounting for less than 3% of the total.

The distribution of tree height basically follows a normal distribution, with an average height of 20.82 ± 8.24 m (Figure 3C). The tallest tree was *Parashorea chinensis* in Bama County, Hechi City, Guangxi, reaching 58.8 m, whereas the shortest tree was *Loropetalum chinense*, reaching 4 m. The tree height distribution was mainly concentrated in the 16–20 m (505 trees), 20–24 m (521 trees), and 24–28 m (431 trees) groups, accounting for 55.40% of the total trees. There were 2242 LOTs with heights less than 30 m, accounting for 85.25% of the total, indicating that the heights of most LOTs were below 30 m.

The average crown width of LOTs is 21.77 ± 11.43 m (Figure 3D). Among them, *Ficus virens* had the largest crown width of 77.5 m, whereas *Ficus macrocarpa* had the smallest crown width of only 1.9 m. The crown width distribution was mainly concentrated in the groups of 8–12 m (346 trees), 12–16 m (382 trees), and 16–20 m (326 trees), accounting for 40.08% of the total. As crown width increased, the number of trees gradually decreased.

### 3.2 Spatial Distribution of the Species Diversity of LOTs

**FIGURE 4:**
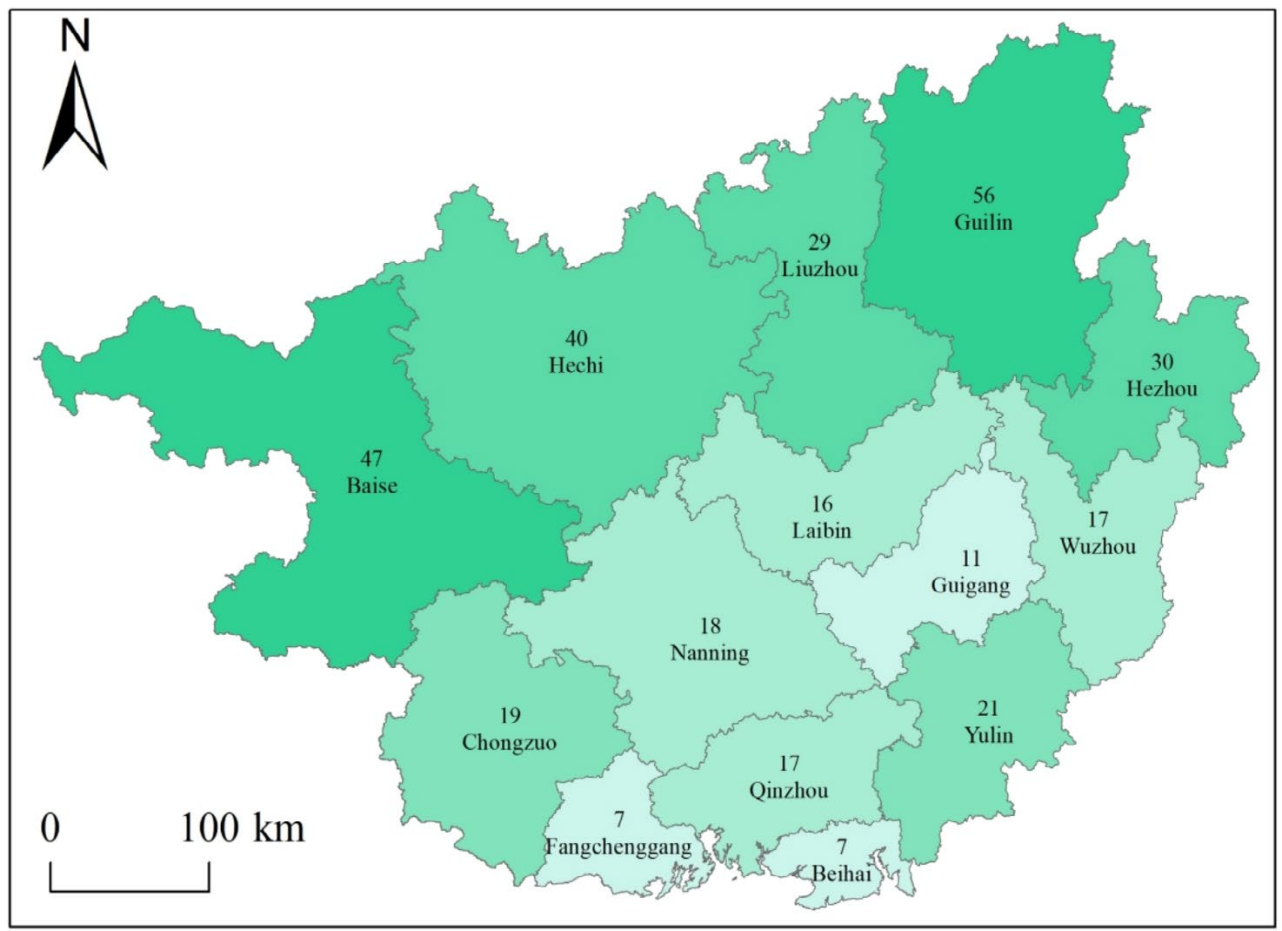
Spatial distribution of the richness of large old trees in 14 prefecture‐level cities in Guangxi.

**FIGURE 5:**
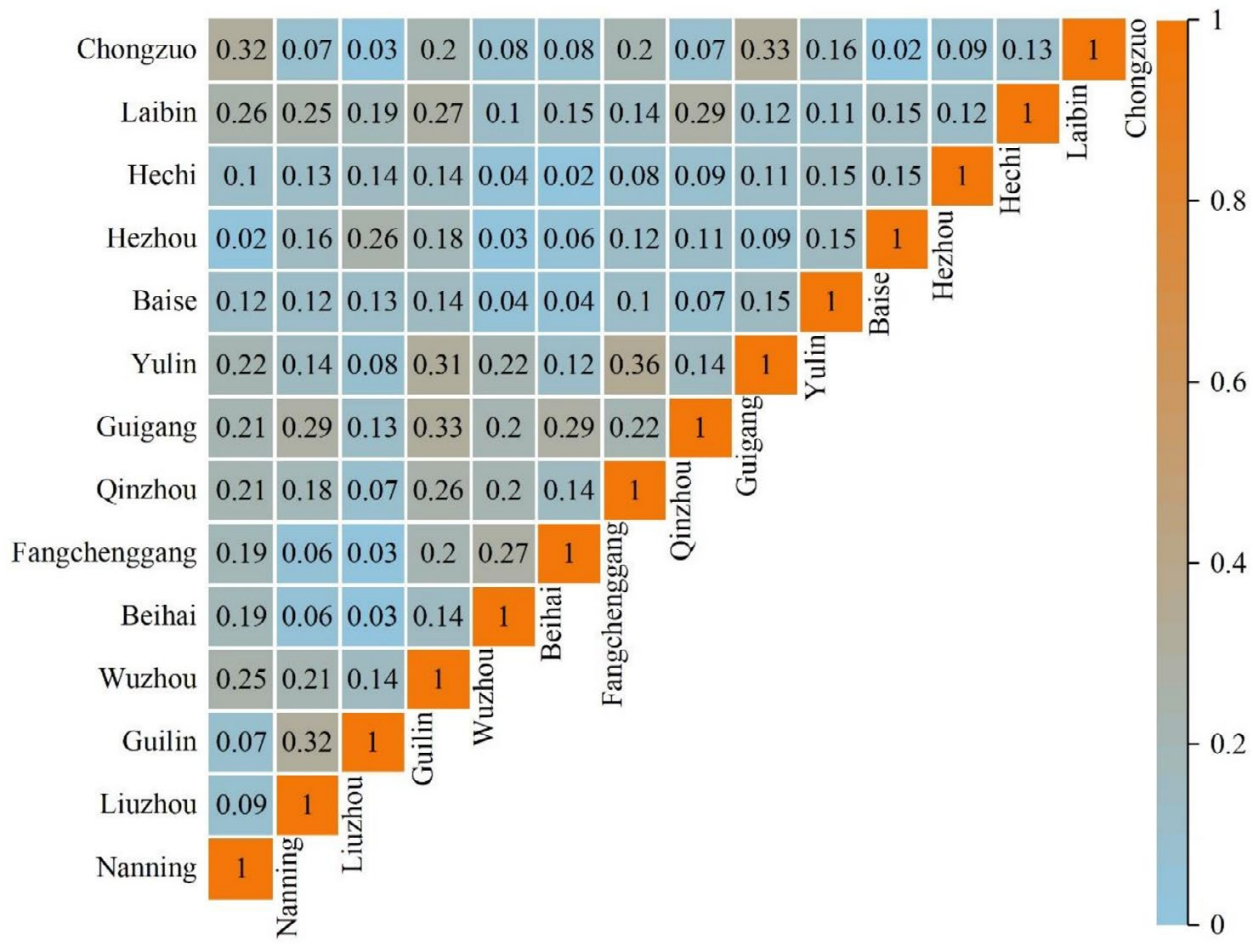
Species similarity distributions for large old trees in 14 prefecture‐level cities in Guangxi.

The distribution of LOTs in 14 prefecture‐level cities showed significant heterogeneity (Figure 4). The results for species similarity (Figure 5) indicated that Qinzhou and Yulin had the highest species similarity (0.36), whereas Nanning and Yulin and Fangchenggang and Hechi had the lowest (0.02). In addition, nearly one‐quarter of the LOTs were distributed in Guilin, which had the most species (56 species), accounting for approximately 37.58% of the total (Table 2). The degree of species overlap among cities was relatively low. Only *Ficus microcarpa* , *Ficus virens*, and *Camphora officinarum* were distributed in the 11 prefecture‐level cities. In terms of the diversity indices, the Shannon–Wiener index values of the 14 prefecture‐level cities range from 1.57 to 2.80, with an average of 2.07. The top three cities were Guilin (2.80), Liuzhou (2.32), and Hezhou (2.31). In contrast, Nanning, a highly urbanized city, ranked second to last in terms of species diversity (1.57), whereas Qinzhou had the lowest value (1.53). The Margalef Index (*D*) varied significantly among cities. Guilin had the highest species richness (8.48), whereas Fangchenggang had the lowest (2.27). The Pielou's evenness index (*J*) did not vary much among the cities, ranging from 0.71 ± 0.14.

**TABLE 2:** Ranking of the 14 cities in descending order according to their Shannon–Wiener index values (*H*).

City	NI	NS	*H*	*D*	*J*
Guilin	656	56	2.80	8.48	0.69
Yulin	64	21	2.50	4.81	0.82
Liuzhou	236	29	2.32	5.12	0.70
Hezhou	260	30	2.31	5.22	0.68
Wuzhou	121	17	2.28	3.34	0.80
Laibin	77	16	2.24	3.45	0.81
Hechi	212	40	2.19	7.28	0.59
Baise	397	47	1.94	7.69	0.50
Beihai	8	7	1.91	2.89	0.98
Chongzuo	168	19	1.88	3.51	0.64
Guigang	44	11	1.87	2.64	0.78
Fangchenggang	14	7	1.67	2.27	0.86
Nanning	217	18	1.57	3.16	0.54
Qinzhou	156	17	1.53	3.17	0.54

Abbreviations: NI, number of individuals; NS, number of species; D, Margalef index. J, Pielou's evenness index.

**FIGURE 6:**
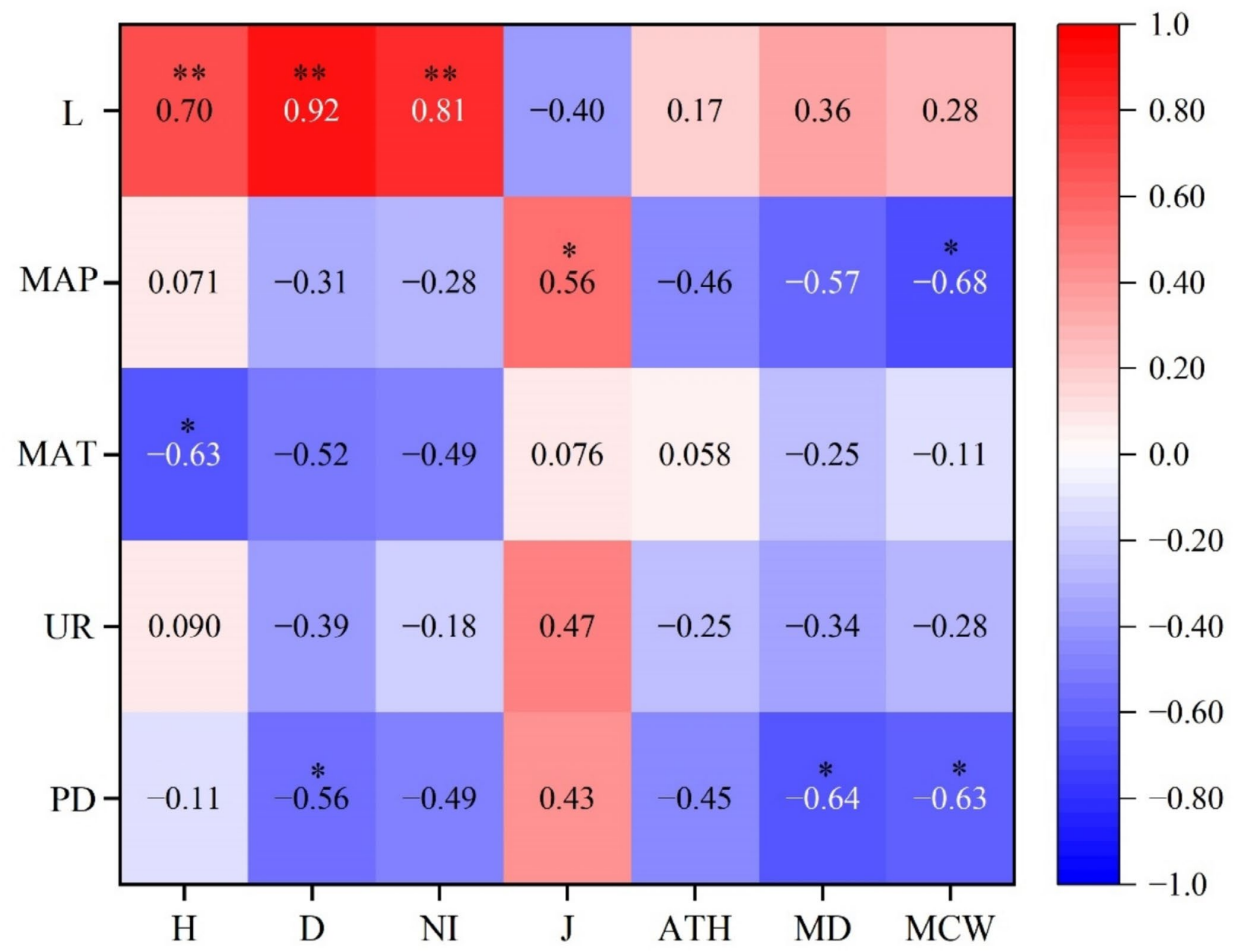
Correlation analysis of tree diversity, structural attributes, and environmental factors for large old trees in Guangxi. *H*, Shannon–Wiener index; *D*, species richness; NI, number of individuals; *J*, Pielou's evenness index; ATH, mean tree height; MD, mean diameter at breast height; MCW, mean crown width; PD, population density; UR, urbanization rate; MAT, mean annual temperature; MAP, annual precipitation; L, latitude. * *ρ* < 0.05; ** *ρ* < 0.01.

Correlation analysis indicated that the Shannon–Wiener index (*H*), species richness index (*D*), and individual number (NI) were significantly positively correlated with latitude (*L*) (*ρ* < 0.01), while the Pielou's evenness index (*J*) and mean annual precipitation (MAP) also exhibited a significant positive correlation (*ρ* < 0.01) (Figure 6). Moreover, the Shannon–Wiener index (*H*) was significantly negatively correlated with mean annual temperature (MAT) (*ρ* < 0.05), and species richness index (D), mean diameter at breast height (MD), and mean crown width (MCW) were similarly negatively correlated with population density (PD) (*ρ* < 0.05). No significant correlations were observed between the remaining environmental factors, such as urbanization rate (UR), and any of the diversity indices examined (Figure 6).

### 3.3 Habitat and Health Status of LOTs

**FIGURE 7:**
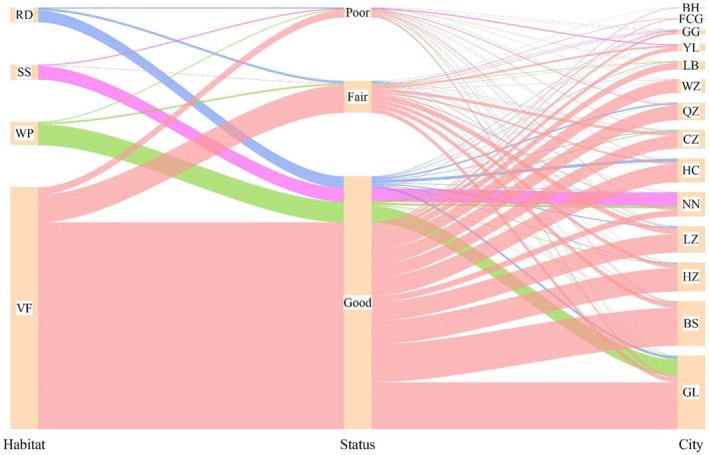
Sankey diagram illustrating the relationships among tree growth status, habitat, and cities in Guangxi. Interrelationships among growth status, habitat, and cities for large old trees in Guangxi. VF, villages and farmlands; WP, wooded areas and plant nurseries; SS, scenic spots; RD, residential districts; GL, Guilin; BS, Baise; HZ, Hezhou; LZ, Liuzhou; NN, Nanning; HC, Hechi; CZ, Chongzuo; QZ, Qinzhou; WZ, Wuzhou; LB, Laibin; YL, Yulin; GG, Guigang; FCG, Fangchenggang; BH, Beihai.

As shown in Figure 6, 85.70% of the LOTs are in “good” condition, whereas 10.91% are in “moderate” condition, and only 3.38% are in “poor” condition. The habitat distribution (Figure 7) revealed that the majority of LOTs were concentrated in villages and farmlands, with 2155 trees (81.94%), significantly surpassing the number of trees located in other habitat types. Wooded areas and plant nurseries ranked second, with 208 trees (7.91%), whereas scenic spots (134 trees, 5.1%) and residential districts (133 trees, 5.06%) had the lowest distribution, accounting for less than 11% of the total. In terms of the distribution across prefecture‐level cities (Figure 7), Guilin has the highest number of LOTs (656 trees), with the most individuals in “good” condition (586 trees) and the highest number in “poor” condition (23 trees) among all cities. In Fangchenggang, among its 14 LOTs, the proportion of trees in “moderate” condition is the highest (6 trees, 42.85%). The number of LOTs in “poor” conditions in prefecture‐level cities, except for Guilin, ranged from 0 to 11.

## 4 Discussion

### 4.1 Taxonomic Diversity of LOTs Over 500 Years Old in Guangxi

This study identified 2630 individual LOTs in Guangxi that belonged to 149 species, 105 genera, and 48 families (Table S1 and Table S3). The *Ficus genus* was represented significantly more than the other taxa. China has a long‐standing tradition of *Ficus* tree cultivation (Guan 2013), and these trees have a particular cultural and religious significance (Chen et al. 2023; Ling et al. 2023). This phenomenon of *Ficus* veneration is common across many tropical regions of Asia (Pokharel and Pokharel 2021). Our findings revealed that large old *Ficus* trees are located predominantly in ethnic minority settlements, where local cultural practices and beliefs contribute to their preservation, which is consistent with the observations of Huang, Tian, et al. (2020). Furthermore, the *Ficus* genus has exceptional longevity (Chakraborty et al. 2022), a biological characteristic that enhances the survival of trees, such as ancient specimens. Guangxi, located in a low‐latitude subtropical zone, experiences high summer temperatures and intense sunlight. The towering and dense canopies of *Ficus* trees provide natural shade, making them ideal gathering locations for cooling and social activities. This functional benefit is one reason for the widespread cultivation and protection of large Ficus trees in southern China (Suthar 2022; Xie, Yan, et al. 2024).

The genus *Camphora* ranks second most abundant group after *Ficus* in terms of population size. Studies have demonstrated that *Camphora* species exhibit exceptional tolerance to both biotic (e.g., pests and pathogens) and abiotic stresses (e.g., environmental pollution and extreme climatic events), which enables their persistence for centuries in spatially constrained urban environments (Jim 2005; Zhang and Jim 2014). These adaptive traits have led to their widespread use in urban greening projects since the 19th century (Stubbs 2012). Furthermore, their broad ecological amplitude facilitates their successful cultivation across tropical and temperate regions in China (Zhang et al. 2017), which may explain the abundance of ancient *Camphora* specimens in Guangxi. The extensive distribution of *Castanopsis* in Guangxi has resulted from multiple interacting factors. First, southern China's geographic position protected Guangxi from direct Quaternary glaciation effects (Hsu 1983), which created a refuge for thermophilic taxa, such as *Castanopsis*, and prevented the extinction patterns observed in Europe and regions north of China's Qinling Mountains (Kaul and Abbe 1984; Kvaček and Walther 1989). Second, the region's characteristic subtropical monsoon climate, featuring warm and humid conditions and mild winters, aligns perfectly with *Castanopsis's* thermal preferences and facilitates natural population regeneration (Kaul et al. 1986). Additionally, as keystone species in subtropical forests (Manos and Stanford 2001), Fagaceae plants establish mutualistic relationships with animal dispersers (e.g., squirrels and birds) through their nut crops, which significantly increases seed dispersal efficiency (Cheuk and Fischer 2021).

*Ficus*, *Camphora*, and *Castanopsis* were the dominant genera in the LOT cohort older than 500 years. The extended lifespans of these trees can be attributed to both cultural reversal and fundamental physiological adaptations that confer resilience to long‐term biotic and abiotic stressors. Emerging evidence indicates that tree longevity is associated with genetic enhancements in DNA repair pathways, stress priming, and immunological responses (Liu et al. 2025). The production of specialized secondary metabolites is integral to this adaptation. *Camphora* species synthesizes camphor and related terpenoids with documented antifungal and insecticidal activities, whereas *Ficus* species contain high levels of flavonoids and phenolic compounds. These metabolites have played a significant role in mitigating biotic and environmental challenges, thereby facilitating survival over millennia (Cui et al. 2024). The synergy between these evolved resistance mechanisms and persistent cultural safeguarding solidified the status of *Ficus*, *Camphora*, and *Castanopsis* as the principal long‐lived LOTs in Guangxi.

Except for the Moraceae, Lauraceae, and Fagaceae families, the remaining 45 families account for only 0.03%–8.17% of the total number of LOTs in Guangxi, which indicates a skewed distribution pattern. We attribute this disparity to anthropogenic selection pressures, which significantly reduced the number of families compared with the three dominant families. For example, Dipterocarpaceae species have significantly declined due to illegal logging and lowland forest habitat destruction (e.g., conversion to palm oil plantations) (Luo et al. 2022). Similarly, Polygalaceae species are facing near extinction in the wild owing to excessive harvesting for medicinal purposes (Jiang et al. 2016). These contrasting patterns reveal the underlying mechanisms governing LOT distribution. While natural selection confers stress resistance to certain taxa, human‐mediated directional selection (through either conservation or exploitation) may increase the survival advantages of specific groups and ultimately shapes the skewed distribution pattern at regional scales.

### 4.2 Spatial Pattern of LOTs in Guangxi

The spatial distribution of LOTs across Guangxi's 14 prefecture‐level cities exhibited pronounced heterogeneity, indicating the historical interplay between natural selection and human activity. This distribution pattern is consistent with the findings of Milton et al. (1994) in Panama, who highlighted the pivotal role of stand disturbance history in structuring the species composition and mortality dynamics of LOTs. The Jaccard similarity index among cities shows extreme differentiation (0.02–0.36), which is consistent with the spatial differences in the number of LOTs (7–56) and the diversity index (*H* = 1.53–2.80). This multidimensional spatial heterogeneity may be attributed to the combined mechanism of action of natural and human factors (Xie, Chen, et al. 2024; Yang et al. 2024).

The unique geographical environment of Guilin–a landscape surrounded by the Lijiang River and a warm and humid subtropical monsoon climate (Hu et al. 2024) provides a diverse growing environment for LOTs, supports the coexistence of different tree species and biodiversity, and thus presents a unique landscape and ecological value. Beihai, Fangchenggang, and Qinzhou are coastal towns that are vulnerable to the effects of the marine climate and high soil salinity. High‐salinity water and typhoons adversely affect tree growth and survival (Halil 2024), which in turn affects the growth of certain tree species. Consequently, the types and numbers of LOTs in these areas were relatively small, which resulted in a habitat diversity index of only 0.03 between Guilin and these coastal towns (Figure 5), further demonstrating the filtering effect of the geographical environment on the composition of species.

Historical development processes may also have influenced the number of species in each city. Guilin is an ancient city in China that dates back to the 5th century BC (Sofield et al. 2019). This strong historical background has enabled Guilin to preserve many LOTs. In contrast, Beihai, Fangchenggang, and Qinzhou are important parts of Beibu Gulf's urban agglomeration. Historically, these have served as important coastal trading ports with significant human activity (Chen et al. 2024). Such long‐term developmental activities have restricted the natural growth and preservation of large old trees in these areas (Liu et al. 2020) and resulted in the lowest numbers of LOTs (8–14) and species (7) among all cities. Notably, Nanning and Qinzhou present a special “paradox of medium quantity ‐ low diversity.” Although they had a medium number of individuals, their Shannon–Wiener indices (1.57 and 1.53) were the lowest in the region. Table S2 shows that the proportion of single dominant species (Cycadaceae and Sapindaceae) in these two cities exceeded 50%, resulting in a Pielou's evenness index as low as 0.54. The observed LOT simplification could have resulted from municipal greening policies that, during rapid urban expansion, prioritize species for their economic utility and adaptive resilience (Jim 2017). The age structure of these LOTs serves as a distinct archive of historical human impacts. Notably, an outlier in the 1000–1100 years age group (Figure 3A), which disrupts the typical reverse J‐shaped distribution, may signal a pivotal period in past socioeconomic development. The survival of these millennials underscores the crucial role of culturally embedded protection, sustained through enduring beliefs and customary practices (Huang, Tian, et al. 2020; Salick et al. 2007).

Urbanization can also affect the spatial distribution of LOTs (Chi et al. 2020; Li and Zhang 2021). Nanning is used as an example. As the capital city of Guangxi, its Shannon–Wiener index (*H* = 1.57) is 0.5, which is lower than the overall diversity index of Guangxi (*H* = 2.07). Environmental pressures, such as land hardening and heat island effects caused by urbanization (Czaja et al. 2020), may limit root expansion and nutrient acquisition in some vulnerable LOTs (Huang, Jin, et al. 2020) and thus reduce species diversity in this area. Additionally, within the disciplinary consensus of landscape architecture, the historically documented transplantation of LOTs, given their characteristically low success rates, is considered a plausible contributing factor to the diminished species diversity of LOTs found in Nanning. Previous studies indicate that LOTs, particularly mature individuals, frequently suffer from compromised root architecture and diminished resilience following transplantation, culminating in mortality years after the event (Jim 2013). Similarly, research on LOTs in Guangzhou, China, indicates that the practice of relocating trees during urban regeneration and infrastructure projects can inflict lasting damage to LOT stocks (Jim 2005). However, empirical data on transplantation‐related mortality specific to Nanning's LOTs are currently lacking. Future research avenues such as mining historical landscape archives and conducting longitudinal studies on transplanted cases may provide more direct evidence. This transplantation legacy may represent another key driver behind the lower species diversity and richness of LOTs in Nanning than those in other cities.

### 4.3 Suggestions for the Conservation of LOTs in Guangxi

The growth conditions of LOTs in Guangxi exhibited significant variations across four habitat types: villages and farmland, wooded areas, plant nurseries, scenic spots, and residential districts. Over 80% of LOTs are located in villages and farmland, and more than 80% of them exhibit “good” condition. This finding aligns closely with Huang, Tian, et al.'s (2020) report on ethnic minority areas in Guizhou, corroborating the idea that a culturally driven protection mechanism represents a widespread and effective model in southwestern China. This further confirms the effectiveness of culturally driven conservation mechanisms in rural areas, as supported by Salick et al. (2007), Andersson and Östlund (2004), Hu et al. (2011), and Hernández‐Morcillo et al. (2013). Notably, 14.52% of the trees in villages and farmland are in “moderate” or “poor” condition, a phenomenon which may be linked to soil compaction around tree roots due to road construction for convenience (Coffin et al. 2021) or microhabitat degradation caused by localized human disturbances, such as tourism development or agricultural expansion. Similar interference effects have been documented in studies on trees in traditional conservation areas, such as the stress on LOTs in Ethiopian church forests due to human activities (Wassie et al. 2010) and the decline in tree health associated with microhabitat changes caused by habitat fragmentation in urban fringe areas (Chen et al. 2018). In addition, while only 133 LOTs are found in residential districts, more than 20% of them are in “moderate” or “poor” condition, which is consistent with the stress pressures typical of such habitats (Chi et al. 2020; Tian et al. 2024). Trees in residential districts often face restricted root activity due to ground hardening (Liu et al. 2022), which negatively affects their growth.

At the regional scale, Guilin, the core distribution area of LOTs (with 656 trees), may benefit from the influence of natural and human factors on high‐density individuals (Hu et al. 2024). This city has the largest number of “poor”‐grade individuals (23 trees) possibly related to the physiological decline in LOTs. Martínez‐Vilalta et al. (2007) found a significant age‐related decline in growth efficiency (biomass production per unit leaf area) for Scots pine ( *Pinus sylvestris* ), with older trees (> 200 years) exhibiting less than 40% of the growth efficiency observed in younger individuals. This decline was attributed to the synergistic effects of hydraulic limitations (reduced whole‐tree hydraulic conductance and stomatal canopy conductance) and diminished nitrogen availability in the needles. These findings align with the broader ecological paradigm of metabolic decline in aging trees. In support of this universal pattern, field data from Guilin City, China demonstrated that 25.5% of LOTs exceeded 800 years of age, further corroborating the general relationship between advanced tree senescence and reduced metabolic vigor. In addition, in Fangchenggang, the proportion of LOTs in “moderate” condition (42.85% vs. the average of 14.64%) is relatively high, but there are no individuals in “poor” condition. We speculate that this phenomenon may be related to the dual effects of the coastal environment. A mild and humid climate and abundant precipitation are conducive to tree growth (Vieira et al. 2020). However, the uneven seasonal distribution of precipitation, sea‐level rise, soil salinization, and extreme weather events, such as strong winds and storms, may have a negative impact on tree growth (Vieira et al. 2020; Halil 2024). The Guangxi government plays an important role in LOT conservation. There are no more than 11 LOTs in “poor” conditions in any city, except for Guilin, which reflects unique conservation achievements.

Based on the heterogeneous distribution of LOTs in Guangxi, we propose implementing differentiated conservation policies to formulate targeted protection and management plans. First, it is necessary to maintain the stability of the dominant families, genera, and species to prevent ecological vulnerability caused by the existence of a single dominant species. For rare tree species and extremely small populations, urgent ex‐situ conservation measures should be initiated, detailed archives established, and translocation assessments necessary. For dominant tree species, while maintaining population stability, the potential impacts of overexpansion on the ecological balance must be monitored, to influence LOT diversity from a state of “skewed survival” toward one of “balanced coexistence.” Second, because Guangxi is inhabited by ethnic minorities, its unique cultural belief system effectively protects most LOTs. Management resources should be concentrated preferentially on LOTs that may not benefit from cultural protection. For instance, LOTs growing in residential areas or along roadsides, where community‐based conservation is absent, may not benefit from the voluntary protection mechanisms typically associated with village Feng Shui forests or religious sites. Finally, habitat‐specific conservation plans must be formulated at the regional adaptive management level. For example, the stress resistance of LOTs in coastal areas should be enhanced. For example, three‐dimensional laser scanning technology can be used to assess the mechanical and structural vulnerability of trees (Calders et al. 2020). Elastic support frames can be installed for high‐risk individuals, preventive pruning of tree crowns can be performed, and early warning systems for pests and diseases can be established. Biological control techniques such as anthracnose and borers can be used to prevent and control diseases, such as anthracnose and borers (Zeming et al. 1991). Soil acidification in karst regions has been a long‐standing challenge (Yun et al. 2016). In particular, for LOTs that rely on specific soil conditions (such as *Excentrodendron tonkinense* and *Garcinia paucinervis*), soil acidification may lead to the loss of nutrients, activation of heavy metals, and imbalances in microbial communities, which threaten the health and survival of trees. Thus, a calcium cycle monitoring and regulation system can be constructed. When the pH value of the root soil of the calcium‐rich tree species decreased to a threshold of 6.5 (Altland and Jeong 2016), certain additives (such as ultrafine dolomite powder) can be used for restoration to increase the nutrient use efficiency. Within the framework of these conservation policies, systematic monitoring of LOTs health can be conducted at regular intervals (e.g., every 3–5 years). This includes tracking the population dynamics of rare LOTs and measuring habitat variables, such as rhizosphere soil pH, heavy metal bioavailability, and microbial community structure. In coastal regions frequently affected by typhoons, the critical parameters to assess include the rate of support structure installation for high‐risk trees, adoption of preventive canopy pruning, and the accuracy of pest and disease forecasting. In karst areas, emphasis should be placed on monitoring the rate at which the rhizosphere soil pH of calcicolous species recovers to levels above 6.5, along with the corresponding improvements in soil elemental conditions. In general, it is recommended that the government and forestry bureau establish a comprehensive evaluation and long‐term monitoring mechanism, incorporate local beliefs into protection policies, and construct an ecological‐cultural‐driven LOT protection model to provide a basis for the conservation of LOTs in Guangxi, South China.

We acknowledge the limitations of this study. The reliance on public government data and field surveys suggests that our inventory may be limited, particularly for remote locations or less dominant species. Furthermore, age estimates obtained from government records, published age–DBH equations, and historical corroboration are of limited reliability. Future research may be supported by stronger cross‐regional partnerships to develop long‐term monitoring networks and from exploring the integration of novel approaches such as remote‐sensing‐based individual/community mapping and DNA barcoding into conservation practices.

## 5 Conclusions

To the best of our knowledge, this is the first study to evaluate the diversity, distribution, and preservation strategies of LOTs aged over 500 years in Guangxi, South China. Our survey revealed 2630 individuals belonging to 149 species, 105 genera, and 48 families. The *Ficus* genus dominated this assemblage, representing 37.64% of all samples, with *Ficus virens* and *Camphora officinarum* emerging as the dominant species distributed across 11 prefecture‐level cities. The age structure followed an inverted J‐shaped distribution, with most trees exhibiting stem diameters of 150–300 cm. Spatial analysis revealed heterogeneous distribution patterns across all 14 prefecture‐level cities, likely resulting from combined climatic, historical, and urbanization factors. We recommend implementing an integrated ecological–cultural management approach for LOT conservation in Guangxi. Key strategies include (1) enhanced in situ protection of underrepresented species, (2) prioritized management of culturally unprotected species, and (3) habitat‐specific adaptation measures. Future research should investigate climate change and land‐use impacts, while establishing cross‐regional collaboration frameworks and long‐term monitoring systems to optimize conservation outcomes.

## Author Contributions

**Jiayi Yan:** formal analysis (equal), investigation (equal), methodology (equal), visualization (equal), writing – original draft (equal), writing – review and editing (equal). **Jianyong Lin:** investigation (equal), resources (equal), visualization (equal), writing – review and editing (equal). **Aihua Wang:** investigation (equal), methodology (equal), writing – review and editing (equal). **Yadong Qie:** investigation (equal), methodology (equal), writing – review and editing (equal). **Cong Hu:** data curation (equal), investigation (equal), visualization (equal), writing – original draft (equal). **Zhonghua Zhang:** conceptualization (equal), data curation (equal), formal analysis (equal), funding acquisition (equal), investigation (equal), methodology (equal), project administration (equal), visualization (equal), writing – original draft (equal), writing – review and editing (equal). **Gang Hu:** conceptualization (equal), data curation (equal), formal analysis (equal), funding acquisition (equal), investigation (equal), methodology (equal), project administration (equal), visualization (equal), writing – original draft (equal), writing – review and editing (equal).

## Funding

This work was supported by Guangxi Natural Science Foundation (2021GXNSFFA196005, 2025JJA130183), the National Natural Science Foundation of China (31960275) and the Special Funding for Guangxi Bagui Young Top Talents Program (to Zhonghua Zhang).

## Conflicts of Interest

The authors declare no conflicts of interest.

## Supporting information


**Data S1:** ece373043‐sup‐0001‐Supinfo01.docx.

**Data S2:** ece373043‐sup‐0002‐Supinfo02.xlsx.

**Data S3:** ece373043‐sup‐0003‐Supinfo03.docx.

## Data Availability

All the required data are uploaded as Supporting Information.
