# Supplementary material for: Conserving Large Old Trees in Guangxi, South China: Diversity, Distribution, and Preservation Strategies
Source: Ecol Evol. 2026 Feb 12;16(2):e73043. doi: 10.1002/ece3.73043 (PMC12900623; doi:10.1002/ece3.73043)
Supplement: Supplementary file 1 — Data S1: ece373043‐sup‐0001‐Supinfo01.docx. [file ECE3-16-e73043-s002.docx]

**Supplementary Materials**

**Conserving Large Old Trees in Guangxi, South China: Diversity, Distribution, and Preservation Strategies**

Jiayi Yan^1,2#^, Jianyong Lin^3#^, Aihua Wang^1,2^, Yadong Qie^1^, Cong Hu^1^, Zhonghua Zhang^1,2*^, Gang Hu^1,2*^

^1^ Key Laboratory of Environment Change and Resources Use in Beibu Gulf, Ministry of Education, Nanning Normal University, Nanning 530100, China

^2^ Guangxi Key Laboratory of Earth Surface Processes and Intelligent Simulation, Nanning Normal University, Nanning 530100, China

^3^ Key Laboratory of Central South Fast-growing Timber Cultivation of Forestry Ministry of China, Guangxi Forestry Research Institute, Nanning 530028, China

Correspondence: Zhonghua Zhang (gxtczzh@126.com); Gang Hu (ahhugang@126.com)

^#^ These authors contributed equally to this work.

**Table S1.**  Families and species with many large old trees in Guangxi, South China. BA, basal area at breast height (m^2^); RF, relative frequency; RA, relative abundance; RD, relative dominance; IV, importance value.

| **Species** | **Family** | **BA(m^2^)** | **Abundance** | **RF** | **RA** | **RD** | **IV** |
| --- | --- | --- | --- | --- | --- | --- | --- |
| *Ficus virens* | Moraceae | 2617.16 | 421 | 0.79 | 0.16 | 0.30 | 41.39 |
| *Camphora officinarum* | Lauraceae | 1139.30 | 337 | 0.79 | 0.13 | 0.13 | 34.76 |
| *Ficus microcarpa* | Moraceae | 1111.17 | 211 | 0.79 | 0.08 | 0.13 | 33.05 |
| *Ficus concinna* | Moraceae | 1391.50 | 210 | 0.57 | 0.08 | 0.16 | 26.96 |
| *Ficus altissima* | Moraceae | 799.03 | 128 | 0.5 | 0.05 | 0.09 | 21.3 |
| *Cycas pectinata* | Cycadaceae | 23.30 | 119 | 0.14 | 0.05 | 0.00 | 6.36 |
| *Litchi chinensis* | Sapindaceae | 157.68 | 111 | 0.29 | 0.04 | 0.02 | 11.53 |
| *Dimocarpus longan* | Sapindaceae | 80.80 | 88 | 0.57 | 0.03 | 0.01 | 20.47 |
| *Tsuga chinensis* | Pinaceae | 22.61 | 86 | 0.14 | 0.03 | 0.00 | 5.94 |
| *Excentrodendron tonkinense* | Malvaceae | 151.20 | 78 | 0.21 | 0.03 | 0.02 | 8.7 |
| *Castanopsis hystrix* | Fagaceae | 106.33 | 74 | 0.57 | 0.03 | 0.01 | 20.39 |
| *Castanopsis sclerophylla* | Fagaceae | 82.84 | 58 | 0.14 | 0.02 | 0.01 | 5.81 |
| *Taxus wallichiana var.mairei* | Taxaceae | 39.23 | 57 | 0.36 | 0.02 | 0.00 | 12.78 |
| *Schima superba* | Theaceae | 54.64 | 46 | 0.5 | 0.02 | 0.01 | 17.46 |
| *Nothotsuga longibracteata* | Pinaceae | 17.61 | 38 | 0.21 | 0.01 | 0.00 | 7.69 |
| *Erythrophleum fordii* | Fabaceae | 30.99 | 24 | 0.5 | 0.01 | 0.00 | 17.09 |
| *Osmanthus fragrans* | Oleaceae | 17.21 | 24 | 0.29 | 0.01 | 0.00 | 9.89 |
| *Dacrycarpus imbricatus* | Podocarpaceae | 26.00 | 24 | 0.57 | 0.01 | 0.00 | 19.45 |
| *Cathaya argyrophylla* | Pinaceae | 4.31 | 22 | 0.14 | 0.01 | 0.00 | 5.06 |
| *Ginkgo biloba* | Ginkgoaceae | 48.45 | 20 | 0.07 | 0.01 | 0.01 | 2.82 |
| *Castanopsis chinensis* | Fagaceae | 37.61 | 19 | 0.29 | 0.01 | 0.00 | 9.91 |
| *Castanopsis carlesii* | Fagaceae | 31.39 | 18 | 0.29 | 0.01 | 0.00 | 9.87 |
| *Bischofia polycarpa* | Phyllanthaceae | 67.73 | 17 | 0.36 | 0.01 | 0.01 | 12.38 |
| *Carya sinensis* | Juglandaceae | 51.55 | 15 | 0.14 | 0.01 | 0.01 | 5.15 |
| *Castanopsis eyrei* | Fagaceae | 8.95 | 15 | 0.07 | 0.01 | 0.00 | 2.6 |
| *Averrhoa carambola* | Oxalidaceae | 11.18 | 14 | 0.5 | 0.01 | 0.00 | 16.89 |
| *Castanea henryi* | Fagaceae | 20.49 | 14 | 0.14 | 0.01 | 0.00 | 5.02 |
| *Liquidambar formosana* | Altingiaceae | 29.57 | 11 | 0.29 | 0 | 0.00 | 9.77 |
| *Ilex rotunda* | Aquifoliaceae | 10.21 | 11 | 0.29 | 0 | 0.00 | 9.7 |
| *Pistacia chinensis* | Anacardiaceae | 34.02 | 10 | 0.21 | 0 | 0.00 | 7.4 |
| *Cunninghamia lanceolata* | Cupressaceae | 15.62 | 10 | 0.21 | 0 | 0.00 | 7.33 |
| *Ficus racemosa* | Moraceae | 54.68 | 9 | 0.43 | 0 | 0.01 | 14.61 |
| *Quercus acutissima* | Fagaceae | 11.56 | 9 | 0.43 | 0 | 0.00 | 14.44 |
| *Quercus glauca* | Fagaceae | 14.69 | 9 | 0.21 | 0 | 0.00 | 7.31 |
| *Zelkova schneideriana* | Ulmaceae | 13.72 | 8 | 0.21 | 0 | 0.00 | 7.3 |
| *Garcinia paucinervis* | Clusiaceae | 6.82 | 8 | 0.21 | 0 | 0.00 | 7.27 |
| *Dracontomelon duperreanum* | Anacardiaceae | 60.25 | 8 | 0.14 | 0 | 0.01 | 5.09 |
| *Pinus kwangtungensis* | Pinaceae | 1.56 | 7 | 0.07 | 0 | 0.00 | 2.48 |
| *Styphnolobium japonicum* | Fabaceae | 13.32 | 7 | 0.21 | 0 | 0.00 | 7.28 |
| *Boniodendron minus* | Sapindaceae | 34.02 | 7 | 0.14 | 0 | 0.00 | 4.86 |
| *Castanopsis fargesii* | Fagaceae | 3.64 | 7 | 0.14 | 0 | 0.00 | 4.86 |
| *Acer coriaceifolium* | Sapindaceae | 6.24 | 7 | 0.07 | 0 | 0.00 | 2.49 |
| *Castanopsis tibetana* | Fagaceae | 6.77 | 6 | 0.14 | 0 | 0.00 | 4.86 |
| *Antiaris toxicaria* | Moraceae | 24.39 | 6 | 0.29 | 0 | 0.00 | 9.69 |
| *Pinus massoniana* | Pinaceae | 7.38 | 6 | 0.14 | 0 | 0.00 | 4.87 |
| *Fagus longipetiolata* | Fagaceae | 3.32 | 6 | 0.07 | 0 | 0.00 | 2.47 |
| *Pterocarya stenoptera* | Juglandaceae | 18.91 | 5 | 0.07 | 0 | 0.00 | 2.52 |
| *Pseudotsuga sinensis* | Pinaceae | 4.26 | 5 | 0.07 | 0 | 0.00 | 2.46 |
| *Phoebe bournei* | Lauraceae | 8.03 | 5 | 0.21 | 0 | 0.00 | 7.24 |
| *Bischofia javanica* | Phyllanthaceae | 22.35 | 5 | 0.29 | 0 | 0.00 | 9.67 |
| *Carya cathayensis* | Juglandaceae | 7.91 | 5 | 0.07 | 0 | 0.00 | 2.47 |
| *Quercus variabilis* | Fagaceae | 6.25 | 5 | 0.14 | 0 | 0.00 | 4.85 |
| *Diospyros decandra* | Ebenaceae | 4.09 | 5 | 0.07 | 0 | 0.00 | 2.46 |
| *Toona ciliata* | Meliaceae | 15.68 | 4 | 0.14 | 0 | 0.00 | 4.87 |
| *Castanopsis faberi* | Fagaceae | 9.49 | 4 | 0.21 | 0 | 0.00 | 7.23 |
| *Bombax ceiba* | Malvaceae | 19.15 | 4 | 0.21 | 0 | 0.00 | 7.27 |
| *Celtis sinensis* | Cannabaceae | 9.27 | 4 | 0.14 | 0 | 0.00 | 4.85 |
| *Vitex quinata* | Lamiaceae | 4.84 | 4 | 0.21 | 0 | 0.00 | 7.21 |
| *Gleditsia sinensis* | Fabaceae | 6.74 | 4 | 0.14 | 0 | 0.00 | 4.84 |
| *Liriodendron chinense* | Magnoliaceae | 2.16 | 3 | 0.14 | 0 | 0.00 | 4.81 |
| *Keteleeria davidiana var.calcarea* | Pinaceae | 3.98 | 3 | 0.14 | 0 | 0.00 | 4.81 |
| *Choerospondias axillaris* | Anacardiaceae | 14.21 | 3 | 0.21 | 0 | 0.00 | 7.23 |
| *Zenia insignis* | Fabaceae | 6.96 | 3 | 0.14 | 0 | 0.00 | 4.83 |
| *Keteleeria pubescens* | Pinaceae | 8.05 | 3 | 0.14 | 0 | 0.00 | 4.83 |
| *Elaeocarpus sylvestris* | Elaeocarpaceae | 3.68 | 3 | 0.21 | 0 | 0.00 | 7.19 |
| *Phoebe calcarea* | Lauraceae | 4.59 | 3 | 0.07 | 0 | 0.00 | 2.44 |
| *Keteleeria davidiana* | Pinaceae | 6.73 | 3 | 0.14 | 0 | 0.00 | 4.83 |
| *Lysidice rhodostegia* | Fabaceae | 7.08 | 3 | 0.21 | 0 | 0.00 | 7.21 |
| *Schima argentea* | Theaceae | 2.15 | 3 | 0.07 | 0 | 0.00 | 2.43 |
| *Hovenia acerba* | Rhamnaceae | 2.27 | 3 | 0.21 | 0 | 0.00 | 7.19 |
| *Quercus fabri* | Fagaceae | 3.29 | 2 | 0.14 | 0 | 0.00 | 4.8 |
| *Prunus dulcis* | Rosaceae | 7.57 | 2 | 0.07 | 0 | 0.00 | 2.43 |
| *Aphananthe aspera* | Cannabaceae | 1.87 | 2 | 0.14 | 0 | 0.00 | 4.79 |
| *Ilex chinensis* | Aquifoliaceae | 1.74 | 2 | 0.14 | 0 | 0.00 | 4.79 |
| *Photinia bodinieri* | Rosaceae | 6.01 | 2 | 0.07 | 0 | 0.00 | 2.43 |
| *Artocarpus parvus* | Moraceae | 2.05 | 2 | 0.14 | 0 | 0.00 | 4.79 |
| *Adenanthera microsperma* | Fabaceae | 3.44 | 2 | 0.14 | 0 | 0.00 | 4.8 |
| *Cephalotaxus hainanensis* | Cephalotaxaceae | 2.80 | 2 | 0.14 | 0 | 0.00 | 4.8 |
| *Ulmus parvifolia* | Ulmaceae | 2.19 | 2 | 0.07 | 0 | 0.00 | 2.41 |
| *Podocarpus macrophyllus* | Podocarpaceae | 1.98 | 2 | 0.14 | 0 | 0.00 | 4.79 |
| *Ligustrum lucidum* | Oleaceae | 4.58 | 2 | 0.14 | 0 | 0.00 | 4.8 |
| *Syzygium levinei* | Myrtaceae | 2.01 | 2 | 0.14 | 0 | 0.00 | 4.79 |
| *Alstonia scholaris* | Apocynaceae | 6.65 | 2 | 0.14 | 0 | 0.00 | 4.81 |
| *Sinosideroxylon pedunculatum* | Sapotaceae | 3.95 | 2 | 0.14 | 0 | 0.00 | 4.8 |
| *Manglietia aromatica* | Magnoliaceae | 5.30 | 2 | 0.14 | 0 | 0.00 | 4.81 |
| *Lithocarpus corneus* | Fagaceae | 3.75 | 2 | 0.07 | 0 | 0.00 | 2.42 |
| *Castanopsis indica* | Fagaceae | 3.81 | 2 | 0.07 | 0 | 0.00 | 2.42 |
| *Keteleeria fortunei* | Pinaceae | 4.06 | 2 | 0.07 | 0 | 0.00 | 2.42 |
| *Saraca dives* | Fabaceae | 4.42 | 2 | 0.07 | 0 | 0.00 | 2.42 |
| *Nageia nagi* | Podocarpaceae | 2.84 | 2 | 0.07 | 0 | 0.00 | 2.42 |
| *Cupressus funebris* | Cupressaceae | 1.99 | 1 | 0.07 | 0 | 0.00 | 2.4 |
| *Machilus leptophylla* | Lauraceae | 1.45 | 1 | 0.07 | 0 | 0.00 | 2.4 |
| *Platycladus orientalis* | Cupressaceae | 2.06 | 1 | 0.07 | 0 | 0.00 | 2.4 |
| *Sassafras tzumu* | Lauraceae | 0.52 | 1 | 0.07 | 0 | 0.00 | 2.4 |
| *Litsea glutinosa* | Lauraceae | 1.27 | 1 | 0.07 | 0 | 0.00 | 2.4 |
| *Platyosprion platycarpum* | Fabaceae | 1.79 | 1 | 0.07 | 0 | 0.00 | 2.4 |
| *Ailanthus altissima* | Simaroubaceae | 1.77 | 1 | 0.07 | 0 | 0.00 | 2.4 |
| *Calocedrus macrolepis* | Cupressaceae | 3.77 | 1 | 0.07 | 0 | 0.00 | 2.41 |
| *Ficus hookeriana* | Moraceae | 9.98 | 1 | 0.07 | 0 | 0.00 | 2.43 |
| *Aphananthe cuspidata* | Cannabaceae | 1.84 | 1 | 0.07 | 0 | 0.00 | 2.4 |
| *Quercus disciformis* | Fagaceae | 3.37 | 1 | 0.07 | 0 | 0.00 | 2.41 |
| *Artocarpus styracifolius* | Moraceae | 1.84 | 1 | 0.07 | 0 | 0.00 | 2.4 |
| *Fraxinus griffithii* | Oleaceae | 0.77 | 1 | 0.07 | 0 | 0.00 | 2.4 |
| *Lindera megaphylla* | Lauraceae | 2.59 | 1 | 0.07 | 0 | 0.00 | 2.4 |
| *Sloanea sinensis* | Elaeocarpaceae | 0.57 | 1 | 0.07 | 0 | 0.00 | 2.4 |
| *Morus cathayana* | Moraceae | 1.04 | 1 | 0.07 | 0 | 0.00 | 2.4 |
| *Dalbergia hupeana* | Fabaceae | 1.13 | 1 | 0.07 | 0 | 0.00 | 2.4 |
| *Xanthophyllum hainanense* | Polygalaceae | 0.46 | 1 | 0.07 | 0 | 0.00 | 2.4 |
| *Elaeocarpus limitaneus* | Elaeocarpaceae | 0.38 | 1 | 0.07 | 0 | 0.00 | 2.4 |
| *Loropetalum chinense* | Hamamelidaceae | 0.44 | 1 | 0.07 | 0 | 0.00 | 2.4 |
| *Keteleeria fortunei var.cyclolepis* | Pinaceae | 2.12 | 1 | 0.07 | 0 | 0.00 | 2.4 |
| *Craibiodendron stellatum* | Ericaceae | 1.00 | 1 | 0.07 | 0 | 0.00 | 2.4 |
| *Corylopsis sinensis* | Hamamelidaceae | 0.54 | 1 | 0.07 | 0 | 0.00 | 2.4 |
| *Tarennoidea wallichii* | Rubiaceae | 1.61 | 1 | 0.07 | 0 | 0.00 | 2.4 |
| *Cryptomeria japonica var.sinensis* | Cupressaceae | 1.13 | 1 | 0.07 | 0 | 0.00 | 2.4 |
| *Chukrasia tabularis* | Meliaceae | 2.94 | 1 | 0.07 | 0 | 0.00 | 2.4 |
| *Castanea seguinii* | Fagaceae | 1.15 | 1 | 0.07 | 0 | 0.00 | 2.4 |
| *Manglietia fordiana* | Magnoliaceae | 6.38 | 1 | 0.07 | 0 | 0.00 | 2.42 |
| *Ormosia pubescens* | Fabaceae | 1.77 | 1 | 0.07 | 0 | 0.00 | 2.4 |
| *Cephalotaxus fortunei* | Cephalotaxaceae | 0.77 | 1 | 0.07 | 0 | 0.00 | 2.4 |
| *Aphanamixis polystachya* | Meliaceae | 1.61 | 1 | 0.07 | 0 | 0.00 | 2.4 |
| *Diospyros japonica* | Ebenaceae | 1.34 | 1 | 0.07 | 0 | 0.00 | 2.4 |
| *Alphonsea mollis* | Annonaceae | 1.21 | 1 | 0.07 | 0 | 0.00 | 2.4 |
| *Mallotus repandus* | Euphorbiaceae | 0.83 | 1 | 0.07 | 0 | 0.00 | 2.4 |
| *Glyptostrobus pensilis* | Cupressaceae | 1.09 | 1 | 0.07 | 0 | 0.00 | 2.4 |
| *Syzygium nervosum* | Myrtaceae | 2.50 | 1 | 0.07 | 0 | 0.00 | 2.4 |
| *Cycas szechuanensis* | Cycadaceae | 0.08 | 1 | 0.07 | 0 | 0.00 | 2.39 |
| *Malania oleifera* | Olacaceae | 0.35 | 1 | 0.07 | 0 | 0.00 | 2.39 |
| *Pouteria annamensis* | Sapotaceae | 0.77 | 1 | 0.07 | 0 | 0.00 | 2.4 |
| *Manilkara hexandra* | Sapotaceae | 1.21 | 1 | 0.07 | 0 | 0.00 | 2.4 |
| *Aglaia lawii* | Meliaceae | 1.04 | 1 | 0.07 | 0 | 0.00 | 2.4 |
| *Parashorea chinensis* | Dipterocarpaceae | 1.50 | 1 | 0.07 | 0 | 0.00 | 2.4 |
| *Lagerstroemia caudata* | Lythraceae | 1.12 | 1 | 0.07 | 0 | 0.00 | 2.4 |
| *Syzygium euonymifolium* | Myrtaceae | 1.67 | 1 | 0.07 | 0 | 0.00 | 2.4 |
| *Machilus wenshanensis* | Lauraceae | 2.22 | 1 | 0.07 | 0 | 0.00 | 2.4 |
| *Diospyros eriantha* | Ebenaceae | 0.82 | 1 | 0.07 | 0 | 0.00 | 2.4 |
| *Ilex pentagona* | Aquifoliaceae | 1.09 | 1 | 0.07 | 0 | 0.00 | 2.4 |
| *Machilus rehderi* | Lauraceae | 2.25 | 1 | 0.07 | 0 | 0.00 | 2.4 |
| *Toona sinensis* | Meliaceae | 2.99 | 1 | 0.07 | 0 | 0.00 | 2.4 |
| *Lindera communis* | Lauraceae | 0.79 | 1 | 0.07 | 0 | 0.00 | 2.4 |
| *Helicia cochinchinensis* | Proteaceae | 0.94 | 1 | 0.07 | 0 | 0.00 | 2.4 |
| *Morella rubra* | Myricaceae | 2.84 | 1 | 0.07 | 0 | 0.00 | 2.4 |
| *Crateva religiosa* | Capparaceae | 0.92 | 1 | 0.07 | 0 | 0.00 | 2.4 |
| *Juniperus chinensis* | Cupressaceae | 1.06 | 1 | 0.07 | 0 | 0.00 | 2.4 |
| *Aesculus assamica* | Sapindaceae | 1.89 | 1 | 0.07 | 0 | 0.00 | 2.4 |
| *Handeliodendron bodinieri* | Sapindaceae | 0.48 | 1 | 0.07 | 0 | 0.00 | 2.4 |
| *Celtis biondii* | Cannabaceae | 1.91 | 1 | 0.07 | 0 | 0.00 | 2.4 |
| *Madhuca pasquieri* | Sapotaceae | 0.98 | 1 | 0.07 | 0 | 0.00 | 2.4 |
| *Phoebe sheareri* | Lauraceae | 1.33 | 1 | 0.07 | 0 | 0.00 | 2.4 |

**Table S2.** Distribution and abundance of large old trees across 14 prefecture-level cities in Guangxi, South China.

| **Species** | **Nanning** | **Liuzhou** | **Guilin** | **Wuzhou** | **Beihai** | **Fangchenggang** | **Qinzhou** | **Guigang** | **Yulin** | **Baise** | **Hezhou** | **Hechi** | **Laibin** | **Chongzuo** |
| --- | --- | --- | --- | --- | --- | --- | --- | --- | --- | --- | --- | --- | --- | --- |
| *Ficus virens* | 6 | 20 | 1 | 5 |  |  | 6 | 16 | 5 | 233 |  | 94 | 21 | 14 |
| *Camphora officinarum* | 2 | 31 | 198 | 18 | 1 | 1 |  | 5 | 4 |  | 64 | 3 | 10 |  |
| *Ficus microcarpa* | 8 | 20 | 50 | 16 | 1 |  | 18 | 10 | 8 | 24 |  | 48 | 8 |  |
| *Ficus concinna* |  | 76 | 32 | 26 |  |  | 1 |  | 1 |  | 58 | 2 | 14 |  |
| *Ficus altissima* | 28 |  |  |  | 1 | 4 | 11 |  | 2 | 33 |  |  |  | 49 |
| *Cycas pectinata* | 117 |  |  |  |  |  |  |  |  |  |  |  |  | 2 |
| *Litchi chinensis* |  |  |  |  |  |  | 95 |  | 10 | 4 |  |  |  | 2 |
| *Dimocarpus longan* | 10 | 3 |  | 12 |  |  | 8 |  | 15 | 9 |  | 2 |  | 29 |
| *Tsuga chinensis* |  |  | 83 |  |  |  |  |  |  |  |  |  | 3 |  |
| *Excentrodendron tonkinense* | 10 |  |  |  |  |  |  |  |  | 17 |  |  |  | 51 |
| *Castanopsis hystrix* |  | 27 | 14 | 19 |  |  |  |  | 1 | 2 | 9 | 1 |  | 1 |
| *Castanopsis sclerophylla* |  |  | 2 |  |  |  |  |  |  |  | 56 |  |  |  |
| *Taxus wallichiana var.mairei* |  | 3 | 45 |  |  |  |  |  |  |  | 1 | 2 | 6 |  |
| *Schima superba* |  | 20 | 12 | 3 |  |  | 2 | 1 |  |  | 7 | 1 |  |  |
| *Nothotsuga longibracteata* | 3 |  | 33 |  |  |  |  |  |  |  |  |  | 2 |  |
| *Erythrophleum fordii* | 1 |  |  | 9 | 1 | 1 | 5 | 1 | 6 |  |  |  |  |  |
| *Osmanthus fragrans* |  |  | 15 | 1 |  |  |  |  | 1 |  | 7 |  |  |  |
| *Dacrycarpus imbricatus* |  | 8 | 2 | 3 |  | 1 | 1 | 4 |  |  | 3 |  | 2 |  |
| *Cathaya argyrophylla* |  |  | 21 |  |  |  |  |  |  |  |  |  | 1 |  |
| *Ginkgo biloba* |  |  | 20 |  |  |  |  |  |  |  |  |  |  |  |
| *Castanopsis chinensis* |  |  | 13 | 1 |  |  |  |  |  | 4 | 1 |  |  |  |
| *Castanopsis carlesii* |  |  | 7 |  |  |  |  |  |  | 4 | 6 | 1 |  |  |
| *Bischofia polycarpa* |  | 1 | 8 |  |  |  |  |  |  | 1 | 6 |  | 1 |  |
| *Carya sinensis* |  |  |  |  |  |  |  |  |  | 8 |  | 7 |  |  |
| *Castanopsis eyrei* | 15 |  |  |  |  |  |  |  |  |  |  |  |  |  |
| *Averrhoa carambola* | 5 |  |  | 3 |  | 1 |  | 1 |  | 1 |  |  | 2 | 1 |
| *Castanea henryi* |  | 1 | 13 |  |  |  |  |  |  |  |  |  |  |  |
| *Liquidambar formosana* |  | 2 | 3 |  |  |  |  |  |  | 3 |  | 3 |  |  |
| *Ilex rotunda* | 1 |  |  |  |  |  | 2 |  | 1 |  | 7 |  |  |  |
| *Pistacia chinensis* |  |  | 2 |  |  |  |  |  |  | 4 | 4 |  |  |  |
| *Cunninghamia lanceolata* |  | 2 | 5 |  |  |  |  |  |  |  |  |  | 3 |  |
| *Ficus racemosa* | 1 |  |  | 1 |  |  |  |  |  | 3 |  | 2 | 1 | 1 |
| *Quercus acutissima* |  | 1 | 4 |  |  |  |  | 1 |  | 1 | 1 |  | 1 |  |
| *Quercus glauca* |  |  | 2 |  |  |  |  |  |  | 3 | 0 | 4 |  |  |
| *Zelkova schneideriana* |  |  | 2 |  |  |  |  |  |  | 4 | 2 |  |  |  |
| *Garcinia paucinervis* | 5 |  |  |  |  |  |  |  |  |  |  | 1 |  | 2 |
| *Dracontomelon duperreanum* |  |  |  |  |  |  |  |  | 1 |  |  |  |  | 7 |
| *Pinus kwangtungensis* |  |  | 7 |  |  |  |  |  |  |  |  |  |  |  |
| *Styphnolobium japonicum* |  |  | 1 |  |  |  |  |  |  |  | 5 | 1 |  |  |
| *Boniodendron minus* |  |  |  |  |  |  |  |  |  |  | 1 | 6 |  |  |
| *Castanopsis fargesii* | 1 |  | 6 |  |  |  |  |  |  |  |  |  |  |  |
| *Acer coriaceifolium* |  |  |  |  |  |  |  |  |  |  | 7 |  |  |  |
| *Castanopsis tibetana* |  | 1 | 5 |  |  |  |  |  |  |  |  |  |  |  |
| *Antiaris toxicaria* |  |  |  |  | 2 |  | 1 |  | 1 |  |  |  |  | 2 |
| *Pinus massoniana* |  | 3 |  |  |  |  |  | 3 |  |  |  |  |  |  |
| *Fagus longipetiolata* |  |  | 6 |  |  |  |  |  |  |  |  |  |  |  |
| *Pterocarya stenoptera* |  |  | 5 |  |  |  |  |  |  |  |  |  |  |  |
| *Pseudotsuga sinensis* |  | 5 |  |  |  |  |  |  |  |  |  |  |  |  |
| *Phoebe bournei* |  | 1 | 3 |  |  |  |  |  |  |  | 1 |  |  |  |
| *Bischofia javanica* |  |  |  | 1 |  |  |  |  | 1 | 2 |  |  |  | 1 |
| *Carya cathayensis* |  |  |  |  |  |  |  |  |  |  |  | 5 |  |  |
| *Quercus variabilis* |  |  | 4 |  |  |  |  |  |  | 1 |  |  |  |  |
| *Diospyros decandra* |  |  |  |  |  | 5 |  |  |  |  |  |  |  |  |
| *Toona ciliata* |  |  | 2 |  |  |  |  |  |  |  |  | 2 |  |  |
| *Castanopsis faberi* |  | 1 | 1 |  |  |  |  |  |  |  | 2 |  |  |  |
| *Bombax ceiba* | 2 |  |  |  |  |  |  |  |  |  |  |  | 1 | 1 |
| *Celtis sinensis* |  |  |  |  |  |  |  |  |  | 3 | 1 |  |  |  |
| *Vitex quinata* |  | 1 | 2 |  |  |  |  | 1 |  |  |  |  |  |  |
| *Gleditsia sinensis* |  |  | 1 |  |  |  |  |  |  |  | 3 |  |  |  |
| *Liriodendron chinense* |  | 1 | 2 |  |  |  |  |  |  |  |  |  |  |  |
| *Keteleeria davidiana var.calcarea* |  |  |  |  |  |  |  |  |  |  | 1 | 2 |  |  |
| *Choerospondias axillaris* |  | 1 | 1 |  |  |  |  | 1 |  |  |  |  |  |  |
| *Zenia insignis* |  |  |  |  |  |  |  |  |  |  |  | 2 |  | 1 |
| *Keteleeria pubescens* |  |  | 2 |  |  |  |  |  |  |  |  | 1 |  |  |
| *Elaeocarpus sylvestris* |  |  |  |  |  |  | 1 |  |  | 1 | 1 |  |  |  |
| *Phoebe calcarea* |  |  |  |  |  |  |  |  |  | 3 |  |  |  |  |
| *Keteleeria davidiana* |  | 1 |  |  |  |  |  |  |  | 2 |  |  |  |  |
| *Lysidice rhodostegia* |  |  |  |  |  |  |  |  | 1 | 1 |  | 1 |  |  |
| *Schima argentea* |  |  | 3 |  |  |  |  |  |  |  |  |  |  |  |
| *Hovenia acerba* |  | 1 |  |  |  |  | 1 |  |  |  | 1 |  |  |  |
| *Quercus fabri* |  |  |  |  |  |  |  |  |  | 1 | 1 |  |  |  |
| *Prunus dulcis* |  |  |  |  |  |  |  |  |  | 2 |  |  |  |  |
| *Aphananthe aspera* |  | 1 | 1 |  |  |  |  |  |  |  |  |  |  |  |
| *Ilex chinensis* |  |  | 1 |  |  |  |  |  |  |  | 1 |  |  |  |
| *Photinia bodinieri* |  |  |  |  |  |  |  |  |  |  |  | 2 |  |  |
| *Artocarpus parvus* |  |  |  |  |  |  | 1 |  | 1 |  |  |  |  |  |
| *Adenanthera microsperma* |  |  |  |  |  |  |  |  | 1 |  |  |  |  | 1 |
| *Cephalotaxus hainanensis* |  |  |  |  |  |  |  |  | 1 | 1 |  |  |  |  |
| *Ulmus parvifolia* |  |  |  |  |  |  |  |  |  | 2 |  |  |  |  |
| *Podocarpus macrophyllus* |  | 1 | 1 |  |  |  |  |  |  |  |  |  |  |  |
| *Ligustrum lucidum* |  |  |  |  |  |  |  |  |  |  | 1 | 1 |  |  |
| *Syzygium levinei* |  |  |  |  |  |  | 1 |  |  |  |  |  |  | 1 |
| *Alstonia scholaris* |  |  |  |  |  |  |  |  | 1 |  |  |  |  | 1 |
| *Sinosideroxylon pedunculatum* |  |  |  |  |  |  |  |  |  | 1 |  | 1 |  |  |
| *Manglietia aromatica* |  |  |  |  |  |  |  |  |  | 1 |  | 1 |  |  |
| *Lithocarpus corneus* |  |  |  |  |  |  |  |  |  | 2 |  |  |  |  |
| *Castanopsis indica* |  |  |  |  |  |  |  |  |  |  |  | 2 |  |  |
| *Keteleeria fortunei* |  |  |  |  |  |  |  |  |  |  |  | 2 |  |  |
| *Saraca dives* |  |  |  |  |  |  |  |  |  | 2 |  |  |  |  |
| *Nageia nagi* |  |  | 2 |  |  |  |  |  |  |  |  |  |  |  |
| *Cupressus funebris* |  |  | 1 |  |  |  |  |  |  |  |  |  |  |  |
| *Machilus leptophylla* |  |  | 1 |  |  |  |  |  |  |  |  |  |  |  |
| *Platycladus orientalis* |  |  | 1 |  |  |  |  |  |  |  |  |  |  |  |
| *Sassafras tzumu* |  |  | 1 |  |  |  |  |  |  |  |  |  |  |  |
| *Litsea glutinosa* |  |  |  |  |  |  |  |  |  | 1 |  |  |  |  |
| *Platyosprion platycarpum* |  |  | 1 |  |  |  |  |  |  |  |  |  |  |  |
| *Ailanthus altissima* |  |  |  |  |  |  |  |  |  |  |  | 1 |  |  |
| *Calocedrus macrolepis* |  |  |  |  |  |  |  |  |  | 1 |  |  |  |  |
| *Ficus hookeriana* |  |  |  |  |  |  |  |  |  | 1 |  |  |  |  |
| *Aphananthe cuspidata* | 1 |  |  |  |  |  |  |  |  |  |  |  |  |  |
| *Quercus disciformis* |  |  |  |  |  |  |  |  |  | 1 |  |  |  |  |
| *Artocarpus styracifolius* |  |  | 1 |  |  |  |  |  |  |  |  |  |  |  |
| *Fraxinus griffithii* |  |  | 1 |  |  |  |  |  |  |  |  |  |  |  |
| *Lindera megaphylla* |  |  |  |  |  |  |  |  |  | 1 |  |  |  |  |
| *Sloanea sinensis* |  |  |  |  |  |  |  |  |  |  |  | 1 |  |  |
| *Morus cathayana* |  |  | 1 |  |  |  |  |  |  |  |  |  |  |  |
| *Dalbergia hupeana* |  | 1 |  |  |  |  |  |  |  |  |  |  |  |  |
| *Xanthophyllum hainanense* |  |  |  |  |  |  | 1 |  |  |  |  |  |  |  |
| *Elaeocarpus limitaneus* |  |  | 1 |  |  |  |  |  |  |  |  |  |  |  |
| *Loropetalum chinense* |  |  | 1 |  |  |  |  |  |  |  |  |  |  |  |
| *Keteleeria fortunei var.cyclolepis* |  |  |  |  |  |  |  |  |  |  |  |  | 1 |  |
| *Craibiodendron stellatum* |  |  |  | 1 |  |  |  |  |  |  |  |  |  |  |
| *Corylopsis sinensis* |  | 1 |  |  |  |  |  |  |  |  |  |  |  |  |
| *Tarennoidea wallichii* |  |  |  |  |  |  |  |  |  |  |  |  |  | 1 |
| *Cryptomeria japonica var.sinensis* |  |  | 1 |  |  |  |  |  |  |  |  |  |  |  |
| *Chukrasia tabularis* |  |  |  |  |  |  |  |  |  | 1 |  |  |  |  |
| *Castanea seguinii* |  |  |  |  |  |  |  |  |  |  |  | 1 |  |  |
| *Manglietia fordiana* |  |  |  |  |  |  |  |  |  |  |  | 1 |  |  |
| *Ormosia pubescens* |  |  |  |  |  | 1 |  |  |  |  |  |  |  |  |
| *Cephalotaxus fortunei* |  |  |  |  |  |  |  |  |  |  |  | 1 |  |  |
| *Aphanamixis polystachya* |  |  |  |  |  |  |  |  |  |  |  | 1 |  |  |
| *Diospyros japonica* |  |  |  |  |  |  |  |  |  |  |  | 1 |  |  |
| *Alphonsea mollis* |  |  |  |  |  |  |  |  |  | 1 |  |  |  |  |
| *Mallotus repandus* |  |  |  |  |  |  |  |  | 1 |  |  |  |  |  |
| *Glyptostrobus pensilis* | 1 |  |  |  |  |  |  |  |  |  |  |  |  |  |
| *Syzygium nervosum* |  |  |  |  |  |  | 1 |  |  |  |  |  |  |  |
| *Cycas szechuanensis* |  |  |  |  |  |  |  |  |  |  | 1 |  |  |  |
| *Malania oleifera* |  |  |  |  |  |  |  |  |  | 1 |  |  |  |  |
| *Pouteria annamensis* |  |  |  |  | 1 |  |  |  |  |  |  |  |  |  |
| *Manilkara hexandra* |  |  |  |  | 1 |  |  |  |  |  |  |  |  |  |
| *Aglaia lawii* |  |  |  |  |  |  |  |  |  | 1 |  |  |  |  |
| *Parashorea chinensis* |  |  |  |  |  |  |  |  |  |  |  | 1 |  |  |
| *Lagerstroemia caudata* |  |  |  |  |  |  |  |  |  |  |  | 1 |  |  |
| *Syzygium euonymifolium* |  |  |  | 1 |  |  |  |  |  |  |  |  |  |  |
| *Machilus wenshanensis* |  |  |  |  |  |  |  |  |  | 1 |  |  |  |  |
| *Diospyros eriantha* |  |  |  | 1 |  |  |  |  |  |  |  |  |  |  |
| *Ilex pentagona* |  |  |  |  |  |  |  |  |  |  |  | 1 |  |  |
| *Machilus rehderi* |  |  |  |  |  |  |  |  |  | 1 |  |  |  |  |
| *Toona sinensis* |  |  |  |  |  |  |  |  |  |  |  | 1 |  |  |
| *Lindera communis* |  | 1 |  |  |  |  |  |  |  |  |  |  |  |  |
| *Helicia cochinchinensis* |  |  | 1 |  |  |  |  |  |  |  |  |  |  |  |
| *Morella rubra* |  |  |  |  |  |  |  |  |  |  |  | 1 |  |  |
| *Crateva religiosa* |  |  | 1 |  |  |  |  |  |  |  |  |  |  |  |
| *Juniperus chinensis* |  |  |  |  |  |  |  |  |  |  | 1 |  |  |  |
| *Aesculus assamica* |  |  |  |  |  |  |  |  |  | 1 |  |  |  |  |
| *Handeliodendron bodinieri* |  |  |  |  |  |  |  |  |  | 1 |  |  |  |  |
| *Celtis biondii* |  |  | 1 |  |  |  |  |  |  |  |  |  |  |  |
| *Madhuca pasquieri* |  |  |  |  |  |  |  |  | 1 |  |  |  |  |  |
| *Phoebe sheareri* |  |  |  |  |  |  |  |  |  | 1 |  |  |  |  |

**Table S3.** List of large old trees in Guangxi, South China.

| **No.** | **Prefecture-level City** | **County/District** | **Family** | **Species** | **Age (years)** | **Height (m)** | **DBH (cm)** | **Crown width (m)** | **Habitat** | **Growth status** |
| --- | --- | --- | --- | --- | --- | --- | --- | --- | --- | --- |
| 1 | Nanning | Qingxiu District | Cycadaceae | *Cycas pectinata* Griff. | 1010 | 7.1 | 58.0 | 4.8 | Scenic spots | Good |
| 2 | Nanning | Qingxiu District | Cycadaceae | *Cycas pectinata* Griff. | 1000 | 5.8 | 75.0 | 5.0 | Scenic spots | Good |
| 3 | Nanning | Qingxiu District | Cycadaceae | *Cycas pectinata* Griff. | 1100 | 10.2 | 89.0 | 5.4 | Scenic spots | Good |
| 4 | Nanning | Qingxiu District | Cycadaceae | *Cycas pectinata* Griff. | 1250 | 8.2 | 91.0 | 4.6 | Scenic spots | Good |
| 5 | Nanning | Qingxiu District | Cycadaceae | *Cycas pectinata* Griff. | 1230 | 6.5 | 93.0 | 5.3 | Scenic spots | Good |
| 6 | Nanning | Qingxiu District | Cycadaceae | *Cycas pectinata* Griff. | 1360 | 7.8 | 95.0 | 5.2 | Scenic spots | Good |
| 7 | Nanning | Qingxiu District | Cycadaceae | *Cycas pectinata* Griff. | 1160 | 6.2 | 87.0 | 4.8 | Scenic spots | Good |
| 8 | Nanning | Qingxiu District | Cycadaceae | *Cycas pectinata* Griff. | 1070 | 7.8 | 58.5 | 5.3 | Scenic spots | Good |
| 9 | Nanning | Qingxiu District | Cycadaceae | *Cycas pectinata* Griff. | 1200 | 9.3 | 58.0 | 4.7 | Scenic spots | Good |
| 10 | Nanning | Qingxiu District | Cycadaceae | *Cycas pectinata* Griff. | 1230 | 9.5 | 57.6 | 4.8 | Scenic spots | Good |
| 11 | Nanning | Qingxiu District | Cycadaceae | *Cycas pectinata* Griff. | 1290 | 10.3 | 57.5 | 4.0 | Scenic spots | Good |
| 12 | Nanning | Qingxiu District | Cycadaceae | *Cycas pectinata* Griff. | 1300 | 9.7 | 80.0 | 4.4 | Scenic spots | Good |
| 13 | Nanning | Qingxiu District | Cycadaceae | *Cycas pectinata* Griff. | 1200 | 8.0 | 90.0 | 5.7 | Scenic spots | Good |
| 14 | Nanning | Yongning District | Moraceae | *Ficus microcarp*a L. f. | 1000 | 29.0 | 353.3 | 43.0 | Villages and farmlands | Good |
| 15 | Nanning | Wuming District | Clusiaceae | *Garcinia paucinervis* Chun ex F. C. How | 1000 | 15.1 | 111.4 | 13.4 | Villages and farmlands | Good |
| 16 | Nanning | Wuming District | Moraceae | *Ficus microcarp*a L. f. | 1000 | 22.5 | 310.3 | 43.1 | Villages and farmlands | Good |
| 17 | Nanning | Wuming District | Moraceae | *Ficus racemosa* L. | 1000 | 12.6 | 344.0 | 8.2 | Villages and farmlands | Moderate |
| 18 | Nanning | Wuming District | Clusiaceae | *Garcinia paucinervis* Chun ex F. C. How | 1100 | 20.0 | 125.1 | 19.5 | Villages and farmlands | Good |
| 19 | Nanning | Long'an County | Malvaceae | *Excentrodendron* *tonkinense* (A. Chev.) H. T. Chang & R. H. Miao | 1730 | 20.3 | 310.0 | 17.0 | Villages and farmlands | Moderate |
| 20 | Nanning | Long'an County | Malvaceae | *Excentrodendron* *tonkinense* (A. Chev.) H. T. Chang & R. H. Miao | 1000 | 20.5 | 166.0 | 21.9 | Villages and farmlands | Good |
| 21 | Nanning | Long'an County | Moraceae | *Ficus altissima* Blume. | 1000 | 28.7 | 445.2 | 41.8 | Villages and farmlands | Good |
| 22 | Nanning | Long'an County | Moraceae | *Ficus altissima* Blume. | 1000 | 15.6 | 300.0 | 47.6 | Villages and farmlands | Good |
| 23 | Nanning | Mashan County | Moraceae | *Ficus virens* Aiton. | 1000 | 25.0 | 410.6 | 46.0 | Villages and farmlands | Moderate |
| 24 | Nanning | Xingning District | Sapindaceae | *Dimocarpus longan* Lour. | 540 | 12.0 | 111.0 | 16.5 | Villages and farmlands | Good |
| 25 | Nanning | Qingxiu District | Oxalidaceae | *Averrhoa carambola* L. | 500 | 11.1 | 92.3 | 8.0 | Residential districts | Good |
| 26 | Nanning | Qingxiu District | Cycadaceae | *Cycas pectinata* Griff. | 620 | 6.0 | 29.0 | 3.0 | Scenic spots | Good |
| 27 | Nanning | Qingxiu District | Cycadaceae | *Cycas pectinata* Griff. | 720 | 5.4 | 44.2 | 3.7 | Scenic spots | Good |
| 28 | Nanning | Qingxiu District | Cycadaceae | *Cycas pectinata* Griff. | 640 | 5.1 | 41.0 | 4.1 | Scenic spots | Good |
| 29 | Nanning | Qingxiu District | Cycadaceae | *Cycas pectinata* Griff. | 630 | 4.5 | 48.0 | 3.8 | Scenic spots | Good |
| 30 | Nanning | Qingxiu District | Cycadaceae | *Cycas pectinata* Griff. | 850 | 6.7 | 41.0 | 3.9 | Scenic spots | Good |
| 31 | Nanning | Qingxiu District | Cycadaceae | *Cycas pectinata* Griff. | 680 | 5.8 | 36.0 | 3.7 | Scenic spots | Good |
| 32 | Nanning | Qingxiu District | Cycadaceae | *Cycas pectinata* Griff. | 550 | 4.5 | 42.0 | 3.2 | Scenic spots | Good |
| 33 | Nanning | Qingxiu District | Cycadaceae | *Cycas pectinata* Griff. | 920 | 4.5 | 42.0 | 5.5 | Scenic spots | Good |
| 34 | Nanning | Qingxiu District | Cycadaceae | *Cycas pectinata* Griff. | 600 | 5.2 | 37.0 | 4.0 | Scenic spots | Good |
| 35 | Nanning | Qingxiu District | Cycadaceae | *Cycas pectinata* Griff. | 550 | 4.5 | 43.0 | 3.1 | Scenic spots | Good |
| 36 | Nanning | Qingxiu District | Cycadaceae | *Cycas pectinata* Griff. | 950 | 4.5 | 76.0 | 5.6 | Scenic spots | Good |
| 37 | Nanning | Qingxiu District | Cycadaceae | *Cycas pectinata* Griff. | 540 | 5.4 | 31.0 | 4.4 | Scenic spots | Good |
| 38 | Nanning | Qingxiu District | Cycadaceae | *Cycas pectinata* Griff. | 740 | 5.5 | 44.0 | 5.5 | Scenic spots | Good |
| 39 | Nanning | Qingxiu District | Cycadaceae | *Cycas pectinata* Griff. | 940 | 6.2 | 56.0 | 5.9 | Scenic spots | Good |
| 40 | Nanning | Qingxiu District | Cycadaceae | *Cycas pectinata* Griff. | 870 | 6.4 | 44.0 | 6.4 | Scenic spots | Good |
| 41 | Nanning | Qingxiu District | Cycadaceae | *Cycas pectinata* Griff. | 750 | 5.1 | 49.0 | 4.9 | Scenic spots | Good |
| 42 | Nanning | Qingxiu District | Cycadaceae | *Cycas pectinata* Griff. | 520 | 3.7 | 50.0 | 4.7 | Scenic spots | Good |
| 43 | Nanning | Qingxiu District | Cycadaceae | *Cycas pectinata* Griff. | 550 | 4.5 | 42.0 | 3.6 | Scenic spots | Good |
| 44 | Nanning | Qingxiu District | Cycadaceae | *Cycas pectinata* Griff. | 590 | 5.4 | 34.0 | 3.8 | Scenic spots | Good |
| 45 | Nanning | Qingxiu District | Cycadaceae | *Cycas pectinata* Griff. | 590 | 4.3 | 47.5 | 5.0 | Scenic spots | Good |
| 46 | Nanning | Qingxiu District | Cycadaceae | *Cycas pectinata* Griff. | 550 | 4.0 | 48.4 | 4.9 | Scenic spots | Good |
| 47 | Nanning | Qingxiu District | Cycadaceae | *Cycas pectinata* Griff. | 610 | 3.3 | 61.1 | 5.7 | Scenic spots | Good |
| 48 | Nanning | Qingxiu District | Cycadaceae | *Cycas pectinata* Griff. | 870 | 5.8 | 51.8 | 3.4 | Scenic spots | Good |
| 49 | Nanning | Qingxiu District | Cycadaceae | *Cycas pectinata* Griff. | 870 | 3.5 | 78.5 | 5.8 | Scenic spots | Good |
| 50 | Nanning | Qingxiu District | Cycadaceae | *Cycas pectinata* Griff. | 650 | 2.4 | 74.0 | 4.1 | Scenic spots | Good |
| 51 | Nanning | Qingxiu District | Cycadaceae | *Cycas pectinata* Griff. | 750 | 4.8 | 53.3 | 4.9 | Scenic spots | Good |
| 52 | Nanning | Qingxiu District | Cycadaceae | *Cycas pectinata* Griff. | 680 | 5.4 | 41.0 | 4.2 | Scenic spots | Good |
| 53 | Nanning | Qingxiu District | Cycadaceae | *Cycas pectinata* Griff. | 870 | 5.3 | 58.0 | 6.5 | Scenic spots | Good |
| 54 | Nanning | Qingxiu District | Cycadaceae | *Cycas pectinata* Griff. | 640 | 4.0 | 55.0 | 5.3 | Scenic spots | Good |
| 55 | Nanning | Qingxiu District | Cycadaceae | *Cycas pectinata* Griff. | 630 | 4.0 | 54.3 | 5.8 | Scenic spots | Good |
| 56 | Nanning | Qingxiu District | Cycadaceae | *Cycas pectinata* Griff. | 700 | 4.8 | 49.0 | 5.5 | Scenic spots | Good |
| 57 | Nanning | Qingxiu District | Cycadaceae | *Cycas pectinata* Griff. | 900 | 4.3 | 73.0 | 4.8 | Scenic spots | Good |
| 58 | Nanning | Qingxiu District | Cycadaceae | *Cycas pectinata* Griff. | 620 | 4.1 | 52.0 | 5.0 | Scenic spots | Good |
| 59 | Nanning | Qingxiu District | Cycadaceae | *Cycas pectinata* Griff. | 910 | 4.3 | 75.0 | 4.4 | Scenic spots | Good |
| 60 | Nanning | Qingxiu District | Cycadaceae | *Cycas pectinata* Griff. | 750 | 4.7 | 54.0 | 5.0 | Scenic spots | Good |
| 61 | Nanning | Qingxiu District | Cycadaceae | *Cycas pectinata* Griff. | 590 | 5.2 | 36.2 | 4.1 | Scenic spots | Good |
| 62 | Nanning | Qingxiu District | Cycadaceae | *Cycas pectinata* Griff. | 710 | 3.0 | 72.0 | 4.5 | Scenic spots | Good |
| 63 | Nanning | Qingxiu District | Cycadaceae | *Cycas pectinata* Griff. | 960 | 4.8 | 75.0 | 4.5 | Scenic spots | Good |
| 64 | Nanning | Qingxiu District | Cycadaceae | *Cycas pectinata* Griff. | 790 | 3.3 | 77.0 | 5.3 | Scenic spots | Good |
| 65 | Nanning | Qingxiu District | Cycadaceae | *Cycas pectinata* Griff. | 590 | 4.7 | 42.2 | 3.5 | Scenic spots | Good |
| 66 | Nanning | Qingxiu District | Cycadaceae | *Cycas pectinata* Griff. | 800 | 4.3 | 62.0 | 4.9 | Scenic spots | Good |
| 67 | Nanning | Qingxiu District | Cycadaceae | *Cycas pectinata* Griff. | 770 | 4.0 | 67.4 | 4.1 | Scenic spots | Good |
| 68 | Nanning | Qingxiu District | Cycadaceae | *Cycas pectinata* Griff. | 500 | 4.5 | 40.7 | 4.5 | Scenic spots | Good |
| 69 | Nanning | Qingxiu District | Cycadaceae | *Cycas pectinata* Griff. | 750 | 4.6 | 54.2 | 3.7 | Scenic spots | Good |
| 70 | Nanning | Qingxiu District | Cycadaceae | *Cycas pectinata* Griff. | 880 | 4.2 | 72.0 | 4.0 | Scenic spots | Good |
| 71 | Nanning | Qingxiu District | Cycadaceae | *Cycas pectinata* Griff. | 960 | 4.5 | 79.5 | 5.5 | Scenic spots | Good |
| 72 | Nanning | Qingxiu District | Cycadaceae | *Cycas pectinata* Griff. | 610 | 4.3 | 49.3 | 5.5 | Scenic spots | Good |
| 73 | Nanning | Qingxiu District | Cycadaceae | *Cycas pectinata* Griff. | 680 | 3.5 | 62.5 | 5.1 | Scenic spots | Good |
| 74 | Nanning | Qingxiu District | Cycadaceae | *Cycas pectinata* Griff. | 930 | 5.5 | 63.5 | 4.4 | Scenic spots | Good |
| 75 | Nanning | Qingxiu District | Cycadaceae | *Cycas pectinata* Griff. | 880 | 3.1 | 84.1 | 4.9 | Scenic spots | Good |
| 76 | Nanning | Qingxiu District | Cycadaceae | *Cycas pectinata* Griff. | 680 | 3.5 | 63.6 | 4.7 | Scenic spots | Good |
| 77 | Nanning | Qingxiu District | Cycadaceae | *Cycas pectinata* Griff. | 730 | 3.8 | 63.0 | 4.8 | Scenic spots | Good |
| 78 | Nanning | Qingxiu District | Cycadaceae | *Cycas pectinata* Griff. | 880 | 3.6 | 78.9 | 4.5 | Scenic spots | Good |
| 79 | Nanning | Qingxiu District | Cycadaceae | *Cycas pectinata* Griff. | 590 | 4.5 | 45.2 | 5.2 | Scenic spots | Good |
| 80 | Nanning | Qingxiu District | Cycadaceae | *Cycas pectinata* Griff. | 730 | 4.2 | 57.5 | 4.3 | Scenic spots | Good |
| 81 | Nanning | Qingxiu District | Cycadaceae | *Cycas pectinata* Griff. | 880 | 6.3 | 47.0 | 4.3 | Scenic spots | Good |
| 82 | Nanning | Qingxiu District | Cycadaceae | *Cycas pectinata* Griff. | 570 | 2.8 | 64.0 | 5.6 | Scenic spots | Good |
| 83 | Nanning | Qingxiu District | Cycadaceae | *Cycas pectinata* Griff. | 580 | 3.5 | 56.4 | 4.6 | Scenic spots | Good |
| 84 | Nanning | Qingxiu District | Cycadaceae | *Cycas pectinata* Griff. | 720 | 5.6 | 41.5 | 4.0 | Scenic spots | Good |
| 85 | Nanning | Qingxiu District | Cycadaceae | *Cycas pectinata* Griff. | 950 | 7.0 | 47.5 | 4.0 | Scenic spots | Good |
| 86 | Nanning | Qingxiu District | Cycadaceae | *Cycas pectinata* Griff. | 720 | 4.8 | 50.2 | 4.4 | Scenic spots | Good |
| 87 | Nanning | Qingxiu District | Cycadaceae | *Cycas pectinata* Griff. | 580 | 3.6 | 55.8 | 5.3 | Scenic spots | Good |
| 88 | Nanning | Qingxiu District | Cycadaceae | *Cycas pectinata* Griff. | 920 | 6.1 | 54.0 | 4.5 | Scenic spots | Good |
| 89 | Nanning | Qingxiu District | Cycadaceae | *Cycas pectinata* Griff. | 860 | 4.8 | 63.1 | 4.6 | Scenic spots | Good |
| 90 | Nanning | Qingxiu District | Cycadaceae | *Cycas pectinata* Griff. | 720 | 4.7 | 51.5 | 3.2 | Scenic spots | Good |
| 91 | Nanning | Qingxiu District | Cycadaceae | *Cycas pectinata* Griff. | 720 | 3.5 | 66.1 | 4.4 | Scenic spots | Good |
| 92 | Nanning | Qingxiu District | Cycadaceae | *Cycas pectinata* Griff. | 610 | 5.3 | 37.5 | 3.5 | Scenic spots | Good |
| 93 | Nanning | Qingxiu District | Cycadaceae | *Cycas pectinata* Griff. | 840 | 5.6 | 48.5 | 4.7 | Scenic spots | Good |
| 94 | Nanning | Qingxiu District | Cycadaceae | *Cycas pectinata* Griff. | 670 | 5.0 | 46.0 | 5.6 | Scenic spots | Good |
| 95 | Nanning | Qingxiu District | Cycadaceae | *Cycas pectinata* Griff. | 550 | 3.0 | 61.0 | 5.3 | Scenic spots | Good |
| 96 | Nanning | Qingxiu District | Cycadaceae | *Cycas pectinata* Griff. | 850 | 5.0 | 58.0 | 5.7 | Scenic spots | Good |
| 97 | Nanning | Qingxiu District | Cycadaceae | *Cycas pectinata* Griff. | 850 | 3.3 | 95.0 | 5.3 | Scenic spots | Good |
| 98 | Nanning | Qingxiu District | Cycadaceae | *Cycas pectinata* Griff. | 980 | 7.5 | 46.0 | 3.8 | Scenic spots | Good |
| 99 | Nanning | Qingxiu District | Cycadaceae | *Cycas pectinata* Griff. | 550 | 4.5 | 43.0 | 5.5 | Scenic spots | Good |
| 100 | Nanning | Qingxiu District | Cycadaceae | *Cycas pectinata* Griff. | 780 | 4.8 | 58.5 | 5.5 | Scenic spots | Good |
| 101 | Nanning | Qingxiu District | Cycadaceae | *Cycas pectinata* Griff. | 500 | 5.3 | 30.0 | 3.9 | Scenic spots | Good |
| 102 | Nanning | Qingxiu District | Cycadaceae | *Cycas pectinata* Griff. | 610 | 5.0 | 41.0 | 4.9 | Scenic spots | Good |
| 103 | Nanning | Qingxiu District | Cycadaceae | *Cycas pectinata* Griff. | 500 | 3.0 | 57.0 | 5.3 | Scenic spots | Good |
| 104 | Nanning | Qingxiu District | Cycadaceae | *Cycas pectinata* Griff. | 860 | 3.5 | 77.0 | 5.2 | Scenic spots | Good |
| 105 | Nanning | Qingxiu District | Cycadaceae | *Cycas pectinata* Griff. | 760 | 4.8 | 56.2 | 4.7 | Scenic spots | Good |
| 106 | Nanning | Qingxiu District | Cycadaceae | *Cycas pectinata* Griff. | 560 | 4.0 | 49.2 | 3.6 | Scenic spots | Good |
| 107 | Nanning | Qingxiu District | Cycadaceae | *Cycas pectinata* Griff. | 570 | 4.5 | 44.0 | 4.6 | Scenic spots | Good |
| 108 | Nanning | Qingxiu District | Cycadaceae | *Cycas pectinata* Griff. | 620 | 5.0 | 41.0 | 4.4 | Scenic spots | Good |
| 109 | Nanning | Qingxiu District | Cycadaceae | *Cycas pectinata* Griff. | 650 | 4.6 | 48.5 | 4.9 | Scenic spots | Good |
| 110 | Nanning | Qingxiu District | Cycadaceae | *Cycas pectinata* Griff. | 720 | 6.0 | 36.5 | 4.0 | Scenic spots | Good |
| 111 | Nanning | Qingxiu District | Cycadaceae | *Cycas pectinata* Griff. | 650 | 5.2 | 41.0 | 4.1 | Scenic spots | Good |
| 112 | Nanning | Qingxiu District | Cycadaceae | *Cycas pectinata* Griff. | 510 | 3.8 | 48.0 | 3.9 | Scenic spots | Good |
| 113 | Nanning | Qingxiu District | Cycadaceae | *Cycas pectinata* Griff. | 680 | 5.8 | 35.9 | 3.6 | Scenic spots | Good |
| 114 | Nanning | Qingxiu District | Cycadaceae | *Cycas pectinata* Griff. | 520 | 5.0 | 35.0 | 3.4 | Scenic spots | Good |
| 115 | Nanning | Qingxiu District | Cycadaceae | *Cycas pectinata* Griff. | 700 | 5.5 | 41.7 | 4.4 | Scenic spots | Good |
| 116 | Nanning | Qingxiu District | Cycadaceae | *Cycas pectinata* Griff. | 680 | 5.1 | 44.5 | 2.0 | Scenic spots | Good |
| 117 | Nanning | Qingxiu District | Cycadaceae | *Cycas pectinata* Griff. | 630 | 3.6 | 59.2 | 4.6 | Scenic spots | Good |
| 118 | Nanning | Qingxiu District | Cycadaceae | *Cycas pectinata* Griff. | 950 | 6.1 | 59.1 | 4.5 | Scenic spots | Good |
| 119 | Nanning | Qingxiu District | Cycadaceae | *Cycas pectinata* Griff. | 530 | 3.2 | 56.6 | 4.8 | Scenic spots | Good |
| 120 | Nanning | Qingxiu District | Cycadaceae | *Cycas pectinata* Griff. | 530 | 4.4 | 42.4 | 3.4 | Scenic spots | Good |
| 121 | Nanning | Qingxiu District | Cycadaceae | *Cycas pectinata* Griff. | 810 | 3.9 | 70.5 | 4.9 | Scenic spots | Good |
| 122 | Nanning | Qingxiu District | Cycadaceae | *Cycas pectinata* Griff. | 820 | 3.7 | 72.0 | 5.1 | Scenic spots | Good |
| 123 | Nanning | Qingxiu District | Cycadaceae | *Cycas pectinata* Griff. | 670 | 5.1 | 44.8 | 4.0 | Scenic spots | Good |
| 124 | Nanning | Qingxiu District | Cycadaceae | *Cycas pectinata* Griff. | 500 | 5.2 | 34.0 | 3.8 | Scenic spots | Good |
| 125 | Nanning | Qingxiu District | Cycadaceae | *Cycas pectinata* Griff. | 510 | 4.3 | 42.3 | 4.1 | Scenic spots | Good |
| 126 | Nanning | Qingxiu District | Cycadaceae | *Cycas pectinata* Griff. | 860 | 4.8 | 64.0 | 4.5 | Scenic spots | Good |
| 127 | Nanning | Qingxiu District | Cycadaceae | *Cycas pectinata* Griff. | 590 | 4.0 | 51.8 | 4.5 | Scenic spots | Good |
| 128 | Nanning | Qingxiu District | Cycadaceae | *Cycas pectinata* Griff. | 500 | 3.6 | 50.0 | 4.1 | Scenic spots | Good |
| 129 | Nanning | Qingxiu District | Cycadaceae | *Cycas pectinata* Griff. | 590 | 4.5 | 45.0 | 3.6 | Scenic spots | Good |
| 130 | Nanning | Xixiangtang District | Moraceae | *Ficus altissima* Blume. | 550 | 30.0 | 206.0 | 48.0 | Villages and farmlands | Moderate |
| 131 | Nanning | Xixiangtang District | Moraceae | *Ficus altissima* Blume. | 700 | 32.0 | 210.0 | 28.5 | Villages and farmlands | Moderate |
| 132 | Nanning | Liangqing District | Moraceae | *Ficus altissima* Blume. | 810 | 21.2 | 319.0 | 38.5 | Villages and farmlands | Moderate |
| 133 | Nanning | Yongning District | Moraceae | *Ficus altissima* Blume. | 900 | 17.4 | 325.0 | 56.0 | Villages and farmlands | Good |
| 134 | Nanning | Yongning District | Moraceae | *Ficus microcarpa* L. f. | 500 | 33.0 | 280.0 | 26.5 | Villages and farmlands | Good |
| 135 | Nanning | Yongning District | Moraceae | *Ficus altissima* Blume. | 530 | 26.0 | 277.9 | 43.0 | Villages and farmlands | Good |
| 136 | Nanning | Yongning District | Moraceae | *Ficus virens* Aiton. | 650 | 20.0 | 324.7 | 40.0 | Residential districts | Good |
| 137 | Nanning | Yongning District | Moraceae | *Ficus microcarpa* L. f. | 580 | 17.0 | 270.6 | 38.0 | Villages and farmlands | Good |
| 138 | Nanning | Wuming District | Moraceae | *Ficus altissima* Blume. | 700 | 19.4 | 308.7 | 35.3 | Villages and farmlands | Good |
| 139 | Nanning | Wuming District | Moraceae | *Ficus microcarpa* L. f. | 750 | 18.5 | 305.6 | 25.5 | Villages and farmlands | Good |
| 140 | Nanning | Wuming District | Moraceae | *Ficus microcarpa* L. f. | 710 | 26.8 | 290.3 | 28.7 | Villages and farmlands | Good |
| 141 | Nanning | Wuming District | Moraceae | *Ficus virens* Aiton. | 530 | 14.2 | 285.9 | 22.6 | Villages and farmlands | Good |
| 142 | Nanning | Wuming District | Moraceae | *Ficus virens* Aiton. | 670 | 20.8 | 327.9 | 27.5 | Villages and farmlands | Good |
| 143 | Nanning | Wuming District | Moraceae | *Ficus altissima* Blume. | 510 | 17.5 | 264.2 | 31.2 | Villages and farmlands | Good |
| 144 | Nanning | Wuming District | Moraceae | *Ficus altissima* Blume. | 530 | 18.6 | 270.6 | 25.4 | Villages and farmlands | Good |
| 145 | Nanning | Wuming District | Moraceae | *Ficus altissima* Blume. | 550 | 23.5 | 272.3 | 42.8 | Villages and farmlands | Good |
| 146 | Nanning | Wuming District | Moraceae | *Ficus altissima* Blume. | 730 | 27.6 | 306.1 | 38.1 | Villages and farmlands | Moderate |
| 147 | Nanning | Wuming District | Moraceae | *Ficus altissima* Blume. | 600 | 20.1 | 280.1 | 56.1 | Villages and farmlands | Good |
| 148 | Nanning | Wuming District | Moraceae | *Ficus virens* Aiton. | 570 | 18.2 | 296.2 | 35.7 | Villages and farmlands | Good |
| 149 | Nanning | Wuming District | Moraceae | *Ficus altissima* Blume. | 630 | 18.6 | 289.6 | 33.5 | Villages and farmlands | Good |
| 150 | Nanning | Wuming District | Fabaceae | *Erythrophleum fordii* Oliv. | 650 | 26.9 | 117.8 | 26.8 | Villages and farmlands | Good |
| 151 | Nanning | Wuming District | Aquifoliaceae | *Ilex rotunda* Thunb. | 800 | 12.8 | 105.0 | 16.9 | Villages and farmlands | Good |
| 152 | Nanning | Wuming District | Sapindaceae | *Dimocarpus longan* Lour. | 650 | 13.2 | 120.7 | 15.4 | Villages and farmlands | Good |
| 153 | Nanning | Wuming District | Sapindaceae | *Dimocarpus longan* Lour. | 500 | 14.2 | 111.4 | 16.3 | Villages and farmlands | Poor |
| 154 | Nanning | Wuming District | Moraceae | *Ficus virens* Aiton. | 600 | 22.5 | 307.5 | 31.3 | Villages and farmlands | Good |
| 155 | Nanning | Long'an County | Moraceae | *Ficus altissima* Blume. | 700 | 13.0 | 337.0 | 33.0 | Villages and farmlands | Moderate |
| 156 | Nanning | Long'an County | Moraceae | *Ficus altissima* Blume. | 600 | 18.0 | 310.0 | 21.5 | Villages and farmlands | Moderate |
| 157 | Nanning | Long'an County | Moraceae | *Ficus altissima* Blume. | 550 | 18.0 | 288.0 | 30.0 | Villages and farmlands | Good |
| 158 | Nanning | Long'an County | Moraceae | *Ficus altissima* Blume. | 600 | 27.0 | 380.0 | 14.0 | Villages and farmlands | Good |
| 159 | Nanning | Long'an County | Moraceae | *Ficus altissima* Blume. | 700 | 17.0 | 330.0 | 39.0 | Villages and farmlands | Good |
| 160 | Nanning | Long'an County | Moraceae | *Ficus altissima* Blume. | 550 | 18.0 | 290.0 | 29.5 | Villages and farmlands | Good |
| 161 | Nanning | Long'an County | Oxalidaceae | *Averrhoa carambola* L. | 600 | 16.4 | 116.8 | 13.6 | Residential districts | Good |
| 162 | Nanning | Long'an County | Malvaceae | *Excentrodendron tonkinense* (A. Chev.) H. T. Chang & R. H. Miao | 615 | 30.0 | 136.9 | 20.5 | Villages and farmlands | Moderate |
| 163 | Nanning | Long'an County | Malvaceae | *Excentrodendron tonkinense* (A. Chev.) H. T. Chang & R. H. Miao | 550 | 32.1 | 113.0 | 12.4 | Villages and farmlands | Good |
| 164 | Nanning | Long'an County | Malvaceae | *Excentrodendron tonkinense* (A. Chev.) H. T. Chang & R. H. Miao | 550 | 32.1 | 121.0 | 11.3 | Villages and farmlands | Good |
| 165 | Nanning | Long'an County | Malvaceae | *Excentrodendron tonkinense* (A. Chev.) H. T. Chang & R. H. Miao | 500 | 20.2 | 117.2 | 15.2 | Villages and farmlands | Good |
| 166 | Nanning | Long'an County | Moraceae | *Ficus altissima* Blume. | 650 | 17.8 | 294.6 | 35.1 | Villages and farmlands | Good |
| 167 | Nanning | Long'an County | Moraceae | *Ficus altissima* Blume. | 680 | 20.7 | 330.2 | 30.0 | Villages and farmlands | Good |
| 168 | Nanning | Long'an County | Lauraceae | *Camphora officinarum Nees.* | 550 | 11.3 | 163.0 | 10.2 | Villages and farmlands | Good |
| 169 | Nanning | Long'an County | Moraceae | *Ficus altissima* Blume. | 600 | 19.0 | 324.0 | 27.5 | Villages and farmlands | Good |
| 170 | Nanning | Long'an County | Malvaceae | *Excentrodendron tonkinense* (A. Chev.) H. T. Chang & R. H. Miao | 700 | 18.7 | 149.7 | 22.1 | Villages and farmlands | Good |
| 171 | Nanning | Long'an County | Oxalidaceae | *Averrhoa carambola* L. | 510 | 15.0 | 95.5 | 11.9 | Villages and farmlands | Good |
| 172 | Nanning | Long'an County | Moraceae | *Ficus microcarpa* L. f. | 650 | 22.0 | 286.0 | 33.5 | Residential districts | Good |
| 173 | Nanning | Long'an County | Clusiaceae | *Garcinia paucinervis* Chun ex F. C. How | 610 | 16.0 | 98.0 | 14.5 | Villages and farmlands | Good |
| 174 | Nanning | Long'an County | Clusiaceae | *Garcinia paucinervis* Chun ex F. C. How | 500 | 14.0 | 85.0 | 14.0 | Villages and farmlands | Good |
| 175 | Nanning | Long'an County | Malvaceae | *Excentrodendron tonkinense* (A. Chev.) H. T. Chang & R. H. Miao | 500 | 32.0 | 111.0 | 25.0 | Villages and farmlands | Good |
| 176 | Nanning | Long'an County | Clusiaceae | *Garcinia paucinervis* Chun ex F. C. How | 750 | 21.0 | 108.0 | 12.5 | Villages and farmlands | Good |
| 177 | Nanning | Long'an County | Malvaceae | *Excentrodendron tonkinense* (A. Chev.) H. T. Chang & R. H. Miao | 500 | 13.0 | 117.6 | 10.5 | Villages and farmlands | Good |
| 178 | Nanning | Long'an County | Malvaceae | *Excentrodendron tonkinense* (A. Chev.) H. T. Chang & R. H. Miao | 520 | 16.0 | 120.0 | 15.5 | Villages and farmlands | Good |
| 179 | Nanning | Mashan County | Moraceae | *Ficus altissima* Blume. | 550 | 19.0 | 279.8 | 39.0 | Villages and farmlands | Good |
| 180 | Nanning | Shanglin County | Malvaceae | *Bombax ceiba* L. | 800 | 26.0 | 287.6 | 32.5 | Villages and farmlands | Good |
| 181 | Nanning | Shanglin County | Oxalidaceae | *Averrhoa carambola* L. | 500 | 12.5 | 105.0 | 9.5 | Villages and farmlands | Poor |
| 182 | Nanning | Shanglin County | Sapindaceae | *Dimocarpus longan* Lour. | 500 | 13.4 | 115.0 | 15.0 | Residential districts | Poor |
| 183 | Nanning | Shanglin County | Oxalidaceae | *Averrhoa carambola* L. | 500 | 16.5 | 110.0 | 17.5 | Villages and farmlands | Good |
| 184 | Nanning | Shanglin County | Sapindaceae | *Dimocarpus longan* Lour. | 540 | 16.0 | 125.0 | 12.5 | Villages and farmlands | Poor |
| 185 | Nanning | Shanglin County | Sapindaceae | *Dimocarpus longan* Lour. | 520 | 17.0 | 120.0 | 14.0 | Villages and farmlands | Poor |
| 186 | Nanning | Shanglin County | Sapindaceae | *Dimocarpus longan* Lour. | 540 | 17.0 | 125.0 | 16.5 | Villages and farmlands | Poor |
| 187 | Nanning | Shanglin County | Sapindaceae | *Dimocarpus longan* Lour. | 500 | 14.0 | 110.0 | 6.5 | Villages and farmlands | Poor |
| 188 | Nanning | Shanglin County | Sapindaceae | *Dimocarpus longan* Lour. | 550 | 13.5 | 128.3 | 8.5 | Villages and farmlands | Poor |
| 189 | Nanning | Shanglin County | Malvaceae | *Bombax ceiba* L. | 500 | 20.0 | 260.0 | 24.0 | Villages and farmlands | Poor |
| 190 | Nanning | Shanglin County | Fagaceae | *Castanopsis eyrei* (Champ. ex Benth.) Tutcher | 550 | 15.6 | 77.4 | 6.4 | Wooded areas and plant nurseries | Good |
| 191 | Nanning | Shanglin County | Fagaceae | *Castanopsis eyrei* (Champ. ex Benth.) Tutcher | 500 | 13.2 | 70.4 | 6.3 | Wooded areas and plant nurseries | Good |
| 192 | Nanning | Shanglin County | Fagaceae | *Castanopsis eyrei* (Champ. ex Benth.) Tutcher | 500 | 12.5 | 87.3 | 6.2 | Wooded areas and plant nurseries | Good |
| 193 | Nanning | Shanglin County | Fagaceae | *Castanopsis eyrei* (Champ. ex Benth.) Tutcher | 550 | 13.1 | 89.2 | 6.4 | Wooded areas and plant nurseries | Good |
| 194 | Nanning | Shanglin County | Pinaceae | *Nothotsuga longibracteata* (W. C. Cheng) Hu ex C. N. Page | 660 | 25.6 | 60.8 | 7.6 | Wooded areas and plant nurseries | Good |
| 195 | Nanning | Shanglin County | Pinaceae | *Nothotsuga longibracteata* (W. C. Cheng) Hu ex C. N. Page | 580 | 16.7 | 57.1 | 8.2 | Wooded areas and plant nurseries | Good |
| 196 | Nanning | Shanglin County | Fagaceae | *Castanopsis eyrei* (Champ. ex Benth.) Tutcher | 500 | 9.6 | 76.8 | 5.7 | Wooded areas and plant nurseries | Good |
| 197 | Nanning | Shanglin County | Pinaceae | *Nothotsuga longibracteata* (W. C. Cheng) Hu ex C. N. Page | 660 | 23.8 | 59.2 | 8.5 | Wooded areas and plant nurseries | Good |
| 198 | Nanning | Shanglin County | Fagaceae | *Castanopsis eyrei* (Champ. ex Benth.) Tutcher | 550 | 15.6 | 108.3 | 8.5 | Wooded areas and plant nurseries | Good |
| 199 | Nanning | Shanglin County | Fagaceae | *Castanopsis fargesii* Franch. | 650 | 16.8 | 57.3 | 9.8 | Wooded areas and plant nurseries | Good |
| 200 | Nanning | Shanglin County | Fagaceae | *Castanopsis eyrei* (Champ. ex Benth.) Tutcher | 500 | 13.9 | 71.3 | 4.0 | Wooded areas and plant nurseries | Good |
| 201 | Nanning | Shanglin County | Fagaceae | *Castanopsis eyrei* (Champ. ex Benth.) Tutcher | 550 | 7.8 | 92.4 | 5.1 | Wooded areas and plant nurseries | Good |
| 202 | Nanning | Shanglin County | Fagaceae | *Castanopsis eyrei* (Champ. ex Benth.) Tutcher | 500 | 7.6 | 73.9 | 4.0 | Wooded areas and plant nurseries | Good |
| 203 | Nanning | Shanglin County | Fagaceae | *Castanopsis eyrei* (Champ. ex Benth.) Tutcher | 550 | 7.7 | 93.3 | 5.0 | Wooded areas and plant nurseries | Good |
| 204 | Nanning | Shanglin County | Fagaceae | *Castanopsis eyrei* (Champ. ex Benth.) Tutcher | 550 | 8.5 | 92.5 | 5.2 | Wooded areas and plant nurseries | Good |
| 205 | Nanning | Shanglin County | Fagaceae | *Castanopsis eyrei* (Champ. ex Benth.) Tutcher | 500 | 11.2 | 73.2 | 4.8 | Wooded areas and plant nurseries | Good |
| 206 | Nanning | Shanglin County | Fagaceae | *Castanopsis eyrei* (Champ. ex Benth.) Tutcher | 600 | 8.2 | 96.3 | 5.8 | Wooded areas and plant nurseries | Good |
| 207 | Nanning | Shanglin County | Fagaceae | *Castanopsis eyrei* (Champ. ex Benth.) Tutcher | 550 | 8.3 | 95.5 | 5.3 | Wooded areas and plant nurseries | Good |
| 208 | Nanning | Shanglin County | Fagaceae | *Castanopsis eyrei* (Champ. ex Benth.) Tutcher | 600 | 7.7 | 98.4 | 5.3 | Wooded areas and plant nurseries | Good |
| 209 | Nanning | Binyang County | Cannabaceae | *Aphananthe cuspidata* (Blume) Planch. | 500 | 24.0 | 153.0 | 16.5 | Villages and farmlands | Moderate |
| 210 | Nanning | Binyang County | Moraceae | *Ficus altissima* Blume. | 600 | 22.0 | 274.0 | 35.0 | Villages and farmlands | Good |
| 211 | Nanning | Binyang County | Cupressaceae | *Glyptostrobus pensilis* (Staunton ex D. Don) K. Koch | 700 | 11.0 | 117.8 | 8.5 | Villages and farmlands | Good |
| 212 | Nanning | Hengzhou City | Lauraceae | *Camphora officinarum Nees* | 500 | 15.0 | 200.0 | 19.5 | Villages and farmlands | Good |
| 213 | Nanning | Wuming District | Sapindaceae | *Dimocarpus longan* Lour. | 500 | 10.0 | 117.0 | 8.0 | Villages and farmlands | Good |
| 214 | Nanning | Wuming District | Moraceae | *Ficus microcarpa* L. f. | 500 | 13.5 | 257.8 | 30.5 | Residential districts | Good |
| 215 | Nanning | Wuming District | Moraceae | *Ficus altissima* Blume. | 550 | 16.5 | 270.0 | 31.0 | Residential districts | Poor |
| 216 | Nanning | Wuming District | Moraceae | *Ficus altissima* Blume. | 500 | 20.0 | 296.5 | 24.5 | Villages and farmlands | Poor |
| 217 | Nanning | Wuming District | Moraceae | *Ficus altissima* Blume. | 500 | 20.0 | 270.7 | 22.0 | Villages and farmlands | Good |
| 218 | Liuzhou | Yufeng District | Moraceae | *Ficus microcarpa* L. f. | 1000 | 23.0 | 254.8 | 21.5 | Residential districts | Good |
| 219 | Liuzhou | Liucheng County | Moraceae | *Ficus virens* Aiton | 1000 | 25.0 | 412.4 | 29.0 | Villages and farmlands | Moderate |
| 220 | Liuzhou | Luzhai County | Moraceae | *Ficus virens* Aiton. | 1000 | 25.0 | 460.0 | 29.5 | Residential districts | Moderate |
| 221 | Liuzhou | Rong'an County | Moraceae | *Ficus microcarpa* L. f. | 1000 | 18.0 | 302.0 | 30.0 | Residential districts | Good |
| 222 | Liuzhou | Rongshui County | Anacardiaceae | *Choerospondias axillaris* (Roxb.) B. L. Burtt & A. W. Hill | 1000 | 29.5 | 327.0 | 24.5 | Villages and farmlands | Good |
| 223 | Liuzhou | Rongshui County | Moraceae | *Ficus concinna* (Miq.) Miq. | 1000 | 25.0 | 350.8 | 45.0 | Villages and farmlands | Good |
| 224 | Liuzhou | Rongshui County | Moraceae | *Ficus concinna* (Miq.) Miq. | 1000 | 25.0 | 334.2 | 23.5 | Villages and farmlands | Good |
| 225 | Liuzhou | Rongshui County | Moraceae | *Ficus concinna* (Miq.) Miq. | 1000 | 23.0 | 321.5 | 39.0 | Villages and farmlands | Good |
| 226 | Liuzhou | Rongshui County | Moraceae | *Ficus concinna* (Miq.) Miq. | 1000 | 28.0 | 442.5 | 39.0 | Villages and farmlands | Good |
| 227 | Liuzhou | Rongshui County | Moraceae | *Ficus concinna* (Miq.) Miq. | 1000 | 23.0 | 156.0 | 31.5 | Villages and farmlands | Good |
| 228 | Liuzhou | Rongshui County | Pinaceae | *Pseudotsuga sinensis* Dode. | 1000 | 27.0 | 78.9 | 23.0 | Villages and farmlands | Good |
| 229 | Liuzhou | Rongshui County | Pinaceae | *Pseudotsuga sinensis* Dode. | 1100 | 28.0 | 102.5 | 21.5 | Villages and farmlands | Good |
| 230 | Liuzhou | Rongshui County | Pinaceae | *Pseudotsuga sinensis* Dode. | 1000 | 38.0 | 101.9 | 22.0 | Villages and farmlands | Good |
| 231 | Liuzhou | Rongshui County | Pinaceae | *Pseudotsuga sinensis* Dode. | 1000 | 23.0 | 114.0 | 17.5 | Villages and farmlands | Good |
| 232 | Liuzhou | Rongshui County | Pinaceae | *Pseudotsuga sinensis* Dode. | 1250 | 33.0 | 118.7 | 20.0 | Villages and farmlands | Good |
| 233 | Liuzhou | Rongshui County | Theaceae | *Schima superba* Gardner & Champ. | 1000 | 24.0 | 152.8 | 26.0 | Villages and farmlands | Good |
| 234 | Liuzhou | Rongshui County | Theaceae | *Schima superba* Gardner & Champ. | 1050 | 34.0 | 210.4 | 19.0 | Villages and farmlands | Good |
| 235 | Liuzhou | Rongshui County | Moraceae | *Ficus concinna* (Miq.) Miq. | 1000 | 24.5 | 305.6 | 45.5 | Villages and farmlands | Good |
| 236 | Liuzhou | Rongshui County | Moraceae | *Ficus concinna* (Miq.) Miq. | 1000 | 22.0 | 339.5 | 34.5 | Villages and farmlands | Good |
| 237 | Liuzhou | Rongshui County | Theaceae | *Schima superba* Gardner & Champ. | 1000 | 33.0 | 166.0 | 20.5 | Villages and farmlands | Good |
| 238 | Liuzhou | Rongshui County | Moraceae | *Ficus concinna* (Miq.) Miq. | 1000 | 26.0 | 312.0 | 30.0 | Villages and farmlands | Good |
| 239 | Liuzhou | Sanjiang County | Moraceae | *Ficus concinna* (Miq.) Miq. | 1000 | 20.0 | 302.4 | 31.0 | Residential districts | Good |
| 240 | Liuzhou | Sanjiang County | Moraceae | *Ficus concinna* (Miq.) Miq. | 1000 | 20.0 | 302.7 | 37.5 | Villages and farmlands | Good |
| 241 | Liuzhou | Sanjiang County | Moraceae | *Ficus concinna* (Miq.) Miq. | 1000 | 12.0 | 308.7 | 14.0 | Villages and farmlands | Good |
| 242 | Liuzhou | Sanjiang County | Moraceae | *Ficus concinna* (Miq.) Miq. | 1000 | 30.0 | 354.0 | 49.0 | Villages and farmlands | Good |
| 243 | Liuzhou | Sanjiang County | Theaceae | *Schima superba* Gardner & Champ. | 1100 | 18.6 | 177.6 | 16.9 | Villages and farmlands | Good |
| 244 | Liuzhou | Sanjiang County | Moraceae | S*chima superba* Gardner & Champ. | 1000 | 25.6 | 359.7 | 45.5 | Villages and farmlands | Good |
| 245 | Liuzhou | Sanjiang County | Moraceae | *Ficus concinna* (Miq.) Miq. | 1000 | 22.5 | 371.0 | 25.3 | Villages and farmlands | Good |
| 246 | Liuzhou | Sanjiang County | Moraceae | *Ficus concinna* (Miq.) Miq. | 1000 | 19.0 | 305.0 | 32.0 | Villages and farmlands | Good |
| 247 | Liuzhou | Sanjiang County | Moraceae | *Ficus concinna* (Miq.) Miq. | 1000 | 18.0 | 302.0 | 31.5 | Villages and farmlands | Good |
| 248 | Liuzhou | Sanjiang County | Moraceae | *Ficus concinna* (Miq.) Miq. | 1000 | 18.0 | 364.5 | 20.0 | Villages and farmlands | Good |
| 249 | Liuzhou | Sanjiang County | Theaceae | *Ficus concinna* (Miq.) Miq. | 1000 | 16.0 | 156.8 | 7.0 | Villages and farmlands | Good |
| 250 | Liuzhou | Sanjiang County | Moraceae | *Schima superba* Gardner & Champ. | 1000 | 19.0 | 311.0 | 34.0 | Villages and farmlands | Good |
| 251 | Liuzhou | Sanjiang County | Moraceae | *Ficus concinna* (Miq.) Miq. | 1450 | 33.0 | 413.8 | 59.0 | Villages and farmlands | Good |
| 252 | Liuzhou | Sanjiang County | Moraceae | *Ficus concinna* (Miq.) Miq. | 1000 | 32.0 | 332.1 | 50.5 | Villages and farmlands | Good |
| 253 | Liuzhou | Sanjiang County | Moraceae | *Ficus concinna* (Miq.) Miq. | 1000 | 18.0 | 304.0 | 38.0 | Villages and farmlands | Good |
| 254 | Liuzhou | Sanjiang County | Moraceae | *Ficus concinna* (Miq.) Miq. | 1000 | 20.0 | 304.0 | 32.5 | Villages and farmlands | Good |
| 255 | Liuzhou | Sanjiang County | Moraceae | *Ficus concinna* (Miq.) Miq. | 1000 | 25.0 | 305.0 | 34.0 | Villages and farmlands | Good |
| 256 | Liuzhou | Sanjiang County | Moraceae | *Ficus concinna* (Miq.) Miq. | 1000 | 22.0 | 302.0 | 38.0 | Villages and farmlands | Good |
| 257 | Liuzhou | Sanjiang County | Moraceae | *Ficus concinna* (Miq.) Miq. | 1000 | 20.0 | 304.0 | 42.5 | Villages and farmlands | Good |
| 258 | Liuzhou | Chengzhong District | Sapindaceae | *Dimocarpus longan* Lour. | 500 | 8.0 | 73.0 | 19.0 | Villages and farmlands | Good |
| 259 | Liuzhou | Chengzhong District | Sapindaceae | *Dimocarpus longan* Lour. | 500 | 14.0 | 109.0 | 11.0 | Villages and farmlands | Good |
| 260 | Liuzhou | Chengzhong District | Sapindaceae | *Dimocarpus longan* Lour. | 550 | 10.0 | 113.0 | 10.0 | Villages and farmlands | Good |
| 261 | Liuzhou | Yufeng District | Lauraceae | *Camphora officinarum Nees.* | 500 | 13.0 | 200.0 | 17.0 | Residential districts | Poor |
| 262 | Liuzhou | Liudong New District | Lauraceae | *Camphora officinarum Nees.* | 800 | 14.0 | 292.0 | 15.0 | Villages and farmlands | Poor |
| 263 | Liuzhou | Liujiang District | Moraceae | *Ficus virens* Aiton. | 580 | 32.0 | 216.0 | 34.0 | Villages and farmlands | Moderate |
| 264 | Liuzhou | Liujiang District | Moraceae | *Ficus virens* Aiton. | 650 | 19.0 | 96.0 | 24.0 | Villages and farmlands | Good |
| 265 | Liuzhou | Liujiang District | Lauraceae | *Camphora officinarum Nees.* | 800 | 20.0 | 204.0 | 29.0 | Villages and farmlands | Poor |
| 266 | Liuzhou | Liujiang District | Lauraceae | *Camphora officinarum Nees.* | 800 | 17.0 | 89.0 | 27.0 | Villages and farmlands | Poor |
| 267 | Liuzhou | Liujiang District | Moraceae | *Ficus virens* Aiton. | 520 | 21.0 | 192.0 | 25.0 | Villages and farmlands | Moderate |
| 268 | Liuzhou | Liujiang District | Moraceae | *Ficus virens* Aiton. | 560 | 20.0 | 170.0 | 26.0 | Villages and farmlands | Moderate |
| 269 | Liuzhou | Liujiang District | Moraceae | *Ficus virens* Aiton. | 550 | 32.0 | 268.0 | 36.0 | Villages and farmlands | Good |
| 270 | Liuzhou | Liucheng County | Moraceae | *Ficus virens* Aiton. | 800 | 25.0 | 353.5 | 39.5 | Villages and farmlands | Moderate |
| 271 | Liuzhou | Liucheng County | Moraceae | *Ficus microcarpa* L. f. | 500 | 24.0 | 238.9 | 17.5 | Villages and farmlands | Good |
| 272 | Liuzhou | Liucheng County | Moraceae | *Ficus virens* Aiton. | 650 | 25.0 | 331.1 | 27.0 | Villages and farmlands | Good |
| 273 | Liuzhou | Liucheng County | Moraceae | *Ficus virens* Aiton. | 550 | 31.0 | 363.1 | 38.5 | Villages and farmlands | Good |
| 274 | Liuzhou | Luzhai County | Moraceae | *Ficus virens* Aiton. | 600 | 20.0 | 223.0 | 35.0 | Residential districts | Good |
| 275 | Liuzhou | Luzhai County | Moraceae | *Ficus virens* Aiton. | 600 | 23.0 | 255.0 | 31.5 | Residential districts | Good |
| 276 | Liuzhou | Luzhai County | Lauraceae | *Camphora officinarum Nees.* | 600 | 27.0 | 267.8 | 36.0 | Villages and farmlands | Moderate |
| 277 | Liuzhou | Luzhai County | Lauraceae | *Camphora officinarum Nees.* | 600 | 28.0 | 205.4 | 27.0 | Villages and farmlands | Good |
| 278 | Liuzhou | Luzhai County | Lauraceae | *Camphora officinarum Nees.* | 600 | 22.0 | 154.5 | 18.5 | Villages and farmlands | Good |
| 279 | Liuzhou | Luzhai County | Lauraceae | *Camphora officinarum Nees.* | 600 | 24.0 | 169.4 | 18.0 | Villages and farmlands | Good |
| 280 | Liuzhou | Luzhai County | Lauraceae | *Camphora officinarum Nees.* | 600 | 26.0 | 140.1 | 22.0 | Villages and farmlands | Good |
| 281 | Liuzhou | Luzhai County | Lauraceae | *Camphora officinarum Nees.* | 600 | 23.0 | 145.2 | 22.0 | Villages and farmlands | Good |
| 282 | Liuzhou | Luzhai County | Lauraceae | *Camphora officinarum Nees.* | 600 | 28.0 | 239.8 | 24.0 | Villages and farmlands | Good |
| 283 | Liuzhou | Luzhai County | Lauraceae | *Camphora officinarum Nees.* | 600 | 29.0 | 144.6 | 20.0 | Villages and farmlands | Good |
| 284 | Liuzhou | Luzhai County | Lauraceae | *Camphora officinarum Nees.* | 600 | 34.0 | 167.2 | 25.5 | Villages and farmlands | Good |
| 285 | Liuzhou | Luzhai County | Lauraceae | *Camphora officinarum Nees.* | 600 | 32.0 | 228.0 | 25.0 | Villages and farmlands | Good |
| 286 | Liuzhou | Luzhai County | Lauraceae | *Camphora officinarum Nees.* | 600 | 18.0 | 228.8 | 30.0 | Villages and farmlands | Good |
| 287 | Liuzhou | Luzhai County | Moraceae | *Ficus virens* Aiton. | 650 | 22.0 | 327.0 | 24.0 | Residential districts | Good |
| 288 | Liuzhou | Luzhai County | Lauraceae | *Camphora officinarum Nees.* | 520 | 7.0 | 229.2 | 7.5 | Villages and farmlands | Good |
| 289 | Liuzhou | Luzhai County | Moraceae | *Ficus microcarpa* L. f. | 600 | 22.0 | 270.0 | 23.5 | Villages and farmlands | Good |
| 290 | Liuzhou | Rong'an County | Lauraceae | *Camphora officinarum Nees.* | 700 | 32.0 | 286.0 | 45.5 | Villages and farmlands | Good |
| 291 | Liuzhou | Rong'an County | Lauraceae | *Camphora officinarum Nees.* | 500 | 30.0 | 235.0 | 34.0 | Villages and farmlands | Good |
| 292 | Liuzhou | Rong'an County | Hamamelidaceae | *Corylopsis sinensis* Hemsl. | 500 | 16.0 | 83.0 | 10.0 | Villages and farmlands | Poor |
| 293 | Liuzhou | Rong'an County | Lauraceae | *Camphora officinarum Nees.* | 620 | 30.0 | 269.0 | 27.0 | Villages and farmlands | Good |
| 294 | Liuzhou | Rong'an County | Altingiaceae | *Liquidambar formosana* Hance. | 550 | 30.0 | 200.6 | 15.0 | Villages and farmlands | Moderate |
| 295 | Liuzhou | Rong'an County | Altingiaceae | *Liquidambar formosana* Hance. | 550 | 30.0 | 184.7 | 9.0 | Villages and farmlands | Moderate |
| 296 | Liuzhou | Rong'an County | Lauraceae | *Camphora officinarum Nees.* | 500 | 20.0 | 197.0 | 25.0 | Villages and farmlands | Good |
| 297 | Liuzhou | Rong'an County | Phyllanthaceae | *Bischofia polycarpa* (H. Lév.) Airy Shaw | 590 | 24.0 | 240.0 | 18.0 | Villages and farmlands | Good |
| 298 | Liuzhou | Rong'an County | Fagaceae | *Castanea henryi* (Skan) Rehder & E. H. Wilson | 680 | 22.0 | 178.0 | 13.0 | Villages and farmlands | Moderate |
| 299 | Liuzhou | Rongshui County | Moraceae | *Ficus virens* Aiton. | 550 | 21.0 | 352.7 | 25.0 | Villages and farmlands | Moderate |
| 300 | Liuzhou | Rongshui County | Moraceae | *Ficus virens* Aiton. | 500 | 30.0 | 310.0 | 28.0 | Villages and farmlands | Good |
| 301 | Liuzhou | Rongshui County | Moraceae | *Ficus virens* Aiton. | 500 | 31.0 | 283.9 | 49.0 | Villages and farmlands | Good |
| 302 | Liuzhou | Rongshui County | Moraceae | *Ficus virens* Aiton. | 500 | 16.0 | 274.0 | 25.0 | Villages and farmlands | Good |
| 303 | Liuzhou | Rongshui County | Moraceae | *Ficus virens* Aiton. | 600 | 22.0 | 325.0 | 33.0 | Villages and farmlands | Good |
| 304 | Liuzhou | Rongshui County | Lauraceae | *Camphora officinarum Nees.* | 600 | 27.0 | 275.3 | 34.5 | Villages and farmlands | Moderate |
| 305 | Liuzhou | Rongshui County | Lauraceae | *Camphora officinarum Nees.* | 500 | 26.3 | 225.7 | 29.7 | Villages and farmlands | Good |
| 306 | Liuzhou | Rongshui County | Lauraceae | *Camphora officinarum Nees.* | 500 | 16.0 | 175.1 | 27.1 | Villages and farmlands | Good |
| 307 | Liuzhou | Rongshui County | Lauraceae | *Camphora officinarum Nees.* | 500 | 26.8 | 200.5 | 32.5 | Villages and farmlands | Good |
| 308 | Liuzhou | Rongshui County | Lauraceae | *Camphora officinarum Nees.* | 500 | 27.5 | 205.0 | 39.5 | Villages and farmlands | Good |
| 309 | Liuzhou | Rongshui County | Lauraceae | *Camphora officinarum Nees.* | 500 | 27.0 | 218.4 | 28.5 | Villages and farmlands | Good |
| 310 | Liuzhou | Rongshui County | Lauraceae | *Camphora officinarum Nees.* | 510 | 19.0 | 209.5 | 31.2 | Villages and farmlands | Good |
| 311 | Liuzhou | Rongshui County | Moraceae | *Ficus virens* Aiton. | 500 | 25.0 | 273.0 | 48.5 | Villages and farmlands | Good |
| 312 | Liuzhou | Rongshui County | Moraceae | *Ficus virens* Aiton. | 700 | 23.0 | 315.0 | 32.5 | Villages and farmlands | Good |
| 313 | Liuzhou | Rongshui County | Moraceae | *Ficus concinna (*Miq.) Miq. | 500 | 18.0 | 274.4 | 45.5 | Villages and farmlands | Good |
| 314 | Liuzhou | Rongshui County | Moraceae | *Ficus microcarpa* L. f. | 550 | 23.0 | 287.8 | 49.5 | Villages and farmlands | Good |
| 315 | Liuzhou | Rongshui County | Moraceae | *Ficus microcarpa* L. f. | 550 | 25.0 | 283.3 | 41.5 | Villages and farmlands | Good |
| 316 | Liuzhou | Rongshui County | Moraceae | *Ficus microcarpa* L. f. | 550 | 25.0 | 276.3 | 30.0 | Villages and farmlands | Good |
| 317 | Liuzhou | Rongshui County | Moraceae | *Ficus microcarpa* L. f. | 600 | 24.0 | 292.8 | 37.0 | Villages and farmlands | Good |
| 318 | Liuzhou | Rongshui County | Moraceae | *Ficus microcarpa* L. f. | 550 | 15.0 | 282.0 | 43.0 | Villages and farmlands | Good |
| 319 | Liuzhou | Rongshui County | Moraceae | *Ficus microcarpa* L. f. | 600 | 24.0 | 286.0 | 42.0 | Villages and farmlands | Good |
| 320 | Liuzhou | Rongshui County | Moraceae | *Ficus microcarpa* L. f. | 500 | 22.0 | 270.6 | 37.5 | Villages and farmlands | Good |
| 321 | Liuzhou | Rongshui County | Moraceae | *Ficus microcarpa* L. f. | 500 | 22.0 | 270.6 | 37.5 | Villages and farmlands | Good |
| 322 | Liuzhou | Rongshui County | Moraceae | *Ficus microcarpa* L. f. | 800 | 36.0 | 280.3 | 30.0 | Residential districts | Good |
| 323 | Liuzhou | Rongshui County | Moraceae | *Ficus microcarpa* L. f. | 800 | 19.0 | 180.3 | 24.0 | Residential districts | Good |
| 324 | Liuzhou | Rongshui County | Moraceae | *Ficus microcarpa* L. f. | 800 | 27.0 | 299.2 | 30.0 | Villages and farmlands | Good |
| 325 | Liuzhou | Rongshui County | Moraceae | *Ficus microcarpa* L. f. | 800 | 22.0 | 299.4 | 34.0 | Villages and farmlands | Good |
| 326 | Liuzhou | Rongshui County | Fagaceae | *Castanopsis hystrix* Hook. f. & Thomson ex A. DC. | 550 | 24.0 | 146.0 | 11.0 | Villages and farmlands | Moderate |
| 327 | Liuzhou | Rongshui County | Fagaceae | *Castanopsis hystrix* Hook. f. & Thomson ex A. DC. | 600 | 30.0 | 159.0 | 16.0 | Villages and farmlands | Good |
| 328 | Liuzhou | Rongshui County | Moraceae | *Ficus microcarpa* L. f. | 500 | 24.0 | 258.0 | 24.0 | Villages and farmlands | Good |
| 329 | Liuzhou | Rongshui County | Moraceae | *Ficus microcarpa* L. f. | 600 | 25.0 | 271.0 | 38.0 | Villages and farmlands | Good |
| 330 | Liuzhou | Rongshui County | Theaceae | *Schima superba* Gardner & Champ. | 500 | 5.0 | 120.0 | 3.0 | Villages and farmlands | Good |
| 331 | Liuzhou | Rongshui County | Podocarpaceae | *Dacrycarpus imbricatus* (Blume) de Laub. | 550 | 31.0 | 111.0 | 18.0 | Villages and farmlands | Good |
| 332 | Liuzhou | Rongshui County | Podocarpaceae | *Dacrycarpus imbricatus* (Blume) de Laub. | 700 | 17.0 | 124.0 | 14.0 | Villages and farmlands | Good |
| 333 | Liuzhou | Rongshui County | Podocarpaceae | *Dacrycarpus imbricatus* (Blume) de Laub. | 500 | 18.0 | 107.0 | 18.0 | Villages and farmlands | Good |
| 334 | Liuzhou | Rongshui County | Podocarpaceae | *Dacrycarpus imbricatus* (Blume) de Laub. | 550 | 25.0 | 113.0 | 17.0 | Villages and farmlands | Good |
| 335 | Liuzhou | Rongshui County | Podocarpaceae | *Dacrycarpus imbricatus* (Blume) de Laub. | 700 | 28.0 | 127.0 | 18.0 | Villages and farmlands | Good |
| 336 | Liuzhou | Rongshui County | Podocarpaceae | *Dacrycarpus imbricatus* (Blume) de Laub. | 650 | 30.0 | 121.0 | 20.0 | Villages and farmlands | Good |
| 337 | Liuzhou | Rongshui County | Pinaceae | *Pinus massoniana* Lamb. | 550 | 24.5 | 193.0 | 25.5 | Villages and farmlands | Good |
| 338 | Liuzhou | Rongshui County | Pinaceae | *Pinus massoniana* Lamb. | 500 | 20.5 | 128.3 | 21.5 | Villages and farmlands | Good |
| 339 | Liuzhou | Rongshui County | Cupressaceae | *Cunninghamia lanceolata* (Lamb.) Hook. | 500 | 29.0 | 128.3 | 12.7 | Villages and farmlands | Good |
| 340 | Liuzhou | Rongshui County | Cannabaceae | *Aphananthe aspera* (Thunb.) Planch. | 550 | 16.0 | 154.1 | 14.0 | Villages and farmlands | Moderate |
| 341 | Liuzhou | Rongshui County | Pinaceae | *Pinus massoniana* Lamb. | 500 | 42.0 | 147.1 | 18.5 | Villages and farmlands | Good |
| 342 | Liuzhou | Rongshui County | Moraceae | *Ficus microcarpa* L. f. | 500 | 17.0 | 256.3 | 34.0 | Villages and farmlands | Good |
| 343 | Liuzhou | Rongshui County | Taxaceae | *Taxus wallichiana* var. *mairei* (Lemée & H. Lév.) L. K. Fu & Nan Li | 500 | 13.0 | 108.2 | 19.0 | Villages and farmlands | Good |
| 344 | Liuzhou | Rongshui County | Taxaceae | *Taxus wallichiana* var. *mairei* (Lemée & H. Lév.) L. K. Fu & Nan Li | 600 | 22.0 | 86.0 | 15.3 | Villages and farmlands | Good |
| 345 | Liuzhou | Rongshui County | Pinaceae | *Keteleeria davidiana* (C. E. Bertrand) Beissn. | 600 | 27.0 | 177.6 | 15.0 | Villages and farmlands | Good |
| 346 | Liuzhou | Rongshui County | Theaceae | *Schima superba* Gardner & Champ. | 500 | 31.0 | 132.4 | 17.0 | Villages and farmlands | Good |
| 347 | Liuzhou | Rongshui County | Fagaceae | *Castanopsis hystrix* Hook. f. & Thomson ex A. DC. | 600 | 22.5 | 159.2 | 9.0 | Villages and farmlands | Good |
| 348 | Liuzhou | Rongshui County | Theaceae | *Schima superba G*ardner & Champ. | 550 | 24.0 | 124.0 | 11.0 | Villages and farmlands | Good |
| 349 | Liuzhou | Rongshui County | Fagaceae | *Castanopsis hystrix* Hook. f. & Thomson ex A. DC. | 500 | 15.0 | 140.0 | 9.0 | Villages and farmlands | Good |
| 350 | Liuzhou | Rongshui County | Theaceae | *Schima superba* Gardner & Champ. | 500 | 24.0 | 191.0 | 13.0 | Villages and farmlands | Good |
| 351 | Liuzhou | Rongshui County | Cupressaceae | *Cunninghamia lanceolata* (Lamb.) Hook. | 500 | 18.0 | 137.0 | 6.0 | Villages and farmlands | Good |
| 352 | Liuzhou | Rongshui County | Moraceae | *Ficus microcarpa* L. f. | 700 | 27.5 | 289.0 | 36.0 | Villages and farmlands | Good |
| 353 | Liuzhou | Rongshui County | Theaceae | *Schima superba* Gardner & Champ. | 650 | 25.0 | 134.0 | 11.5 | Villages and farmlands | Good |
| 354 | Liuzhou | Rongshui County | Lauraceae | *Phoebe bournei (*Hemsl.) Yen C. Yang | 500 | 25.0 | 167.0 | 14.0 | Villages and farmlands | Good |
| 355 | Liuzhou | Rongshui County | Fagaceae | *Castanopsis tibetana* Hance | 500 | 16.0 | 140.0 | 15.0 | Villages and farmlands | Poor |
| 356 | Liuzhou | Sanjiang County | Moraceae | *Ficus concinna (*Miq.) Miq. | 600 | 21.0 | 261.0 | 31.5 | Residential districts | Good |
| 357 | Liuzhou | Sanjiang County | Fagaceae | *Castanopsis hystrix* Hook. f. & Thomson ex A. DC. | 550 | 26.0 | 152.8 | 21.5 | Villages and farmlands | Good |
| 358 | Liuzhou | Sanjiang County | Fagaceae | *Castanopsis hystrix* Hook. f. & Thomson ex A. DC. | 500 | 27.0 | 143.2 | 19.5 | Villages and farmlands | Good |
| 359 | Liuzhou | Sanjiang County | Fagaceae | *Castanopsis hystrix* Hook. f. & Thomson ex A. DC. | 550 | 15.0 | 146.0 | 18.0 | Villages and farmlands | Good |
| 360 | Liuzhou | Sanjiang County | Fagaceae | *Castanopsis hystrix* Hook. f. & Thomson ex A. DC. | 500 | 28.0 | 135.7 | 12.5 | Villages and farmlands | Good |
| 361 | Liuzhou | Sanjiang County | Moraceae | *Ficus concinna (*Miq.) Miq. | 500 | 27.0 | 254.6 | 35.5 | Villages and farmlands | Moderate |
| 362 | Liuzhou | Sanjiang County | Moraceae | *Ficus concinna (*Miq.) Miq. | 600 | 24.0 | 286.0 | 38.0 | Villages and farmlands | Moderate |
| 363 | Liuzhou | Sanjiang County | Moraceae | *Ficus concinna (*Miq.) Miq. | 500 | 30.0 | 254.7 | 22.0 | Villages and farmlands | Moderate |
| 364 | Liuzhou | Sanjiang County | Fagaceae | *Castanopsis hystrix* Hook. f. & Thomson ex A. DC. | 550 | 30.0 | 146.4 | 28.0 | Villages and farmlands | Good |
| 365 | Liuzhou | Sanjiang County | Moraceae | *Ficus concinna (*Miq.) Miq. | 500 | 20.0 | 297.8 | 37.0 | Villages and farmlands | Moderate |
| 366 | Liuzhou | Sanjiang County | Fagaceae | *Castanopsis hystrix* Hook. f. & Thomson ex A. DC. | 650 | 29.0 | 166.2 | 13.5 | Residential districts | Good |
| 367 | Liuzhou | Sanjiang County | Fagaceae | *Castanopsis hystrix* Hook. f. & Thomson ex A. DC. | 650 | 35.0 | 165.6 | 19.0 | Villages and farmlands | Good |
| 368 | Liuzhou | Sanjiang County | Fagaceae | *Castanopsis hystrix* Hook. f. & Thomson ex A. DC. | 500 | 18.0 | 136.6 | 18.0 | Villages and farmlands | Good |
| 369 | Liuzhou | Sanjiang County | Moraceae | *Ficus concinna (*Miq.) Miq. | 510 | 27.0 | 257.9 | 31.5 | Villages and farmlands | Good |
| 370 | Liuzhou | Sanjiang County | Fagaceae | *Castanopsis hystrix* Hook. f. & Thomson ex A. DC. | 700 | 19.0 | 171.9 | 17.0 | Villages and farmlands | Good |
| 371 | Liuzhou | Sanjiang County | Fagaceae | *Castanopsis hystrix* Hook. f. & Thomson ex A. DC. | 520 | 22.0 | 143.3 | 25.0 | Villages and farmlands | Good |
| 372 | Liuzhou | Sanjiang County | Fagaceae | *Castanopsis hystrix* Hook. f. & Thomson ex A. DC. | 700 | 21.0 | 175.1 | 13.5 | Villages and farmlands | Good |
| 373 | Liuzhou | Sanjiang County | Fagaceae | *Castanopsis hystrix* Hook. f. & Thomson ex A. DC. | 500 | 29.0 | 136.3 | 25.0 | Villages and farmlands | Good |
| 374 | Liuzhou | Sanjiang County | Fagaceae | *Castanopsis hystrix* Hook. f. & Thomson ex A. DC. | 500 | 27.0 | 136.9 | 24.5 | Villages and farmlands | Good |
| 375 | Liuzhou | Sanjiang County | Podocarpaceae | *Podocarpus macrophyllus* (Thunb.) Sweet | 550 | 15.0 | 112.4 | 6.5 | Villages and farmlands | Good |
| 376 | Liuzhou | Sanjiang County | Fagaceae | *Castanopsis hystrix* Hook. f. & Thomson ex A. DC. | 800 | 30.0 | 191.0 | 25.5 | Villages and farmlands | Good |
| 377 | Liuzhou | Sanjiang County | Theaceae | *Schima superba* Gardner & Champ. | 550 | 28.0 | 127.3 | 4.0 | Villages and farmlands | Good |
| 378 | Liuzhou | Sanjiang County | Fagaceae | *Castanopsis hystrix* Hook. f. & Thomson ex A. DC. | 500 | 25.0 | 133.7 | 25.0 | Villages and farmlands | Good |
| 379 | Liuzhou | Sanjiang County | Lauraceae | *Lindera communis* Hemsl. | 500 | 9.0 | 100.0 | 8.5 | Villages and farmlands | Good |
| 380 | Liuzhou | Sanjiang County | Fagaceae | *Castanopsis hystrix* Hook. f. & Thomson ex A. DC. | 500 | 26.0 | 143.3 | 21.0 | Villages and farmlands | Good |
| 381 | Liuzhou | Sanjiang County | Fagaceae | *Castanopsis hystrix* Hook. f. & Thomson ex A. DC. | 650 | 34.0 | 165.6 | 24.0 | Villages and farmlands | Good |
| 382 | Liuzhou | Sanjiang County | Fagaceae | *Castanopsis hystrix* Hook. f. & Thomson ex A. DC. | 600 | 26.0 | 159.2 | 19.0 | Villages and farmlands | Good |
| 383 | Liuzhou | Sanjiang County | Theaceae | *Schima superba G*ardner & Champ. | 500 | 35.0 | 118.0 | 15.0 | Villages and farmlands | Good |
| 384 | Liuzhou | Sanjiang County | Fagaceae | *Castanopsis hystrix* Hook. f. & Thomson ex A. DC. | 600 | 20.0 | 156.0 | 10.0 | Villages and farmlands | Good |
| 385 | Liuzhou | Sanjiang County | Fagaceae | *Castanopsis faberi* Hance. | 560 | 25.0 | 135.3 | 19.0 | Villages and farmlands | Good |
| 386 | Liuzhou | Sanjiang County | Moraceae | *Ficus concinna (*Miq.) Miq. | 500 | 21.0 | 143.8 | 24.0 | Residential districts | Moderate |
| 387 | Liuzhou | Sanjiang County | Moraceae | *Ficus concinna (*Miq.) Miq. | 500 | 17.0 | 140.1 | 23.0 | Residential districts | Moderate |
| 388 | Liuzhou | Sanjiang County | Moraceae | *Ficus concinna (*Miq.) Miq. | 510 | 22.0 | 213.6 | 22.0 | Residential districts | Moderate |
| 389 | Liuzhou | Sanjiang County | Moraceae | *Ficus concinna (*Miq.) Miq. | 500 | 17.0 | 113.0 | 22.0 | Residential districts | Moderate |
| 390 | Liuzhou | Sanjiang County | Lauraceae | *Camphora officinarum Nees.* | 500 | 28.2 | 226.1 | 26.8 | Villages and farmlands | Good |
| 391 | Liuzhou | Sanjiang County | Fabaceae | *Dalbergia hupeana* Hance. | 500 | 17.5 | 120.0 | 20.5 | Villages and farmlands | Good |
| 392 | Liuzhou | Sanjiang County | Fagaceae | *Castanopsis hystrix* Hook. f. & Thomson ex A. DC. | 500 | 28.0 | 136.9 | 20.9 | Villages and farmlands | Good |
| 393 | Liuzhou | Sanjiang County | Moraceae | *Ficus concinna (*Miq.) Miq. | 500 | 28.0 | 257.9 | 32.5 | Villages and farmlands | Moderate |
| 394 | Liuzhou | Sanjiang County | Theaceae | *Schima superba* Gardner & Champ. | 500 | 40.0 | 117.8 | 18.0 | Villages and farmlands | Good |
| 395 | Liuzhou | Sanjiang County | Rhamnaceae | *Hovenia acerba* Lindl. | 500 | 19.0 | 94.0 | 8.8 | Villages and farmlands | Poor |
| 396 | Liuzhou | Sanjiang County | Moraceae | *Ficus concinna (*Miq.) Miq. | 800 | 25.0 | 300.0 | 36.0 | Villages and farmlands | Moderate |
| 397 | Liuzhou | Sanjiang County | Moraceae | *Ficus concinna (*Miq.) Miq. | 700 | 38.0 | 286.0 | 38.5 | Villages and farmlands | Moderate |
| 398 | Liuzhou | Sanjiang County | Fagaceae | *Quercus acutissima* Carruth. | 550 | 34.0 | 119.0 | 8.5 | Villages and farmlands | Good |
| 399 | Liuzhou | Sanjiang County | Taxaceae | *Taxus wallichiana* var. *mairei* (Lemée & H. Lév.) L. K. Fu & Nan Li | 600 | 24.3 | 120.0 | 15.5 | Villages and farmlands | Good |
| 400 | Liuzhou | Sanjiang County | Fagaceae | *Castanopsis hystrix* Hook. f. & Thomson ex A. DC. | 500 | 30.0 | 133.7 | 14.5 | Villages and farmlands | Good |
| 401 | Liuzhou | Sanjiang County | Moraceae | *Ficus concinna (*Miq.) Miq. | 530 | 18.0 | 220.0 | 21.5 | Villages and farmlands | Moderate |
| 402 | Liuzhou | Sanjiang County | Moraceae | *Ficus concinna (*Miq.) Miq. | 510 | 14.5 | 250.0 | 30.0 | Villages and farmlands | Moderate |
| 403 | Liuzhou | Sanjiang County | Lauraceae | *Camphora officinarum Nees* | 500 | 18.0 | 220.0 | 19.5 | Villages and farmlands | Moderate |
| 404 | Liuzhou | Sanjiang County | Moraceae | *Ficus concinna (*Miq.) Miq. | 500 | 20.0 | 259.0 | 30.5 | Villages and farmlands | Moderate |
| 405 | Liuzhou | Sanjiang County | Moraceae | *Ficus concinna (*Miq.) Miq. | 620 | 19.0 | 280.0 | 31.0 | Villages and farmlands | Moderate |
| 406 | Liuzhou | Sanjiang County | Moraceae | *Ficus concinna (*Miq.) Miq. | 620 | 18.0 | 280.0 | 34.5 | Villages and farmlands | Moderate |
| 407 | Liuzhou | Sanjiang County | Moraceae | *Ficus concinna (*Miq.) Miq. | 500 | 16.0 | 257.0 | 27.5 | Villages and farmlands | Moderate |
| 408 | Liuzhou | Sanjiang County | Lauraceae | *Camphora officinarum Nees* | 500 | 25.0 | 220.0 | 19.0 | Villages and farmlands | Moderate |
| 409 | Liuzhou | Sanjiang County | Moraceae | *Ficus concinna (*Miq.) Miq. | 500 | 18.0 | 258.0 | 20.0 | Villages and farmlands | Moderate |
| 410 | Liuzhou | Sanjiang County | Lauraceae | *Camphora officinarum Nees* | 500 | 22.0 | 230.0 | 18.5 | Villages and farmlands | Moderate |
| 411 | Liuzhou | Sanjiang County | Theaceae | *Schima superba* Gardner & Champ. | 500 | 22.0 | 120.0 | 15.5 | Villages and farmlands | Good |
| 412 | Liuzhou | Sanjiang County | Theaceae | *Schima superba* Gardner & Champ. | 550 | 20.0 | 126.0 | 16.0 | Villages and farmlands | Good |
| 413 | Liuzhou | Sanjiang County | Theaceae | *Schima superba* Gardner & Champ. | 500 | 22.0 | 120.0 | 12.5 | Villages and farmlands | Good |
| 414 | Liuzhou | Sanjiang County | Moraceae | *Ficus concinna (*Miq.) Miq. | 500 | 17.0 | 258.0 | 27.5 | Villages and farmlands | Moderate |
| 415 | Liuzhou | Sanjiang County | Moraceae | *Ficus concinna (*Miq.) Miq. | 600 | 15.0 | 275.2 | 25.0 | Villages and farmlands | Moderate |
| 416 | Liuzhou | Sanjiang County | Moraceae | *Ficus concinna (*Miq.) Miq. | 500 | 24.0 | 275.3 | 38.0 | Villages and farmlands | Moderate |
| 417 | Liuzhou | Sanjiang County | Moraceae | *Ficus concinna (*Miq.) Miq. | 850 | 12.0 | 302.5 | 19.0 | Villages and farmlands | Moderate |
| 418 | Liuzhou | Sanjiang County | Moraceae | *Ficus concinna (*Miq.) Miq. | 700 | 14.0 | 286.2 | 29.0 | Villages and farmlands | Moderate |
| 419 | Liuzhou | Sanjiang County | Theaceae | *Schima superba* Gardner & Champ. | 600 | 24.0 | 130.5 | 17.0 | Villages and farmlands | Good |
| 420 | Liuzhou | Sanjiang County | Theaceae | *Schima superba* Gardner & Champ. | 500 | 21.5 | 120.9 | 15.0 | Villages and farmlands | Good |
| 421 | Liuzhou | Sanjiang County | Moraceae | *Ficus concinna (*Miq.) Miq. | 700 | 21.0 | 289.0 | 24.5 | Villages and farmlands | Moderate |
| 422 | Liuzhou | Sanjiang County | Theaceae | *Schima superba* Gardner & Champ. | 500 | 16.0 | 119.0 | 7.5 | Villages and farmlands | Moderate |
| 423 | Liuzhou | Sanjiang County | Podocarpaceae | *Dacrycarpus imbricatus* (Blume) de Laub. | 850 | 21.0 | 138.0 | 11.5 | Villages and farmlands | Good |
| 424 | Liuzhou | Sanjiang County | Podocarpaceae | *Dacrycarpus imbricatus* (Blume) de Laub. | 500 | 18.0 | 105.0 | 11.5 | Villages and farmlands | Good |
| 425 | Liuzhou | Sanjiang County | Fagaceae | *Castanopsis hystrix* Hook. f. & Thomson ex A. DC. | 500 | 21.0 | 143.0 | 14.0 | Villages and farmlands | Good |
| 426 | Liuzhou | Sanjiang County | Moraceae | *Ficus concinna (*Miq.) Miq. | 500 | 25.0 | 254.0 | 40.0 | Villages and farmlands | Good |
| 427 | Liuzhou | Sanjiang County | Theaceae | *Schima superba* Gardner & Champ. | 550 | 30.0 | 124.9 | 15.0 | Villages and farmlands | Good |
| 428 | Liuzhou | Sanjiang County | Fagaceae | *Castanopsis hystrix* Hook. f. & Thomson ex A. DC. | 500 | 30.0 | 141.1 | 12.0 | Villages and farmlands | Good |
| 429 | Liuzhou | Sanjiang County | Moraceae | *Ficus concinna (*Miq.) Miq. | 600 | 28.0 | 283.0 | 36.5 | Villages and farmlands | Good |
| 430 | Liuzhou | Sanjiang County | Moraceae | *Ficus concinna (*Miq.) Miq. | 750 | 25.0 | 298.0 | 40.0 | Villages and farmlands | Good |
| 431 | Liuzhou | Sanjiang County | Moraceae | *Ficus concinna (*Miq.) Miq. | 500 | 20.0 | 253.0 | 41.0 | Villages and farmlands | Good |
| 432 | Liuzhou | Sanjiang County | Moraceae | *Ficus concinna (*Miq.) Miq. | 500 | 30.0 | 265.2 | 30.0 | Villages and farmlands | Good |
| 433 | Liuzhou | Sanjiang County | Moraceae | *Ficus concinna (*Miq.) Miq. | 500 | 29.0 | 263.4 | 35.0 | Villages and farmlands | Good |
| 434 | Liuzhou | Sanjiang County | Moraceae | *Ficus concinna (*Miq.) Miq. | 500 | 30.0 | 258.2 | 37.5 | Villages and farmlands | Good |
| 435 | Liuzhou | Sanjiang County | Moraceae | *Ficus concinna (*Miq.) Miq. | 500 | 18.0 | 254.0 | 27.0 | Residential districts | Good |
| 436 | Liuzhou | Sanjiang County | Moraceae | *Ficus concinna (*Miq.) Miq. | 600 | 26.0 | 281.0 | 36.5 | Villages and farmlands | Good |
| 437 | Liuzhou | Sanjiang County | Moraceae | *Ficus concinna (*Miq.) Miq. | 500 | 25.0 | 252.0 | 32.5 | Villages and farmlands | Good |
| 438 | Liuzhou | Sanjiang County | Moraceae | *Ficus concinna (*Miq.) Miq. | 500 | 18.0 | 249.0 | 27.0 | Villages and farmlands | Good |
| 439 | Liuzhou | Sanjiang County | Moraceae | *Ficus concinna (*Miq.) Miq. | 500 | 18.0 | 244.0 | 35.5 | Villages and farmlands | Good |
| 440 | Liuzhou | Sanjiang County | Moraceae | *Ficus concinna (*Miq.) Miq. | 750 | 20.0 | 297.0 | 34.0 | Villages and farmlands | Good |
| 441 | Liuzhou | Sanjiang County | Magnoliaceae | *Liriodendron chinense* (Hemsl.) Sarg. | 500 | 16.0 | 92.0 | 14.5 | Villages and farmlands | Moderate |
| 442 | Liuzhou | Sanjiang County | Lamiaceae | *Vitex quinata* (Lour.) Will. | 500 | 15.0 | 175.0 | 15.5 | Villages and farmlands | Good |
| 443 | Liuzhou | Sanjiang County | Moraceae | *Ficus concinna (*Miq.) Miq. | 800 | 13.0 | 136.0 | 10.5 | Villages and farmlands | Moderate |
| 444 | Liuzhou | Sanjiang County | Moraceae | *Ficus concinna (*Miq.) Miq. | 800 | 12.0 | 79.9 | 7.5 | Villages and farmlands | Moderate |
| 445 | Liuzhou | Sanjiang County | Moraceae | *Ficus concinna (*Miq.) Miq. | 800 | 11.0 | 127.0 | 6.5 | Villages and farmlands | Moderate |
| 446 | Liuzhou | Sanjiang County | Moraceae | *Ficus concinna (*Miq.) Miq. | 800 | 14.8 | 135.9 | 37.5 | Villages and farmlands | Moderate |
| 447 | Liuzhou | Sanjiang County | Moraceae | *Ficus concinna (*Miq.) Miq. | 800 | 10.0 | 167.4 | 17.0 | Villages and farmlands | Moderate |
| 448 | Liuzhou | Sanjiang County | Moraceae | *Ficus concinna (*Miq.) Miq. | 800 | 14.0 | 155.2 | 21.0 | Villages and farmlands | Moderate |
| 449 | Liuzhou | Sanjiang County | Moraceae | *Ficus concinna (*Miq.) Miq. | 800 | 8.0 | 146.0 | 7.0 | Villages and farmlands | Moderate |
| 450 | Liuzhou | Sanjiang County | Moraceae | *Ficus concinna (*Miq.) Miq. | 500 | 14.5 | 95.2 | 16.5 | Villages and farmlands | Good |
| 451 | Liuzhou | Sanjiang County | Moraceae | *Ficus concinna (*Miq.) Miq. | 510 | 25.0 | 262.3 | 24.0 | Villages and farmlands | Moderate |
| 452 | Liuzhou | Sanjiang County | Moraceae | *Ficus concinna (*Miq.) Miq. | 550 | 28.0 | 272.8 | 36.0 | Villages and farmlands | Moderate |
| 453 | Liuzhou | Sanjiang County | Moraceae | *Ficus concinna (*Miq.) Miq. | 800 | 20.5 | 95.7 | 19.0 | Villages and farmlands | Moderate |
| 454 | Guilin | Lingui District | Moraceae | *Ficus concinna* (Miq.) Miq. | 1000 | 32.0 | 347.8 | 28.0 | Villages and farmlands | Good |
| 455 | Guilin | Lingui District | Moraceae | *Ficus concinna* (Miq.) Miq. | 1000 | 23.0 | 292.0 | 39.0 | Villages and farmlands | Good |
| 456 | Guilin | Lingui District | Moraceae | *Ficus microcarpa* L. f. | 1000 | 23.0 | 302.5 | 30.0 | Villages and farmlands | Good |
| 457 | Guilin | Lingui District | Moraceae | *Ficus concinna* (Miq.) Miq. | 1000 | 26.0 | 366.0 | 33.8 | Villages and farmlands | Good |
| 458 | Guilin | Lingui District | Moraceae | *Ficus concinna* (Miq.) Miq. | 1200 | 18.9 | 471.3 | 32.5 | Villages and farmlands | Good |
| 459 | Guilin | Lingui District | Moraceae | *Ficus microcarpa* L. f. | 1000 | 22.0 | 356.0 | 46.5 | Villages and farmlands | Good |
| 460 | Guilin | Lingui District | Moraceae | *Ficus concinna* (Miq.) Miq. | 1000 | 12.5 | 353.0 | 31.0 | Villages and farmlands | Good |
| 461 | Guilin | Lingui District | Moraceae | *Ficus concinna* (Miq.) Miq. | 1000 | 27.5 | 451.0 | 42.9 | Villages and farmlands | Good |
| 462 | Guilin | Lingui District | Lauraceae | *Ficus concinna* (Miq.) Miq. | 1000 | 19.0 | 305.1 | 37.5 | Villages and farmlands | Good |
| 463 | Guilin | Lingui District | Moraceae | *Camphora officinarum Nees.* | 1000 | 23.6 | 366.2 | 33.7 | Villages and farmlands | Good |
| 464 | Guilin | Lingui District | Moraceae | *Ficus concinna* (Miq.) Miq. | 1000 | 21.3 | 340.8 | 38.4 | Villages and farmlands | Good |
| 465 | Guilin | Lingui District | Lamiaceae | *Ficus concinna* (Miq.) Miq. | 1000 | 21.2 | 136.9 | 16.7 | Villages and farmlands | Good |
| 466 | Guilin | Lingui District | Juglandaceae | *Vitex quinata* (Lour.) Will. | 1000 | 25.8 | 233.4 | 25.2 | Villages and farmlands | Good |
| 467 | Guilin | Lingui District | Moraceae | *Pterocarya stenoptera* C. DC. | 1000 | 16.9 | 311.0 | 42.2 | Villages and farmlands | Good |
| 468 | Guilin | Lingui District | Moraceae | *Ficus concinna* (Miq.) Miq. | 1000 | 25.5 | 366.0 | 36.4 | Villages and farmlands | Good |
| 469 | Guilin | Lingui District | Moraceae | *Ficus concinna* (Miq.) Miq. | 1000 | 35.0 | 329.9 | 42.4 | Villages and farmlands | Good |
| 470 | Guilin | Lingui District | Oleaceae | *Ficus concinna* (Miq.) Miq. | 1000 | 13.8 | 114.3 | 19.0 | Villages and farmlands | Good |
| 471 | Guilin | Yangshuo County | Ginkgoaceae | *Osmanthus fragrans* (Thunb.) Lour. | 1000 | 28.0 | 179.0 | 12.0 | Villages and farmlands | Moderate |
| 472 | Guilin | Yangshuo County | Lauraceae | *Ginkgo biloba* L. | 1600 | 25.0 | 283.0 | 45.0 | Villages and farmlands | Good |
| 473 | Guilin | Yangshuo County | Moraceae | *Camphora officinarum Nees.* | 1505 | 18.0 | 286.5 | 40.8 | Residential districts | Good |
| 474 | Guilin | Lingchuan County | Lauraceae | *Ficus microcarpa* L. f. | 1000 | 38.0 | 300.0 | 32.5 | Villages and farmlands | Good |
| 475 | Guilin | Lingchuan County | Juglandaceae | *Camphora officinarum Nees* | 1000 | 18.0 | 229.3 | 20.0 | Villages and farmlands | Poor |
| 476 | Guilin | Lingchuan County | Lauraceae | *Pterocarya stenoptera* C. DC. | 1000 | 30.0 | 314.0 | 43.5 | Villages and farmlands | Good |
| 477 | Guilin | Lingchuan County | Ginkgoaceae | *Camphora officinarum Nees.* | 1400 | 30.0 | 172.0 | 17.5 | Villages and farmlands | Moderate |
| 478 | Guilin | Lingchuan County | Ginkgoaceae | *Ginkgo biloba* L. | 1000 | 21.0 | 153.5 | 11.0 | Villages and farmlands | Moderate |
| 479 | Guilin | Lingchuan County | Ginkgoaceae | *Ginkgo biloba* L. | 1000 | 32.0 | 172.0 | 21.5 | Villages and farmlands | Good |
| 480 | Guilin | Lingchuan County | Phyllanthaceae | *Ginkgo biloba* L. | 1000 | 25.0 | 293.6 | 35.0 | Villages and farmlands | Moderate |
| 481 | Guilin | Lingchuan County | Lauraceae | *Camphora officinarum Nees.* | 1000 | 36.0 | 358.6 | 28.5 | Villages and farmlands | Good |
| 482 | Guilin | Quanzhou County | Lauraceae | *Camphora officinarum Nees.* | 1050 | 26.0 | 334.0 | 39.0 | Villages and farmlands | Good |
| 483 | Guilin | Quanzhou County | Ginkgoaceae | *Ginkgo biloba* L. | 1000 | 24.0 | 333.3 | 15.5 | Villages and farmlands | Good |
| 484 | Guilin | Quanzhou County | Taxaceae | *Taxus wallichiana* var. *mairei* (Lemée & H. Lév.) L. K. Fu & Nan Li | 1200 | 22.0 | 162.4 | 23.5 | Villages and farmlands | Good |
| 485 | Guilin | Quanzhou County | Fagaceae | *Quercus acutissima* Carruth. | 1300 | 20.0 | 136.9 | 29.0 | Villages and farmlands | Good |
| 486 | Guilin | Xing'an County | Lauraceae | *Camphora officinarum Nees.* | 1805 | 20.0 | 423.0 | 18.0 | Villages and farmlands | Moderate |
| 487 | Guilin | Xing'an County | Ginkgoaceae | *Ginkgo biloba* L. | 1200 | 5.0 | 120.0 | 5.5 | Villages and farmlands | Good |
| 488 | Guilin | Yongfu County | Phyllanthaceae | *Bischofia polycarpa* (H. Lév.) Airy Shaw | 1005 | 16.0 | 185.0 | 22.3 | Villages and farmlands | Good |
| 489 | Guilin | Guanyang County | Lauraceae | *Camphora officinarum Nees.* | 1000 | 29.0 | 302.0 | 39.8 | Villages and farmlands | Good |
| 490 | Guilin | Guanyang County | Lauraceae | *Camphora officinarum Nees.* | 1000 | 30.0 | 340.0 | 24.0 | Villages and farmlands | Good |
| 491 | Guilin | Guanyang County | Phyllanthaceae | *Bischofia polycarpa* (H. Lév.) Airy Shaw | 1000 | 25.0 | 225.0 | 23.0 | Villages and farmlands | Good |
| 492 | Guilin | Guanyang County | Taxaceae | *Taxus wallichiana* var. *mairei* (Lemée & H. Lév.) L. K. Fu & Nan Li | 1000 | 15.0 | 116.0 | 16.0 | Villages and farmlands | Good |
| 493 | Guilin | Guanyang County | Taxaceae | *Taxus wallichiana* var. *mairei* (Lemée & H. Lév.) L. K. Fu & Nan Li | 1000 | 23.0 | 135.0 | 13.5 | Villages and farmlands | Good |
| 494 | Guilin | Guanyang County | Pinaceae | *Nothotsuga longibracteata* (W. C. Cheng) Hu ex C. N. Page | 1500 | 24.0 | 118.5 | 13.0 | Wooded areas and plant nurseries | Poor |
| 495 | Guilin | Guanyang County | Pinaceae | *Nothotsuga longibracteata* (W. C. Cheng) Hu ex C. N. Page | 1500 | 21.0 | 114.6 | 13.0 | Wooded areas and plant nurseries | Poor |
| 496 | Guilin | Guanyang County | Pinaceae | *Nothotsuga longibracteata* (W. C. Cheng) Hu ex C. N. Page | 1300 | 28.0 | 81.0 | 10.5 | Wooded areas and plant nurseries | Good |
| 497 | Guilin | Guanyang County | Pinaceae | *Nothotsuga longibracteata* (W. C. Cheng) Hu ex C. N. Page | 1200 | 18.0 | 72.0 | 9.5 | Wooded areas and plant nurseries | Moderate |
| 498 | Guilin | Guanyang County | Pinaceae | *Nothotsuga longibracteata* (W. C. Cheng) Hu ex C. N. Page | 1000 | 28.0 | 75.7 | 11.0 | Wooded areas and plant nurseries | Good |
| 499 | Guilin | Guanyang County | Pinaceae | *Nothotsuga longibracteata* (W. C. Cheng) Hu ex C. N. Page | 1000 | 14.0 | 74.5 | 7.0 | Wooded areas and plant nurseries | Good |
| 500 | Guilin | Longsheng County | Pinaceae | *Nothotsuga longibracteata* (W. C. Cheng) Hu ex C. N. Page | 1100 | 20.0 | 112.0 | 12.0 | Villages and farmlands | Good |
| 501 | Guilin | Longsheng County | Pinaceae | *Pinus kwangtungensis* Chun ex Tsiang. | 1800 | 20.0 | 81.0 | 12.5 | Villages and farmlands | Good |
| 502 | Guilin | Longsheng County | Taxaceae | *Taxus wallichiana* var. *mairei* (Lemée & H. Lév.) L. K. Fu & Nan Li | 1200 | 28.0 | 172.0 | 23.5 | Villages and farmlands | Good |
| 503 | Guilin | Ziyuan County | Taxaceae | *Taxus wallichiana* var. *mairei* (Lemée & H. Lév.) L. K. Fu & Nan Li | 1000 | 21.0 | 130.0 | 12.0 | Villages and farmlands | Good |
| 504 | Guilin | Ziyuan County | Fagaceae | *Quercus variabilis* Blume. | 1200 | 27.0 | 149.0 | 21.5 | Villages and farmlands | Poor |
| 505 | Guilin | Ziyuan County | Fagaceae | *Quercus variabilis* Blume. | 1000 | 25.0 | 130.0 | 11.0 | Villages and farmlands | Poor |
| 506 | Guilin | Ziyuan County | Juglandaceae | *Pterocarya stenoptera* C. DC. | 1000 | 19.0 | 220.0 | 8.5 | Villages and farmlands | Good |
| 507 | Guilin | Ziyuan County | Pinaceae | *Tsuga chinensis* (Franch.) E. Pritz. | 1000 | 10.0 | 68.8 | 9.0 | Wooded areas and plant nurseries | Good |
| 508 | Guilin | Ziyuan County | Pinaceae | *Nothotsuga longibracteata* (W. C. Cheng) Hu ex C. N. Page | 1300 | 28.0 | 123.3 | 11.5 | Wooded areas and plant nurseries | Good |
| 509 | Guilin | Ziyuan County | Pinaceae | *Tsuga chinensis* (Franch.) E. Pritz. | 1200 | 14.0 | 104.8 | 11.0 | Wooded areas and plant nurseries | Good |
| 510 | Guilin | Ziyuan County | Pinaceae | *Nothotsuga longibracteata* (W. C. Cheng) Hu ex C. N. Page | 1000 | 13.6 | 100.0 | 12.0 | Wooded areas and plant nurseries | Good |
| 511 | Guilin | Ziyuan County | Pinaceae | *Nothotsuga longibracteata* (W. C. Cheng) Hu ex C. N. Page | 1000 | 30.0 | 95.8 | 15.0 | Wooded areas and plant nurseries | Good |
| 512 | Guilin | Ziyuan County | Pinaceae | *Tsuga chinensis* (Franch.) E. Pritz. | 1100 | 13.0 | 93.0 | 11.5 | Wooded areas and plant nurseries | Good |
| 513 | Guilin | Ziyuan County | Pinaceae | *Nothotsuga longibracteata* (W. C. Cheng) Hu ex C. N. Page | 1000 | 25.0 | 90.0 | 10.0 | Wooded areas and plant nurseries | Good |
| 514 | Guilin | Ziyuan County | Pinaceae | *Nothotsuga longibracteata* (W. C. Cheng) Hu ex C. N. Page | 1000 | 28.0 | 90.0 | 16.0 | Wooded areas and plant nurseries | Good |
| 515 | Guilin | Ziyuan County | Pinaceae | *Tsuga chinensis* (Franch.) E. Pritz. | 1000 | 15.0 | 89.4 | 11.9 | Wooded areas and plant nurseries | Good |
| 516 | Guilin | Ziyuan County | Pinaceae | *Tsuga chinensis* (Franch.) E. Pritz. | 1000 | 14.0 | 88.0 | 12.0 | Wooded areas and plant nurseries | Good |
| 517 | Guilin | Ziyuan County | Pinaceae | *Nothotsuga longibracteata* (W. C. Cheng) Hu ex C. N. Page | 1000 | 26.0 | 87.9 | 15.3 | Wooded areas and plant nurseries | Good |
| 518 | Guilin | Ziyuan County | Pinaceae | *Nothotsuga longibracteata* (W. C. Cheng) Hu ex C. N. Page | 1000 | 28.0 | 87.7 | 11.0 | Wooded areas and plant nurseries | Good |
| 519 | Guilin | Ziyuan County | Pinaceae | *Tsuga chinensis* (Franch.) E. Pritz. | 1000 | 18.0 | 85.6 | 10.0 | Wooded areas and plant nurseries | Good |
| 520 | Guilin | Ziyuan County | Pinaceae | *Nothotsuga longibracteata* (W. C. Cheng) Hu ex C. N. Page | 1000 | 23.0 | 85.5 | 13.5 | Wooded areas and plant nurseries | Good |
| 521 | Guilin | Ziyuan County | Pinaceae | *Tsuga chinensis* (Franch.) E. Pritz. | 1000 | 20.0 | 85.5 | 10.0 | Wooded areas and plant nurseries | Good |
| 522 | Guilin | Ziyuan County | Pinaceae | *Tsuga chinensis* (Franch.) E. Pritz. | 1000 | 29.0 | 85.0 | 11.0 | Wooded areas and plant nurseries | Good |
| 523 | Guilin | Ziyuan County | Pinaceae | *Nothotsuga longibracteata* (W. C. Cheng) Hu ex C. N. Page | 1000 | 29.0 | 85.0 | 14.0 | Wooded areas and plant nurseries | Good |
| 524 | Guilin | Ziyuan County | Pinaceae | *Tsuga chinensis* (Franch.) E. Pritz. | 1000 | 16.0 | 85.0 | 10.5 | Wooded areas and plant nurseries | Good |
| 525 | Guilin | Ziyuan County | Pinaceae | *Tsuga chinensis* (Franch.) E. Pritz. | 1000 | 18.0 | 85.0 | 10.0 | Wooded areas and plant nurseries | Good |
| 526 | Guilin | Ziyuan County | Pinaceae | *Tsuga chinensis* (Franch.) E. Pritz. | 1000 | 18.0 | 85.0 | 10.0 | Wooded areas and plant nurseries | Good |
| 527 | Guilin | Ziyuan County | Pinaceae | *Tsuga chinensis* (Franch.) E. Pritz. | 1000 | 18.0 | 85.0 | 10.5 | Wooded areas and plant nurseries | Good |
| 528 | Guilin | Ziyuan County | Pinaceae | *Tsuga chinensis* (Franch.) E. Pritz. | 1000 | 18.0 | 85.0 | 10.5 | Wooded areas and plant nurseries | Good |
| 529 | Guilin | Ziyuan County | Pinaceae | *Tsuga chinensis* (Franch.) E. Pritz. | 1000 | 18.0 | 85.0 | 10.5 | Wooded areas and plant nurseries | Good |
| 530 | Guilin | Ziyuan County | Pinaceae | *Nothotsuga longibracteata* (W. C. Cheng) Hu ex C. N. Page | 1000 | 29.0 | 84.0 | 14.4 | Wooded areas and plant nurseries | Good |
| 531 | Guilin | Ziyuan County | Pinaceae | *Tsuga chinensis* (Franch.) E. Pritz. | 1000 | 14.0 | 83.0 | 11.5 | Wooded areas and plant nurseries | Good |
| 532 | Guilin | Ziyuan County | Pinaceae | *Tsuga chinensis* (Franch.) E. Pritz. | 1000 | 15.0 | 83.0 | 12.0 | Wooded areas and plant nurseries | Good |
| 533 | Guilin | Ziyuan County | Pinaceae | *Tsuga chinensis* (Franch.) E. Pritz. | 1000 | 12.4 | 83.0 | 11.1 | Wooded areas and plant nurseries | Good |
| 534 | Guilin | Ziyuan County | Pinaceae | *Tsuga chinensis* (Franch.) E. Pritz. | 1000 | 12.3 | 82.0 | 10.6 | Wooded areas and plant nurseries | Good |
| 535 | Guilin | Ziyuan County | Pinaceae | *Tsuga chinensis* (Franch.) E. Pritz. | 1000 | 11.0 | 81.0 | 9.0 | Wooded areas and plant nurseries | Good |
| 536 | Guilin | Ziyuan County | Pinaceae | *Tsuga chinensis* (Franch.) E. Pritz. | 1000 | 16.0 | 80.3 | 11.9 | Wooded areas and plant nurseries | Good |
| 537 | Guilin | Ziyuan County | Pinaceae | *Nothotsuga longibracteata* (W. C. Cheng) Hu ex C. N. Page | 1000 | 28.0 | 80.0 | 14.5 | Wooded areas and plant nurseries | Good |
| 538 | Guilin | Ziyuan County | Pinaceae | *Nothotsuga longibracteata* (W. C. Cheng) Hu ex C. N. Page | 1000 | 26.0 | 80.0 | 13.5 | Wooded areas and plant nurseries | Good |
| 539 | Guilin | Ziyuan County | Pinaceae | *Tsuga chinensis* (Franch.) E. Pritz. | 1000 | 14.0 | 80.0 | 11.0 | Wooded areas and plant nurseries | Good |
| 540 | Guilin | Ziyuan County | Pinaceae | *Tsuga chinensis* (Franch.) E. Pritz. | 1000 | 13.0 | 80.0 | 10.5 | Wooded areas and plant nurseries | Good |
| 541 | Guilin | Ziyuan County | Pinaceae | *Tsuga chinensis* (Franch.) E. Pritz. | 1000 | 12.0 | 79.9 | 9.8 | Wooded areas and plant nurseries | Good |
| 542 | Guilin | Ziyuan County | Pinaceae | *Tsuga chinensis* (Franch.) E. Pritz. | 1000 | 13.0 | 79.0 | 10.0 | Wooded areas and plant nurseries | Good |
| 543 | Guilin | Ziyuan County | Pinaceae | *Tsuga chinensis* (Franch.) E. Pritz. | 1200 | 25.0 | 146.0 | 9.5 | Wooded areas and plant nurseries | Good |
| 544 | Guilin | Ziyuan County | Pinaceae | *Tsuga chinensis* (Franch.) E. Pritz. | 1100 | 18.0 | 97.0 | 7.5 | Wooded areas and plant nurseries | Good |
| 545 | Guilin | Ziyuan County | Pinaceae | *Tsuga chinensis* (Franch.) E. Pritz. | 1000 | 20.0 | 85.0 | 9.0 | Wooded areas and plant nurseries | Good |
| 546 | Guilin | Ziyuan County | Pinaceae | *Tsuga chinensis* (Franch.) E. Pritz. | 1000 | 17.0 | 77.0 | 10.8 | Wooded areas and plant nurseries | Good |
| 547 | Guilin | Ziyuan County | Pinaceae | *Tsuga chinensis* (Franch.) E. Pritz. | 1000 | 24.0 | 88.0 | 7.0 | Wooded areas and plant nurseries | Good |
| 548 | Guilin | Pingle County | Lauraceae | *Camphora officinarum Nees.* | 1020 | 18.6 | 380.2 | 34.2 | Villages and farmlands | Good |
| 549 | Guilin | Pingle County | Moraceae | *Ficus concinna* (Miq.) Miq. | 1050 | 15.0 | 420.0 | 42.0 | Residential districts | Good |
| 550 | Guilin | Pingle County | Moraceae | *Ficus concinna* (Miq.) Miq. | 1000 | 15.9 | 290.2 | 38.2 | Residential districts | Good |
| 551 | Guilin | Pingle County | Moraceae | *Ficus concinna* (Miq.) Miq. | 1000 | 16.5 | 300.2 | 33.0 | Residential districts | Good |
| 552 | Guilin | Pingle County | Moraceae | *Ficus concinna* (Miq.) Miq. | 1000 | 14.5 | 290.0 | 32.5 | Residential districts | Good |
| 553 | Guilin | Pingle County | Moraceae | *Ficus concinna* (Miq.) Miq. | 1000 | 18.5 | 340.0 | 30.0 | Villages and farmlands | Good |
| 554 | Guilin | Pingle County | Lauraceae | *Camphora officinarum Nees.* | 1000 | 22.5 | 314.0 | 22.6 | Villages and farmlands | Good |
| 555 | Guilin | Pingle County | Moraceae | *Ficus concinna* (Miq.) Miq. | 1000 | 21.0 | 330.9 | 30.0 | Villages and farmlands | Good |
| 556 | Guilin | Pingle County | Moraceae | *Ficus concinna* (Miq.) Miq. | 1000 | 27.0 | 365.0 | 51.0 | Villages and farmlands | Good |
| 557 | Guilin | Pingle County | Moraceae | *Ficus concinna* (Miq.) Miq. | 1000 | 31.0 | 325.0 | 31.0 | Villages and farmlands | Good |
| 558 | Guilin | Pingle County | Moraceae | *Ficus concinna* (Miq.) Miq. | 1000 | 22.0 | 337.0 | 19.0 | Villages and farmlands | Good |
| 559 | Guilin | Pingle County | Moraceae | *Ficus concinna* (Miq.) Miq. | 1000 | 25.0 | 312.0 | 39.0 | Villages and farmlands | Good |
| 560 | Guilin | Pingle County | Moraceae | *Ficus concinna* (Miq.) Miq. | 1000 | 24.0 | 376.0 | 36.0 | Villages and farmlands | Good |
| 561 | Guilin | Pingle County | Moraceae | *Ficus concinna* (Miq.) Miq. | 1000 | 25.0 | 350.0 | 30.0 | Villages and farmlands | Good |
| 562 | Guilin | Pingle County | Moraceae | *Ficus concinna* (Miq.) Miq. | 1000 | 25.0 | 350.0 | 27.5 | Villages and farmlands | Good |
| 563 | Guilin | Lipu City | Lauraceae | *Phoebe bournei* (Hemsl.) Yen C. Yang | 1050 | 32.0 | 197.0 | 25.0 | Wooded areas and plant nurseries | Good |
| 564 | Guilin | Lipu City | Moraceae | *Ficus concinna* (Miq.) Miq. | 1200 | 37.0 | 438.9 | 15.5 | Residential districts | Good |
| 565 | Guilin | Lipu City | Moraceae | *Ficus concinna* (Miq.) Miq. | 1500 | 38.0 | 496.9 | 25.5 | Residential districts | Good |
| 566 | Guilin | Lipu City | Moraceae | *Ficus concinna* (Miq.) Miq. | 1000 | 39.0 | 306.0 | 40.5 | Villages and farmlands | Good |
| 567 | Guilin | Lipu City | Lauraceae | *Camphora officinarum Nees.* | 1000 | 6.5 | 296.6 | 16.0 | Villages and farmlands | Good |
| 568 | Guilin | Lipu City | Moraceae | *Ficus concinna* (Miq.) Miq. | 1050 | 22.4 | 401.3 | 41.0 | Villages and farmlands | Good |
| 569 | Guilin | Lipu City | Moraceae | *Ficus concinna* (Miq.) Miq. | 1000 | 16.2 | 308.9 | 30.0 | Villages and farmlands | Good |
| 570 | Guilin | Lipu City | Lauraceae | *Camphora officinarum Nees* | 1000 | 15.0 | 360.0 | 6.5 | Villages and farmlands | Good |
| 571 | Guilin | Gongcheng County | Pinaceae | *Keteleeria pubescens* W. C. Cheng & L. K. Fu | 1000 | 35.0 | 200.0 | 19.0 | Villages and farmlands | Good |
| 572 | Guilin | Gongcheng County | Pinaceae | *Keteleeria pubescens* W. C. Cheng & L. K. Fu | 1000 | 38.0 | 185.0 | 13.0 | Villages and farmlands | Good |
| 573 | Guilin | Xiufeng District | Lauraceae | *Camphora officinarum Nees.* | 760 | 25.8 | 287.0 | 28.5 | Residential districts | Good |
| 574 | Guilin | Xiufeng District | Lauraceae | *Camphora officinarum Nees.* | 500 | 24.4 | 219.0 | 38.0 | Residential districts | Good |
| 575 | Guilin | Xiufeng District | Lauraceae | *Camphora officinarum Nees.* | 680 | 21.2 | 267.0 | 25.0 | Villages and farmlands | Good |
| 576 | Guilin | Xiufeng District | Lauraceae | *Camphora officinarum Nees.* | 570 | 17.8 | 242.0 | 11.0 | Villages and farmlands | Good |
| 577 | Guilin | Diecai District | Lauraceae | *Camphora officinarum Nees.* | 540 | 22.5 | 229.0 | 33.0 | Villages and farmlands | Good |
| 578 | Guilin | Qixing District | Lauraceae | *Camphora officinarum Nees.* | 500 | 11.8 | 220.0 | 7.5 | Residential districts | Good |
| 579 | Guilin | Yanshan District | Lauraceae | *Camphora officinarum Nees.* | 550 | 22.0 | 223.0 | 37.4 | Residential districts | Good |
| 580 | Guilin | Yanshan District | Lauraceae | *Camphora officinarum Nees.* | 500 | 28.0 | 223.0 | 43.0 | Residential districts | Good |
| 581 | Guilin | Yanshan District | Lauraceae | *Camphora officinarum Nees.* | 600 | 20.0 | 253.0 | 22.0 | Villages and farmlands | Good |
| 582 | Guilin | Yanshan District | Phyllanthaceae | *Bischofia polycarpa* (H. Lév.) Airy Shaw | 550 | 19.0 | 235.5 | 22.5 | Villages and farmlands | Moderate |
| 583 | Guilin | Yanshan District | Oleaceae | *Osmanthus fragrans* (Thunb.) Lour. | 510 | 10.0 | 94.3 | 9.5 | Villages and farmlands | Good |
| 584 | Guilin | Lingui District | Lauraceae | *Camphora officinarum Nees.* | 800 | 15.0 | 292.9 | 8.9 | Villages and farmlands | Good |
| 585 | Guilin | Lingui District | Oleaceae | *Fraxinus griffithii* C. B. Clarke | 650 | 9.5 | 98.7 | 10.7 | Villages and farmlands | Good |
| 586 | Guilin | Lingui District | Lauraceae | *Camphora officinarum Nees* | 570 | 32.5 | 241.0 | 42.0 | Villages and farmlands | Good |
| 587 | Guilin | Lingui District | Aquifoliaceae | *Ilex chinensis* Sims. | 500 | 17.2 | 102.2 | 17.4 | Villages and farmlands | Moderate |
| 588 | Guilin | Lingui District | Lamiaceae | *Vitex quinata* (Lour.) Will. | 580 | 27.0 | 96.2 | 22.6 | Villages and farmlands | Good |
| 589 | Guilin | Lingui District | Lauraceae | *Camphora officinarum Nees.* | 736 | 30.0 | 280.0 | 42.8 | Villages and farmlands | Good |
| 590 | Guilin | Lingui District | Lauraceae | *Camphora officinarum Nees.* | 612 | 27.5 | 251.6 | 26.5 | Villages and farmlands | Good |
| 591 | Guilin | Lingui District | Lauraceae | *Camphora officinarum Nees.* | 600 | 23.3 | 253.0 | 27.6 | Villages and farmlands | Good |
| 592 | Guilin | Lingui District | Lauraceae | *Camphora officinarum Nees.* | 640 | 23.0 | 258.6 | 34.0 | Villages and farmlands | Good |
| 593 | Guilin | Lingui District | Proteaceae | *Camphora officinarum Nees.* | 500 | 27.9 | 109.6 | 17.4 | Villages and farmlands | Moderate |
| 594 | Guilin | Lingui District | Lauraceae | *Camphora officinarum Nees.* | 500 | 28.7 | 223.6 | 27.0 | Villages and farmlands | Good |
| 595 | Guilin | Lingui District | Lauraceae | *Camphora officinarum Nees.* | 530 | 29.8 | 228.0 | 27.4 | Villages and farmlands | Good |
| 596 | Guilin | Lingui District | Lauraceae | *Camphora officinarum Nees.* | 576 | 28.2 | 240.4 | 33.6 | Villages and farmlands | Good |
| 597 | Guilin | Lingui District | Lauraceae | *Camphora officinarum Nees.* | 510 | 23.3 | 224.5 | 30.4 | Villages and farmlands | Good |
| 598 | Guilin | Lingui District | Moraceae | *Ficus microcarpa* L. f. | 550 | 16.0 | 266.6 | 28.5 | Villages and farmlands | Good |
| 599 | Guilin | Lingui District | Theaceae | *Schima superba* Gardner & Champ. | 825 | 20.3 | 140.1 | 18.5 | Villages and farmlands | Good |
| 600 | Guilin | Lingui District | Lauraceae | *Camphora officinarum Nees* | 530 | 29.5 | 229.3 | 24.5 | Villages and farmlands | Good |
| 601 | Guilin | Lingui District | Theaceae | *Schima superba* Gardner & Champ. | 500 | 26.2 | 120.0 | 15.6 | Villages and farmlands | Good |
| 602 | Guilin | Lingui District | Moraceae | *Ficus microcarpa* L. f. | 780 | 16.5 | 297.8 | 26.5 | Villages and farmlands | Good |
| 603 | Guilin | Lingui District | Moraceae | *Ficus microcarpa* L. f. | 787 | 20.0 | 298.7 | 23.6 | Villages and farmlands | Good |
| 604 | Guilin | Lingui District | Moraceae | *Ficus microcarpa* L. f. | 635 | 8.1 | 280.0 | 27.2 | Villages and farmlands | Good |
| 605 | Guilin | Lingui District | Lauraceae | *Camphora officinarum Nees* | 502 | 20.1 | 221.3 | 40.0 | Villages and farmlands | Good |
| 606 | Guilin | Lingui District | Moraceae | *Ficus microcarpa* L. f. | 620 | 19.7 | 277.7 | 27.4 | Villages and farmlands | Good |
| 607 | Guilin | Lingui District | Moraceae | *Ficus microcarpa* L. f. | 703 | 23.2 | 289.0 | 34.0 | Villages and farmlands | Good |
| 608 | Guilin | Lingui District | Lauraceae | *Camphora officinarum Nees* | 540 | 18.5 | 232.4 | 17.0 | Villages and farmlands | Moderate |
| 609 | Guilin | Lingui District | Moraceae | *Ficus microcarpa* L. f. | 700 | 22.0 | 296.8 | 31.0 | Villages and farmlands | Moderate |
| 610 | Guilin | Lingui District | Juglandaceae | *Pterocarya stenoptera* C. DC. | 600 | 17.8 | 203.0 | 26.1 | Villages and farmlands | Good |
| 611 | Guilin | Lingui District | Lauraceae | *Camphora officinarum Nees* | 625 | 26.0 | 254.5 | 27.9 | Villages and farmlands | Moderate |
| 612 | Guilin | Lingui District | Lauraceae | *Camphora officinarum Nees* | 620 | 27.0 | 253.2 | 33.1 | Villages and farmlands | Good |
| 613 | Guilin | Lingui District | Lauraceae | *Camphora officinarum Nees* | 825 | 32.0 | 297.1 | 34.5 | Villages and farmlands | Poor |
| 614 | Guilin | Lingui District | Oleaceae | *Osmanthus fragrans* (Thunb.) Lour. | 725 | 11.1 | 100.6 | 16.0 | Villages and farmlands | Good |
| 615 | Guilin | Lingui District | Oleaceae | *Osmanthus fragrans* (Thunb.) Lour. | 795 | 9.8 | 101.9 | 18.2 | Villages and farmlands | Good |
| 616 | Guilin | Lingui District | Cannabaceae | *Celtis biondii* Pamp. | 530 | 15.2 | 156.0 | 13.8 | Villages and farmlands | Good |
| 617 | Guilin | Lingui District | Fagaceae | *Castanopsis carlesii* (Hemsl.) Hayata | 590 | 27.3 | 150.0 | 27.7 | Villages and farmlands | Good |
| 618 | Guilin | Lingui District | Moraceae | *Ficus microcarpa* L. f. | 700 | 19.7 | 315.3 | 32.3 | Villages and farmlands | Good |
| 619 | Guilin | Lingui District | Moraceae | *Ficus microcarpa* L. f. | 742 | 21.4 | 293.0 | 33.8 | Villages and farmlands | Good |
| 620 | Guilin | Lingui District | Lauraceae | *Camphora officinarum Nees.* | 670 | 28.5 | 265.9 | 26.3 | Villages and farmlands | Good |
| 621 | Guilin | Lingui District | Moraceae | *Ficus microcarpa* L. f. | 588 | 19.5 | 272.3 | 25.5 | Villages and farmlands | Good |
| 622 | Guilin | Lingui District | Fagaceae | *Castanopsis chinensis* (Spreng.) Hance | 517 | 21.8 | 139.5 | 19.1 | Villages and farmlands | Good |
| 623 | Guilin | Lingui District | Moraceae | *Ficus microcarpa* L. f. | 630 | 18.1 | 280.2 | 30.0 | Villages and farmlands | Good |
| 624 | Guilin | Lingui District | Cupressaceae | *Platycladus orientalis* (L.) Franco | 540 | 31.4 | 161.8 | 15.6 | Villages and farmlands | Good |
| 625 | Guilin | Lingui District | Pinaceae | *Cathaya argyrophylla* Chun & Kuang | 500 | 21.0 | 79.6 | 14.5 | Wooded areas and plant nurseries | Good |
| 626 | Guilin | Lingui District | Pinaceae | *Cathaya argyrophylla* Chun & Kuang | 500 | 24.0 | 89.2 | 15.5 | Wooded areas and plant nurseries | Good |
| 627 | Guilin | Lingui District | Pinaceae | *Cathaya argyrophylla* Chun & Kuang | 500 | 8.0 | 60.0 | 4.5 | Wooded areas and plant nurseries | Good |
| 628 | Guilin | Lingui District | Pinaceae | *Cathaya argyrophylla* Chun & Kuang | 500 | 15.0 | 62.0 | 8.3 | Wooded areas and plant nurseries | Good |
| 629 | Guilin | Yangshuo County | Lauraceae | *Camphora officinarum Nees.* | 510 | 11.0 | 210.0 | 15.0 | Residential districts | Good |
| 630 | Guilin | Yangshuo County | Lauraceae | *Camphora officinarum Nees.* | 850 | 16.0 | 290.0 | 16.5 | Residential districts | Good |
| 631 | Guilin | Yangshuo County | Phyllanthaceae | *Bischofia polycarpa* (H. Lév.) Airy Shaw | 550 | 16.0 | 235.6 | 12.0 | Villages and farmlands | Good |
| 632 | Guilin | Yangshuo County | Lauraceae | *Camphora officinarum Nees* | 550 | 18.8 | 203.7 | 31.2 | Villages and farmlands | Good |
| 633 | Guilin | Yangshuo County | Fagaceae | *Castanopsis sclerophylla* (Lindl.) Schottky | 700 | 16.2 | 219.0 | 20.5 | Villages and farmlands | Good |
| 634 | Guilin | Yangshuo County | Oleaceae | *Osmanthus fragrans* (Thunb.) Lour. | 550 | 18.5 | 100.3 | 21.1 | Villages and farmlands | Good |
| 635 | Guilin | Yangshuo County | Lauraceae | *Camphora officinarum Nees.* | 550 | 15.0 | 211.7 | 24.5 | Villages and farmlands | Moderate |
| 636 | Guilin | Yangshuo County | Lauraceae | *Camphora officinarum Nees.* | 500 | 23.0 | 220.0 | 21.5 | Villages and farmlands | Moderate |
| 637 | Guilin | Yangshuo County | Lauraceae | *Camphora officinarum Nees.* | 500 | 25.0 | 210.0 | 22.5 | Villages and farmlands | Moderate |
| 638 | Guilin | Yangshuo County | Lauraceae | *Camphora officinarum Nees.* | 600 | 20.0 | 250.0 | 17.5 | Villages and farmlands | Moderate |
| 639 | Guilin | Yangshuo County | Lauraceae | *Camphora officinarum Nees.* | 500 | 18.0 | 199.0 | 11.0 | Villages and farmlands | Moderate |
| 640 | Guilin | Yangshuo County | Lauraceae | *Camphora officinarum Nees.* | 500 | 23.0 | 218.0 | 26.5 | Villages and farmlands | Moderate |
| 641 | Guilin | Yangshuo County | Lauraceae | *Camphora officinarum Nees.* | 650 | 23.0 | 237.6 | 34.0 | Villages and farmlands | Moderate |
| 642 | Guilin | Yangshuo County | Lauraceae | *Camphora officinarum Nees.* | 650 | 20.0 | 238.0 | 31.2 | Villages and farmlands | Moderate |
| 643 | Guilin | Yangshuo County | Lauraceae | *Camphora officinarum Nees.* | 750 | 20.0 | 270.0 | 31.8 | Villages and farmlands | Moderate |
| 644 | Guilin | Yangshuo County | Moraceae | *Ficus microcarpa* L. f. | 500 | 14.0 | 254.6 | 31.0 | Residential districts | Good |
| 645 | Guilin | Yangshuo County | Lauraceae | *Camphora officinarum Nees* | 550 | 9.0 | 222.8 | 10.0 | Villages and farmlands | Good |
| 646 | Guilin | Yangshuo County | Fagaceae | *Castanopsis carlesii* (Hemsl.) Hayata | 510 | 13.0 | 138.5 | 13.5 | Villages and farmlands | Good |
| 647 | Guilin | Yangshuo County | Moraceae | *Ficus microcarpa* L. f. | 500 | 12.0 | 254.6 | 42.5 | Villages and farmlands | Good |
| 648 | Guilin | Yangshuo County | Oleaceae | *Osmanthus fragrans* (Thunb.) Lour. | 600 | 8.0 | 127.3 | 14.5 | Villages and farmlands | Good |
| 649 | Guilin | Yangshuo County | Moraceae | *Ficus microcarpa* L. f. | 500 | 13.0 | 254.6 | 23.5 | Villages and farmlands | Good |
| 650 | Guilin | Yangshuo County | Moraceae | *Ficus microcarpa* L. f. | 500 | 14.0 | 222.8 | 29.0 | Villages and farmlands | Good |
| 651 | Guilin | Yangshuo County | Lauraceae | *Camphora officinarum Nees.* | 550 | 13.0 | 229.2 | 21.3 | Villages and farmlands | Good |
| 652 | Guilin | Yangshuo County | Lauraceae | *Camphora officinarum Nees.* | 720 | 23.0 | 241.9 | 28.5 | Villages and farmlands | Good |
| 653 | Guilin | Yangshuo County | Lauraceae | *Camphora officinarum Nees.* | 700 | 22.0 | 264.2 | 22.3 | Villages and farmlands | Good |
| 654 | Guilin | Yangshuo County | Lauraceae | *Camphora officinarum Nees.* | 550 | 20.0 | 234.0 | 30.0 | Villages and farmlands | Good |
| 655 | Guilin | Yangshuo County | Lauraceae | *Camphora officinarum Nees.* | 500 | 20.0 | 207.0 | 29.0 | Villages and farmlands | Good |
| 656 | Guilin | Yangshuo County | Lauraceae | *Camphora officinarum Nees.* | 530 | 25.0 | 227.6 | 31.5 | Villages and farmlands | Good |
| 657 | Guilin | Yangshuo County | Anacardiaceae | *Choerospondias axillaris* (Roxb.) B. L. Burtt & A. W. Hill | 500 | 20.0 | 229.2 | 16.8 | Villages and farmlands | Good |
| 658 | Guilin | Yangshuo County | Lauraceae | *Camphora officinarum Nees.* | 510 | 25.0 | 216.5 | 23.7 | Villages and farmlands | Good |
| 659 | Guilin | Yangshuo County | Lauraceae | *Camphora officinarum Nees.* | 500 | 20.0 | 219.6 | 24.8 | Villages and farmlands | Good |
| 660 | Guilin | Lingchuan County | Lauraceae | *Camphora officinarum Nees.* | 600 | 20.0 | 216.6 | 23.0 | Villages and farmlands | Good |
| 661 | Guilin | Lingchuan County | Lauraceae | *Camphora officinarum Nees.* | 500 | 30.0 | 187.3 | 20.0 | Villages and farmlands | Good |
| 662 | Guilin | Lingchuan County | Lauraceae | *Camphora officinarum Nees.* | 500 | 20.0 | 184.7 | 18.0 | Villages and farmlands | Good |
| 663 | Guilin | Lingchuan County | Lauraceae | *Camphora officinarum Nees.* | 500 | 7.0 | 195.5 | 5.0 | Villages and farmlands | Good |
| 664 | Guilin | Lingchuan County | Lauraceae | *Camphora officinarum Nees.* | 800 | 28.0 | 248.4 | 45.5 | Villages and farmlands | Good |
| 665 | Guilin | Lingchuan County | Lauraceae | *Camphora officinarum Nees.* | 600 | 28.0 | 192.4 | 26.0 | Villages and farmlands | Good |
| 666 | Guilin | Lingchuan County | Lauraceae | *Camphora officinarum Nees.* | 600 | 25.0 | 248.4 | 17.5 | Villages and farmlands | Good |
| 667 | Guilin | Lingchuan County | Lauraceae | *Camphora officinarum Nees.* | 500 | 26.0 | 191.1 | 10.5 | Villages and farmlands | Good |
| 668 | Guilin | Lingchuan County | Lauraceae | *Camphora officinarum Nees.* | 650 | 16.0 | 248.4 | 11.0 | Villages and farmlands | Good |
| 669 | Guilin | Lingchuan County | Lauraceae | *Camphora officinarum Nees.* | 500 | 26.0 | 198.1 | 25.0 | Villages and farmlands | Good |
| 670 | Guilin | Lingchuan County | Lauraceae | *Camphora officinarum Nees.* | 550 | 24.0 | 163.4 | 18.5 | Villages and farmlands | Good |
| 671 | Guilin | Lingchuan County | Lauraceae | *Camphora officinarum Nees.* | 500 | 31.0 | 183.1 | 23.5 | Residential districts | Good |
| 672 | Guilin | Lingchuan County | Lauraceae | *Camphora officinarum Nees.* | 560 | 17.0 | 238.9 | 14.5 | Villages and farmlands | Good |
| 673 | Guilin | Lingchuan County | Moraceae | *Ficus concinna (*Miq.) Miq. | 500 | 20.0 | 250.3 | 30.0 | Villages and farmlands | Good |
| 674 | Guilin | Lingchuan County | Moraceae | *Ficus concinna(M*iq.) Miq. | 600 | 25.0 | 265.6 | 29.0 | Villages and farmlands | Good |
| 675 | Guilin | Lingchuan County | Lauraceae | *Camphora officinarum Nees.* | 500 | 24.0 | 248.4 | 37.0 | Villages and farmlands | Good |
| 676 | Guilin | Lingchuan County | Lauraceae | *Camphora officinarum Nees.* | 520 | 10.0 | 229.9 | 14.8 | Villages and farmlands | Good |
| 677 | Guilin | Lingchuan County | Lauraceae | *Camphora officinarum Nees.* | 500 | 34.0 | 213.0 | 26.5 | Villages and farmlands | Good |
| 678 | Guilin | Lingchuan County | Lauraceae | *Camphora officinarum Nees.* | 500 | 31.0 | 229.9 | 24.5 | Villages and farmlands | Good |
| 679 | Guilin | Lingchuan County | Lauraceae | *Camphora officinarum Nees.* | 500 | 29.0 | 200.0 | 27.0 | Villages and farmlands | Good |
| 680 | Guilin | Lingchuan County | Lauraceae | *Camphora officinarum Nees.* | 800 | 36.0 | 274.8 | 30.0 | Villages and farmlands | Good |
| 681 | Guilin | Lingchuan County | Lauraceae | *Camphora officinarum Nees.* | 500 | 39.0 | 219.8 | 41.5 | Villages and farmlands | Good |
| 682 | Guilin | Lingchuan County | Lauraceae | *Camphora officinarum Nees.* | 600 | 26.0 | 197.4 | 29.0 | Villages and farmlands | Poor |
| 683 | Guilin | Lingchuan County | Lauraceae | *Camphora officinarum Nees.* | 500 | 28.0 | 194.3 | 31.0 | Villages and farmlands | Poor |
| 684 | Guilin | Lingchuan County | Fagaceae | *Castanea henryi* (Skan) Rehder & E. H. Wilson | 500 | 16.0 | 138.2 | 20.0 | Villages and farmlands | Good |
| 685 | Guilin | Lingchuan County | Lauraceae | *Camphora officinarum Nees.* | 550 | 22.0 | 216.6 | 26.5 | Villages and farmlands | Good |
| 686 | Guilin | Lingchuan County | Lauraceae | *Camphora officinarum Nees.* | 550 | 38.0 | 233.1 | 32.0 | Villages and farmlands | Good |
| 687 | Guilin | Lingchuan County | Oleaceae | *Osmanthus fragrans* (Thunb.) Lour. | 500 | 14.0 | 92.4 | 20.0 | Villages and farmlands | Good |
| 688 | Guilin | Lingchuan County | Phyllanthaceae | *Bischofia polycarpa* (H. Lév.) Airy Shaw | 800 | 28.0 | 272.6 | 37.0 | Villages and farmlands | Good |
| 689 | Guilin | Lingchuan County | Lauraceae | *Camphora officinarum Nees.* | 500 | 20.0 | 210.2 | 12.5 | Villages and farmlands | Good |
| 690 | Guilin | Lingchuan County | Lauraceae | *Camphora officinarum Nees.* | 500 | 16.0 | 213.4 | 12.0 | Villages and farmlands | Good |
| 691 | Guilin | Lingchuan County | Lauraceae | *Camphora officinarum Nees.* | 500 | 16.0 | 207.0 | 19.0 | Villages and farmlands | Good |
| 692 | Guilin | Lingchuan County | Fagaceae | *Castanopsis fargesii* Franch. | 550 | 20.0 | 74.8 | 12.0 | Villages and farmlands | Good |
| 693 | Guilin | Lingchuan County | Ginkgoaceae | *Ginkgo biloba* L. | 600 | 24.0 | 140.1 | 19.0 | Villages and farmlands | Good |
| 694 | Guilin | Lingchuan County | Ginkgoaceae | *Ginkgo biloba* L. | 500 | 16.0 | 165.6 | 12.5 | Villages and farmlands | Good |
| 695 | Guilin | Lingchuan County | Ginkgoaceae | *Ginkgo biloba* L. | 500 | 20.0 | 181.5 | 14.0 | Villages and farmlands | Good |
| 696 | Guilin | Lingchuan County | Ginkgoaceae | *Ginkgo biloba* L. | 500 | 20.0 | 191.1 | 11.0 | Villages and farmlands | Good |
| 697 | Guilin | Lingchuan County | Ginkgoaceae | *Ginkgo biloba* L. | 500 | 24.0 | 168.8 | 10.5 | Villages and farmlands | Good |
| 698 | Guilin | Lingchuan County | Ginkgoaceae | *Ginkgo biloba* L. | 500 | 15.0 | 172.0 | 7.5 | Villages and farmlands | Good |
| 699 | Guilin | Lingchuan County | Lauraceae | *Phoebe bournei (*Hemsl.) Yen C. Yang | 500 | 46.0 | 121.0 | 26.0 | Villages and farmlands | Good |
| 700 | Guilin | Lingchuan County | Lauraceae | *Camphora officinarum Nees.* | 500 | 26.0 | 170.4 | 31.0 | Villages and farmlands | Good |
| 701 | Guilin | Lingchuan County | Fagaceae | *Castanopsis fargesii* Franch. | 550 | 18.0 | 78.3 | 16.0 | Villages and farmlands | Good |
| 702 | Guilin | Lingchuan County | Fagaceae | *Castanopsis carlesii* (Hemsl.) Hayata | 600 | 26.0 | 163.4 | 20.5 | Villages and farmlands | Good |
| 703 | Guilin | Lingchuan County | Fagaceae | *Castanopsis fargesii* Franch. | 600 | 22.0 | 98.4 | 19.5 | Villages and farmlands | Good |
| 704 | Guilin | Quanzhou County | Lauraceae | *Camphora officinarum Nees.* | 650 | 28.0 | 260.0 | 28.0 | Residential districts | Good |
| 705 | Guilin | Quanzhou County | Lauraceae | *Camphora officinarum Nees.* | 600 | 30.0 | 248.0 | 26.0 | Villages and farmlands | Good |
| 706 | Guilin | Quanzhou County | Lauraceae | *Camphora officinarum Nees.* | 500 | 27.0 | 215.0 | 47.5 | Villages and farmlands | Good |
| 707 | Guilin | Quanzhou County | Lauraceae | *Camphora officinarum Nees.* | 500 | 25.0 | 234.0 | 39.0 | Villages and farmlands | Good |
| 708 | Guilin | Quanzhou County | Cupressaceae | *Cupressus funebris* Endl. | 520 | 25.0 | 159.2 | 11.0 | Villages and farmlands | Good |
| 709 | Guilin | Quanzhou County | Lauraceae | *Camphora officinarum Nees* | 600 | 30.0 | 256.6 | 29.0 | Villages and farmlands | Good |
| 710 | Guilin | Quanzhou County | Taxaceae | *Taxus wallichiana* var. *mairei* (Lemée & H. Lév.) L. K. Fu & Nan Li | 950 | 25.0 | 146.0 | 20.0 | Villages and farmlands | Good |
| 711 | Guilin | Quanzhou County | Lauraceae | *Camphora officinarum Nees.* | 510 | 28.0 | 222.0 | 27.0 | Villages and farmlands | Good |
| 712 | Guilin | Quanzhou County | Lauraceae | *Camphora officinarum Nees.* | 500 | 13.0 | 221.3 | 8.0 | Villages and farmlands | Good |
| 713 | Guilin | Quanzhou County | Lauraceae | *Camphora officinarum Nees.* | 700 | 28.0 | 274.0 | 43.0 | Villages and farmlands | Good |
| 714 | Guilin | Quanzhou County | Lauraceae | *Camphora officinarum Nees.* | 500 | 25.0 | 222.9 | 28.0 | Villages and farmlands | Good |
| 715 | Guilin | Quanzhou County | Phyllanthaceae | *Bischofia polycarpa* (H. Lév.) Airy Shaw | 500 | 20.0 | 190.3 | 19.5 | Villages and farmlands | Good |
| 716 | Guilin | Quanzhou County | Lauraceae | *Camphora officinarum Nees* | 500 | 22.0 | 220.9 | 28.5 | Villages and farmlands | Good |
| 717 | Guilin | Quanzhou County | Fabaceae | *Styphnolobium japonicum* (L.) Schott | 540 | 20.0 | 146.5 | 16.5 | Villages and farmlands | Good |
| 718 | Guilin | Quanzhou County | Lauraceae | *Camphora officinarum Nees.* | 600 | 17.0 | 257.9 | 21.0 | Villages and farmlands | Good |
| 719 | Guilin | Quanzhou County | Oleaceae | *Osmanthus fragrans* (Thunb.) Lour. | 720 | 12.0 | 96.5 | 13.0 | Villages and farmlands | Good |
| 720 | Guilin | Quanzhou County | Fagaceae | *Quercus acutissima* Carruth. | 510 | 18.0 | 105.1 | 16.5 | Villages and farmlands | Good |
| 721 | Guilin | Quanzhou County | Lauraceae | *Camphora officinarum Nees* | 500 | 22.0 | 220.7 | 28.5 | Villages and farmlands | Good |
| 722 | Guilin | Quanzhou County | Hamamelidaceae | *Loropetalum chinense* (R. Br.) Oliv. | 500 | 10.0 | 44.6 | 8.5 | Villages and farmlands | Moderate |
| 723 | Guilin | Quanzhou County | Lauraceae | *Camphora officinarum Nees.* | 860 | 28.0 | 305.0 | 21.5 | Villages and farmlands | Good |
| 724 | Guilin | Quanzhou County | Lauraceae | *Camphora officinarum Nees.* | 500 | 32.0 | 221.0 | 27.0 | Villages and farmlands | Good |
| 725 | Guilin | Quanzhou County | Lauraceae | *Camphora officinarum Nees.* | 620 | 22.0 | 255.0 | 19.0 | Villages and farmlands | Good |
| 726 | Guilin | Quanzhou County | Lauraceae | *Camphora officinarum Nees.* | 700 | 25.0 | 262.0 | 27.0 | Villages and farmlands | Good |
| 727 | Guilin | Quanzhou County | Lauraceae | *Camphora officinarum Nees.* | 550 | 26.0 | 237.0 | 22.5 | Villages and farmlands | Good |
| 728 | Guilin | Quanzhou County | Lauraceae | *Camphora officinarum Nees.* | 600 | 25.0 | 252.0 | 29.0 | Villages and farmlands | Good |
| 729 | Guilin | Quanzhou County | Lauraceae | *Camphora officinarum Nees.* | 600 | 18.0 | 248.0 | 19.0 | Villages and farmlands | Good |
| 730 | Guilin | Quanzhou County | Lauraceae | *Camphora officinarum Nees.* | 800 | 22.0 | 287.0 | 17.0 | Villages and farmlands | Good |
| 731 | Guilin | Quanzhou County | Anacardiaceae | *Camphora officinarum Nees.* | 550 | 25.0 | 197.5 | 18.0 | Villages and farmlands | Good |
| 732 | Guilin | Quanzhou County | Lauraceae | *Camphora officinarum Nees.* | 700 | 18.0 | 267.5 | 19.0 | Villages and farmlands | Good |
| 733 | Guilin | Quanzhou County | Lauraceae | *Camphora officinarum Nees.* | 550 | 22.0 | 238.0 | 39.0 | Villages and farmlands | Good |
| 734 | Guilin | Quanzhou County | Lauraceae | *Camphora officinarum Nees.* | 550 | 27.0 | 235.0 | 42.0 | Villages and farmlands | Good |
| 735 | Guilin | Quanzhou County | Lauraceae | *Camphora officinarum Nees.* | 500 | 33.0 | 228.0 | 40.0 | Villages and farmlands | Good |
| 736 | Guilin | Quanzhou County | Lauraceae | *Camphora officinarum Nees.* | 550 | 22.0 | 236.0 | 38.0 | Villages and farmlands | Good |
| 737 | Guilin | Quanzhou County | Ginkgoaceae | *Ginkgo biloba* L. | 580 | 26.0 | 240.0 | 25.0 | Villages and farmlands | Good |
| 738 | Guilin | Xing'an County | Phyllanthaceae | *Bischofia polycarpa* (H. Lév.) Airy Shaw | 700 | 22.0 | 136.9 | 26.0 | Residential districts | Poor |
| 739 | Guilin | Xing'an County | Lauraceae | *Camphora officinarum Nees* | 520 | 24.0 | 199.0 | 13.0 | Villages and farmlands | Poor |
| 740 | Guilin | Xing'an County | Oleaceae | *Osmanthus fragrans* (Thunb.) Lour. | 500 | 18.0 | 90.0 | 25.0 | Villages and farmlands | Good |
| 741 | Guilin | Xing'an County | Lauraceae | *Camphora officinarum Nees.* | 600 | 26.0 | 242.0 | 31.0 | Villages and farmlands | Good |
| 742 | Guilin | Xing'an County | Lauraceae | *Camphora officinarum Nees.* | 500 | 20.0 | 219.0 | 20.0 | Villages and farmlands | Good |
| 743 | Guilin | Xing'an County | Lauraceae | *Camphora officinarum Nees.* | 500 | 28.0 | 203.0 | 21.5 | Villages and farmlands | Good |
| 744 | Guilin | Xing'an County | Lauraceae | *Camphora officinarum Nees.* | 500 | 30.0 | 232.0 | 20.5 | Villages and farmlands | Good |
| 745 | Guilin | Xing'an County | Lauraceae | *Camphora officinarum Nees.* | 500 | 28.0 | 203.0 | 27.0 | Villages and farmlands | Good |
| 746 | Guilin | Xing'an County | Lauraceae | *Camphora officinarum Nees.* | 800 | 30.0 | 226.0 | 26.5 | Villages and farmlands | Good |
| 747 | Guilin | Xing'an County | Lauraceae | *Camphora officinarum Nees.* | 510 | 16.0 | 200.0 | 11.5 | Villages and farmlands | Good |
| 748 | Guilin | Xing'an County | Lauraceae | *Camphora officinarum Nees.* | 500 | 19.0 | 200.0 | 31.5 | Villages and farmlands | Good |
| 749 | Guilin | Xing'an County | Lauraceae | *Camphora officinarum Nees.* | 600 | 25.0 | 167.0 | 26.0 | Villages and farmlands | Good |
| 750 | Guilin | Xing'an County | Lauraceae | *Camphora officinarum Nees.* | 600 | 24.0 | 156.0 | 20.5 | Villages and farmlands | Good |
| 751 | Guilin | Xing'an County | Lauraceae | *Camphora officinarum Nees.* | 500 | 16.0 | 237.0 | 22.5 | Villages and farmlands | Good |
| 752 | Guilin | Xing'an County | Lauraceae | *Camphora officinarum Nees.* | 500 | 26.0 | 220.0 | 9.0 | Villages and farmlands | Good |
| 753 | Guilin | Xing'an County | Fagaceae | *Quercus acutissima* Carruth. | 500 | 22.0 | 155.0 | 13.5 | Villages and farmlands | Good |
| 754 | Guilin | Xing'an County | Theaceae | *Schima superba* Gardner & Champ. | 500 | 20.0 | 148.0 | 15.0 | Villages and farmlands | Good |
| 755 | Guilin | Xing'an County | Taxaceae | *Taxus wallichiana* var. *mairei* (Lemée & H. Lév.) L. K. Fu & Nan Li | 500 | 12.0 | 100.0 | 5.9 | Villages and farmlands | Good |
| 756 | Guilin | Xing'an County | Oleaceae | *Osmanthus fragrans* (Thunb.) Lour. | 500 | 8.0 | 115.0 | 16.5 | Villages and farmlands | Good |
| 757 | Guilin | Xing'an County | Moraceae | *Morus cathayana* Hemsl. | 500 | 14.0 | 115.0 | 8.0 | Villages and farmlands | Good |
| 758 | Guilin | Xing'an County | Lauraceae | *Camphora officinarum Nees.* | 500 | 11.0 | 210.0 | 12.0 | Villages and farmlands | Good |
| 759 | Guilin | Xing'an County | Fagaceae | *Quercus acutissima* Carruth. | 800 | 14.0 | 126.0 | 8.0 | Villages and farmlands | Good |
| 760 | Guilin | Xing'an County | Pinaceae | *Tsuga chinensis* (Franch.) E. Pritz. | 550 | 11.0 | 57.6 | 9.5 | Wooded areas and plant nurseries | Good |
| 761 | Guilin | Xing'an County | Pinaceae | *Tsuga chinensis* (Franch.) E. Pritz. | 850 | 13.0 | 64.6 | 10.0 | Wooded areas and plant nurseries | Good |
| 762 | Guilin | Xing'an County | Pinaceae | *Tsuga chinensis* (Franch.) E. Pritz. | 900 | 13.0 | 68.4 | 8.0 | Wooded areas and plant nurseries | Good |
| 763 | Guilin | Xing'an County | Pinaceae | *Tsuga chinensis* (Franch.) E. Pritz. | 500 | 13.0 | 55.0 | 8.8 | Wooded areas and plant nurseries | Good |
| 764 | Guilin | Xing'an County | Pinaceae | *Tsuga chinensis* (Franch.) E. Pritz. | 900 | 12.0 | 73.0 | 10.0 | Wooded areas and plant nurseries | Good |
| 765 | Guilin | Xing'an County | Pinaceae | *Tsuga chinensis* (Franch.) E. Pritz. | 900 | 12.0 | 62.0 | 12.0 | Wooded areas and plant nurseries | Good |
| 766 | Guilin | Xing'an County | Pinaceae | *Tsuga chinensis* (Franch.) E. Pritz. | 550 | 13.0 | 57.0 | 11.0 | Wooded areas and plant nurseries | Good |
| 767 | Guilin | Yongfu County | Lauraceae | *Camphora officinarum Nees.* | 520 | 33.0 | 223.0 | 36.0 | Villages and farmlands | Good |
| 768 | Guilin | Yongfu County | Lauraceae | *Camphora officinarum Nees.* | 590 | 36.0 | 246.0 | 24.0 | Villages and farmlands | Good |
| 769 | Guilin | Yongfu County | Fagaceae | *Castanopsis hystrix* Hook. f. & Thomson ex A. DC. | 550 | 26.0 | 146.0 | 27.0 | Villages and farmlands | Good |
| 770 | Guilin | Yongfu County | Fagaceae | *Castanopsis carlesii* (Hemsl.) Hayata | 500 | 24.0 | 137.0 | 9.5 | Villages and farmlands | Poor |
| 771 | Guilin | Yongfu County | Lauraceae | *Camphora officinarum Nees.* | 700 | 31.0 | 274.0 | 15.5 | Villages and farmlands | Poor |
| 772 | Guilin | Yongfu County | Fagaceae | *Castanopsis sclerophylla* (Lindl.) Schottky | 600 | 30.0 | 165.0 | 20.0 | Villages and farmlands | Good |
| 773 | Guilin | Yongfu County | Lauraceae | *Camphora officinarum Nees.* | 600 | 32.0 | 258.0 | 19.0 | Villages and farmlands | Good |
| 774 | Guilin | Yongfu County | Lauraceae | *Camphora officinarum Nees.* | 550 | 21.0 | 245.0 | 29.5 | Villages and farmlands | Good |
| 775 | Guilin | Yongfu County | Fagaceae | *Castanopsis chinensis* (Spreng.) Hance | 720 | 24.0 | 178.0 | 19.0 | Villages and farmlands | Good |
| 776 | Guilin | Yongfu County | Fagaceae | *Castanopsis chinensis* (Spreng.) Hance | 600 | 26.0 | 160.0 | 13.5 | Villages and farmlands | Good |
| 777 | Guilin | Yongfu County | Lauraceae | *Camphora officinarum Nees.* | 530 | 27.0 | 232.0 | 23.0 | Residential districts | Good |
| 778 | Guilin | Yongfu County | Lauraceae | *Camphora officinarum Nees.* | 505 | 30.0 | 222.0 | 33.0 | Villages and farmlands | Good |
| 779 | Guilin | Yongfu County | Lauraceae | *Camphora officinarum Nees.* | 500 | 15.0 | 226.0 | 9.6 | Villages and farmlands | Good |
| 780 | Guilin | Yongfu County | Lauraceae | *Camphora officinarum Nees.* | 550 | 22.0 | 237.0 | 30.0 | Villages and farmlands | Good |
| 781 | Guilin | Yongfu County | Lauraceae | *Camphora officinarum Nees.* | 560 | 30.0 | 240.0 | 29.0 | Villages and farmlands | Good |
| 782 | Guilin | Yongfu County | Fagaceae | *Castanopsis hystrix* Hook. f. & Thomson ex A. DC. | 650 | 30.6 | 168.7 | 25.4 | Villages and farmlands | Good |
| 783 | Guilin | Yongfu County | Fagaceae | *Castanopsis chinensis* (Spreng.) Hance | 520 | 30.0 | 138.2 | 10.0 | Villages and farmlands | Good |
| 784 | Guilin | Yongfu County | Fagaceae | *Castanopsis chinensis* (Spreng.) Hance | 580 | 30.0 | 152.9 | 15.0 | Villages and farmlands | Good |
| 785 | Guilin | Yongfu County | Theaceae | *Schima superba* Gardner & Champ. | 500 | 25.0 | 121.0 | 15.0 | Villages and farmlands | Good |
| 786 | Guilin | Yongfu County | Fagaceae | *Castanopsis chinensis* (Spreng.) Hance | 800 | 30.0 | 181.5 | 20.0 | Villages and farmlands | Good |
| 787 | Guilin | Yongfu County | Fagaceae | *Castanopsis chinensis*(Spreng.) Hance | 500 | 25.0 | 137.1 | 20.0 | Villages and farmlands | Good |
| 788 | Guilin | Yongfu County | Fagaceae | *Castanopsis hystrix* Hook. f. & Thomson ex A. DC. | 630 | 20.0 | 159.2 | 10.0 | Villages and farmlands | Good |
| 789 | Guilin | Guanyang County | Lauraceae | *Camphora officinarum Nees.* | 550 | 35.0 | 241.0 | 28.0 | Villages and farmlands | Good |
| 790 | Guilin | Guanyang County | Lauraceae | *Camphora officinarum Nees.* | 550 | 25.0 | 230.0 | 30.0 | Villages and farmlands | Good |
| 791 | Guilin | Guanyang County | Lauraceae | *Camphora officinarum Nees.* | 600 | 22.0 | 248.0 | 23.0 | Villages and farmlands | Good |
| 792 | Guilin | Guanyang County | Lauraceae | *Camphora officinarum Nees.* | 650 | 24.0 | 258.0 | 28.4 | Villages and farmlands | Good |
| 793 | Guilin | Guanyang County | Lauraceae | *Camphora officinarum Nees.* | 570 | 20.0 | 239.0 | 24.7 | Villages and farmlands | Good |
| 794 | Guilin | Guanyang County | Lauraceae | *Camphora officinarum Nees.* | 550 | 20.0 | 238.0 | 30.0 | Villages and farmlands | Good |
| 795 | Guilin | Guanyang County | Lauraceae | *Camphora officinarum Nees.* | 510 | 19.0 | 223.0 | 22.5 | Villages and farmlands | Good |
| 796 | Guilin | Guanyang County | Fabaceae | *Platyosprion platycarpum* (Maxim.) Maxim. | 550 | 24.0 | 151.0 | 24.5 | Villages and farmlands | Moderate |
| 797 | Guilin | Guanyang County | Lauraceae | *Camphora officinarum Nees.* | 500 | 12.0 | 223.0 | 11.5 | Villages and farmlands | Good |
| 798 | Guilin | Guanyang County | Lauraceae | *Camphora officinarum Nees.* | 550 | 25.0 | 235.0 | 24.0 | Villages and farmlands | Good |
| 799 | Guilin | Guanyang County | Lauraceae | *Camphora officinarum Nees.* | 550 | 24.0 | 240.0 | 19.0 | Villages and farmlands | Good |
| 800 | Guilin | Guanyang County | Lauraceae | *Camphora officinarum Nees.* | 500 | 18.0 | 230.0 | 25.0 | Villages and farmlands | Good |
| 801 | Guilin | Guanyang County | Lauraceae | *Camphora officinarum Nees.* | 500 | 30.0 | 206.0 | 46.0 | Villages and farmlands | Good |
| 802 | Guilin | Guanyang County | Lauraceae | *Camphora officinarum Nees.* | 550 | 25.0 | 224.0 | 26.0 | Villages and farmlands | Good |
| 803 | Guilin | Guanyang County | Podocarpaceae | *Podocarpus macrophyllus* (Thunb.) Sweet | 800 | 9.0 | 112.0 | 7.2 | Villages and farmlands | Poor |
| 804 | Guilin | Guanyang County | Taxaceae | *Taxus wallichiana* var. *mairei* (Lemée & H. Lév.) L. K. Fu & Nan Li | 600 | 23.0 | 115.0 | 19.1 | Villages and farmlands | Good |
| 805 | Guilin | Guanyang County | Taxaceae | *Taxus wallichiana* var. *mairei* (Lemée & H. Lév.) L. K. Fu & Nan Li | 550 | 19.0 | 110.0 | 15.1 | Villages and farmlands | Good |
| 806 | Guilin | Guanyang County | Taxaceae | *Taxus wallichiana* var. *mairei* (Lemée & H. Lév.) L. K. Fu & Nan Li | 550 | 19.0 | 105.0 | 12.4 | Villages and farmlands | Good |
| 807 | Guilin | Guanyang County | Taxaceae | *Taxus wallichiana* var. *mairei* (Lemée & H. Lév.) L. K. Fu & Nan Li | 540 | 14.0 | 108.0 | 13.8 | Villages and farmlands | Good |
| 808 | Guilin | Guanyang County | Taxaceae | *Taxus wallichiana* var. *mairei* (Lemée & H. Lév.) L. K. Fu & Nan Li | 600 | 18.0 | 116.0 | 16.0 | Villages and farmlands | Good |
| 809 | Guilin | Guanyang County | Taxaceae | *Taxus wallichiana* var. *mairei* (Lemée & H. Lév.) L. K. Fu & Nan Li | 750 | 15.0 | 130.0 | 12.0 | Villages and farmlands | Good |
| 810 | Guilin | Guanyang County | Taxaceae | *Taxus wallichiana* var. *mairei* (Lemée & H. Lév.) L. K. Fu & Nan Li | 500 | 20.0 | 105.0 | 14.1 | Villages and farmlands | Good |
| 811 | Guilin | Guanyang County | Taxaceae | *Taxus wallichiana* var. *mairei* (Lemée & H. Lév.) L. K. Fu & Nan Li | 700 | 27.5 | 124.0 | 13.6 | Villages and farmlands | Good |
| 812 | Guilin | Guanyang County | Ginkgoaceae | *Ginkgo biloba* L. | 500 | 25.0 | 121.0 | 17.4 | Villages and farmlands | Good |
| 813 | Guilin | Guanyang County | Ginkgoaceae | *Ginkgo biloba* L. | 500 | 30.0 | 136.0 | 20.5 | Villages and farmlands | Good |
| 814 | Guilin | Guanyang County | Ginkgoaceae | *Ginkgo biloba* L. | 600 | 32.0 | 175.0 | 25.5 | Villages and farmlands | Good |
| 815 | Guilin | Guanyang County | Lauraceae | *Camphora officinarum Nees.* | 500 | 24.0 | 224.0 | 21.0 | Residential districts | Good |
| 816 | Guilin | Guanyang County | Lauraceae | *Camphora officinarum Nees.* | 550 | 32.0 | 231.0 | 22.0 | Villages and farmlands | Good |
| 817 | Guilin | Guanyang County | Lauraceae | *Camphora officinarum Nees.* | 550 | 30.0 | 240.0 | 27.0 | Villages and farmlands | Good |
| 818 | Guilin | Guanyang County | Pinaceae | *Nothotsuga longibracteata* (W. C. Cheng) Hu ex C. N. Page | 520 | 18.0 | 55.6 | 9.0 | Wooded areas and plant nurseries | Poor |
| 819 | Guilin | Guanyang County | Pinaceae | *Nothotsuga longibracteata* (W. C. Cheng) Hu ex C. N. Page | 800 | 16.0 | 64.0 | 9.5 | Wooded areas and plant nurseries | Moderate |
| 820 | Guilin | Guanyang County | Pinaceae | *Nothotsuga longibracteata* (W. C. Cheng) Hu ex C. N. Page | 550 | 21.0 | 57.1 | 8.5 | Wooded areas and plant nurseries | Poor |
| 821 | Guilin | Guanyang County | Pinaceae | *Nothotsuga longibracteata* (W. C. Cheng) Hu ex C. N. Page | 600 | 15.0 | 58.4 | 9.0 | Wooded areas and plant nurseries | Poor |
| 822 | Guilin | Guanyang County | Pinaceae | *Nothotsuga longibracteata* (W. C. Cheng) Hu ex C. N. Page | 650 | 19.0 | 59.8 | 12.0 | Wooded areas and plant nurseries | Good |
| 823 | Guilin | Guanyang County | Magnoliaceae | *Liriodendron chinense* (Hemsl.) Sarg. | 500 | 26.0 | 87.7 | 18.0 | Wooded areas and plant nurseries | Good |
| 824 | Guilin | Guanyang County | Pinaceae | *Nothotsuga longibracteata* (W. C. Cheng) Hu ex C. N. Page | 800 | 22.0 | 63.0 | 6.5 | Wooded areas and plant nurseries | Moderate |
| 825 | Guilin | Guanyang County | Pinaceae | *Nothotsuga longibracteata* (W. C. Cheng) Hu ex C. N. Page | 550 | 18.0 | 57.0 | 13.5 | Wooded areas and plant nurseries | Moderate |
| 826 | Guilin | Guanyang County | Pinaceae | *Nothotsuga longibracteata* (W. C. Cheng) Hu ex C. N. Page | 850 | 18.0 | 64.0 | 10.0 | Wooded areas and plant nurseries | Moderate |
| 827 | Guilin | Longsheng County | Lauraceae | *Camphora officinarum Nees.* | 800 | 30.0 | 250.0 | 32.0 | Residential districts | Moderate |
| 828 | Guilin | Longsheng County | Lauraceae | *Camphora officinarum Nees.* | 750 | 25.0 | 230.0 | 43.0 | Residential districts | Moderate |
| 829 | Guilin | Longsheng County | Lauraceae | *Camphora officinarum Nees.* | 800 | 22.0 | 255.0 | 35.5 | Residential districts | Moderate |
| 830 | Guilin | Longsheng County | Fagaceae | *Castanopsis hystrix* Hook. f. & Thomson ex A. DC. | 600 | 22.0 | 120.0 | 11.0 | Residential districts | Good |
| 831 | Guilin | Longsheng County | Fagaceae | *Castanopsis hystrix* Hook. f. & Thomson ex A. DC. | 700 | 24.0 | 162.0 | 23.0 | Villages and farmlands | Good |
| 832 | Guilin | Longsheng County | Fagaceae | *Castanopsis chinensis* (Spreng.) Hance | 900 | 21.0 | 202.0 | 19.0 | Villages and farmlands | Good |
| 833 | Guilin | Longsheng County | Lauraceae | *Machilus leptophylla* Hand.-Mazz. | 550 | 23.0 | 136.0 | 17.5 | Villages and farmlands | Good |
| 834 | Guilin | Longsheng County | Taxaceae | *Taxus wallichiana* var. *mairei* (Lemée & H. Lév.) L. K. Fu & Nan Li | 500 | 23.0 | 116.0 | 11.0 | Villages and farmlands | Good |
| 835 | Guilin | Longsheng County | Taxaceae | *Taxus wallichiana* var. *mairei* (Lemée & H. Lév.) L. K. Fu & Nan Li | 900 | 22.0 | 117.0 | 14.0 | Villages and farmlands | Good |
| 836 | Guilin | Longsheng County | Meliaceae | *Toona ciliata M.* Roem. | 520 | 20.0 | 194.3 | 9.0 | Villages and farmlands | Moderate |
| 837 | Guilin | Longsheng County | Lauraceae | *Camphora officinarum Nees.* | 500 | 22.0 | 210.0 | 17.0 | Villages and farmlands | Good |
| 838 | Guilin | Longsheng County | Lauraceae | *Camphora officinarum Nees.* | 500 | 24.0 | 217.0 | 12.0 | Villages and farmlands | Good |
| 839 | Guilin | Longsheng County | Lauraceae | *Camphora officinarum Nees.* | 600 | 20.0 | 221.0 | 26.5 | Villages and farmlands | Good |
| 840 | Guilin | Longsheng County | Moraceae | *Ficus microcarpa* L. f. | 500 | 26.0 | 230.0 | 41.0 | Residential districts | Good |
| 841 | Guilin | Longsheng County | Lauraceae | *Camphora officinarum Nees.* | 600 | 25.0 | 230.0 | 29.0 | Villages and farmlands | Good |
| 842 | Guilin | Longsheng County | Fagaceae | *Castanopsis tibetana* Hance. | 500 | 17.0 | 150.0 | 13.0 | Villages and farmlands | Poor |
| 843 | Guilin | Longsheng County | Fagaceae | *Castanopsis tibetana* Hance. | 550 | 15.0 | 115.0 | 13.0 | Villages and farmlands | Moderate |
| 844 | Guilin | Longsheng County | Taxaceae | *Taxus wallichiana* var. *mairei* (Lemée & H. Lév.) L. K. Fu & Nan Li | 650 | 22.0 | 120.0 | 19.0 | Villages and farmlands | Good |
| 845 | Guilin | Longsheng County | Fagaceae | *Castanopsis tibetana* Hance | 550 | 13.0 | 106.0 | 10.0 | Villages and farmlands | Good |
| 846 | Guilin | Longsheng County | Magnoliaceae | *Liriodendron chinense* (Hemsl.) Sarg. | 550 | 25.0 | 106.6 | 15.0 | Villages and farmlands | Good |
| 847 | Guilin | Longsheng County | Taxaceae | *Taxus wallichiana* var. *mairei* (Lemée & H. Lév.) L. K. Fu & Nan Li | 520 | 9.0 | 127.3 | 8.0 | Villages and farmlands | Good |
| 848 | Guilin | Longsheng County | Fagaceae | *Castanopsis hystrix* Hook. f. & Thomson ex A. DC. | 500 | 15.0 | 156.1 | 15.0 | Villages and farmlands | Good |
| 849 | Guilin | Longsheng County | Lauraceae | *Phoebe bournei (*Hemsl.) Yen C. Yang | 500 | 20.0 | 86.0 | 19.5 | Villages and farmlands | Good |
| 850 | Guilin | Longsheng County | Fagaceae | *Castanopsis hystrix* Hook. f. & Thomson ex A. DC. | 500 | 25.0 | 153.0 | 15.5 | Villages and farmlands | Good |
| 851 | Guilin | Longsheng County | Fagaceae | *Castanopsis hystrix* Hook. f. & Thomson ex A. DC. | 500 | 20.0 | 143.0 | 20.0 | Villages and farmlands | Good |
| 852 | Guilin | Longsheng County | Altingiaceae | *Liquidambar form*o*sana* Hance | 500 | 21.0 | 124.0 | 9.5 | Villages and farmlands | Good |
| 853 | Guilin | Longsheng County | Fagaceae | *Castanopsis hystrix* Hook. f. & Thomson ex A. DC. | 500 | 26.0 | 140.0 | 16.1 | Villages and farmlands | Good |
| 854 | Guilin | Longsheng County | Altingiaceae | *Liquidambar formosana* Hance. | 550 | 22.0 | 134.6 | 15.0 | Villages and farmlands | Good |
| 855 | Guilin | Longsheng County | Fagaceae | *Quercus glauca T*hunb. | 500 | 15.0 | 79.6 | 15.5 | Villages and farmlands | Good |
| 856 | Guilin | Longsheng County | Lauraceae | *Camphora officinarum Nees* | 500 | 18.0 | 207.0 | 37.3 | Villages and farmlands | Good |
| 857 | Guilin | Longsheng County | Fagaceae | *Castanopsis carlesii* (Hemsl.) Hayata | 500 | 25.0 | 54.0 | 11.0 | Wooded areas and plant nurseries | Good |
| 858 | Guilin | Longsheng County | Fagaceae | *Fagus longipetiolata* Seemen. | 500 | 26.0 | 89.1 | 16.0 | Wooded areas and plant nurseries | Good |
| 859 | Guilin | Longsheng County | Fagaceae | *Fagus longipetiolata* Seemen. | 500 | 19.0 | 76.5 | 10.0 | Wooded areas and plant nurseries | Good |
| 860 | Guilin | Longsheng County | Fagaceae | *Fagus longipetiolata* Seemen. | 500 | 18.0 | 77.6 | 10.0 | Wooded areas and plant nurseries | Good |
| 861 | Guilin | Longsheng County | Fagaceae | *Fagus longipetiolata* Seemen. | 500 | 26.0 | 93.3 | 16.5 | Wooded areas and plant nurseries | Good |
| 862 | Guilin | Longsheng County | Fagaceae | *Fagus longipetiolata* Seemen. | 500 | 20.0 | 77.5 | 20.0 | Wooded areas and plant nurseries | Good |
| 863 | Guilin | Longsheng County | Fagaceae | *Fagus longipetiolata* Seemen. | 500 | 15.0 | 88.2 | 23.5 | Wooded areas and plant nurseries | Good |
| 864 | Guilin | Longsheng County | Theaceae | *Schima superba* Gardner & Champ. | 510 | 25.0 | 109.8 | 23.5 | Wooded areas and plant nurseries | Good |
| 865 | Guilin | Longsheng County | Lauraceae | *Sassafras tzumu* (Hemsl.) Hemsl. | 500 | 19.0 | 81.5 | 11.5 | Wooded areas and plant nurseries | Good |
| 866 | Guilin | Longsheng County | Fagaceae | *Quercus glauca* Thunb. | 550 | 20.0 | 130.0 | 9.0 | Villages and farmlands | Good |
| 867 | Guilin | Longsheng County | Theaceae | *Schima superba* Gardner & Champ. | 500 | 35.0 | 150.0 | 10.0 | Villages and farmlands | Good |
| 868 | Guilin | Longsheng County | Ginkgoaceae | *Ginkgo biloba* L. | 900 | 20.0 | 150.0 | 7.0 | Villages and farmlands | Good |
| 869 | Guilin | Longsheng County | Ginkgoaceae | *Ginkgo biloba* L. | 900 | 20.0 | 150.0 | 7.0 | Villages and farmlands | Good |
| 870 | Guilin | Longsheng County | Theaceae | *Schima superba* Gardner & Champ. | 500 | 35.0 | 150.0 | 10.0 | Villages and farmlands | Good |
| 871 | Guilin | Longsheng County | Fagaceae | *Castanopsis chinensis* (Spreng.) Hance | 500 | 19.0 | 140.0 | 7.0 | Villages and farmlands | Good |
| 872 | Guilin | Longsheng County | Fagaceae | *Castanopsis chinensis* (Spreng.) Hance | 500 | 19.0 | 148.0 | 7.0 | Villages and farmlands | Good |
| 873 | Guilin | Longsheng County | Fagaceae | *Castanopsis chinensis* (Spreng.) Hance | 500 | 19.0 | 150.0 | 7.0 | Villages and farmlands | Good |
| 874 | Guilin | Longsheng County | Altingiaceae | *Liquidambar form*o*sana* Hance | 500 | 27.0 | 92.0 | 10.0 | Villages and farmlands | Good |
| 875 | Guilin | Longsheng County | Pinaceae | *Pinus kwangtungensis* Chun ex Tsiang | 500 | 20.0 | 80.0 | 9.0 | Villages and farmlands | Good |
| 876 | Guilin | Longsheng County | Pinaceae | *Pinus kwangtungensis* Chun ex Tsiang | 500 | 12.0 | 81.0 | 9.5 | Villages and farmlands | Good |
| 877 | Guilin | Longsheng County | Pinaceae | *Pinus kwangtungensis* Chun ex Tsiang | 500 | 10.0 | 80.0 | 8.5 | Villages and farmlands | Good |
| 878 | Guilin | Longsheng County | Pinaceae | *Pinus kwangtungensis* Chun ex Tsiang | 520 | 12.0 | 80.0 | 9.0 | Villages and farmlands | Good |
| 879 | Guilin | Longsheng County | Pinaceae | *Pinus kwangtungensis* Chun ex Tsiang | 500 | 10.0 | 80.0 | 19.0 | Villages and farmlands | Good |
| 880 | Guilin | Longsheng County | Pinaceae | *Pinus kwangtungensis* Chun ex Tsiang | 500 | 15.0 | 83.0 | 15.0 | Villages and farmlands | Good |
| 881 | Guilin | Longsheng County | Taxaceae | *Taxus wallichiana* var. *mairei* (Lemée & H. Lév.) L. K. Fu & Nan Li | 650 | 20.0 | 115.0 | 21.0 | Villages and farmlands | Moderate |
| 882 | Guilin | Longsheng County | Taxaceae | *Taxus wallichiana* var. *mairei* (Lemée & H. Lév.) L. K. Fu & Nan Li | 500 | 27.0 | 105.0 | 11.0 | Villages and farmlands | Moderate |
| 883 | Guilin | Longsheng County | Taxaceae | *Taxus wallichiana* var. *mairei* (Lemée & H. Lév.) L. K. Fu & Nan Li | 550 | 18.0 | 110.0 | 16.5 | Villages and farmlands | Moderate |
| 884 | Guilin | Longsheng County | Taxaceae | *Taxus wallichiana* var. *mairei* (Lemée & H. Lév.) L. K. Fu & Nan Li | 500 | 30.0 | 105.0 | 11.5 | Villages and farmlands | Moderate |
| 885 | Guilin | Longsheng County | Taxaceae | *Taxus wallichiana* var. *mairei* (Lemée & H. Lév.) L. K. Fu & Nan Li | 600 | 30.0 | 117.0 | 9.5 | Villages and farmlands | Moderate |
| 886 | Guilin | Longsheng County | Fagaceae | *Castanea henryi* (Skan) Rehder & E. H. Wilson | 500 | 20.0 | 180.0 | 18.0 | Villages and farmlands | Moderate |
| 887 | Guilin | Longsheng County | Fagaceae | *Castanea henryi* (Skan) Rehder & E. H. Wilson | 500 | 22.0 | 180.0 | 13.0 | Villages and farmlands | Good |
| 888 | Guilin | Longsheng County | Fagaceae | *Castanea henryi* (Skan) Rehder & E. H. Wilson | 500 | 25.0 | 160.0 | 11.0 | Villages and farmlands | Poor |
| 889 | Guilin | Longsheng County | Fagaceae | *Castanea henryi* (Skan) Rehder & E. H. Wilson | 500 | 26.0 | 130.0 | 10.5 | Villages and farmlands | Good |
| 890 | Guilin | Longsheng County | Fagaceae | *Castanea henryi* (Skan) Rehder & E. H. Wilson | 500 | 15.0 | 130.0 | 13.5 | Villages and farmlands | Good |
| 891 | Guilin | Longsheng County | Fagaceae | *Castanea henryi* (Skan) Rehder & E. H. Wilson | 500 | 30.0 | 156.0 | 10.0 | Villages and farmlands | Good |
| 892 | Guilin | Longsheng County | Fagaceae | *Castanea henryi* (Skan) Rehder & E. H. Wilson | 500 | 20.0 | 207.0 | 11.0 | Villages and farmlands | Good |
| 893 | Guilin | Longsheng County | Taxaceae | *Taxus wallichiana* var. *mairei* (Lemée & H. Lév.) L. K. Fu & Nan Li | 500 | 30.0 | 96.0 | 19.0 | Villages and farmlands | Good |
| 894 | Guilin | Longsheng County | Taxaceae | *Taxus wallichiana* var. *mairei* (Lemée & H. Lév.) L. K. Fu & Nan Li | 500 | 14.0 | 159.0 | 10.5 | Villages and farmlands | Good |
| 895 | Guilin | Longsheng County | Cupressaceae | *Cunninghamia lanceolata* (Lamb.) Hook. | 500 | 32.0 | 140.0 | 15.5 | Villages and farmlands | Good |
| 896 | Guilin | Longsheng County | Cupressaceae | *Cunninghamia lanceolata* (Lamb.) Hook. | 500 | 25.0 | 136.0 | 6.5 | Villages and farmlands | Good |
| 897 | Guilin | Longsheng County | Fagaceae | *Castanea henryi* (Skan) Rehder & E. H. Wilson | 500 | 45.0 | 130.0 | 23.5 | Villages and farmlands | Good |
| 898 | Guilin | Longsheng County | Taxaceae | *Taxus wallichiana* var. *mairei* (Lemée & H. Lév.) L. K. Fu & Nan Li | 500 | 22.0 | 81.0 | 9.0 | Villages and farmlands | Good |
| 899 | Guilin | Longsheng County | Fagaceae | *Castanea henryi* (Skan) Rehder & E. H. Wilson | 500 | 20.0 | 140.0 | 18.0 | Villages and farmlands | Good |
| 900 | Guilin | Longsheng County | Taxaceae | *Taxus wallichiana* var. *mairei* (Lemée & H. Lév.) L. K. Fu & Nan Li | 500 | 35.0 | 120.0 | 14.0 | Villages and farmlands | Good |
| 901 | Guilin | Longsheng County | Cupressaceae | *Cunninghamia lan*ceolata (Lamb.) Hook. | 500 | 35.0 | 110.0 | 13.5 | Villages and farmlands | Good |
| 902 | Guilin | Longsheng County | Taxaceae | *Taxus wallichiana* var. *mairei* (Lemée & H. Lév.) L. K. Fu & Nan Li | 500 | 18.0 | 90.0 | 14.5 | Villages and farmlands | Good |
| 903 | Guilin | Longsheng County | Taxaceae | *Taxus wallichiana* var. *mairei* (Lemée & H. Lév.) L. K. Fu & Nan Li | 500 | 20.0 | 100.0 | 11.0 | Villages and farmlands | Good |
| 904 | Guilin | Longsheng County | Taxaceae | *Taxus wallichiana* var. *mairei* (Lemée & H. Lév.) L. K. Fu & Nan Li | 800 | 30.0 | 105.0 | 13.0 | Villages and farmlands | Good |
| 905 | Guilin | Longsheng County | Taxaceae | *Taxus wallichiana* var. *mairei* (Lemée & H. Lév.) L. K. Fu & Nan Li | 550 | 26.0 | 100.0 | 13.0 | Villages and farmlands | Good |
| 906 | Guilin | Longsheng County | Fagaceae | *Castanopsis chinensis* (Spreng.) Hance | 550 | 38.0 | 137.0 | 16.0 | Villages and farmlands | Poor |
| 907 | Guilin | Longsheng County | Podocarpaceae | *Nageia nagi* (Thunb.) Kuntze. | 550 | 35.0 | 152.0 | 11.3 | Villages and farmlands | Good |
| 908 | Guilin | Longsheng County | Podocarpaceae | *Nageia nagi* (Thunb.) Kuntze. | 550 | 34.0 | 114.0 | 12.0 | Villages and farmlands | Good |
| 909 | Guilin | Longsheng County | Fagaceae | *Castanopsis hystrix* Hook. f. & Thomson ex A. DC. | 550 | 26.0 | 92.3 | 9.0 | Villages and farmlands | Moderate |
| 910 | Guilin | Longsheng County | Fagaceae | *Castanopsis hystrix* Hook. f. & Thomson ex A. DC. | 550 | 23.0 | 136.0 | 7.0 | Villages and farmlands | Moderate |
| 911 | Guilin | Longsheng County | Fagaceae | *Castanopsis hystrix* Hook. f. & Thomson ex A. DC. | 550 | 32.0 | 143.0 | 16.0 | Villages and farmlands | Good |
| 912 | Guilin | Longsheng County | Fagaceae | *Castanopsis hystrix* Hook. f. & Thomson ex A. DC. | 500 | 32.0 | 143.0 | 16.0 | Villages and farmlands | Good |
| 913 | Guilin | Longsheng County | Fagaceae | *Castanopsis hystrix* Hook. f. & Thomson ex A. DC. | 500 | 23.0 | 136.0 | 7.0 | Villages and farmlands | Good |
| 914 | Guilin | Longsheng County | Cupressaceae | *Cunninghamia lanceolata* (Lamb.) Hook. | 700 | 40.0 | 160.0 | 7.0 | Villages and farmlands | Good |
| 915 | Guilin | Longsheng County | Hamamelidaceae | *Loropetalum chinense* (R. Br.) Oliv. | 800 | 4.0 | 60.0 | 8.0 | Villages and farmlands | Good |
| 916 | Guilin | Longsheng County | Fagaceae | *Castanopsis fargesii* Franch. | 900 | 21.0 | 74.0 | 9.0 | Wooded areas and plant nurseries | Good |
| 917 | Guilin | Longsheng County | Fagaceae | *Castanopsis fargesii* Franch. | 900 | 29.0 | 110.0 | 14.0 | Wooded areas and plant nurseries | Good |
| 918 | Guilin | Longsheng County | Fagaceae | *Castanopsis fargesii* Franch. | 800 | 19.0 | 64.0 | 8.0 | Wooded areas and plant nurseries | Good |
| 919 | Guilin | Longsheng County | Fagaceae | *Castanopsis faberi* Hance. | 800 | 27.0 | 228.0 | 13.5 | Wooded areas and plant nurseries | Good |
| 920 | Guilin | Longsheng County | Pinaceae | *Cathaya argyrophylla* Chun & Kuang | 500 | 12.9 | 42.0 | 10.5 | Wooded areas and plant nurseries | Good |
| 921 | Guilin | Longsheng County | Pinaceae | *Cathaya argyrophylla* Chun & Kuang | 500 | 14.4 | 41.4 | 3.9 | Wooded areas and plant nurseries | Good |
| 922 | Guilin | Longsheng County | Pinaceae | *Cathaya argyrophylla* Chun & Kuang | 500 | 14.6 | 44.3 | 6.3 | Wooded areas and plant nurseries | Good |
| 923 | Guilin | Longsheng County | Pinaceae | *Cathaya argyrophylla* Chun & Kuang | 500 | 13.1 | 48.0 | 5.4 | Wooded areas and plant nurseries | Good |
| 924 | Guilin | Longsheng County | Pinaceae | *Cathaya argyrophylla* Chun & Kuang | 500 | 9.3 | 51.9 | 6.5 | Wooded areas and plant nurseries | Good |
| 925 | Guilin | Longsheng County | Pinaceae | *Cathaya argyrophylla* Chun & Kuang | 500 | 9.8 | 42.0 | 7.4 | Wooded areas and plant nurseries | Good |
| 926 | Guilin | Longsheng County | Pinaceae | *Cathaya argyrophylla* Chun & Kuang | 500 | 15.4 | 42.7 | 6.8 | Wooded areas and plant nurseries | Good |
| 927 | Guilin | Longsheng County | Pinaceae | *Cathaya argyrophylla* Chun & Kuang | 500 | 9.8 | 37.3 | 7.0 | Wooded areas and plant nurseries | Good |
| 928 | Guilin | Longsheng County | Pinaceae | *Cathaya argyrophylla* Chun & Kuang | 500 | 15.0 | 56.0 | 4.3 | Wooded areas and plant nurseries | Good |
| 929 | Guilin | Longsheng County | Pinaceae | *Cathaya argyrophylla* Chun & Kuang | 500 | 6.5 | 50.3 | 7.3 | Wooded areas and plant nurseries | Good |
| 930 | Guilin | Longsheng County | Pinaceae | *Cathaya argyrophylla* Chun & Kuang | 500 | 8.5 | 45.2 | 7.8 | Wooded areas and plant nurseries | Good |
| 931 | Guilin | Longsheng County | Pinaceae | *Cathaya argyrophylla* Chun & Kuang | 500 | 13.2 | 44.0 | 6.8 | Wooded areas and plant nurseries | Good |
| 932 | Guilin | Longsheng County | Pinaceae | *Cathaya argyrophylla* Chun & Kuang | 500 | 13.0 | 44.0 | 5.2 | Wooded areas and plant nurseries | Good |
| 933 | Guilin | Longsheng County | Pinaceae | *Cathaya argyrophylla* Chun & Kuang | 500 | 12.5 | 43.6 | 5.1 | Wooded areas and plant nurseries | Good |
| 934 | Guilin | Longsheng County | Pinaceae | *Cathaya argyrophylla* Chun & Kuang | 500 | 10.0 | 24.0 | 4.3 | Wooded areas and plant nurseries | Good |
| 935 | Guilin | Longsheng County | Pinaceae | *Cathaya argyrophylla* Chun & Kuang | 500 | 9.6 | 20.7 | 3.5 | Wooded areas and plant nurseries | Good |
| 936 | Guilin | Longsheng County | Pinaceae | *Cathaya argyrophylla* Chun & Kuang | 500 | 7.0 | 26.1 | 6.1 | Wooded areas and plant nurseries | Good |
| 937 | Guilin | Longsheng County | Elaeocarpaceae | *Elaeocarpus limitaneus* Hand.-Mazz. | 700 | 17.0 | 70.0 | 8.5 | Wooded areas and plant nurseries | Good |
| 938 | Guilin | Longsheng County | Theaceae | *Schima argentea* E. Pritz. | 500 | 13.0 | 95.0 | 8.5 | Wooded areas and plant nurseries | Good |
| 939 | Guilin | Longsheng County | Theaceae | *Schima argentea* E. Pritz. | 500 | 17.0 | 75.0 | 6.5 | Wooded areas and plant nurseries | Good |
| 940 | Guilin | Ziyuan County | Lauraceae | *Camphora officinarum Nees* | 600 | 15.0 | 228.0 | 18.5 | Residential districts | Good |
| 941 | Guilin | Ziyuan County | Taxaceae | *Taxus wallichiana* var. *mairei* (Lemée & H. Lév.) L. K. Fu & Nan Li | 640 | 22.0 | 118.0 | 13.5 | Villages and farmlands | Good |
| 942 | Guilin | Ziyuan County | Taxaceae | *Taxus wallichiana* var. *mairei* (Lemée & H. Lév.) L. K. Fu & Nan Li | 540 | 16.0 | 108.0 | 12.5 | Villages and farmlands | Good |
| 943 | Guilin | Ziyuan County | Taxaceae | *Taxus wallichiana* var. *mairei* (Lemée & H. Lév.) L. K. Fu & Nan Li | 700 | 24.0 | 124.0 | 19.0 | Villages and farmlands | Good |
| 944 | Guilin | Ziyuan County | Taxaceae | *Taxus wallichiana* var. *mairei* (Lemée & H. Lév.) L. K. Fu & Nan Li | 520 | 17.0 | 108.0 | 11.0 | Villages and farmlands | Good |
| 945 | Guilin | Ziyuan County | Taxaceae | *Taxus wallichiana* var. *mairei* (Lemée & H. Lév.) L. K. Fu & Nan Li | 650 | 22.0 | 118.0 | 11.5 | Villages and farmlands | Good |
| 946 | Guilin | Ziyuan County | Taxaceae | *Taxus wallichiana* var. *mairei* (Lemée & H. Lév.) L. K. Fu & Nan Li | 500 | 22.0 | 104.0 | 11.5 | Villages and farmlands | Good |
| 947 | Guilin | Ziyuan County | Taxaceae | *Taxus wallichiana* var. *mairei* (Lemée & H. Lév.) L. K. Fu & Nan Li | 500 | 20.0 | 99.0 | 13.0 | Villages and farmlands | Good |
| 948 | Guilin | Ziyuan County | Taxaceae | *Taxus wallichiana* var. *mairei* (Lemée & H. Lév.) L. K. Fu & Nan Li | 550 | 25.0 | 110.0 | 18.5 | Villages and farmlands | Good |
| 949 | Guilin | Ziyuan County | Taxaceae | *Taxus wallichiana* var. *mairei* (Lemée & H. Lév.) L. K. Fu & Nan Li | 540 | 18.0 | 108.0 | 16.0 | Villages and farmlands | Good |
| 950 | Guilin | Ziyuan County | Lauraceae | *Camphora officinarum Nees* | 500 | 26.0 | 213.0 | 24.5 | Villages and farmlands | Moderate |
| 951 | Guilin | Ziyuan County | Ulmaceae | *Zelkova schneideriana* Hand.-Mazz. | 500 | 34.0 | 147.0 | 24.5 | Villages and farmlands | Poor |
| 952 | Guilin | Ziyuan County | Ulmaceae | *Zelkova schneideriana* Hand.-Mazz. | 500 | 21.0 | 172.0 | 12.5 | Wooded areas and plant nurseries | Moderate |
| 953 | Guilin | Ziyuan County | Taxaceae | *Taxus wallichiana* var. *mairei* (Lemée & H. Lév.) L. K. Fu & Nan Li | 520 | 17.0 | 108.0 | 16.0 | Villages and farmlands | Moderate |
| 954 | Guilin | Ziyuan County | Taxaceae | *Taxus wallichiana* var. *mairei* (Lemée & H. Lév.) L. K. Fu & Nan Li | 700 | 22.0 | 123.0 | 15.0 | Villages and farmlands | Moderate |
| 955 | Guilin | Ziyuan County | Fagaceae | *Castanopsis carlesii* (Hemsl.) Hayata | 500 | 6.0 | 120.9 | 5.5 | Villages and farmlands | Good |
| 956 | Guilin | Ziyuan County | Fagaceae | *Castanea henryi (*Skan) Rehder & E. H. Wilson | 500 | 14.0 | 121.0 | 10.5 | Villages and farmlands | Good |
| 957 | Guilin | Ziyuan County | Theaceae | *Schima argentea* E. Pritz. | 500 | 26.0 | 113.0 | 34.0 | Villages and farmlands | Good |
| 958 | Guilin | Ziyuan County | Fagaceae | *Quercus variabil*is Blume. | 650 | 31.0 | 122.0 | 23.5 | Villages and farmlands | Good |
| 959 | Guilin | Ziyuan County | Fagaceae | *Castanea henryi* (Skan) Rehder & E. H. Wilson | 500 | 21.0 | 129.0 | 17.5 | Villages and farmlands | Poor |
| 960 | Guilin | Ziyuan County | Cupressaceae | *Cryptomeria japonica* var. sinensis Miq. | 500 | 21.0 | 120.0 | 9.0 | Villages and farmlands | Good |
| 961 | Guilin | Ziyuan County | Ginkgoaceae | *Ginkgo biloba* L. | 500 | 30.0 | 167.0 | 21.0 | Villages and farmlands | Good |
| 962 | Guilin | Ziyuan County | Cupressaceae | *Cunninghamia lanceolata* (Lamb.) Hook. | 500 | 35.0 | 113.0 | 7.5 | Villages and farmlands | Good |
| 963 | Guilin | Ziyuan County | Juglandaceae | *Pterocarya stenoptera* C. DC. | 800 | 22.0 | 210.0 | 13.5 | Villages and farmlands | Moderate |
| 964 | Guilin | Ziyuan County | Fagaceae | *Castanopsis tibetana* Hance. | 500 | 23.0 | 140.0 | 12.5 | Villages and farmlands | Good |
| 965 | Guilin | Ziyuan County | Fagaceae | *Castanopsis tibetana* Hance. | 500 | 25.0 | 140.0 | 20.5 | Villages and farmlands | Good |
| 966 | Guilin | Ziyuan County | Oleaceae | *Osmanthus fragrans* (Thunb.) Lour. | 550 | 10.0 | 97.0 | 7.0 | Villages and farmlands | Moderate |
| 967 | Guilin | Ziyuan County | Taxaceae | *Taxus wallichiana* var. *mairei* (Lemée & H. Lév.) L. K. Fu & Nan Li | 500 | 18.0 | 106.0 | 15.0 | Villages and farmlands | Good |
| 968 | Guilin | Ziyuan County | Fagaceae | *Castanea henryi* (Skan) Rehder & E. H. Wilson | 550 | 30.0 | 131.0 | 10.5 | Villages and farmlands | Poor |
| 969 | Guilin | Ziyuan County | Pinaceae | *Tsuga chinensis* (Franch.) E. Pritz. | 750 | 12.0 | 62.7 | 9.6 | Wooded areas and plant nurseries | Good |
| 970 | Guilin | Ziyuan County | Pinaceae | *Tsuga chinensis* (Franch.) E. Pritz. | 900 | 14.3 | 78.0 | 11.5 | Wooded areas and plant nurseries | Good |
| 971 | Guilin | Ziyuan County | Pinaceae | *Tsuga chinensis* (Franch.) E. Pritz. | 900 | 15.0 | 77.0 | 11.8 | Wooded areas and plant nurseries | Good |
| 972 | Guilin | Ziyuan County | Pinaceae | *Tsuga chinensis* (Franch.) E. Pritz. | 900 | 13.0 | 76.7 | 10.2 | Wooded areas and plant nurseries | Good |
| 973 | Guilin | Ziyuan County | Pinaceae | *Tsuga chinensis* (Franch.) E. Pritz. | 900 | 14.0 | 76.0 | 10.8 | Wooded areas and plant nurseries | Good |
| 974 | Guilin | Ziyuan County | Pinaceae | *Tsuga chinensis* (Franch.) E. Pritz. | 900 | 13.5 | 76.0 | 10.8 | Wooded areas and plant nurseries | Good |
| 975 | Guilin | Ziyuan County | Pinaceae | *Nothotsuga longibracteata* (W. C. Cheng) Hu ex C. N. Page | 900 | 26.0 | 75.7 | 14.5 | Wooded areas and plant nurseries | Good |
| 976 | Guilin | Ziyuan County | Pinaceae | *Nothotsuga longibracteata* (W. C. Cheng) Hu ex C. N. Page | 900 | 26.0 | 75.7 | 14.0 | Wooded areas and plant nurseries | Good |
| 977 | Guilin | Ziyuan County | Pinaceae | *Nothotsuga longibracteata* (W. C. Cheng) Hu ex C. N. Page | 900 | 26.0 | 75.5 | 13.5 | Wooded areas and plant nurseries | Good |
| 978 | Guilin | Ziyuan County | Pinaceae | *Tsuga chinensis* (Franch.) E. Pritz. | 900 | 13.0 | 75.0 | 10.5 | Wooded areas and plant nurseries | Good |
| 979 | Guilin | Ziyuan County | Pinaceae | *Tsuga chinensis* (Franch.) E. Pritz. | 900 | 12.0 | 75.0 | 10.1 | Wooded areas and plant nurseries | Good |
| 980 | Guilin | Ziyuan County | Pinaceae | *Tsuga chinensis* (Franch.) E. Pritz. | 900 | 13.0 | 75.0 | 10.5 | Wooded areas and plant nurseries | Good |
| 981 | Guilin | Ziyuan County | Pinaceae | *Tsuga chinensis* (Franch.) E. Pritz. | 900 | 13.0 | 75.0 | 10.0 | Wooded areas and plant nurseries | Good |
| 982 | Guilin | Ziyuan County | Pinaceae | *Tsuga chinensis* (Franch.) E. Pritz. | 900 | 10.0 | 75.0 | 9.8 | Wooded areas and plant nurseries | Good |
| 983 | Guilin | Ziyuan County | Pinaceae | *Tsuga chinensis* (Franch.) E. Pritz. | 900 | 13.5 | 74.4 | 11.5 | Wooded areas and plant nurseries | Good |
| 984 | Guilin | Ziyuan County | Pinaceae | *Nothotsuga longibracteata* (W. C. Cheng) Hu ex C. N. Page | 900 | 32.0 | 74.3 | 14.0 | Wooded areas and plant nurseries | Good |
| 985 | Guilin | Ziyuan County | Pinaceae | *Tsuga chinensis* (Franch.) E. Pritz. | 900 | 13.0 | 74.3 | 10.3 | Wooded areas and plant nurseries | Good |
| 986 | Guilin | Ziyuan County | Pinaceae | *Tsuga chinensis* (Franch.) E. Pritz. | 800 | 12.0 | 74.0 | 10.8 | Wooded areas and plant nurseries | Good |
| 987 | Guilin | Ziyuan County | Pinaceae | *Nothotsuga longibracteata* (W. C. Cheng) Hu ex C. N. Page | 900 | 33.0 | 73.1 | 14.0 | Wooded areas and plant nurseries | Good |
| 988 | Guilin | Ziyuan County | Pinaceae | *Nothotsuga longibracteata* (W. C. Cheng) Hu ex C. N. Page | 900 | 19.0 | 73.0 | 10.8 | Wooded areas and plant nurseries | Good |
| 989 | Guilin | Ziyuan County | Pinaceae | *Tsuga chinensis* (Franch.) E. Pritz. | 900 | 15.0 | 73.0 | 12.4 | Wooded areas and plant nurseries | Good |
| 990 | Guilin | Ziyuan County | Pinaceae | *Tsuga chinensis* (Franch.) E. Pritz. | 900 | 13.0 | 70.0 | 10.8 | Wooded areas and plant nurseries | Good |
| 991 | Guilin | Ziyuan County | Pinaceae | *Tsuga chinensis* (Franch.) E. Pritz. | 900 | 15.0 | 72.0 | 12.3 | Wooded areas and plant nurseries | Good |
| 992 | Guilin | Ziyuan County | Pinaceae | *Tsuga chinensis* (Franch.) E. Pritz. | 900 | 15.0 | 72.0 | 11.9 | Wooded areas and plant nurseries | Good |
| 993 | Guilin | Ziyuan County | Pinaceae | *Tsuga chinensis* (Franch.) E. Pritz. | 900 | 16.0 | 71.5 | 12.9 | Wooded areas and plant nurseries | Good |
| 994 | Guilin | Ziyuan County | Pinaceae | *Tsuga chinensis* (Franch.) E. Pritz. | 900 | 15.0 | 71.5 | 11.5 | Wooded areas and plant nurseries | Good |
| 995 | Guilin | Ziyuan County | Pinaceae | *Tsuga chinensis* (Franch.) E. Pritz. | 900 | 14.0 | 75.7 | 10.8 | Wooded areas and plant nurseries | Good |
| 996 | Guilin | Ziyuan County | Pinaceae | *Tsuga chinensis* (Franch.) E. Pritz. | 500 | 18.0 | 56.0 | 7.5 | Wooded areas and plant nurseries | Good |
| 997 | Guilin | Ziyuan County | Pinaceae | *Tsuga chinensis* (Franch.) E. Pritz. | 900 | 18.0 | 73.0 | 8.5 | Wooded areas and plant nurseries | Good |
| 998 | Guilin | Ziyuan County | Pinaceae | *Tsuga chinensis* (Franch.) E. Pritz. | 500 | 14.0 | 55.0 | 7.5 | Wooded areas and plant nurseries | Good |
| 999 | Guilin | Ziyuan County | Pinaceae | *Tsuga chinensis* (Franch.) E. Pritz. | 800 | 24.0 | 64.0 | 10.3 | Wooded areas and plant nurseries | Good |
| 1000 | Guilin | Ziyuan County | Pinaceae | *Tsuga chinensis* (Franch.) E. Pritz. | 500 | 23.0 | 55.0 | 12.5 | Wooded areas and plant nurseries | Good |
| 1001 | Guilin | Ziyuan County | Pinaceae | *Tsuga chinensis* (Franch.) E. Pritz. | 900 | 24.0 | 68.0 | 9.3 | Wooded areas and plant nurseries | Good |
| 1002 | Guilin | Ziyuan County | Pinaceae | *Tsuga chinensis* (Franch.) E. Pritz. | 800 | 24.0 | 64.0 | 10.3 | Wooded areas and plant nurseries | Good |
| 1003 | Guilin | Ziyuan County | Pinaceae | *Tsuga chinensis* (Franch.) E. Pritz. | 800 | 24.0 | 64.0 | 10.8 | Wooded areas and plant nurseries | Good |
| 1004 | Guilin | Ziyuan County | Pinaceae | *Tsuga chinensis* (Franch.) E. Pritz. | 500 | 20.0 | 55.0 | 11.0 | Wooded areas and plant nurseries | Good |
| 1005 | Guilin | Ziyuan County | Pinaceae | *Tsuga chinensis* (Franch.) E. Pritz. | 500 | 22.0 | 55.5 | 11.5 | Wooded areas and plant nurseries | Good |
| 1006 | Guilin | Ziyuan County | Pinaceae | *Tsuga chinensis* (Franch.) E. Pritz. | 500 | 24.0 | 55.0 | 11.0 | Wooded areas and plant nurseries | Good |
| 1007 | Guilin | Ziyuan County | Pinaceae | *Tsuga chinensis* (Franch.) E. Pritz. | 700 | 23.0 | 61.0 | 10.5 | Wooded areas and plant nurseries | Good |
| 1008 | Guilin | Ziyuan County | Pinaceae | *Tsuga chinensis* (Franch.) E. Pritz. | 700 | 21.0 | 61.0 | 11.5 | Wooded areas and plant nurseries | Good |
| 1009 | Guilin | Ziyuan County | Pinaceae | *Tsuga chinensis* (Franch.) E. Pritz. | 650 | 22.0 | 60.5 | 10.0 | Wooded areas and plant nurseries | Good |
| 1010 | Guilin | Ziyuan County | Pinaceae | *Tsuga chinensis* (Franch.) E. Pritz. | 650 | 20.0 | 60.0 | 10.5 | Wooded areas and plant nurseries | Good |
| 1011 | Guilin | Ziyuan County | Pinaceae | *Tsuga chinensis* (Franch.) E. Pritz. | 650 | 22.0 | 59.0 | 10.3 | Wooded areas and plant nurseries | Good |
| 1012 | Guilin | Ziyuan County | Pinaceae | *Tsuga chinensis* (Franch.) E. Pritz. | 750 | 22.0 | 63.0 | 11.5 | Wooded areas and plant nurseries | Good |
| 1013 | Guilin | Ziyuan County | Pinaceae | *Tsuga chinensis* (Franch.) E. Pritz. | 500 | 20.0 | 55.0 | 10.5 | Wooded areas and plant nurseries | Good |
| 1014 | Guilin | Ziyuan County | Pinaceae | *Tsuga chinensis* (Franch.) E. Pritz. | 520 | 20.0 | 56.0 | 10.5 | Wooded areas and plant nurseries | Good |
| 1015 | Guilin | Ziyuan County | Pinaceae | *Tsuga chinensis* (Franch.) E. Pritz. | 500 | 19.0 | 55.0 | 10.5 | Wooded areas and plant nurseries | Good |
| 1016 | Guilin | Ziyuan County | Pinaceae | *Tsuga chinensis* (Franch.) E. Pritz. | 950 | 16.0 | 67.0 | 7.5 | Wooded areas and plant nurseries | Good |
| 1017 | Guilin | Ziyuan County | Pinaceae | *Tsuga chinensis* (Franch.) E. Pritz. | 700 | 20.0 | 61.5 | 6.5 | Wooded areas and plant nurseries | Good |
| 1018 | Guilin | Ziyuan County | Pinaceae | *Tsuga chinensis* (Franch.) E. Pritz. | 650 | 20.0 | 60.5 | 8.3 | Wooded areas and plant nurseries | Good |
| 1019 | Guilin | Ziyuan County | Pinaceae | *Tsuga chinensis* (Franch.) E. Pritz. | 550 | 19.0 | 56.0 | 7.8 | Wooded areas and plant nurseries | Good |
| 1020 | Guilin | Ziyuan County | Pinaceae | *Tsuga chinensis* (Franch.) E. Pritz. | 650 | 23.0 | 60.0 | 7.5 | Wooded areas and plant nurseries | Good |
| 1021 | Guilin | Ziyuan County | Pinaceae | *Tsuga chinensis* (Franch.) E. Pritz. | 750 | 23.0 | 62.0 | 9.3 | Wooded areas and plant nurseries | Good |
| 1022 | Guilin | Pingle County | Moraceae | *Ficus microcarpa* L. f. | 600 | 15.0 | 359.0 | 39.0 | Villages and farmlands | Good |
| 1023 | Guilin | Pingle County | Moraceae | *Ficus microcarpa* L. f. | 508 | 16.3 | 261.0 | 32.9 | Residential districts | Good |
| 1024 | Guilin | Pingle County | Moraceae | *Ficus microcarpa* L. f. | 508 | 15.9 | 257.0 | 27.5 | Residential districts | Good |
| 1025 | Guilin | Pingle County | Fagaceae | *Quercus variabilis* Blume | 650 | 16.7 | 120.0 | 14.7 | Villages and farmlands | Good |
| 1026 | Guilin | Pingle County | Moraceae | *Ficus microcarpa* L. f. | 730 | 23.5 | 293.0 | 39.5 | Villages and farmlands | Good |
| 1027 | Guilin | Pingle County | Moraceae | *Ficus microcarpa* L. f. | 500 | 18.2 | 257.0 | 22.2 | Villages and farmlands | Good |
| 1028 | Guilin | Pingle County | Moraceae | *Ficus virens* Aiton. | 510 | 16.0 | 250.0 | 21.0 | Villages and farmlands | Good |
| 1029 | Guilin | Pingle County | Lauraceae | *Camphora officinarum Nees.* | 520 | 21.0 | 229.0 | 32.5 | Villages and farmlands | Good |
| 1030 | Guilin | Pingle County | Moraceae | *Ficus microcarpa* L. f. | 510 | 19.0 | 255.0 | 32.5 | Villages and farmlands | Good |
| 1031 | Guilin | Pingle County | Lauraceae | *Camphora officinarum Nees.* | 500 | 23.0 | 210.0 | 29.0 | Villages and farmlands | Good |
| 1032 | Guilin | Pingle County | Lauraceae | *Camphora officinarum Nees.* | 500 | 22.0 | 208.7 | 32.0 | Villages and farmlands | Good |
| 1033 | Guilin | Pingle County | Moraceae | *Ficus microcarpa* L. f. | 500 | 14.5 | 228.9 | 33.0 | Villages and farmlands | Good |
| 1034 | Guilin | Pingle County | Lauraceae | *Camphora officinarum Nees.* | 500 | 23.0 | 200.0 | 32.5 | Villages and farmlands | Good |
| 1035 | Guilin | Pingle County | Moraceae | *Ficus microcarpa*L. f. | 580 | 23.5 | 299.2 | 38.0 | Villages and farmlands | Good |
| 1036 | Guilin | Pingle County | Moraceae | *Ficus microcarpa*L. f. | 600 | 16.0 | 302.5 | 18.0 | Villages and farmlands | Good |
| 1037 | Guilin | Pingle County | Lauraceae | *Camphora officinarum Nees.* | 510 | 18.6 | 216.3 | 18.2 | Villages and farmlands | Good |
| 1038 | Guilin | Pingle County | Moraceae | *Ficus concinna (*Miq.) Miq. | 700 | 32.0 | 340.7 | 39.0 | Villages and farmlands | Good |
| 1039 | Guilin | Pingle County | Moraceae | *Ficus microcarpa* L. f. | 500 | 17.0 | 258.0 | 33.0 | Residential districts | Good |
| 1040 | Guilin | Pingle County | Moraceae | *Ficus microcarpa* L. f. | 550 | 17.0 | 280.2 | 19.2 | Villages and farmlands | Good |
| 1041 | Guilin | Pingle County | Moraceae | *Ficus microcarpa* L. f. | 505 | 17.5 | 260.0 | 34.2 | Villages and farmlands | Good |
| 1042 | Guilin | Pingle County | Moraceae | *Ficus microcarpa* L. f. | 500 | 15.5 | 263.0 | 23.2 | Villages and farmlands | Good |
| 1043 | Guilin | Pingle County | Lauraceae | *Camphora officinarum Nees.* | 610 | 20.0 | 251.0 | 36.0 | Villages and farmlands | Good |
| 1044 | Guilin | Pingle County | Moraceae | *Ficus microcarpa* L. f. | 730 | 22.0 | 294.0 | 32.0 | Villages and farmlands | Good |
| 1045 | Guilin | Pingle County | Moraceae | *Ficus microcarpa* L. f. | 650 | 15.0 | 290.2 | 42.0 | Villages and farmlands | Good |
| 1046 | Guilin | Pingle County | Anacardiaceae | *Pistacia chinensis* Bunge. | 500 | 26.0 | 190.2 | 31.0 | Villages and farmlands | Poor |
| 1047 | Guilin | Pingle County | Moraceae | *Ficus microcarpa* L. f. | 500 | 15.0 | 256.7 | 35.0 | Villages and farmlands | Good |
| 1048 | Guilin | Pingle County | Lauraceae | *Camphora officinarum Nees.* | 500 | 32.0 | 219.8 | 12.0 | Villages and farmlands | Good |
| 1049 | Guilin | Pingle County | Lauraceae | *Camphora officinarum Nees.* | 530 | 22.0 | 227.1 | 42.0 | Villages and farmlands | Good |
| 1050 | Guilin | Pingle County | Lauraceae | *Camphora officinarum Nees.* | 730 | 25.0 | 277.1 | 41.0 | Villages and farmlands | Good |
| 1051 | Guilin | Pingle County | Moraceae | *Ficus microcarpa* L. f. | 600 | 17.0 | 274.8 | 34.0 | Villages and farmlands | Good |
| 1052 | Guilin | Pingle County | Moraceae | *Ficus microcarpa* L. f. | 510 | 29.0 | 258.9 | 29.0 | Villages and farmlands | Good |
| 1053 | Guilin | Pingle County | Lauraceae | *Camphora officinarum Nees.* | 850 | 15.0 | 302.6 | 7.0 | Villages and farmlands | Good |
| 1054 | Guilin | Pingle County | Lauraceae | *Camphora officinarum Nees.* | 500 | 24.0 | 216.6 | 25.0 | Villages and farmlands | Good |
| 1055 | Guilin | Pingle County | Moraceae | *Ficus microcarpa* L. f. | 500 | 21.0 | 338.2 | 41.0 | Villages and farmlands | Good |
| 1056 | Guilin | Pingle County | Lauraceae | *Camphora officinarum Nees.* | 550 | 19.0 | 213.4 | 22.0 | Villages and farmlands | Good |
| 1057 | Guilin | Pingle County | Lauraceae | *Camphora officinarum Nees.* | 560 | 18.0 | 238.6 | 28.0 | Villages and farmlands | Good |
| 1058 | Guilin | Pingle County | Moraceae | *Ficus microcarpa* L. f. | 500 | 24.0 | 254.1 | 32.0 | Villages and farmlands | Good |
| 1059 | Guilin | Pingle County | Moraceae | *Ficus microcarpa* L. f. | 505 | 10.0 | 255.0 | 32.5 | Villages and farmlands | Good |
| 1060 | Guilin | Pingle County | Lauraceae | *Camphora officinarum Nees.* | 510 | 18.0 | 250.0 | 18.0 | Villages and farmlands | Good |
| 1061 | Guilin | Pingle County | Moraceae | *Ficus microcarpa* L. f. | 510 | 21.0 | 267.0 | 28.5 | Villages and farmlands | Good |
| 1062 | Guilin | Lipu City | Oleaceae | *Osmanthus fragrans* (Thunb.) Lour. | 550 | 7.0 | 96.0 | 11.0 | Villages and farmlands | Good |
| 1063 | Guilin | Lipu City | Oleaceae | *Osmanthus fragrans* (Thunb.) Lour. | 800 | 8.0 | 102.0 | 16.5 | Villages and farmlands | Good |
| 1064 | Guilin | Lipu City | Lauraceae | *Camphora officinarum Nees.* | 600 | 24.2 | 248.0 | 38.5 | Villages and farmlands | Good |
| 1065 | Guilin | Lipu City | Moraceae | *Ficus microcarpa* L. f. | 650 | 18.4 | 283.1 | 41.5 | Villages and farmlands | Good |
| 1066 | Guilin | Lipu City | Moraceae | *Ficus microcarpa* L. f. | 615 | 24.8 | 277.0 | 43.4 | Villages and farmlands | Good |
| 1067 | Guilin | Lipu City | Moraceae | *Ficus microcarpa* L. f. | 600 | 14.3 | 273.0 | 34.0 | Villages and farmlands | Good |
| 1068 | Guilin | Lipu City | Capparaceae | *Crateva religiosa* G. Forster. | 550 | 16.0 | 108.3 | 9.0 | Villages and farmlands | Good |
| 1069 | Guilin | Lipu City | Moraceae | *Artocarpus styracifolius* Pierre | 500 | 18.0 | 153.0 | 8.5 | Villages and farmlands | Moderate |
| 1070 | Guilin | Lipu City | Lauraceae | *Camphora officinarum Nees.* | 640 | 22.5 | 259.0 | 27.0 | Villages and farmlands | Good |
| 1071 | Guilin | Lipu City | Lauraceae | *Camphora officinarum Nees.* | 600 | 35.1 | 245.2 | 39.5 | Villages and farmlands | Good |
| 1072 | Guilin | Lipu City | Moraceae | *Ficus microcarpa* L. f. | 850 | 16.1 | 305.7 | 25.5 | Villages and farmlands | Good |
| 1073 | Guilin | Lipu City | Lauraceae | *Camphora officinarum Nees.* | 900 | 13.0 | 310.0 | 3.0 | Villages and farmlands | Good |
| 1074 | Guilin | Lipu City | Theaceae | *Schima superba* Gardner & Champ. | 500 | 30.0 | 120.0 | 15.0 | Villages and farmlands | Good |
| 1075 | Guilin | Lipu City | Theaceae | *Schima superba* Gardner & Champ. | 520 | 26.8 | 121.6 | 13.0 | Villages and farmlands | Good |
| 1076 | Guilin | Gongcheng County | Lauraceae | *Camphora officinarum Nees.* | 510 | 24.0 | 224.0 | 22.0 | Villages and farmlands | Good |
| 1077 | Guilin | Gongcheng County | Lauraceae | *Camphora officinarum Nees.* | 620 | 17.0 | 252.0 | 25.0 | Villages and farmlands | Good |
| 1078 | Guilin | Gongcheng County | Lauraceae | *Camphora officinarum Nees.* | 550 | 23.0 | 236.0 | 27.0 | Villages and farmlands | Good |
| 1079 | Guilin | Gongcheng County | Lauraceae | *Camphora officinarum Nees.* | 600 | 20.0 | 250.0 | 39.0 | Villages and farmlands | Good |
| 1080 | Guilin | Gongcheng County | Oleaceae | *Osmanthus fragrans* (Thunb.) Lour. | 500 | 15.0 | 94.0 | 16.0 | Villages and farmlands | Good |
| 1081 | Guilin | Gongcheng County | Moraceae | *Ficus microcarpa* L. f. | 630 | 25.0 | 280.0 | 36.0 | Villages and farmlands | Good |
| 1082 | Guilin | Gongcheng County | Moraceae | *Ficus microcarpa* L. f. | 630 | 29.0 | 277.0 | 37.0 | Villages and farmlands | Good |
| 1083 | Guilin | Gongcheng County | Moraceae | *Ficus microcarpa* L. f. | 630 | 29.0 | 277.0 | 37.0 | Villages and farmlands | Good |
| 1084 | Guilin | Gongcheng County | Fabaceae | *Gleditsia sinensis* Lam. | 500 | 35.0 | 146.0 | 24.0 | Villages and farmlands | Good |
| 1085 | Guilin | Gongcheng County | Lauraceae | *Camphora officinarum Nees.* | 550 | 43.0 | 237.0 | 32.0 | Villages and farmlands | Good |
| 1086 | Guilin | Gongcheng County | Lauraceae | *Camphora officinarum Nees.* | 580 | 39.0 | 245.0 | 37.0 | Villages and farmlands | Good |
| 1087 | Guilin | Gongcheng County | Lauraceae | *Camphora officinarum Nees.* | 580 | 19.0 | 245.0 | 29.0 | Villages and farmlands | Good |
| 1088 | Guilin | Gongcheng County | Lauraceae | *Camphora officinarum Nees.* | 650 | 32.0 | 236.0 | 22.0 | Villages and farmlands | Good |
| 1089 | Guilin | Gongcheng County | Theaceae | *Schima superba* Gardner & Champ. | 500 | 27.0 | 121.0 | 6.0 | Villages and farmlands | Good |
| 1090 | Guilin | Gongcheng County | Theaceae | *Schima superba* Gardner & Champ. | 500 | 24.0 | 120.0 | 11.0 | Villages and farmlands | Good |
| 1091 | Guilin | Gongcheng County | Theaceae | *Schima superba* Gardner & Champ. | 500 | 27.0 | 120.0 | 10.0 | Villages and farmlands | Good |
| 1092 | Guilin | Gongcheng County | Taxaceae | *Taxus wallichiana* var. *mairei* (Lemée & H. Lév.) L. K. Fu & Nan Li | 580 | 28.0 | 114.0 | 17.0 | Villages and farmlands | Good |
| 1093 | Guilin | Gongcheng County | Lauraceae | *Camphora officinarum Nees.* | 550 | 15.0 | 245.0 | 13.0 | Villages and farmlands | Good |
| 1094 | Guilin | Gongcheng County | Lauraceae | *Camphora officinarum Nees.* | 620 | 28.0 | 252.0 | 21.0 | Villages and farmlands | Good |
| 1095 | Guilin | Gongcheng County | Lauraceae | *Camphora officinarum Nees.* | 555 | 15.0 | 239.0 | 11.0 | Villages and farmlands | Good |
| 1096 | Guilin | Gongcheng County | Meliaceae | *Toona ciliata* M. Roem. | 600 | 25.0 | 220.0 | 7.3 | Villages and farmlands | Good |
| 1097 | Guilin | Gongcheng County | Ginkgoaceae | *Ginkgo biloba* L. | 800 | 23.0 | 299.0 | 17.0 | Villages and farmlands | Good |
| 1098 | Guilin | Gongcheng County | Oleaceae | *Osmanthus fragrans* (Thunb.) Lour. | 500 | 10.0 | 93.0 | 10.0 | Villages and farmlands | Good |
| 1099 | Guilin | Gongcheng County | Lauraceae | *Camphora officinarum Nees.* | 500 | 28.0 | 228.0 | 20.0 | Villages and farmlands | Good |
| 1100 | Guilin | Gongcheng County | Fagaceae | *Castanopsis chinensis* (Spreng.) Hance | 900 | 18.0 | 204.0 | 16.0 | Villages and farmlands | Good |
| 1101 | Guilin | Gongcheng County | Lauraceae | *Camphora officinarum Nees.* | 600 | 18.0 | 252.0 | 18.0 | Villages and farmlands | Good |
| 1102 | Guilin | Gongcheng County | Lauraceae | *Camphora officinarum Nees.* | 550 | 20.0 | 235.0 | 22.0 | Villages and farmlands | Good |
| 1103 | Guilin | Gongcheng County | Moraceae | *Ficus microcarpa* L. f. | 600 | 16.0 | 300.0 | 25.0 | Villages and farmlands | Good |
| 1104 | Guilin | Gongcheng County | Fagaceae | *Castanopsis carlesii* (Hemsl.) Hayata | 550 | 14.0 | 142.5 | 11.0 | Villages and farmlands | Good |
| 1105 | Guilin | Gongcheng County | Moraceae | *Ficus microcarpa* L. f. | 590 | 15.0 | 270.0 | 20.0 | Villages and farmlands | Good |
| 1106 | Guilin | Gongcheng County | Lauraceae | *Camphora officinarum Nees.* | 550 | 25.0 | 234.6 | 31.0 | Villages and farmlands | Good |
| 1107 | Guilin | Gongcheng County | Lauraceae | *Camphora officinarum Nees.* | 600 | 22.0 | 250.2 | 20.0 | Villages and farmlands | Good |
| 1108 | Guilin | Gongcheng County | Lauraceae | *Camphora officinarum Nees.* | 500 | 25.0 | 223.0 | 27.5 | Villages and farmlands | Good |
| 1109 | Wuzhou | Changzhou District | Moraceae | *Ficus concinna* (Miq.) Miq. | 1000 | 20.0 | 270.0 | 39.0 | Villages and farmlands | Good |
| 1110 | Wuzhou | Changzhou District | Moraceae | *Ficus concinna* (Miq.) Miq. | 1000 | 20.0 | 280.0 | 29.0 | Villages and farmlands | Good |
| 1111 | Wuzhou | Longxu District | Fabaceae | *Erythrophleum fordii* Oliv | 1300 | 18.5 | 134.4 | 27.0 | Villages and farmlands | Good |
| 1112 | Wuzhou | Teng County | Fabaceae | *Erythrophleum fordii* Oliv. | 1000 | 28.0 | 132.0 | 15.0 | Villages and farmlands | Good |
| 1113 | Wuzhou | Teng County | Fabaceae | *Erythrophleum fordii* Oliv | 1000 | 21.0 | 140.0 | 16.5 | Villages and farmlands | Good |
| 1114 | Wuzhou | Teng County | Fabaceae | *Erythrophleum fordii* Oliv. | 1000 | 15.0 | 166.0 | 10.0 | Villages and farmlands | Good |
| 1115 | Wuzhou | Teng County | Moraceae | *Ficus virens* Aiton. | 1000 | 22.0 | 360.0 | 20.0 | Villages and farmlands | Good |
| 1116 | Wuzhou | Mengshan County | Moraceae | *Ficus concinna* (Miq.) Miq. | 1050 | 23.5 | 400.0 | 52.2 | Residential districts | Good |
| 1117 | Wuzhou | Mengshan County | Moraceae | *Ficus concinna* (Miq.) Miq. | 1000 | 17.0 | 305.7 | 26.5 | Villages and farmlands | Good |
| 1118 | Wuzhou | Mengshan County | Moraceae | *Ficus concinna* (Miq.) Miq. | 1000 | 18.0 | 309.8 | 34.0 | Villages and farmlands | Good |
| 1119 | Wuzhou | Mengshan County | Moraceae | *Ficus concinna* (Miq.) Miq. | 1020 | 14.0 | 377.3 | 15.0 | Villages and farmlands | Good |
| 1120 | Wuzhou | Mengshan County | Moraceae | *Ficus concinna* (Miq.) Miq. | 1200 | 22.0 | 433.0 | 44.0 | Villages and farmlands | Good |
| 1121 | Wuzhou | Mengshan County | Moraceae | *Ficus concinna* (Miq.) Miq. | 1100 | 18.0 | 350.3 | 37.5 | Villages and farmlands | Good |
| 1122 | Wuzhou | Mengshan County | Moraceae | *Ficus concinna* (Miq.) Miq. | 1000 | 28.0 | 327.0 | 36.9 | Villages and farmlands | Good |
| 1123 | Wuzhou | Mengshan County | Moraceae | *Ficus concinna* (Miq.) Miq. | 1000 | 20.0 | 337.5 | 37.0 | Villages and farmlands | Good |
| 1124 | Wuzhou | Mengshan County | Moraceae | *Ficus concinna* (Miq.) Miq. | 1000 | 24.0 | 326.4 | 43.5 | Villages and farmlands | Good |
| 1125 | Wuzhou | Mengshan County | Moraceae | *Ficus concinna* (Miq.) Miq. | 1050 | 18.0 | 398.1 | 35.0 | Villages and farmlands | Good |
| 1126 | Wuzhou | Mengshan County | Moraceae | *Ficus concinna* (Miq.) Miq. | 1000 | 15.0 | 278.0 | 35.5 | Villages and farmlands | Good |
| 1127 | Wuzhou | Mengshan County | Moraceae | *Ficus concinna* (Miq.) Miq. | 1000 | 18.0 | 324.2 | 38.0 | Villages and farmlands | Good |
| 1128 | Wuzhou | Mengshan County | Moraceae | *Ficus concinna* (Miq.) Miq. | 1000 | 17.0 | 312.1 | 34.0 | Villages and farmlands | Good |
| 1129 | Wuzhou | Mengshan County | Moraceae | *Ficus concinna* (Miq.) Miq. | 1000 | 18.0 | 312.1 | 42.0 | Villages and farmlands | Good |
| 1130 | Wuzhou | Cenxi City | Phyllanthaceae | *Bischofia javanica* Blume. | 1000 | 16.0 | 256.0 | 17.8 | Villages and farmlands | Good |
| 1131 | Wuzhou | Cenxi City | Fabaceae | *Erythrophleum fordii* Oliv. | 1200 | 26.5 | 143.0 | 23.8 | Villages and farmlands | Good |
| 1132 | Wuzhou | Cenxi City | Moraceae | *Ficus concinna* (Miq.) Miq. | 1100 | 40.0 | 351.0 | 35.8 | Villages and farmlands | Good |
| 1133 | Wuzhou | Wanxiu District | Lauraceae | *Camphora officinarum Nees.* | 600 | 24.3 | 254.0 | 28.5 | Villages and farmlands | Good |
| 1134 | Wuzhou | Wanxiu District | Moraceae | *Ficus microcarpa* L. f. | 500 | 32.0 | 255.0 | 28.2 | Villages and farmlands | Good |
| 1135 | Wuzhou | Wanxiu District | Moraceae | *Ficus concinna (*Miq.) Miq. | 800 | 15.5 | 312.0 | 33.4 | Villages and farmlands | Good |
| 1136 | Wuzhou | Wanxiu District | Moraceae | *Ficus virens* Aiton. | 500 | 14.8 | 337.0 | 13.0 | Villages and farmlands | Good |
| 1137 | Wuzhou | Changzhou District | Moraceae | *Ficus microcarpa* L. f. | 750 | 20.0 | 264.0 | 32.5 | Villages and farmlands | Good |
| 1138 | Wuzhou | Changzhou District | Oleaceae | *Osmanthus fragrans* (Thunb.) Lour. | 500 | 8.0 | 85.0 | 10.5 | Villages and farmlands | Good |
| 1139 | Wuzhou | Changzhou District | Lauraceae | *Camphora officinarum Nees.* | 550 | 17.0 | 239.0 | 25.0 | Villages and farmlands | Good |
| 1140 | Wuzhou | Longxu District | Moraceae | *Ficus virens* Aiton. | 510 | 23.0 | 280.0 | 28.0 | Residential districts | Poor |
| 1141 | Wuzhou | Longxu District | Lauraceae | *Camphora officinarum Nees.* | 500 | 20.5 | 215.0 | 20.7 | Villages and farmlands | Moderate |
| 1142 | Wuzhou | Longxu District | Fagaceae | *Castanopsis hystrix* Hook. f. & Thomson ex A. DC. | 500 | 19.0 | 136.9 | 9.0 | Villages and farmlands | Good |
| 1143 | Wuzhou | Longxu District | Lauraceae | *Camphora officinarum Nees.* | 800 | 21.0 | 287.0 | 22.0 | Villages and farmlands | Good |
| 1144 | Wuzhou | Longxu District | Lauraceae | *Camphora officinarum Nees.* | 500 | 20.0 | 223.0 | 23.0 | Villages and farmlands | Good |
| 1145 | Wuzhou | Longxu District | Moraceae | *Ficus microcarpa* L. f. | 580 | 15.0 | 270.0 | 35.6 | Villages and farmlands | Good |
| 1146 | Wuzhou | Longxu District | Fagaceae | *Castanopsis hystrix* Hook. f. & Thomson ex A. DC. | 500 | 21.0 | 136.9 | 29.0 | Villages and farmlands | Good |
| 1147 | Wuzhou | Longxu District | Moraceae | *Ficus microcarpa* L. f. | 530 | 22.0 | 261.0 | 33.5 | Villages and farmlands | Good |
| 1148 | Wuzhou | Longxu District | Lauraceae | *Camphora officinarum Nees.* | 508 | 28.0 | 213.0 | 34.0 | Villages and farmlands | Good |
| 1149 | Wuzhou | Cangwu County | Oxalidaceae | *Averrhoa carambo*la L. | 500 | 11.5 | 89.8 | 8.5 | Villages and farmlands | Good |
| 1150 | Wuzhou | Cangwu County | Fagaceae | *Castanopsis chinensis* (Spreng.) Hance | 600 | 18.5 | 157.0 | 19.5 | Villages and farmlands | Moderate |
| 1151 | Wuzhou | Cangwu County | Oxalidaceae | *Averrhoa carambola* L. | 500 | 16.3 | 105.0 | 12.1 | Villages and farmlands | Good |
| 1152 | Wuzhou | Cangwu County | Fagaceae | *Castanopsis hystrix* Hook. f. & Thomson ex A. DC. | 600 | 18.5 | 168.7 | 29.5 | Villages and farmlands | Good |
| 1153 | Wuzhou | Cangwu County | Myrtaceae | *Syzygium euonymifolium* (F. P. Metcalf) Merr. & L. M. Perry | 500 | 18.0 | 146.0 | 16.0 | Villages and farmlands | Good |
| 1154 | Wuzhou | Cangwu County | Theaceae | *Schima superba* Gardner & Champ. | 500 | 24.1 | 121.1 | 21.5 | Villages and farmlands | Good |
| 1155 | Wuzhou | Cangwu County | Fagaceae | *Castanopsis hystrix* Hook. f. & Thomson ex A. DC. | 600 | 26.0 | 153.0 | 17.0 | Villages and farmlands | Good |
| 1156 | Wuzhou | Cangwu County | Fagaceae | *Castanopsis hystrix* Hook. f. & Thomson ex A. DC. | 550 | 23.5 | 143.0 | 10.0 | Villages and farmlands | Good |
| 1157 | Wuzhou | Cangwu County | Fagaceae | *Castanopsis hystrix* Hook. f. & Thomson ex A. DC. | 530 | 24.0 | 141.7 | 17.0 | Villages and farmlands | Good |
| 1158 | Wuzhou | Cangwu County | Fagaceae | *Castanopsis hystrix* Hook. f. & Thomson ex A. DC. | 550 | 18.0 | 140.0 | 13.5 | Villages and farmlands | Good |
| 1159 | Wuzhou | Cangwu County | Fagaceae | *Castanopsis hystrix* Hook. f. & Thomson ex A. DC. | 550 | 12.0 | 142.8 | 13.0 | Villages and farmlands | Good |
| 1160 | Wuzhou | Teng County | Sapindaceae | *Dimocarpus longan* Lour. | 900 | 11.8 | 133.0 | 12.8 | Villages and farmlands | Good |
| 1161 | Wuzhou | Teng County | Lauraceae | *Camphora officinarum Nees* | 520 | 8.0 | 240.0 | 11.0 | Villages and farmlands | Good |
| 1162 | Wuzhou | Teng County | Sapindaceae | *Dimocarpus longan* Lour. | 650 | 14.0 | 121.7 | 14.5 | Villages and farmlands | Moderate |
| 1163 | Wuzhou | Teng County | Sapindaceae | *Dimocarpus longan* Lour. | 800 | 11.0 | 130.0 | 9.0 | Villages and farmlands | Good |
| 1164 | Wuzhou | Teng County | Sapindaceae | *Dimocarpus longan* Lour. | 500 | 13.0 | 110.0 | 10.5 | Villages and farmlands | Good |
| 1165 | Wuzhou | Teng County | Moraceae | *Ficus racemosa* L. | 680 | 21.0 | 280.0 | 12.5 | Villages and farmlands | Good |
| 1166 | Wuzhou | Teng County | Lauraceae | *Camphora officinarum Nees.* | 500 | 20.0 | 220.0 | 21.0 | Villages and farmlands | Good |
| 1167 | Wuzhou | Teng County | Sapindaceae | *Dimocarpus longan* Lour. | 730 | 9.0 | 125.0 | 9.0 | Villages and farmlands | Good |
| 1168 | Wuzhou | Teng County | Lauraceae | *Camphora officinarum Nees.* | 550 | 20.0 | 220.0 | 21.0 | Villages and farmlands | Good |
| 1169 | Wuzhou | Teng County | Moraceae | *Ficus microcarpa* L. f. | 650 | 25.0 | 282.0 | 32.5 | Villages and farmlands | Good |
| 1170 | Wuzhou | Teng County | Lauraceae | *Camphora officinarum Nees.* | 700 | 28.0 | 280.0 | 30.0 | Villages and farmlands | Good |
| 1171 | Wuzhou | Teng County | Moraceae | *Ficus microcarpa* L. f. | 600 | 25.0 | 273.0 | 30.0 | Villages and farmlands | Good |
| 1172 | Wuzhou | Teng County | Moraceae | *Ficus microcarpa* L. f. | 700 | 21.0 | 310.0 | 40.0 | Villages and farmlands | Good |
| 1173 | Wuzhou | Teng County | Moraceae | *Ficus microcarpa* L. f. | 700 | 18.0 | 350.0 | 33.5 | Villages and farmlands | Good |
| 1174 | Wuzhou | Teng County | Moraceae | *Ficus microcarpa* L. f. | 580 | 20.0 | 270.7 | 35.0 | Villages and farmlands | Good |
| 1175 | Wuzhou | Teng County | Moraceae | *Ficus microcarpa* L. f. | 500 | 21.0 | 301.0 | 22.0 | Villages and farmlands | Good |
| 1176 | Wuzhou | Teng County | Sapindaceae | *Dimocarpus longan* Lour. | 650 | 9.0 | 120.8 | 7.0 | Villages and farmlands | Good |
| 1177 | Wuzhou | Teng County | Sapindaceae | *Dimocarpus longan* Lour. | 570 | 13.0 | 115.0 | 14.5 | Villages and farmlands | Good |
| 1178 | Wuzhou | Teng County | Sapindaceae | *Dimocarpus longan* Lour. | 750 | 15.0 | 127.0 | 17.0 | Villages and farmlands | Good |
| 1179 | Wuzhou | Teng County | Moraceae | *Ficus microcarpa* L. f. | 500 | 17.0 | 254.0 | 25.0 | Villages and farmlands | Good |
| 1180 | Wuzhou | Teng County | Lauraceae | *Camphora officinarum Nees.* | 500 | 18.0 | 220.0 | 20.0 | Villages and farmlands | Good |
| 1181 | Wuzhou | Teng County | Moraceae | *Ficus microcarpa* L. f. | 600 | 16.0 | 277.0 | 12.0 | Villages and farmlands | Good |
| 1182 | Wuzhou | Teng County | Lauraceae | *Camphora officinarum Nees.* | 700 | 18.0 | 273.0 | 21.0 | Villages and farmlands | Good |
| 1183 | Wuzhou | Teng County | Fabaceae | *Erythrophleum fordii* Oliv. | 500 | 25.0 | 114.5 | 22.0 | Villages and farmlands | Good |
| 1184 | Wuzhou | Teng County | Lauraceae | *Camphora officinarum Nees.* | 600 | 30.0 | 250.0 | 28.5 | Villages and farmlands | Good |
| 1185 | Wuzhou | Teng County | Lauraceae | *Camphora officinarum Nees.* | 600 | 25.0 | 220.0 | 13.5 | Villages and farmlands | Good |
| 1186 | Wuzhou | Teng County | Lauraceae | *Camphora officinarum Nees.* | 500 | 30.0 | 165.0 | 16.5 | Villages and farmlands | Good |
| 1187 | Wuzhou | Teng County | Fabaceae | *Erythrophleum fordii* Oliv. | 890 | 32.0 | 122.0 | 26.5 | Villages and farmlands | Good |
| 1188 | Wuzhou | Teng County | Lauraceae | *Camphora officinarum Nees.* | 520 | 18.0 | 110.0 | 14.0 | Villages and farmlands | Good |
| 1189 | Wuzhou | Teng County | Lauraceae | *Camphora officinarum Nees.* | 630 | 20.0 | 258.0 | 28.0 | Villages and farmlands | Good |
| 1190 | Wuzhou | Teng County | Fagaceae | *Castanopsis hystrix* Hook. f. & Thomson ex A. DC. | 570 | 28.2 | 151.0 | 24.5 | Villages and farmlands | Good |
| 1191 | Wuzhou | Teng County | Fagaceae | *Castanopsis hystrix* Hook. f. & Thomson ex A. DC. | 540 | 25.0 | 145.0 | 14.0 | Villages and farmlands | Good |
| 1192 | Wuzhou | Teng County | Fabaceae | *Erythrophleum fordii* Oliv. | 750 | 23.0 | 120.0 | 22.5 | Villages and farmlands | Good |
| 1193 | Wuzhou | Teng County | Moraceae | *Ficus microcarpa* L. f. | 600 | 21.5 | 277.0 | 23.5 | Villages and farmlands | Good |
| 1194 | Wuzhou | Teng County | Podocarpaceae | *Dacrycarpus imbricatus* (Blume) de Laub. | 530 | 17.0 | 108.0 | 14.5 | Villages and farmlands | Good |
| 1195 | Wuzhou | Teng County | Podocarpaceae | *Dacrycarpus imbricatus* (Blume) de Laub. | 600 | 28.0 | 115.0 | 23.0 | Villages and farmlands | Good |
| 1196 | Wuzhou | Mengshan County | Fagaceae | *Castanopsis hystrix* Hook. f. & Thomson ex A. DC. | 510 | 30.0 | 140.0 | 30.5 | Villages and farmlands | Good |
| 1197 | Wuzhou | Mengshan County | Fagaceae | *Castanopsis hystrix* Hook. f. & Thomson ex A. DC. | 500 | 30.0 | 140.1 | 16.5 | Villages and farmlands | Good |
| 1198 | Wuzhou | Mengshan County | Fagaceae | *Castanopsis hystrix* Hook. f. & Thomson ex A. DC. | 520 | 19.0 | 149.7 | 7.5 | Villages and farmlands | Good |
| 1199 | Wuzhou | Mengshan County | Moraceae | *Ficus concinna (*Miq.) Miq. | 500 | 16.0 | 262.7 | 29.0 | Villages and farmlands | Good |
| 1200 | Wuzhou | Mengshan County | Moraceae | *Ficus concinna (*Miq.) Miq. | 800 | 15.0 | 299.3 | 18.0 | Villages and farmlands | Good |
| 1201 | Wuzhou | Mengshan County | Moraceae | *Ficus concinna (*Miq.) Miq. | 550 | 19.0 | 267.5 | 31.0 | Villages and farmlands | Good |
| 1202 | Wuzhou | Mengshan County | Moraceae | *Ficus concinna (*Miq.) Miq. | 520 | 19.0 | 261.0 | 40.0 | Villages and farmlands | Good |
| 1203 | Wuzhou | Mengshan County | Moraceae | *Ficus concinna (*Miq.) Miq. | 640 | 16.0 | 280.2 | 35.0 | Villages and farmlands | Good |
| 1204 | Wuzhou | Mengshan County | Moraceae | *Ficus concinna (*Miq.) Miq. | 610 | 15.0 | 277.1 | 29.0 | Villages and farmlands | Good |
| 1205 | Wuzhou | Mengshan County | Moraceae | *Ficus concinna (*Miq.) Miq. | 770 | 16.0 | 296.2 | 25.5 | Villages and farmlands | Good |
| 1206 | Wuzhou | Mengshan County | Fagaceae | *Castanopsis hystrix* Hook. f. & Thomson ex A. DC. | 500 | 18.0 | 140.1 | 22.5 | Villages and farmlands | Good |
| 1207 | Wuzhou | Mengshan County | Podocarpaceae | *Dacrycarpus imbricatus* (Blume) de Laub. | 720 | 37.0 | 127.4 | 20.0 | Villages and farmlands | Good |
| 1208 | Wuzhou | Mengshan County | Fagaceae | *Castanopsis hystrix* Hook. f. & Thomson ex A. DC. | 830 | 28.0 | 192.4 | 18.0 | Villages and farmlands | Good |
| 1209 | Wuzhou | Mengshan County | Fagaceae | *Castanopsis hystrix* Hook. f. & Thomson ex A. DC. | 500 | 16.0 | 136.9 | 16.1 | Villages and farmlands | Good |
| 1210 | Wuzhou | Mengshan County | Fagaceae | *Castanopsis hystrix* Hook. f. & Thomson ex A. DC. | 500 | 19.0 | 143.3 | 18.0 | Villages and farmlands | Good |
| 1211 | Wuzhou | Mengshan County | Fagaceae | *Castanopsis hystrix* Hook. f. & Thomson ex A. DC. | 500 | 21.0 | 136.9 | 16.5 | Villages and farmlands | Good |
| 1212 | Wuzhou | Cenxi City | Moraceae | *Ficus virens* Aiton. | 800 | 18.5 | 366.2 | 34.5 | Villages and farmlands | Good |
| 1213 | Wuzhou | Cenxi City | Moraceae | *Ficus virens* Aiton. | 500 | 29.0 | 274.7 | 30.6 | Villages and farmlands | Good |
| 1214 | Wuzhou | Cenxi City | Moraceae | *Ficus concinna (*Miq.) Miq. | 550 | 11.5 | 267.5 | 23.0 | Villages and farmlands | Good |
| 1215 | Wuzhou | Cenxi City | Oxalidaceae | *Averrhoa carambola* L. | 500 | 14.0 | 92.3 | 16.0 | Villages and farmlands | Good |
| 1216 | Wuzhou | Cenxi City | Sapindaceae | *Dimocarpus longan* Lour. | 550 | 9.0 | 114.6 | 15.0 | Villages and farmlands | Good |
| 1217 | Wuzhou | Cenxi City | Moraceae | *Ficus microcarpa* L. f. | 650 | 25.0 | 287.0 | 40.0 | Villages and farmlands | Good |
| 1218 | Wuzhou | Cenxi City | Sapindaceae | *Dimocarpus longan* Lour. | 520 | 19.0 | 111.0 | 7.5 | Villages and farmlands | Good |
| 1219 | Wuzhou | Cenxi City | Ebenaceae | *Diospyros eriantha* Champion ex Bentham | 500 | 12.0 | 102.0 | 9.0 | Villages and farmlands | Good |
| 1220 | Wuzhou | Cenxi City | Moraceae | *Ficus microcarpa* L. f. | 700 | 30.0 | 292.0 | 39.0 | Villages and farmlands | Good |
| 1221 | Wuzhou | Cenxi City | Theaceae | *Schima superba* Gardner & Champ. | 550 | 17.5 | 124.0 | 13.0 | Villages and farmlands | Good |
| 1222 | Wuzhou | Cenxi City | Moraceae | *Ficus microcarpa* L. f. | 600 | 27.0 | 275.0 | 35.0 | Villages and farmlands | Good |
| 1223 | Wuzhou | Cenxi City | Sapindaceae | *Dimocarpus longan* Lour. | 500 | 15.7 | 109.5 | 16.6 | Villages and farmlands | Good |
| 1224 | Wuzhou | Cenxi City | Lauraceae | *Camphora officinarum Nees.* | 600 | 20.0 | 245.0 | 28.3 | Villages and farmlands | Good |
| 1225 | Wuzhou | Cenxi City | Sapindaceae | *Dimocarpus longan* Lour. | 650 | 10.0 | 121.0 | 17.5 | Villages and farmlands | Good |
| 1226 | Wuzhou | Cenxi City | Theaceae | *Schima superba* Gardner & Champ. | 600 | 28.0 | 127.3 | 14.5 | Villages and farmlands | Good |
| 1227 | Wuzhou | Cenxi City | Ericaceae | *Craibiodendron stellatum* (Pierre) W. W. Sm. | 500 | 30.0 | 113.0 | 13.5 | Villages and farmlands | Good |
| 1228 | Wuzhou | Cenxi City | Fagaceae | *Castanopsis hystrix* Hook. f. & Thomson ex A. DC. | 580 | 26.0 | 152.0 | 17.5 | Villages and farmlands | Good |
| 1229 | Wuzhou | Cenxi City | Fabaceae | *Erythrophleum fordii* Oliv. | 500 | 26.0 | 113.0 | 23.5 | Villages and farmlands | Good |
| 1230 | Beihai | Yinhai District | Sapotaceae | *Pouteria annamensis* (Pierre) Baehni | 500 | 37.2 | 98.7 | 12.5 | Villages and farmlands | Good |
| 1231 | Beihai | Tieshan Port District | Moraceae | *Ficus altissima* Blume | 600 | 22.0 | 287.0 | 22.0 | Villages and farmlands | Good |
| 1232 | Beihai | Tieshan Port District | Sapotaceae | *Manilkara hexandra* (Roxb.) Dubard | 500 | 15.0 | 124.0 | 14.5 | Villages and farmlands | Good |
| 1233 | Beihai | Tieshan Port District | Moraceae | *Antiaris toxicaria* Lesch. | 550 | 28.0 | 250.0 | 22.0 | Villages and farmlands | Good |
| 1234 | Beihai | Hepu County | Moraceae | *Ficus microcarpa* L. f. | 500 | 20.2 | 277.0 | 34.0 | Residential districts | Good |
| 1235 | Beihai | Hepu County | Fabaceae | *Erythrophleum fordii* Oliv. | 700 | 24.5 | 126.0 | 22.1 | Villages and farmlands | Good |
| 1236 | Beihai | Hepu County | Moraceae | *Antiaris toxicaria* Lesch. | 512 | 30.0 | 166.6 | 29.0 | Villages and farmlands | Good |
| 1237 | Beihai | Hepu County | Lauraceae | *Camphora officinarum Nees.* | 500 | 21.5 | 217.8 | 21.8 | Villages and farmlands | Poor |
| 1238 | Fangchenggang | Fangcheng District | Moraceae | *Ficus altissima* Blume. | 1000 | 25.0 | 439.0 | 38.0 | Villages and farmlands | Poor |
| 1239 | Fangchenggang | Fangcheng District | Podocarpaceae | *Dacrycarpus imbricatus* (Blume) de Laub. | 600 | 22.0 | 110.0 | 16.0 | Villages and farmlands | Moderate |
| 1240 | Fangchenggang | Fangcheng District | Fabaceae | *Ormosia pubescens* R. H. Chang | 500 | 19.0 | 150.0 | 20.0 | Villages and farmlands | Good |
| 1241 | Fangchenggang | Shangsi County | Oxalidaceae | *Averrhoa carambola* L. | 610 | 14.5 | 95.5 | 7.3 | Villages and farmlands | Good |
| 1242 | Fangchenggang | Shangsi County | Lauraceae | *Camphora officinarum Nees.* | 500 | 22.8 | 221.2 | 15.2 | Villages and farmlands | Good |
| 1243 | Fangchenggang | Shangsi County | Fabaceae | *Erythrophleum fordii* Oliv. | 500 | 18.6 | 114.6 | 21.3 | Villages and farmlands | Good |
| 1244 | Fangchenggang | Shangsi County | Moraceae | *Ficus altissima* Blume. | 500 | 27.1 | 286.2 | 52.9 | Villages and farmlands | Good |
| 1245 | Fangchenggang | Shangsi County | Moraceae | *Ficus altissima* Blume. | 650 | 24.8 | 321.1 | 59.5 | Villages and farmlands | Good |
| 1246 | Fangchenggang | Shangsi County | Moraceae | *Ficus altissima* Blume. | 550 | 16.0 | 280.0 | 23.5 | Villages and farmlands | Good |
| 1247 | Fangchenggang | Dongxing City | Ebenaceae | *Diospyros decandra* Lour. | 500 | 18.0 | 113.3 | 15.0 | Villages and farmlands | Moderate |
| 1248 | Fangchenggang | Dongxing City | Ebenaceae | *Diospyros decandra* Lour. | 500 | 16.0 | 106.6 | 14.0 | Villages and farmlands | Moderate |
| 1249 | Fangchenggang | Dongxing City | Ebenaceae | *Diospyros decandra* Lour. | 500 | 16.0 | 111.7 | 16.0 | Villages and farmlands | Moderate |
| 1250 | Fangchenggang | Dongxing City | Ebenaceae | *Diospyros decandra* Lour. | 500 | 19.0 | 113.3 | 14.5 | Villages and farmlands | Moderate |
| 1251 | Fangchenggang | Dongxing City | Ebenaceae | *Diospyros decandra* Lour. | 500 | 18.0 | 124.1 | 13.0 | Villages and farmlands | Moderate |
| 1252 | Qinzhou | Qinbei District | Moraceae | *Ficus virens* Aiton. | 1005 | 22.5 | 301.4 | 29.3 | Residential districts | Poor |
| 1253 | Qinzhou | Qinbei District | Fabaceae | *Erythrophleum fordii* Oliv. | 1000 | 26.7 | 134.3 | 19.0 | Villages and farmlands | Poor |
| 1254 | Qinzhou | Lingshan County | Sapindaceae | *Litchi chinensis* Sonn. | 1100 | 8.3 | 191.0 | 14.4 | Villages and farmlands | Good |
| 1255 | Qinzhou | Lingshan County | Sapindaceae | *Litchi chinensis* Sonn. | 1556 | 14.5 | 177.0 | 15.4 | Villages and farmlands | Good |
| 1256 | Qinzhou | Lingshan County | Sapindaceae | *Litchi chinensis* Sonn. | 1500 | 7.2 | 173.0 | 6.8 | Villages and farmlands | Good |
| 1257 | Qinzhou | Lingshan County | Sapindaceae | *Litchi chinensis* Sonn. | 1500 | 5.5 | 169.0 | 5.3 | Villages and farmlands | Good |
| 1258 | Qinzhou | Lingshan County | Sapindaceae | *Litchi chinensis* Sonn. | 1300 | 8.2 | 170.0 | 13.4 | Villages and farmlands | Good |
| 1259 | Qinzhou | Lingshan County | Sapindaceae | *Litchi chinensis* Sonn. | 1500 | 21.5 | 195.0 | 18.2 | Villages and farmlands | Good |
| 1260 | Qinzhou | Lingshan County | Sapindaceae | *Litchi chinensis* Sonn. | 1200 | 10.1 | 156.0 | 18.5 | Villages and farmlands | Good |
| 1261 | Qinzhou | Lingshan County | Sapindaceae | *Litchi chinensis* Sonn. | 1500 | 8.5 | 156.0 | 14.7 | Villages and farmlands | Good |
| 1262 | Qinzhou | Lingshan County | Sapindaceae | *Litchi chinensis* Sonn. | 1000 | 6.8 | 286.0 | 18.1 | Villages and farmlands | Good |
| 1263 | Qinzhou | Pubei County | Fabaceae | *Erythrophleum fordii* Oliv. | 1300 | 14.0 | 143.3 | 22.0 | Villages and farmlands | Good |
| 1264 | Qinzhou | Pubei County | Fabaceae | *Erythrophleum fordii* Oliv. | 1200 | 35.0 | 136.9 | 29.0 | Villages and farmlands | Good |
| 1265 | Qinzhou | Pubei County | Aquifoliaceae | *Ilex rotunda* Thunb. | 1000 | 20.0 | 107.0 | 18.5 | Villages and farmlands | Good |
| 1266 | Qinzhou | Pubei County | Podocarpaceae | *Dacrycarpus imbricatus* (Blume) de Laub. | 1000 | 25.0 | 126.0 | 20.0 | Villages and farmlands | Good |
| 1267 | Qinzhou | Pubei County | Moraceae | *Ficus concinna* (Miq.) Miq. | 1300 | 30.0 | 513.0 | 48.0 | Villages and farmlands | Good |
| 1268 | Qinzhou | Qinnan District | Moraceae | *Ficus altissima* Blume. | 600 | 33.0 | 133.0 | 31.5 | Villages and farmlands | Good |
| 1269 | Qinzhou | Qinnan District | Moraceae | *Ficus altissima* Blume. | 950 | 16.0 | 330.0 | 33.5 | Villages and farmlands | Good |
| 1270 | Qinzhou | Qinbei District | Moraceae | *Ficus microcarpa* L. f. | 580 | 24.1 | 175.1 | 24.0 | Residential districts | Good |
| 1271 | Qinzhou | Qinbei District | Moraceae | *Ficus microcarpa* L. f. | 520 | 23.1 | 208.5 | 35.4 | Villages and farmlands | Good |
| 1272 | Qinzhou | Qinbei District | Moraceae | *Ficus altissima* Blume. | 510 | 15.1 | 273.7 | 33.1 | Villages and farmlands | Moderate |
| 1273 | Qinzhou | Qinbei District | Moraceae | *Ficus microcarpa* L. f. | 600 | 24.3 | 165.5 | 22.0 | Villages and farmlands | Good |
| 1274 | Qinzhou | Qinbei District | Moraceae | *Ficus altissima* Blume. | 505 | 25.8 | 154.4 | 9.9 | Villages and farmlands | Poor |
| 1275 | Qinzhou | Qinbei District | Moraceae | *Artocarpus parvus* Gagnep. | 600 | 7.8 | 118.1 | 8.5 | Villages and farmlands | Moderate |
| 1276 | Qinzhou | Qinbei District | Myrtaceae | *Syzygium levinei* (Merr.) Merr. & L. M. Perry | 600 | 20.8 | 106.0 | 16.1 | Villages and farmlands | Poor |
| 1277 | Qinzhou | Qinbei District | Moraceae | *Ficus microcarpa* L. f. | 500 | 22.4 | 260.1 | 23.4 | Villages and farmlands | Good |
| 1278 | Qinzhou | Qinbei District | Moraceae | *Ficus microcarpa* L. f. | 500 | 11.8 | 98.7 | 20.8 | Villages and farmlands | Good |
| 1279 | Qinzhou | Qinbei District | Moraceae | *Ficus altissima* Blume. | 500 | 14.5 | 198.5 | 27.8 | Villages and farmlands | Poor |
| 1280 | Qinzhou | Qinbei District | Moraceae | *Ficus altissima* Blume. | 500 | 13.4 | 130.0 | 29.9 | Villages and farmlands | Poor |
| 1281 | Qinzhou | Qinbei District | Moraceae | *Ficus altissima* Blume. | 650 | 20.1 | 292.8 | 32.9 | Villages and farmlands | Poor |
| 1282 | Qinzhou | Qinbei District | Moraceae | *Ficus altissima* Blume. | 560 | 19.8 | 195.8 | 26.9 | Villages and farmlands | Poor |
| 1283 | Qinzhou | Qinbei District | Moraceae | *Ficus altissima* Blume. | 500 | 13.6 | 279.5 | 33.0 | Villages and farmlands | Poor |
| 1284 | Qinzhou | Qinbei District | Moraceae | *Ficus microcarpa* L. f. | 700 | 18.0 | 191.3 | 28.1 | Villages and farmlands | Good |
| 1285 | Qinzhou | Qinbei District | Polygalaceae | *Xanthophyllum hainanense* Hu. | 500 | 8.6 | 76.5 | 10.6 | Villages and farmlands | Poor |
| 1286 | Qinzhou | Lingshan County | Sapindaceae | *Litchi chinensis* Sonn. | 500 | 12.2 | 185.0 | 16.1 | Villages and farmlands | Good |
| 1287 | Qinzhou | Lingshan County | Sapindaceae | *Litchi chinensis* Sonn. | 650 | 8.5 | 203.8 | 7.1 | Villages and farmlands | Good |
| 1288 | Qinzhou | Lingshan County | Sapindaceae | *Litchi chinensis* Sonn. | 560 | 8.8 | 191.0 | 16.1 | Villages and farmlands | Good |
| 1289 | Qinzhou | Lingshan County | Moraceae | *Ficus microcarpa* L. f. | 720 | 17.2 | 75.0 | 23.6 | Villages and farmlands | Good |
| 1290 | Qinzhou | Lingshan County | Moraceae | *Ficus microcarpa* L. f. | 520 | 15.5 | 78.0 | 13.8 | Villages and farmlands | Good |
| 1291 | Qinzhou | Lingshan County | Moraceae | *Ficus microcarpa* L. f. | 870 | 15.1 | 285.0 | 24.2 | Villages and farmlands | Good |
| 1292 | Qinzhou | Lingshan County | Moraceae | *Ficus microcarpa* L. f. | 920 | 17.3 | 182.0 | 15.7 | Villages and farmlands | Good |
| 1293 | Qinzhou | Lingshan County | Moraceae | *Ficus microcarpa* L. f. | 920 | 9.1 | 152.0 | 17.2 | Villages and farmlands | Good |
| 1294 | Qinzhou | Lingshan County | Moraceae | *Ficus microcarpa* L. f. | 820 | 22.1 | 192.0 | 25.3 | Villages and farmlands | Good |
| 1295 | Qinzhou | Lingshan County | Sapindaceae | *Litchi chinensis* Sonn. | 550 | 12.5 | 191.1 | 8.7 | Villages and farmlands | Good |
| 1296 | Qinzhou | Lingshan County | Sapindaceae | *Litchi chinensis* Sonn. | 530 | 9.5 | 152.0 | 5.6 | Villages and farmlands | Good |
| 1297 | Qinzhou | Lingshan County | Sapindaceae | *Litchi chinensis* Sonn. | 530 | 11.2 | 102.0 | 5.4 | Villages and farmlands | Good |
| 1298 | Qinzhou | Lingshan County | Sapindaceae | *Litchi chinensis* Sonn. | 500 | 9.2 | 102.3 | 15.4 | Villages and farmlands | Good |
| 1299 | Qinzhou | Lingshan County | Sapindaceae | *Litchi chinensis* Sonn. | 600 | 10.5 | 118.6 | 15.6 | Villages and farmlands | Moderate |
| 1300 | Qinzhou | Lingshan County | Sapindaceae | *Litchi chinensis* Sonn. | 600 | 10.5 | 145.9 | 16.7 | Villages and farmlands | Moderate |
| 1301 | Qinzhou | Lingshan County | Moraceae | *Ficus microcarpa* L. f. | 550 | 12.7 | 161.9 | 6.9 | Villages and farmlands | Good |
| 1302 | Qinzhou | Lingshan County | Moraceae | *Ficus altissima* Blume | 560 | 26.5 | 287.0 | 33.1 | Villages and farmlands | Good |
| 1303 | Qinzhou | Lingshan County | Moraceae | *Ficus microcarpa* L. f. | 500 | 9.2 | 267.0 | 9.2 | Villages and farmlands | Good |
| 1304 | Qinzhou | Lingshan County | Moraceae | *Ficus microcarpa* L. f. | 500 | 22.2 | 265.0 | 21.6 | Villages and farmlands | Good |
| 1305 | Qinzhou | Lingshan County | Sapindaceae | *Litchi chinensis* Sonn. | 600 | 10.5 | 198.0 | 14.2 | Villages and farmlands | Good |
| 1306 | Qinzhou | Lingshan County | Sapindaceae | *Litchi chinensis* Sonn. | 670 | 6.3 | 208.0 | 11.9 | Villages and farmlands | Good |
| 1307 | Qinzhou | Lingshan County | Sapindaceae | *Litchi chinensis* Sonn. | 755 | 6.7 | 217.0 | 24.2 | Villages and farmlands | Good |
| 1308 | Qinzhou | Lingshan County | Sapindaceae | *Litchi chinensis* Sonn. | 510 | 7.2 | 182.0 | 13.1 | Villages and farmlands | Good |
| 1309 | Qinzhou | Lingshan County | Sapindaceae | *Litchi chinensis* Sonn. | 530 | 6.4 | 186.0 | 15.3 | Villages and farmlands | Good |
| 1310 | Qinzhou | Lingshan County | Sapindaceae | *Litchi chinensis* Sonn. | 590 | 7.4 | 192.0 | 9.0 | Villages and farmlands | Good |
| 1311 | Qinzhou | Lingshan County | Sapindaceae | *Litchi chinensis* Sonn. | 710 | 6.2 | 222.0 | 16.9 | Villages and farmlands | Good |
| 1312 | Qinzhou | Lingshan County | Sapindaceae | *Litchi chinensis* Sonn. | 520 | 8.2 | 186.0 | 19.0 | Villages and farmlands | Good |
| 1313 | Qinzhou | Lingshan County | Sapindaceae | *Litchi chinensis* Sonn. | 800 | 9.2 | 233.0 | 19.2 | Villages and farmlands | Good |
| 1314 | Qinzhou | Lingshan County | Sapindaceae | *Dimocarpus longan* Lour. | 690 | 6.6 | 123.0 | 11.2 | Villages and farmlands | Good |
| 1315 | Qinzhou | Lingshan County | Sapindaceae | *Dimocarpus longan* Lour. | 500 | 6.8 | 80.0 | 15.0 | Villages and farmlands | Good |
| 1316 | Qinzhou | Lingshan County | Sapindaceae | *Litchi chinensis* Sonn. | 540 | 8.6 | 71.6 | 22.2 | Villages and farmlands | Good |
| 1317 | Qinzhou | Lingshan County | Sapindaceae | *Litchi chinensis* Sonn. | 840 | 7.8 | 227.0 | 14.0 | Villages and farmlands | Good |
| 1318 | Qinzhou | Lingshan County | Sapindaceae | *Litchi chinensis* Sonn. | 570 | 7.6 | 192.0 | 14.1 | Villages and farmlands | Good |
| 1319 | Qinzhou | Lingshan County | Sapindaceae | *Litchi chinensis* Sonn. | 500 | 7.8 | 182.0 | 15.7 | Villages and farmlands | Good |
| 1320 | Qinzhou | Lingshan County | Sapindaceae | *Litchi chinensis* Sonn. | 530 | 6.8 | 186.0 | 17.9 | Villages and farmlands | Good |
| 1321 | Qinzhou | Lingshan County | Sapindaceae | *Litchi chinensis* Sonn. | 510 | 6.5 | 183.0 | 15.5 | Villages and farmlands | Good |
| 1322 | Qinzhou | Lingshan County | Sapindaceae | *Litchi chinensis* Sonn. | 590 | 6.5 | 195.0 | 17.9 | Villages and farmlands | Good |
| 1323 | Qinzhou | Lingshan County | Sapindaceae | *Litchi chinensis* Sonn. | 910 | 6.2 | 234.0 | 22.4 | Villages and farmlands | Good |
| 1324 | Qinzhou | Lingshan County | Sapindaceae | *Litchi chinensis* Sonn. | 650 | 7.8 | 206.0 | 15.7 | Villages and farmlands | Moderate |
| 1325 | Qinzhou | Lingshan County | Sapindaceae | *Litchi chinensis* Sonn. | 755 | 8.2 | 216.0 | 14.5 | Villages and farmlands | Moderate |
| 1326 | Qinzhou | Lingshan County | Sapindaceae | *Litchi chinensis* Sonn. | 500 | 8.5 | 220.0 | 16.9 | Villages and farmlands | Moderate |
| 1327 | Qinzhou | Lingshan County | Sapindaceae | *Litchi chinensis* Sonn. | 850 | 6.3 | 242.0 | 16.9 | Villages and farmlands | Moderate |
| 1328 | Qinzhou | Lingshan County | Sapindaceae | *Litchi chinensis* Sonn. | 710 | 6.5 | 214.0 | 20.6 | Villages and farmlands | Moderate |
| 1329 | Qinzhou | Lingshan County | Sapindaceae | *Litchi chinensis* Sonn. | 660 | 8.2 | 206.0 | 17.0 | Villages and farmlands | Moderate |
| 1330 | Qinzhou | Lingshan County | Sapindaceae | *Litchi chinensis* Sonn. | 900 | 6.7 | 244.0 | 14.3 | Villages and farmlands | Good |
| 1331 | Qinzhou | Lingshan County | Sapindaceae | *Litchi chinensis* Sonn. | 700 | 7.6 | 212.0 | 21.0 | Villages and farmlands | Good |
| 1332 | Qinzhou | Lingshan County | Sapindaceae | *Litchi chinensis* Sonn. | 750 | 6.8 | 218.0 | 18.0 | Villages and farmlands | Good |
| 1333 | Qinzhou | Lingshan County | Sapindaceae | *Litchi chinensis* Sonn. | 580 | 8.3 | 195.0 | 16.9 | Villages and farmlands | Good |
| 1334 | Qinzhou | Lingshan County | Sapindaceae | *Litchi chinensis* Sonn. | 610 | 8.2 | 198.0 | 16.8 | Villages and farmlands | Good |
| 1335 | Qinzhou | Lingshan County | Sapindaceae | *Litchi chinensis* Sonn. | 630 | 7.4 | 192.0 | 19.5 | Villages and farmlands | Good |
| 1336 | Qinzhou | Lingshan County | Sapindaceae | *Litchi chinensis* Sonn. | 510 | 6.4 | 183.0 | 10.6 | Villages and farmlands | Good |
| 1337 | Qinzhou | Lingshan County | Sapindaceae | *Litchi chinensis* Sonn. | 720 | 5.4 | 214.0 | 7.1 | Villages and farmlands | Good |
| 1338 | Qinzhou | Lingshan County | Sapindaceae | *Litchi chinensis* Sonn. | 510 | 6.8 | 184.0 | 8.7 | Villages and farmlands | Good |
| 1339 | Qinzhou | Pubei County | Sapindaceae | *Litchi chinensis* Sonn. | 520 | 8.1 | 184.0 | 17.5 | Villages and farmlands | Good |
| 1340 | Qinzhou | Pubei County | Moraceae | *Ficus microcarpa* L. f. | 620 | 16.0 | 120.0 | 19.5 | Villages and farmlands | Good |
| 1341 | Qinzhou | Pubei County | Moraceae | *Antiaris toxicaria* Lesch. | 500 | 20.0 | 200.0 | 11.5 | Villages and farmlands | Moderate |
| 1342 | Qinzhou | Pubei County | Sapindaceae | *Dimocarpus longan* Lour. | 550 | 15.0 | 114.0 | 19.0 | Villages and farmlands | Moderate |
| 1343 | Qinzhou | Pubei County | Moraceae | *Ficus microcarpa* L. f. | 515 | 20.0 | 260.0 | 34.0 | Villages and farmlands | Good |
| 1344 | Qinzhou | Pubei County | Moraceae | *Ficus altissima* Blume | 570 | 14.0 | 269.0 | 14.5 | Villages and farmlands | Good |
| 1345 | Qinzhou | Pubei County | Sapindaceae | *Litchi chinensis* Sonn. | 500 | 21.0 | 89.2 | 16.5 | Residential districts | Good |
| 1346 | Qinzhou | Pubei County | Sapindaceae | *Litchi chinensis* Sonn. | 600 | 6.0 | 71.0 | 13.0 | Villages and farmlands | Good |
| 1347 | Qinzhou | Pubei County | Sapindaceae | *Litchi chinensis* Sonn. | 600 | 6.0 | 60.0 | 10.0 | Villages and farmlands | Good |
| 1348 | Qinzhou | Pubei County | Sapindaceae | *Litchi chinensis* Sonn. | 600 | 8.0 | 89.0 | 11.0 | Villages and farmlands | Good |
| 1349 | Qinzhou | Pubei County | Sapindaceae | *Litchi chinensis* Sonn. | 600 | 7.0 | 82.8 | 6.5 | Villages and farmlands | Good |
| 1350 | Qinzhou | Pubei County | Sapindaceae | *Litchi chinensis* Sonn. | 600 | 8.0 | 53.1 | 14.0 | Villages and farmlands | Good |
| 1351 | Qinzhou | Pubei County | Sapindaceae | *Litchi chinensis* Sonn. | 600 | 8.0 | 52.0 | 14.0 | Villages and farmlands | Good |
| 1352 | Qinzhou | Pubei County | Aquifoliaceae | *Ilex rotunda* Thunb. | 500 | 5.5 | 92.4 | 9.5 | Villages and farmlands | Moderate |
| 1353 | Qinzhou | Pubei County | Myrtaceae | *Syzygium nervosum* DC. | 700 | 9.0 | 178.3 | 14.0 | Villages and farmlands | Good |
| 1354 | Qinzhou | Pubei County | Sapindaceae | *Dimocarpus longan* Lour. | 600 | 14.0 | 92.0 | 14.0 | Villages and farmlands | Good |
| 1355 | Qinzhou | Pubei County | Sapindaceae | *Litchi chinensis* Sonn. | 500 | 9.0 | 96.0 | 11.0 | Villages and farmlands | Good |
| 1356 | Qinzhou | Pubei County | Sapindaceae | *Litchi chinensis* Sonn. | 603 | 13.0 | 199.0 | 14.0 | Villages and farmlands | Good |
| 1357 | Qinzhou | Pubei County | Moraceae | *Ficus microcarpa* L. f. | 520 | 16.0 | 270.7 | 29.0 | Villages and farmlands | Good |
| 1358 | Qinzhou | Pubei County | Fabaceae | *Erythrophleum fordii* Oliv. | 550 | 35.0 | 114.6 | 29.5 | Villages and farmlands | Good |
| 1359 | Qinzhou | Pubei County | Sapindaceae | *Dimocarpus longan* Lour. | 500 | 15.0 | 103.0 | 20.5 | Villages and farmlands | Good |
| 1360 | Qinzhou | Pubei County | Sapindaceae | *Dimocarpus longan* Lour. | 720 | 14.0 | 125.0 | 14.5 | Villages and farmlands | Good |
| 1361 | Qinzhou | Pubei County | Fabaceae | *Erythrophleum fordii* Oliv. | 900 | 20.0 | 125.0 | 18.0 | Villages and farmlands | Good |
| 1362 | Qinzhou | Pubei County | Moraceae | *Ficus virens* Aiton. | 500 | 21.0 | 280.0 | 24.0 | Villages and farmlands | Moderate |
| 1363 | Qinzhou | Pubei County | Theaceae | *Schima superba* Gardner & Champ. | 700 | 30.0 | 134.7 | 11.0 | Villages and farmlands | Good |
| 1364 | Qinzhou | Pubei County | Moraceae | *Ficus virens* Aiton. | 560 | 11.0 | 300.0 | 25.0 | Villages and farmlands | Poor |
| 1365 | Qinzhou | Pubei County | Moraceae | *Ficus virens* Aiton. | 750 | 10.0 | 350.0 | 30.0 | Villages and farmlands | Good |
| 1366 | Qinzhou | Pubei County | Rhamnaceae | *Hovenia acerba* Lindl. | 549 | 9.0 | 95.0 | 11.0 | Villages and farmlands | Good |
| 1367 | Qinzhou | Pubei County | Elaeocarpaceae | *Elaeocarpus sylvestris* (Lour.) Poir. | 500 | 15.0 | 110.0 | 10.0 | Villages and farmlands | Good |
| 1368 | Qinzhou | Pubei County | Sapindaceae | *Litchi chinensis* Sonn. | 800 | 12.0 | 89.1 | 11.0 | Villages and farmlands | Good |
| 1369 | Qinzhou | Pubei County | Sapindaceae | *Litchi chinensis* Sonn. | 800 | 12.0 | 85.9 | 8.5 | Villages and farmlands | Good |
| 1370 | Qinzhou | Pubei County | Sapindaceae | *Litchi chinensis* Sonn. | 800 | 12.0 | 73.2 | 12.0 | Villages and farmlands | Good |
| 1371 | Qinzhou | Pubei County | Sapindaceae | *Litchi chinensis* Sonn. | 800 | 10.0 | 121.0 | 12.0 | Villages and farmlands | Good |
| 1372 | Qinzhou | Pubei County | Sapindaceae | *Litchi chinensis* Sonn. | 800 | 12.0 | 95.5 | 9.5 | Villages and farmlands | Good |
| 1373 | Qinzhou | Pubei County | Sapindaceae | *Litchi chinensis* Sonn. | 800 | 15.0 | 116.2 | 10.5 | Villages and farmlands | Good |
| 1374 | Qinzhou | Pubei County | Sapindaceae | *Litchi chinensis* Sonn. | 800 | 13.0 | 60.5 | 10.5 | Villages and farmlands | Good |
| 1375 | Qinzhou | Pubei County | Sapindaceae | *Litchi chinensis* Sonn. | 800 | 13.0 | 85.9 | 9.0 | Villages and farmlands | Good |
| 1376 | Qinzhou | Pubei County | Sapindaceae | *Litchi chinensis* Sonn. | 800 | 12.0 | 85.9 | 14.0 | Villages and farmlands | Good |
| 1377 | Qinzhou | Pubei County | Sapindaceae | *Litchi chinensis* Sonn. | 800 | 13.0 | 76.4 | 11.5 | Villages and farmlands | Good |
| 1378 | Qinzhou | Pubei County | Sapindaceae | *Litchi chinensis* Sonn. | 800 | 13.0 | 143.3 | 12.0 | Villages and farmlands | Good |
| 1379 | Qinzhou | Pubei County | Sapindaceae | *Litchi chinensis* Sonn. | 800 | 12.0 | 76.4 | 7.5 | Villages and farmlands | Good |
| 1380 | Qinzhou | Pubei County | Sapindaceae | *Litchi chinensis* Sonn. | 800 | 15.0 | 143.3 | 9.0 | Villages and farmlands | Good |
| 1381 | Qinzhou | Pubei County | Sapindaceae | *Litchi chinensis* Sonn. | 800 | 11.0 | 85.9 | 13.0 | Villages and farmlands | Good |
| 1382 | Qinzhou | Pubei County | Sapindaceae | *Litchi chinensis* Sonn. | 800 | 10.0 | 73.2 | 9.0 | Villages and farmlands | Good |
| 1383 | Qinzhou | Pubei County | Sapindaceae | *Litchi chinensis* Sonn. | 800 | 13.0 | 79.6 | 9.5 | Villages and farmlands | Good |
| 1384 | Qinzhou | Pubei County | Sapindaceae | *Litchi chinensis* Sonn. | 800 | 10.0 | 63.7 | 10.0 | Villages and farmlands | Good |
| 1385 | Qinzhou | Pubei County | Sapindaceae | *Litchi chinensis* Sonn. | 800 | 7.0 | 60.0 | 7.0 | Villages and farmlands | Good |
| 1386 | Qinzhou | Pubei County | Sapindaceae | *Litchi chinensis* Sonn. | 800 | 10.0 | 70.0 | 11.0 | Villages and farmlands | Good |
| 1387 | Qinzhou | Pubei County | Sapindaceae | *Litchi chinensis* Sonn. | 800 | 11.0 | 136.9 | 10.0 | Villages and farmlands | Good |
| 1388 | Qinzhou | Pubei County | Sapindaceae | *Litchi chinensis* Sonn. | 800 | 15.0 | 89.1 | 13.5 | Villages and farmlands | Good |
| 1389 | Qinzhou | Pubei County | Sapindaceae | *Litchi chinensis* Sonn. | 800 | 13.0 | 114.6 | 12.0 | Villages and farmlands | Good |
| 1390 | Qinzhou | Pubei County | Sapindaceae | *Litchi chinensis* Sonn. | 800 | 12.0 | 57.3 | 11.0 | Villages and farmlands | Good |
| 1391 | Qinzhou | Pubei County | Sapindaceae | *Litchi chinensis* Sonn. | 800 | 9.0 | 41.4 | 10.0 | Villages and farmlands | Good |
| 1392 | Qinzhou | Pubei County | Sapindaceae | *Litchi chinensis* Sonn. | 800 | 10.0 | 65.0 | 10.0 | Villages and farmlands | Good |
| 1393 | Qinzhou | Pubei County | Sapindaceae | *Litchi chinensis* Sonn. | 800 | 10.0 | 65.2 | 9.5 | Villages and farmlands | Good |
| 1394 | Qinzhou | Pubei County | Sapindaceae | *Litchi chinensis* Sonn. | 800 | 11.0 | 95.5 | 10.5 | Villages and farmlands | Good |
| 1395 | Qinzhou | Pubei County | Sapindaceae | *Litchi chinensis* Sonn. | 800 | 12.0 | 76.4 | 8.5 | Villages and farmlands | Good |
| 1396 | Qinzhou | Pubei County | Sapindaceae | *Litchi chinensis* Sonn. | 800 | 11.0 | 50.9 | 13.0 | Villages and farmlands | Good |
| 1397 | Qinzhou | Pubei County | Sapindaceae | *Litchi chinensis* Sonn. | 800 | 12.0 | 44.6 | 9.0 | Villages and farmlands | Good |
| 1398 | Qinzhou | Pubei County | Sapindaceae | *Litchi chinensis* Sonn. | 800 | 11.0 | 70.0 | 15.0 | Villages and farmlands | Good |
| 1399 | Qinzhou | Pubei County | Sapindaceae | *Litchi chinensis* Sonn. | 800 | 9.0 | 57.3 | 10.0 | Villages and farmlands | Good |
| 1400 | Qinzhou | Pubei County | Sapindaceae | *Litchi chinensis* Sonn. | 800 | 15.0 | 70.0 | 10.0 | Villages and farmlands | Good |
| 1401 | Qinzhou | Pubei County | Sapindaceae | *Litchi chinensis* Sonn. | 800 | 11.0 | 79.6 | 10.0 | Villages and farmlands | Good |
| 1402 | Qinzhou | Pubei County | Sapindaceae | *Litchi chinensis* Sonn. | 800 | 15.0 | 79.6 | 12.0 | Villages and farmlands | Good |
| 1403 | Qinzhou | Pubei County | Theaceae | *Schima superba* Gardner & Champ. | 603 | 36.0 | 129.0 | 15.5 | Villages and farmlands | Good |
| 1404 | Qinzhou | Pubei County | Sapindaceae | *Dimocarpus longan* Lour. | 802 | 15.0 | 130.0 | 18.5 | Villages and farmlands | Good |
| 1405 | Qinzhou | Pubei County | Moraceae | *Ficus virens* Aiton. | 760 | 26.0 | 350.0 | 25.5 | Villages and farmlands | Good |
| 1406 | Qinzhou | Pubei County | Moraceae | *Ficus virens* Aiton. | 636 | 26.0 | 318.0 | 25.0 | Villages and farmlands | Good |
| 1407 | Qinzhou | Pubei County | Sapindaceae | *Dimocarpus longan* Lour. | 500 | 18.0 | 98.6 | 20.0 | Villages and farmlands | Good |
| 1408 | Guigang | Gangbei District | Podocarpaceae | *Dacrycarpus imbricatus* (Blume) de Laub. | 1100 | 30.0 | 145.8 | 29.0 | Villages and farmlands | Good |
| 1409 | Guigang | Gangbei District | Podocarpaceae | *Dacrycarpus imbricatus* (Blume) de Laub. | 1200 | 17.0 | 146.4 | 21.0 | Villages and farmlands | Good |
| 1410 | Guigang | Pingnan County | Moraceae | *Ficus virens* Aiton | 1100 | 24.0 | 398.0 | 40.5 | Villages and farmlands | Good |
| 1411 | Guigang | Pingnan County | Moraceae | *Ficus virens* Aiton | 1100 | 24.0 | 414.0 | 36.5 | Villages and farmlands | Good |
| 1412 | Guigang | Pingnan County | Moraceae | *Ficus microcarpa* L. f. | 1000 | 12.0 | 312.0 | 22.0 | Villages and farmlands | Good |
| 1413 | Guigang | Guiping City | Moraceae | *Ficus virens* Aiton. | 1100 | 28.8 | 296.2 | 41.5 | Scenic spots | Good |
| 1414 | Guigang | Gangbei District | Moraceae | *Ficus microcarpa* L. f. | 600 | 18.0 | 276.9 | 29.0 | Villages and farmlands | Good |
| 1415 | Guigang | Gangbei District | Moraceae | *Ficus microcarpa* L. f. | 590 | 17.3 | 273.7 | 25.0 | Villages and farmlands | Good |
| 1416 | Guigang | Gangbei District | Moraceae | *Ficus microcarpa* L. f. | 500 | 14.0 | 256.0 | 25.5 | Villages and farmlands | Good |
| 1417 | Guigang | Gangbei District | Fabaceae | *Erythrophleum fordii* Oliv. | 500 | 23.0 | 111.7 | 28.0 | Wooded areas and plant nurseries | Good |
| 1418 | Guigang | Gangbei District | Lamiaceae | *Vitex quinata* (Lour.) Will. | 500 | 8.0 | 54.8 | 7.5 | Villages and farmlands | Good |
| 1419 | Guigang | Gangbei District | Moraceae | *Ficus microcarpa* L. f. | 600 | 32.0 | 287.8 | 34.0 | Villages and farmlands | Good |
| 1420 | Guigang | Gangbei District | Moraceae | *Ficus microcarpa* L. f. | 520 | 17.0 | 261.0 | 27.5 | Villages and farmlands | Good |
| 1421 | Guigang | Gangbei District | Moraceae | *Ficus microcarpa* L. f. | 500 | 17.2 | 257.2 | 21.0 | Villages and farmlands | Good |
| 1422 | Guigang | Gangnan District | Lauraceae | *Camphora officinarum Nees.* | 510 | 15.2 | 220.0 | 16.0 | Villages and farmlands | Good |
| 1423 | Guigang | Gangnan District | Oxalidaceae | *Averrhoa carambo*la L. | 500 | 15.5 | 108.2 | 13.7 | Villages and farmlands | Good |
| 1424 | Guigang | Qintang District | Moraceae | *Ficus microcarpa* L. f. | 550 | 12.0 | 276.1 | 25.5 | Villages and farmlands | Good |
| 1425 | Guigang | Pingnan County | Fagaceae | *Quercus acutissima* Carruth. | 500 | 25.0 | 136.9 | 22.5 | Villages and farmlands | Good |
| 1426 | Guigang | Pingnan County | Lauraceae | *Camphora officinarum Nees.* | 510 | 18.0 | 232.0 | 27.0 | Villages and farmlands | Moderate |
| 1427 | Guigang | Pingnan County | Podocarpaceae | *Dacrycarpus imbricatus* (Blume) de Laub. | 500 | 16.0 | 106.6 | 17.0 | Villages and farmlands | Good |
| 1428 | Guigang | Pingnan County | Moraceae | *Ficus virens* Aiton. | 650 | 15.0 | 334.0 | 19.5 | Villages and farmlands | Moderate |
| 1429 | Guigang | Pingnan County | Moraceae | *Ficus virens* Aiton. | 520 | 17.0 | 286.0 | 32.0 | Villages and farmlands | Good |
| 1430 | Guigang | Pingnan County | Moraceae | *Ficus virens* Aiton. | 650 | 23.0 | 334.4 | 25.5 | Residential districts | Moderate |
| 1431 | Guigang | Pingnan County | Moraceae | *Ficus virens* Aiton. | 500 | 16.0 | 280.0 | 13.0 | Villages and farmlands | Moderate |
| 1432 | Guigang | Pingnan County | Moraceae | *Ficus virens* Aiton. | 600 | 18.0 | 318.0 | 23.5 | Villages and farmlands | Moderate |
| 1433 | Guigang | Pingnan County | Theaceae | *Schima superba* Gardner & Champ. | 500 | 25.0 | 127.0 | 16.9 | Villages and farmlands | Good |
| 1434 | Guigang | Guiping City | Podocarpaceae | *Dacrycarpus imbricatus* (Blume) de Laub. | 850 | 25.5 | 141.5 | 23.5 | Villages and farmlands | Poor |
| 1435 | Guigang | Guiping City | Moraceae | *Ficus microcarpa* L. f. | 510 | 25.0 | 280.3 | 33.8 | Villages and farmlands | Good |
| 1436 | Guigang | Guiping City | Lauraceae | *Camphora officinarum Nees.* | 550 | 24.0 | 242.0 | 24.0 | Villages and farmlands | Good |
| 1437 | Guigang | Guiping City | Moraceae | *Ficus virens* Aiton. | 500 | 21.0 | 259.6 | 29.0 | Residential districts | Moderate |
| 1438 | Guigang | Guiping City | Moraceae | *Ficus virens* Aiton. | 680 | 23.0 | 328.0 | 31.0 | Residential districts | Good |
| 1439 | Guigang | Guiping City | Moraceae | *Ficus virens* Aiton. | 800 | 23.0 | 340.8 | 33.0 | Residential districts | Good |
| 1440 | Guigang | Guiping City | Moraceae | *Ficus virens* Aiton. | 525 | 14.5 | 230.0 | 20.4 | Residential districts | Poor |
| 1441 | Guigang | Guiping City | Moraceae | *Ficus virens* Aiton. | 525 | 16.8 | 312.1 | 23.6 | Residential districts | Poor |
| 1442 | Guigang | Guiping City | Moraceae | *Ficus virens* Aiton. | 550 | 16.8 | 280.3 | 17.2 | Residential districts | Moderate |
| 1443 | Guigang | Guiping City | Moraceae | *Ficus microcarpa* L. f. | 500 | 23.0 | 253.0 | 27.0 | Residential districts | Good |
| 1444 | Guigang | Guiping City | Moraceae | *Ficus virens* Aiton. | 560 | 16.8 | 280.3 | 23.2 | Residential districts | Good |
| 1445 | Guigang | Guiping City | Lauraceae | *Camphora officinarum Nees.* | 550 | 25.0 | 281.9 | 30.8 | Residential districts | Good |
| 1446 | Guigang | Guiping City | Moraceae | *Ficus virens* Aiton. | 500 | 25.0 | 248.4 | 28.0 | Scenic spots | Good |
| 1447 | Guigang | Guiping City | Anacardiaceae | *Choerospondias axillaris* (Roxb.) B. L. Burtt & A. W. Hill | 520 | 21.5 | 146.5 | 16.1 | Scenic spots | Moderate |
| 1448 | Guigang | Guiping City | Pinaceae | *Pinus massoniana* Lamb. | 515 | 28.7 | 82.8 | 11.9 | Scenic spots | Moderate |
| 1449 | Guigang | Guiping City | Pinaceae | *Pinus massoniana* Lamb. | 525 | 34.0 | 108.3 | 14.8 | Scenic spots | Moderate |
| 1450 | Guigang | Guiping City | Pinaceae | *Pinus massoniana* Lamb. | 512 | 32.8 | 108.3 | 11.2 | Scenic spots | Good |
| 1451 | Guigang | Guiping City | Lauraceae | *Camphora officinarum Nees.* | 510 | 24.0 | 191.1 | 18.0 | Scenic spots | Good |
| 1452 | Yulin | Yuzhou District | Lauraceae | *Camphora officinarum Nees* | 1000 | 15.0 | 194.3 | 8.3 | Villages and farmlands | Moderate |
| 1453 | Yulin | Rong County | Moraceae | *Ficus microcarpa* L. f. | 1000 | 17.0 | 308.8 | 35.5 | Villages and farmlands | Good |
| 1454 | Yulin | Rong County | Fabaceae | *Erythrophleum fordii* Oliv. | 1700 | 16.2 | 132.1 | 21.5 | Villages and farmlands | Poor |
| 1455 | Yulin | Rong County | Moraceae | *Ficus concinna* (Miq.) Miq. | 1500 | 18.5 | 350.2 | 44.0 | Villages and farmlands | Moderate |
| 1456 | Yulin | Rong County | Fabaceae | *Lysidice rhodo*stegia Hance | 1000 | 26.4 | 184.6 | 27.0 | Villages and farmlands | Poor |
| 1457 | Yulin | Rong County | Fabaceae | *Erythrophleum fordii* Oliv. | 1750 | 23.4 | 133.7 | 28.5 | Villages and farmlands | Good |
| 1458 | Yulin | Rong County | Moraceae | *Ficus microcarpa* L. f. | 1100 | 19.6 | 330.1 | 41.0 | Villages and farmlands | Good |
| 1459 | Yulin | Rong County | Fabaceae | *Erythrophleum fordii* Oliv. | 1250 | 36.0 | 127.3 | 27.0 | Villages and farmlands | Good |
| 1460 | Yulin | Luchuan County | Sapindaceae | *Litchi chinensis* Sonn. | 1000 | 15.0 | 127.0 | 19.0 | Villages and farmlands | Good |
| 1461 | Yulin | Luchuan County | Moraceae | *Antiaris toxicaria* Lesch. | 1000 | 18.0 | 242.0 | 18.5 | Villages and farmlands | Moderate |
| 1462 | Yulin | Bobai County | Moraceae | *Ficus virens* Aiton. | 1000 | 30.0 | 380.0 | 29.5 | Villages and farmlands | Good |
| 1463 | Yulin | Bobai County | Moraceae | *Ficus microcarpa* L. f. | 1000 | 30.0 | 210.0 | 48.5 | Villages and farmlands | Good |
| 1464 | Yulin | Yuzhou District | Moraceae | *Ficus virens* Aiton. | 628 | 20.0 | 226.0 | 37.5 | Residential districts | Good |
| 1465 | Yulin | Fumian District | Moraceae | *Ficus microcarpa* L. f. | 620 | 20.0 | 280.0 | 34.0 | Residential districts | Good |
| 1466 | Yulin | Fumian District | Sapindaceae | *Dimocarpus longan* Lour. | 550 | 12.0 | 113.0 | 15.0 | Villages and farmlands | Good |
| 1467 | Yulin | Fumian District | Sapindaceae | *Dimocarpus longan* Lour. | 580 | 12.0 | 117.0 | 19.5 | Villages and farmlands | Poor |
| 1468 | Yulin | Fumian District | Sapindaceae | *Dimocarpus longan* Lour. | 610 | 14.0 | 118.0 | 16.0 | Villages and farmlands | Poor |
| 1469 | Yulin | Fumian District | Euphorbiaceae | *Mallotus repandus* (Willd.) Müll. Arg. | 500 | 16.0 | 103.0 | 11.0 | Villages and farmlands | Poor |
| 1470 | Yulin | Fumian District | Sapindaceae | *Dimocarpus longan* Lour. | 500 | 12.0 | 105.0 | 19.5 | Villages and farmlands | Moderate |
| 1471 | Yulin | Rong County | Cephalotaxaceae | *Cephalotaxus hainanensis* H. L. Li | 750 | 14.5 | 125.4 | 15.0 | Villages and farmlands | Poor |
| 1472 | Yulin | Rong County | Sapindaceae | *Dimocarpus longan* Lour. | 500 | 11.7 | 112.0 | 15.5 | Villages and farmlands | Moderate |
| 1473 | Yulin | Rong County | Fabaceae | *Erythrophleum fordii* Oliv. | 790 | 20.4 | 120.3 | 22.5 | Villages and farmlands | Good |
| 1474 | Yulin | Rong County | Fabaceae | *Erythrophleum fordii* Oliv. | 880 | 20.0 | 121.9 | 27.5 | Villages and farmlands | Moderate |
| 1475 | Yulin | Rong County | Fabaceae | *Erythrophleum fordii* Oliv. | 750 | 24.5 | 118.4 | 31.0 | Villages and farmlands | Moderate |
| 1476 | Yulin | Rong County | Lauraceae | *Camphora officinarum Nees* | 590 | 11.2 | 232.4 | 29.5 | Villages and farmlands | Poor |
| 1477 | Yulin | Rong County | Lauraceae | *Camphora officinarum Nees* | 500 | 22.4 | 217.4 | 30.0 | Villages and farmlands | Moderate |
| 1478 | Yulin | Rong County | Moraceae | *Ficus microcarpa* L. f. | 600 | 25.4 | 269.3 | 42.5 | Villages and farmlands | Good |
| 1479 | Yulin | Rong County | Fagaceae | *Castanopsis hystrix* Hook. f. & Thomson ex A. DC. | 600 | 24.8 | 151.8 | 20.5 | Villages and farmlands | Poor |
| 1480 | Yulin | Luchuan County | Sapindaceae | *Dimocarpus longa*n Lour. | 520 | 16.0 | 89.1 | 14.5 | Villages and farmlands | Good |
| 1481 | Yulin | Luchuan County | Sapindaceae | *Litchi chinensis* Sonn. | 800 | 10.0 | 60.0 | 11.5 | Villages and farmlands | Poor |
| 1482 | Yulin | Luchuan County | Sapindaceae | *Litchi chinensis* Sonn. | 800 | 11.0 | 76.0 | 10.5 | Villages and farmlands | Good |
| 1483 | Yulin | Luchuan County | Sapindaceae | *Litchi chinensis* Sonn. | 500 | 9.0 | 57.0 | 9.0 | Villages and farmlands | Good |
| 1484 | Yulin | Luchuan County | Fabaceae | *Adenanthera microsperma* Teijsm. & Binn. | 600 | 17.0 | 85.9 | 9.0 | Villages and farmlands | Poor |
| 1485 | Yulin | Luchuan County | Sapindaceae | *Dimocarpus longan* Lour. | 600 | 10.0 | 116.1 | 9.0 | Villages and farmlands | Moderate |
| 1486 | Yulin | Luchuan County | Sapotaceae | *Madhuca pasquieri* (Dubard) H. J. Lam | 500 | 25.0 | 111.5 | 20.5 | Villages and farmlands | Good |
| 1487 | Yulin | Luchuan County | Sapindaceae | *Dimocarpus longan* Lour. | 500 | 13.0 | 113.4 | 17.5 | Villages and farmlands | Good |
| 1488 | Yulin | Luchuan County | Sapindaceae | *Dimocarpus longan* Lour. | 500 | 14.0 | 110.8 | 8.5 | Villages and farmlands | Moderate |
| 1489 | Yulin | Luchuan County | Sapindaceae | *Dimocarpus longan* Lour. | 750 | 16.0 | 127.4 | 13.0 | Villages and farmlands | Moderate |
| 1490 | Yulin | Luchuan County | Sapindaceae | *Dimocarpus longan* Lour. | 550 | 9.0 | 114.0 | 9.4 | Villages and farmlands | Moderate |
| 1491 | Yulin | Luchuan County | Sapindaceae | *Dimocarpus longan* Lour. | 550 | 12.0 | 114.0 | 10.0 | Villages and farmlands | Moderate |
| 1492 | Yulin | Luchuan County | Sapindaceae | *Dimocarpus longan* Lour. | 549 | 7.0 | 111.8 | 9.5 | Villages and farmlands | Moderate |
| 1493 | Yulin | Luchuan County | Sapindaceae | *Dimocarpus longan* Lour. | 550 | 7.0 | 114.6 | 13.5 | Villages and farmlands | Moderate |
| 1494 | Yulin | Bobai County | Moraceae | *Ficus microcarpa* L. f. | 800 | 25.0 | 300.0 | 32.5 | Villages and farmlands | Good |
| 1495 | Yulin | Bobai County | Moraceae | *Ficus altissima* Blume. | 500 | 27.0 | 150.0 | 38.5 | Villages and farmlands | Good |
| 1496 | Yulin | Bobai County | Phyllanthaceae | *Bischofia javanica* Blume. | 800 | 30.0 | 250.0 | 19.0 | Villages and farmlands | Good |
| 1497 | Yulin | Bobai County | Moraceae | *Artocarpus parvus* Gagnep. | 530 | 24.0 | 110.0 | 25.0 | Villages and farmlands | Good |
| 1498 | Yulin | Bobai County | Sapindaceae | *Dimocarpus longan* Lour. | 600 | 13.0 | 120.0 | 12.5 | Villages and farmlands | Good |
| 1499 | Yulin | Bobai County | Moraceae | *Ficus altissima* Blume. | 500 | 25.0 | 210.0 | 39.0 | Villages and farmlands | Good |
| 1500 | Yulin | Bobai County | Aquifoliaceae | *Ilex rotunda* Thunb. | 500 | 14.0 | 92.0 | 19.0 | Villages and farmlands | Good |
| 1501 | Yulin | Bobai County | Apocynaceae | *Alstonia scholaris* (L.) R. Br. | 600 | 27.0 | 216.0 | 25.0 | Villages and farmlands | Good |
| 1502 | Yulin | Xingye County | Lauraceae | *Camphora officinarum Nees.* | 500 | 20.8 | 210.0 | 32.6 | Villages and farmlands | Moderate |
| 1503 | Yulin | Beiliu City | Oleaceae | *Osmanthus fragrans* (Thunb.) Lour. | 600 | 6.0 | 42.0 | 5.5 | Villages and farmlands | Moderate |
| 1504 | Yulin | Beiliu City | Sapindaceae | *Litchi chinensis* Sonn. | 800 | 24.3 | 172.0 | 15.2 | Villages and farmlands | Good |
| 1505 | Yulin | Beiliu City | Sapindaceae | *Litchi chinensis* Sonn. | 800 | 23.5 | 146.5 | 14.9 | Villages and farmlands | Good |
| 1506 | Yulin | Beiliu City | Sapindaceae | *Litchi chinensis* Sonn. | 800 | 20.6 | 105.1 | 13.0 | Villages and farmlands | Good |
| 1507 | Yulin | Beiliu City | Sapindaceae | *Litchi chinensis* Sonn. | 600 | 14.0 | 133.0 | 15.0 | Villages and farmlands | Good |
| 1508 | Yulin | Beiliu City | Sapindaceae | *Litchi chinensis* Sonn. | 600 | 13.0 | 96.0 | 16.5 | Villages and farmlands | Good |
| 1509 | Yulin | Beiliu City | Sapindaceae | *Litchi chinensis* Sonn. | 600 | 14.0 | 115.0 | 16.5 | Villages and farmlands | Good |
| 1510 | Yulin | Beiliu City | Moraceae | *Ficus virens* Aiton. | 550 | 19.0 | 287.0 | 30.0 | Villages and farmlands | Good |
| 1511 | Yulin | Beiliu City | Moraceae | *Ficus virens* Aiton. | 500 | 18.0 | 286.0 | 29.0 | Villages and farmlands | Good |
| 1512 | Yulin | Beiliu City | Moraceae | *Ficus virens* Aiton. | 550 | 22.0 | 292.0 | 28.5 | Villages and farmlands | Good |
| 1513 | Yulin | Beiliu City | Anacardiaceae | *Dracontomelon duperreanum* Pierre | 550 | 20.0 | 232.0 | 23.0 | Villages and farmlands | Good |
| 1514 | Yulin | Beiliu City | Moraceae | *Ficus microcarpa* L. f. | 650 | 27.0 | 282.0 | 35.5 | Villages and farmlands | Good |
| 1515 | Yulin | Beiliu City | Moraceae | *Ficus microcarpa* L. f. | 500 | 25.0 | 280.0 | 29.5 | Villages and farmlands | Good |
| 1516 | Baise | Youjiang District | Moraceae | *Ficus altissima* Blume. | 1000 | 20.0 | 363.1 | 35.5 | Villages and farmlands | Good |
| 1517 | Baise | Tianyang District | Moraceae | *Ficus altissima* Blume. | 1000 | 17.0 | 414.0 | 27.9 | Villages and farmlands | Good |
| 1518 | Baise | Tianyang District | Moraceae | *Ficus altissima* Blume. | 1000 | 19.5 | 458.6 | 46.4 | Villages and farmlands | Good |
| 1519 | Baise | Tianyang District | Moraceae | *Ficus altissima* Blume. | 1000 | 22.2 | 293.0 | 40.9 | Villages and farmlands | Good |
| 1520 | Baise | Tianyang District | Malvaceae | *Excentrodendron tonkinense* (A. Chev.) H. T. Chang & R. H. Miao | 1000 | 35.5 | 181.2 | 11.0 | Villages and farmlands | Good |
| 1521 | Baise | Tianyang District | Malvaceae | *Excentrodendron tonkinense* (A. Chev.) H. T. Chang & R. H. Miao | 1000 | 25.6 | 165.0 | 18.0 | Villages and farmlands | Good |
| 1522 | Baise | Tiandong County | Moraceae | *Ficus altissima* Blume. | 1000 | 18.0 | 111.5 | 66.0 | Villages and farmlands | Good |
| 1523 | Baise | Tiandong County | Moraceae | *Ficus altissima* Blume. | 1200 | 19.0 | 509.6 | 31.0 | Villages and farmlands | Good |
| 1524 | Baise | Tiandong County | Moraceae | *Ficus altissima* Blume. | 1200 | 21.0 | 541.4 | 30.0 | Villages and farmlands | Good |
| 1525 | Baise | Tiandong County | Moraceae | *Ficus virens* Aiton. | 1000 | 21.0 | 477.7 | 48.0 | Villages and farmlands | Good |
| 1526 | Baise | Pingguo County | Sapotaceae | *Sinosideroxylon pedunculatum* (Hemsl.) H. Chuang | 1000 | 33.0 | 127.0 | 19.5 | Villages and farmlands | Good |
| 1527 | Baise | Debao County | Moraceae | *Ficus virens* Aiton. | 1500 | 31.0 | 568.5 | 54.8 | Villages and farmlands | Good |
| 1528 | Baise | Debao County | Moraceae | *Ficus virens* Aiton. | 1000 | 25.0 | 408.0 | 30.0 | Villages and farmlands | Good |
| 1529 | Baise | Jingxi City | Moraceae | *Ficus virens* Aiton. | 1000 | 30.0 | 415.0 | 36.0 | Villages and farmlands | Good |
| 1530 | Baise | Jingxi City | Moraceae | *Ficus virens* Aiton. | 1000 | 30.0 | 417.0 | 36.8 | Villages and farmlands | Good |
| 1531 | Baise | Jingxi City | Cupressaceae | *Calocedrus macrolepis* Kurz. | 1000 | 21.0 | 219.0 | 16.1 | Villages and farmlands | Good |
| 1532 | Baise | Jingxi City | Moraceae | *Ficus virens* Aiton. | 1000 | 42.0 | 406.0 | 35.5 | Villages and farmlands | Good |
| 1533 | Baise | Jingxi City | Moraceae | *Ficus virens* Aiton. | 1000 | 19.0 | 408.0 | 47.0 | Villages and farmlands | Good |
| 1534 | Baise | Jingxi City | Moraceae | *Ficus virens* Aiton. | 1000 | 24.0 | 403.6 | 29.0 | Villages and farmlands | Good |
| 1535 | Baise | Jingxi City | Moraceae | *Ficus virens* Aiton. | 1000 | 28.0 | 400.0 | 40.0 | Villages and farmlands | Good |
| 1536 | Baise | Jingxi City | Meliaceae | *Chukrasia tabularis* A. Juss. | 1000 | 30.0 | 193.5 | 27.0 | Villages and farmlands | Good |
| 1537 | Baise | Jingxi City | Malvaceae | *Excentrodendron tonkinense* (A. Chev.) H. T. Chang & R. H. Miao | 1000 | 25.0 | 193.0 | 21.0 | Villages and farmlands | Good |
| 1538 | Baise | Napo County | Moraceae | *Ficus virens* Aiton. | 1000 | 20.0 | 380.0 | 40.0 | Villages and farmlands | Good |
| 1539 | Baise | Lingyun County | Moraceae | *Ficus virens* Aiton. | 1100 | 30.0 | 490.0 | 22.5 | Villages and farmlands | Good |
| 1540 | Baise | Lingyun County | Juglandaceae | *Carya sinensis* Dode. | 1100 | 24.0 | 255.0 | 11.0 | Villages and farmlands | Good |
| 1541 | Baise | Lingyun County | Moraceae | *Ficus virens* Aiton. | 1300 | 35.0 | 573.0 | 64.5 | Villages and farmlands | Good |
| 1542 | Baise | Leye County | Juglandaceae | *Carya sinensis* Dode. | 1800 | 40.0 | 322.0 | 18.7 | Villages and farmlands | Good |
| 1543 | Baise | Leye County | Juglandaceae | *Carya sinensis* Dode. | 1800 | 38.0 | 234.7 | 17.0 | Villages and farmlands | Good |
| 1544 | Baise | Leye County | Juglandaceae | *Carya sinensis* Dode. | 1200 | 38.0 | 219.7 | 17.7 | Villages and farmlands | Good |
| 1545 | Baise | Tianlin County | Moraceae | *Ficus virens* Aiton. | 1000 | 23.0 | 450.0 | 29.5 | Villages and farmlands | Good |
| 1546 | Baise | Tianlin County | Moraceae | *Ficus virens* Aiton. | 1000 | 31.0 | 722.0 | 47.5 | Residential districts | Good |
| 1547 | Baise | Tianlin County | Moraceae | *Ficus microcarpa* L. f. | 1000 | 19.0 | 329.6 | 41.0 | Villages and farmlands | Good |
| 1548 | Baise | Xilin County | Moraceae | *Ficus virens* Aiton. | 1100 | 26.0 | 423.0 | 54.0 | Villages and farmlands | Good |
| 1549 | Baise | Longlin County | Moraceae | *Ficus virens* Aiton. | 1000 | 24.0 | 458.0 | 33.5 | Villages and farmlands | Good |
| 1550 | Baise | Longlin County | Moraceae | *Ficus virens* Aiton. | 1000 | 24.0 | 420.0 | 26.0 | Villages and farmlands | Good |
| 1551 | Baise | Longlin County | Moraceae | *Ficus virens* Aiton. | 1000 | 20.0 | 414.0 | 28.0 | Villages and farmlands | Good |
| 1552 | Baise | Longlin County | Moraceae | *Ficus virens* Aiton. | 1000 | 28.0 | 414.0 | 30.0 | Villages and farmlands | Good |
| 1553 | Baise | Longlin County | Moraceae | *Ficus virens* Aiton. | 1300 | 26.0 | 455.0 | 35.0 | Villages and farmlands | Good |
| 1554 | Baise | Longlin County | Moraceae | *Ficus virens* Aiton. | 1300 | 22.0 | 512.7 | 50.0 | Villages and farmlands | Good |
| 1555 | Baise | Longlin County | Moraceae | *Ficus virens* Aiton. | 1000 | 18.0 | 509.3 | 44.0 | Villages and farmlands | Good |
| 1556 | Baise | Longlin County | Moraceae | *Ficus virens* Aiton. | 1000 | 18.0 | 487.0 | 47.0 | Villages and farmlands | Good |
| 1557 | Baise | Longlin County | Moraceae | *Ficus virens* Aiton. | 1000 | 23.0 | 439.0 | 27.0 | Villages and farmlands | Good |
| 1558 | Baise | Longlin County | Moraceae | *Ficus virens* Aiton. | 1000 | 31.0 | 420.0 | 29.0 | Villages and farmlands | Good |
| 1559 | Baise | Youjiang District | Moraceae | *Ficus altissima* Blume. | 600 | 25.0 | 300.0 | 45.0 | Villages and farmlands | Good |
| 1560 | Baise | Youjiang District | Moraceae | *Ficus altissima* Blume. | 500 | 22.0 | 270.0 | 30.0 | Villages and farmlands | Good |
| 1561 | Baise | Youjiang District | Moraceae | *Ficus altissima* Blume. | 740 | 25.0 | 306.5 | 42.0 | Villages and farmlands | Good |
| 1562 | Baise | Youjiang District | Moraceae | *Ficus altissima* Blume. | 500 | 28.0 | 281.0 | 36.0 | Villages and farmlands | Good |
| 1563 | Baise | Youjiang District | Moraceae | *Ficus altissima* Blume. | 500 | 30.0 | 334.0 | 22.0 | Villages and farmlands | Good |
| 1564 | Baise | Youjiang District | Moraceae | *Ficus altissima* Blume. | 500 | 22.0 | 311.0 | 24.5 | Villages and farmlands | Good |
| 1565 | Baise | Youjiang District | Moraceae | *Ficus altissima* Blume. | 500 | 28.0 | 347.0 | 50.0 | Villages and farmlands | Good |
| 1566 | Baise | Youjiang District | Moraceae | *Ficus altissima* Blume. | 500 | 24.0 | 334.0 | 50.0 | Villages and farmlands | Good |
| 1567 | Baise | Youjiang District | Moraceae | *Ficus altissima* Blume. | 500 | 15.0 | 280.2 | 30.5 | Villages and farmlands | Good |
| 1568 | Baise | Youjiang District | Moraceae | *Ficus virens* Aiton. | 500 | 22.0 | 280.0 | 29.5 | Villages and farmlands | Moderate |
| 1569 | Baise | Youjiang District | Moraceae | *Ficus virens* Aiton. | 500 | 25.0 | 310.0 | 30.0 | Villages and farmlands | Good |
| 1570 | Baise | Youjiang District | Moraceae | *Ficus virens* Aiton. | 500 | 17.0 | 216.0 | 19.2 | Villages and farmlands | Moderate |
| 1571 | Baise | Youjiang District | Moraceae | *Ficus virens* Aiton. | 500 | 17.0 | 242.0 | 19.5 | Villages and farmlands | Good |
| 1572 | Baise | Youjiang District | Moraceae | *Ficus virens* Aiton. | 500 | 25.0 | 296.0 | 24.0 | Villages and farmlands | Moderate |
| 1573 | Baise | Youjiang District | Moraceae | *Ficus virens* Aiton. | 500 | 21.0 | 302.0 | 21.5 | Villages and farmlands | Good |
| 1574 | Baise | Youjiang District | Moraceae | *Ficus virens* Aiton. | 500 | 22.0 | 270.0 | 31.5 | Villages and farmlands | Moderate |
| 1575 | Baise | Youjiang District | Moraceae | *Ficus microcarpa* L. f. | 500 | 24.0 | 254.0 | 34.0 | Villages and farmlands | Good |
| 1576 | Baise | Youjiang District | Moraceae | *Ficus altissima* Blume. | 600 | 26.0 | 310.0 | 40.5 | Villages and farmlands | Good |
| 1577 | Baise | Youjiang District | Sapindaceae | *Dimocarpus longan* Lour. | 500 | 10.0 | 121.0 | 11.0 | Villages and farmlands | Moderate |
| 1578 | Baise | Youjiang District | Moraceae | *Ficus altissima* Blume. | 500 | 35.0 | 345.0 | 38.5 | Villages and farmlands | Good |
| 1579 | Baise | Youjiang District | Moraceae | *Ficus virens* Aiton. | 500 | 30.0 | 300.0 | 20.0 | Villages and farmlands | Good |
| 1580 | Baise | Tianyang District | Moraceae | *Ficus virens* Aiton. | 500 | 16.0 | 280.4 | 27.4 | Residential districts | Moderate |
| 1581 | Baise | Tianyang District | Moraceae | *Ficus altissima* Blume. | 600 | 18.0 | 311.9 | 41.4 | Villages and farmlands | Good |
| 1582 | Baise | Tianyang District | Moraceae | *Ficus virens* Aiton. | 500 | 15.2 | 283.4 | 23.4 | Villages and farmlands | Good |
| 1583 | Baise | Tianyang District | Moraceae | *Ficus virens* Aiton. | 700 | 23.0 | 340.0 | 37.5 | Villages and farmlands | Good |
| 1584 | Baise | Tianyang District | Moraceae | *Ficus altissima* Blume. | 500 | 25.0 | 276.4 | 37.0 | Villages and farmlands | Good |
| 1585 | Baise | Tianyang District | Moraceae | *Ficus virens* Aiton. | 600 | 22.5 | 312.1 | 45.0 | Villages and farmlands | Good |
| 1586 | Baise | Tianyang District | Moraceae | *Ficus virens* Aiton. | 850 | 24.2 | 379.0 | 25.0 | Villages and farmlands | Good |
| 1587 | Baise | Tianyang District | Moraceae | *Ficus virens* Aiton. | 500 | 25.0 | 273.9 | 29.0 | Villages and farmlands | Good |
| 1588 | Baise | Tianyang District | Moraceae | *Ficus virens* Aiton. | 700 | 17.2 | 318.5 | 42.0 | Villages and farmlands | Good |
| 1589 | Baise | Tianyang District | Moraceae | *Ficus virens* Aiton. | 550 | 25.0 | 275.5 | 40.0 | Villages and farmlands | Good |
| 1590 | Baise | Tianyang District | Moraceae | *Ficus virens* Aiton. | 650 | 20.0 | 302.6 | 34.0 | Villages and farmlands | Good |
| 1591 | Baise | Tianyang District | Moraceae | *Ficus virens* Aiton. | 500 | 18.0 | 275.0 | 29.0 | Villages and farmlands | Good |
| 1592 | Baise | Tianyang District | Moraceae | *Ficus virens* Aiton. | 500 | 16.5 | 258.0 | 25.8 | Villages and farmlands | Good |
| 1593 | Baise | Tianyang District | Moraceae | *Ficus virens* Aiton. | 750 | 20.0 | 350.3 | 36.8 | Villages and farmlands | Poor |
| 1594 | Baise | Tianyang District | Moraceae | *Ficus virens* Aiton. | 550 | 20.0 | 286.6 | 29.0 | Villages and farmlands | Good |
| 1595 | Baise | Tianyang District | Moraceae | *Ficus microcarpa* L. f. | 900 | 22.0 | 324.8 | 12.5 | Villages and farmlands | Good |
| 1596 | Baise | Tianyang District | Moraceae | *Ficus virens* Aiton. | 900 | 30.0 | 324.8 | 40.0 | Villages and farmlands | Good |
| 1597 | Baise | Tianyang District | Sapindaceae | *Litchi chinensis* Sonn. | 700 | 20.5 | 208.3 | 22.9 | Villages and farmlands | Good |
| 1598 | Baise | Tiandong County | Moraceae | *Ficus microcarpa* L. f. | 520 | 16.0 | 261.0 | 23.0 | Villages and farmlands | Good |
| 1599 | Baise | Tiandong County | Moraceae | *Ficus altissima* Blume. | 850 | 30.2 | 318.5 | 35.0 | Villages and farmlands | Good |
| 1600 | Baise | Tiandong County | Moraceae | *Ficus altissima* Blume. | 600 | 17.3 | 285.1 | 14.3 | Villages and farmlands | Good |
| 1601 | Baise | Tiandong County | Moraceae | *Ficus microcarpa* L. f. | 500 | 25.0 | 254.0 | 25.5 | Villages and farmlands | Good |
| 1602 | Baise | Tiandong County | Moraceae | *Ficus altissima* Blume. | 600 | 19.0 | 280.0 | 24.0 | Villages and farmlands | Good |
| 1603 | Baise | Pingguo County | Moraceae | *Ficus virens* Aiton. | 950 | 21.2 | 327.0 | 19.0 | Villages and farmlands | Good |
| 1604 | Baise | Pingguo County | Malvaceae | *Excentrodendron tonkinense* (A. Chev.) H. T. Chang & R. H. Miao | 510 | 15.0 | 126.0 | 7.5 | Villages and farmlands | Good |
| 1605 | Baise | Pingguo County | Moraceae | *Ficus microcarpa* L. f. | 500 | 10.0 | 260.0 | 12.0 | Villages and farmlands | Good |
| 1606 | Baise | Pingguo County | Moraceae | *Ficus altissima* Blume. | 600 | 16.0 | 275.0 | 29.4 | Villages and farmlands | Good |
| 1607 | Baise | Pingguo County | Oxalidaceae | *Averrhoa carambola* L. | 500 | 11.0 | 98.6 | 11.5 | Villages and farmlands | Poor |
| 1608 | Baise | Pingguo County | Moraceae | *Ficus virens* Aiton. | 500 | 25.0 | 276.0 | 26.5 | Villages and farmlands | Moderate |
| 1609 | Baise | Pingguo County | Annonaceae | *Alphonsea mollis* Dunn. | 500 | 26.0 | 124.0 | 13.0 | Villages and farmlands | Moderate |
| 1610 | Baise | Pingguo County | Moraceae | *Ficus altissima* Blume. | 650 | 21.0 | 300.0 | 24.5 | Villages and farmlands | Good |
| 1611 | Baise | Pingguo County | Malvaceae | *Excentrodendron tonkinense* (A. Chev.) H. T. Chang & R. H. Miao | 650 | 30.0 | 150.0 | 20.0 | Villages and farmlands | Moderate |
| 1612 | Baise | Pingguo County | Moraceae | *Ficus altissima* Blume. | 500 | 23.0 | 270.0 | 28.5 | Villages and farmlands | Good |
| 1613 | Baise | Pingguo County | Moraceae | *Ficus altissima* Blume. | 560 | 20.0 | 280.0 | 19.5 | Villages and farmlands | Good |
| 1614 | Baise | Pingguo County | Malvaceae | *Excentrodendron tonkinense* (A. Chev.) H. T. Chang & R. H. Miao | 550 | 15.0 | 130.0 | 8.0 | Villages and farmlands | Good |
| 1615 | Baise | Debao County | Moraceae | *Ficus virens* Aiton. | 500 | 18.4 | 270.0 | 18.8 | Villages and farmlands | Good |
| 1616 | Baise | Debao County | Anacardiaceae | *Pistacia chinens*is Bunge. | 500 | 14.8 | 190.0 | 18.0 | Villages and farmlands | Moderate |
| 1617 | Baise | Debao County | Moraceae | *Ficus virens* Aiton. | 760 | 27.3 | 350.0 | 32.8 | Villages and farmlands | Good |
| 1618 | Baise | Debao County | Moraceae | *Ficus virens* Aiton. | 500 | 18.6 | 264.3 | 42.5 | Villages and farmlands | Good |
| 1619 | Baise | Debao County | Juglandaceae | *Carya sinensis* Dode. | 800 | 16.0 | 190.0 | 13.0 | Villages and farmlands | Good |
| 1620 | Baise | Debao County | Rosaceae | *Prunus dulcis* (Mill.) D. A. Webb | 500 | 28.0 | 240.0 | 26.1 | Villages and farmlands | Good |
| 1621 | Baise | Debao County | Moraceae | *Ficus virens* Aiton. | 950 | 23.3 | 390.8 | 25.0 | Villages and farmlands | Good |
| 1622 | Baise | Debao County | Moraceae | *Ficus virens* Aiton. | 560 | 22.0 | 300.2 | 22.5 | Villages and farmlands | Good |
| 1623 | Baise | Debao County | Moraceae | *Ficus virens* Aiton. | 750 | 22.0 | 312.2 | 24.0 | Villages and farmlands | Good |
| 1624 | Baise | Debao County | Moraceae | *Ficus virens* Aiton. | 750 | 29.0 | 340.0 | 41.2 | Villages and farmlands | Good |
| 1625 | Baise | Debao County | Moraceae | *Ficus altissima* Blume. | 500 | 32.4 | 280.0 | 30.0 | Villages and farmlands | Good |
| 1626 | Baise | Jingxi City | Malvaceae | *Excentrodendron tonkinense* (A. Chev.) H. T. Chang & R. H. Miao | 550 | 29.0 | 124.1 | 11.0 | Residential districts | Moderate |
| 1627 | Baise | Jingxi City | Moraceae | *Ficus virens* Aiton. | 900 | 30.0 | 382.0 | 33.0 | Villages and farmlands | Good |
| 1628 | Baise | Jingxi City | Moraceae | *Ficus virens* Aiton. | 550 | 30.0 | 299.2 | 33.0 | Villages and farmlands | Good |
| 1629 | Baise | Jingxi City | Moraceae | *Ficus virens* Aiton. | 500 | 16.0 | 305.0 | 27.0 | Villages and farmlands | Good |
| 1630 | Baise | Jingxi City | Moraceae | *Ficus virens* Aiton. | 600 | 21.0 | 366.0 | 22.5 | Villages and farmlands | Good |
| 1631 | Baise | Jingxi City | Moraceae | *Ficus virens* Aiton. | 650 | 17.0 | 312.0 | 17.5 | Villages and farmlands | Good |
| 1632 | Baise | Jingxi City | Moraceae | *Ficus virens* Aiton. | 750 | 25.0 | 238.8 | 24.5 | Villages and farmlands | Good |
| 1633 | Baise | Jingxi City | Sapindaceae | *Dimocarpus longan* Lour. | 550 | 18.0 | 114.0 | 19.0 | Villages and farmlands | Good |
| 1634 | Baise | Jingxi City | Moraceae | *Ficus virens* Aiton. | 650 | 28.3 | 347.3 | 44.0 | Villages and farmlands | Good |
| 1635 | Baise | Jingxi City | Moraceae | *Ficus racemosa* L. | 500 | 9.3 | 280.4 | 12.9 | Villages and farmlands | Good |
| 1636 | Baise | Jingxi City | Moraceae | *Ficus virens* Aiton. | 500 | 17.5 | 280.1 | 28.8 | Villages and farmlands | Good |
| 1637 | Baise | Jingxi City | Moraceae | *Ficus virens* Aiton. | 650 | 36.0 | 318.3 | 40.0 | Villages and farmlands | Good |
| 1638 | Baise | Jingxi City | Moraceae | *Ficus racemosa* L. | 550 | 29.0 | 307.2 | 30.0 | Villages and farmlands | Good |
| 1639 | Baise | Jingxi City | Sapindaceae | *Dimocarpus longan* Lour. | 500 | 18.3 | 115.6 | 21.0 | Villages and farmlands | Good |
| 1640 | Baise | Jingxi City | Moraceae | *Ficus virens* Aiton. | 550 | 17.0 | 292.9 | 40.0 | Villages and farmlands | Good |
| 1641 | Baise | Jingxi City | Moraceae | *Ficus virens* Aiton. | 500 | 23.0 | 283.3 | 36.6 | Villages and farmlands | Good |
| 1642 | Baise | Jingxi City | Moraceae | *Ficus virens* Aiton. | 800 | 17.0 | 355.0 | 27.5 | Villages and farmlands | Good |
| 1643 | Baise | Jingxi City | Moraceae | *Ficus virens* Aiton. | 800 | 16.2 | 417.0 | 19.8 | Villages and farmlands | Good |
| 1644 | Baise | Jingxi City | Moraceae | *Ficus virens* Aiton. | 700 | 16.9 | 337.4 | 33.8 | Villages and farmlands | Good |
| 1645 | Baise | Jingxi City | Moraceae | *Ficus virens* Aiton. | 500 | 20.8 | 264.2 | 40.0 | Villages and farmlands | Good |
| 1646 | Baise | Jingxi City | Sapindaceae | *Dimocarpus longan* Lour. | 600 | 15.7 | 119.4 | 20.9 | Villages and farmlands | Good |
| 1647 | Baise | Jingxi City | Moraceae | *Ficus virens* Aiton. | 500 | 20.0 | 300.0 | 24.5 | Villages and farmlands | Good |
| 1648 | Baise | Jingxi City | Moraceae | *Ficus virens* Aiton. | 750 | 35.0 | 350.0 | 49.0 | Villages and farmlands | Good |
| 1649 | Baise | Jingxi City | Moraceae | *Ficus virens* Aiton. | 650 | 25.0 | 328.0 | 29.0 | Villages and farmlands | Good |
| 1650 | Baise | Jingxi City | Moraceae | *Ficus virens* Aiton. | 800 | 25.0 | 366.0 | 15.5 | Villages and farmlands | Good |
| 1651 | Baise | Jingxi City | Moraceae | *Ficus virens* Aiton. | 700 | 20.0 | 340.0 | 19.0 | Villages and farmlands | Good |
| 1652 | Baise | Jingxi City | Moraceae | *Ficus virens* Aiton. | 550 | 20.0 | 298.0 | 29.5 | Villages and farmlands | Good |
| 1653 | Baise | Jingxi City | Malvaceae | *Excentrodendron tonkinense* (A. Chev.) H. T. Chang & R. H. Miao | 650 | 37.0 | 127.3 | 13.0 | Villages and farmlands | Good |
| 1654 | Baise | Jingxi City | Malvaceae | *Excentrodendron tonkinense* (A. Chev.) H. T. Chang & R. H. Miao | 500 | 12.0 | 114.6 | 10.0 | Villages and farmlands | Moderate |
| 1655 | Baise | Jingxi City | Malvaceae | *Excentrodendron tonkinense* (A. Chev.) H. T. Chang & R. H. Miao | 500 | 26.0 | 110.0 | 13.2 | Villages and farmlands | Good |
| 1656 | Baise | Jingxi City | Moraceae | *Ficus virens* Aiton. | 550 | 24.0 | 286.5 | 36.0 | Villages and farmlands | Good |
| 1657 | Baise | Jingxi City | Malvaceae | *Excentrodendron tonkinense* (A. Chev.) H. T. Chang & R. H. Miao | 500 | 26.0 | 117.8 | 14.5 | Villages and farmlands | Good |
| 1658 | Baise | Jingxi City | Malvaceae | *Excentrodendron tonkinense* (A. Chev.) H. T. Chang & R. H. Miao | 650 | 32.0 | 130.5 | 24.9 | Villages and farmlands | Good |
| 1659 | Baise | Jingxi City | Moraceae | *Ficus virens* Aiton. | 550 | 30.0 | 282.3 | 43.0 | Villages and farmlands | Good |
| 1660 | Baise | Jingxi City | Moraceae | *Ficus virens* Aiton. | 500 | 32.0 | 267.0 | 27.0 | Villages and farmlands | Moderate |
| 1661 | Baise | Jingxi City | Moraceae | *Ficus virens* Aiton. | 500 | 34.0 | 292.0 | 30.0 | Villages and farmlands | Moderate |
| 1662 | Baise | Jingxi City | Moraceae | *Ficus virens* Aiton. | 600 | 35.0 | 320.0 | 29.0 | Villages and farmlands | Good |
| 1663 | Baise | Jingxi City | Moraceae | *Ficus virens* Aiton. | 600 | 32.0 | 310.0 | 40.0 | Villages and farmlands | Good |
| 1664 | Baise | Jingxi City | Moraceae | *Ficus virens* Aiton. | 700 | 36.0 | 360.0 | 25.0 | Villages and farmlands | Good |
| 1665 | Baise | Jingxi City | Moraceae | *Ficus virens* Aiton. | 650 | 30.0 | 320.0 | 32.0 | Villages and farmlands | Good |
| 1666 | Baise | Jingxi City | Moraceae | *Ficus virens* Aiton. | 500 | 31.0 | 280.0 | 31.5 | Villages and farmlands | Good |
| 1667 | Baise | Jingxi City | Moraceae | *Ficus virens* Aiton. | 500 | 35.0 | 285.0 | 40.0 | Villages and farmlands | Good |
| 1668 | Baise | Jingxi City | Moraceae | *Ficus virens* Aiton. | 500 | 18.0 | 283.0 | 24.0 | Villages and farmlands | Good |
| 1669 | Baise | Jingxi City | Moraceae | *Ficus virens* Aiton. | 500 | 35.0 | 276.9 | 60.0 | Villages and farmlands | Good |
| 1670 | Baise | Jingxi City | Moraceae | *Ficus virens* Aiton. | 750 | 33.0 | 350.0 | 34.0 | Villages and farmlands | Good |
| 1671 | Baise | Jingxi City | Moraceae | *Ficus virens* Aiton. | 750 | 30.0 | 350.0 | 14.0 | Villages and farmlands | Good |
| 1672 | Baise | Jingxi City | Moraceae | *Ficus virens* Aiton. | 600 | 30.0 | 310.0 | 15.0 | Villages and farmlands | Good |
| 1673 | Baise | Jingxi City | Sapindaceae | *Dimocarpus longan* Lour. | 500 | 10.0 | 108.0 | 10.0 | Villages and farmlands | Good |
| 1674 | Baise | Jingxi City | Moraceae | *Ficus virens* Aiton. | 650 | 19.0 | 320.0 | 39.0 | Villages and farmlands | Good |
| 1675 | Baise | Jingxi City | Moraceae | *Ficus virens* Aiton. | 650 | 20.0 | 320.0 | 40.5 | Villages and farmlands | Good |
| 1676 | Baise | Jingxi City | Moraceae | *Ficus virens* Aiton. | 550 | 12.0 | 300.0 | 13.0 | Villages and farmlands | Good |
| 1677 | Baise | Jingxi City | Moraceae | *Ficus virens* Aiton. | 550 | 35.0 | 300.0 | 39.5 | Villages and farmlands | Good |
| 1678 | Baise | Jingxi City | Phyllanthaceae | *Bischofia javanica* Blume. | 500 | 19.0 | 223.0 | 18.5 | Villages and farmlands | Poor |
| 1679 | Baise | Jingxi City | Moraceae | *Ficus virens* Aiton. | 600 | 22.0 | 290.0 | 49.0 | Villages and farmlands | Moderate |
| 1680 | Baise | Jingxi City | Moraceae | *Ficus virens* Aiton. | 650 | 18.0 | 330.2 | 11.0 | Villages and farmlands | Moderate |
| 1681 | Baise | Jingxi City | Moraceae | *Ficus racemosa* L. | 500 | 17.0 | 261.0 | 14.0 | Villages and farmlands | Good |
| 1682 | Baise | Jingxi City | Moraceae | *Ficus virens* Aiton. | 750 | 22.0 | 350.2 | 35.5 | Villages and farmlands | Good |
| 1683 | Baise | Jingxi City | Sapindaceae | *Litchi chinensis* Sonn. | 650 | 16.0 | 219.0 | 16.0 | Villages and farmlands | Good |
| 1684 | Baise | Jingxi City | Moraceae | *Ficus virens* Aiton. | 600 | 18.0 | 308.1 | 19.5 | Villages and farmlands | Good |
| 1685 | Baise | Jingxi City | Moraceae | *Ficus virens* Aiton. | 800 | 21.0 | 366.1 | 12.0 | Villages and farmlands | Good |
| 1686 | Baise | Jingxi City | Moraceae | *Ficus virens* Aiton. | 500 | 23.0 | 269.0 | 20.0 | Villages and farmlands | Good |
| 1687 | Baise | Jingxi City | Malvaceae | *Excentrodendron tonkinense* (A. Chev.) H. T. Chang & R. H. Miao | 600 | 22.0 | 130.5 | 17.0 | Villages and farmlands | Good |
| 1688 | Baise | Jingxi City | Moraceae | *Ficus virens* Aiton. | 500 | 22.0 | 296.4 | 16.5 | Villages and farmlands | Good |
| 1689 | Baise | Jingxi City | Moraceae | *Ficus microcarpa* L. f. | 500 | 20.0 | 254.7 | 33.5 | Villages and farmlands | Good |
| 1690 | Baise | Jingxi City | Moraceae | *Ficus virens* Aiton. | 600 | 20.0 | 286.5 | 11.0 | Villages and farmlands | Good |
| 1691 | Baise | Jingxi City | Moraceae | *Ficus virens* Aiton. | 600 | 22.0 | 327.9 | 15.5 | Villages and farmlands | Good |
| 1692 | Baise | Jingxi City | Moraceae | *Ficus virens* Aiton. | 500 | 19.0 | 280.0 | 23.0 | Villages and farmlands | Good |
| 1693 | Baise | Jingxi City | Malvaceae | *Excentrodendron tonkinense* (A. Chev.) H. T. Chang & R. H. Miao | 800 | 18.0 | 168.7 | 15.0 | Villages and farmlands | Good |
| 1694 | Baise | Jingxi City | Malvaceae | *Excentrodendron tonkinense* (A. Chev.) H. T. Chang & R. H. Miao | 750 | 32.5 | 154.4 | 38.0 | Villages and farmlands | Good |
| 1695 | Baise | Jingxi City | Moraceae | *Ficus virens* Aiton. | 500 | 24.0 | 270.6 | 22.5 | Villages and farmlands | Good |
| 1696 | Baise | Jingxi City | Moraceae | *Ficus virens* Aiton. | 600 | 23.0 | 305.6 | 22.3 | Villages and farmlands | Good |
| 1697 | Baise | Jingxi City | Sapindaceae | *Aesculus assamica* Griff. | 600 | 13.0 | 155.0 | 19.5 | Villages and farmlands | Good |
| 1698 | Baise | Jingxi City | Moraceae | *Ficus virens* Aiton. | 650 | 35.0 | 327.8 | 34.0 | Villages and farmlands | Good |
| 1699 | Baise | Jingxi City | Moraceae | *Ficus virens* Aiton. | 500 | 35.0 | 280.0 | 45.0 | Villages and farmlands | Good |
| 1700 | Baise | Jingxi City | Moraceae | *Ficus hookeriana* Corner. | 750 | 28.0 | 356.5 | 49.3 | Villages and farmlands | Good |
| 1701 | Baise | Jingxi City | Moraceae | *Ficus virens* Aiton. | 600 | 25.0 | 324.8 | 50.0 | Villages and farmlands | Good |
| 1702 | Baise | Jingxi City | Moraceae | *Ficus virens* Aiton. | 600 | 30.0 | 308.0 | 43.0 | Villages and farmlands | Good |
| 1703 | Baise | Jingxi City | Moraceae | *Ficus virens* Aiton. | 550 | 19.5 | 280.1 | 20.0 | Villages and farmlands | Good |
| 1704 | Baise | Jingxi City | Ulmaceae | *Zelkova schneideriana* Hand.-Mazz. | 550 | 41.0 | 187.8 | 27.0 | Villages and farmlands | Good |
| 1705 | Baise | Jingxi City | Moraceae | *Ficus virens* Aiton. | 800 | 30.0 | 390.0 | 60.0 | Villages and farmlands | Good |
| 1706 | Baise | Jingxi City | Moraceae | *Ficus virens* Aiton. | 550 | 28.0 | 290.0 | 25.0 | Villages and farmlands | Good |
| 1707 | Baise | Jingxi City | Moraceae | *Ficus virens* Aiton. | 650 | 37.0 | 318.0 | 40.0 | Villages and farmlands | Good |
| 1708 | Baise | Jingxi City | Moraceae | *Ficus microcarpa* L. f. | 500 | 25.0 | 254.0 | 32.5 | Villages and farmlands | Good |
| 1709 | Baise | Jingxi City | Moraceae | *Ficus virens* Aiton. | 650 | 37.0 | 318.0 | 38.0 | Villages and farmlands | Good |
| 1710 | Baise | Jingxi City | Sapindaceae | *Litchi chinensis* Sonn. | 500 | 25.0 | 181.3 | 16.5 | Villages and farmlands | Moderate |
| 1711 | Baise | Jingxi City | Sapindaceae | *Litchi chinensis* Sonn. | 600 | 30.0 | 198.0 | 25.0 | Villages and farmlands | Moderate |
| 1712 | Baise | Jingxi City | Fabaceae | *Saraca dives* Pierre. | 500 | 28.0 | 160.0 | 13.5 | Villages and farmlands | Good |
| 1713 | Baise | Jingxi City | Fabaceae | *Saraca dives* Pierre. | 600 | 30.0 | 175.0 | 15.0 | Villages and farmlands | Good |
| 1714 | Baise | Napo County | Lauraceae | *Phoebe sheareri* (Hemsl.) Gamble | 500 | 30.0 | 130.0 | 17.0 | Villages and farmlands | Moderate |
| 1715 | Baise | Napo County | Lauraceae | *Phoebe calcarea* S. K. Lee & F. N. Wei | 500 | 25.0 | 160.0 | 18.0 | Villages and farmlands | Moderate |
| 1716 | Baise | Napo County | Malvaceae | *Excentrodendron tonkinense* (A. Chev.) H. T. Chang & R. H. Miao | 700 | 28.0 | 150.0 | 14.0 | Villages and farmlands | Good |
| 1717 | Baise | Napo County | Malvaceae | *Excentrodendron tonkinense* (A. Chev.) H. T. Chang & R. H. Miao | 500 | 22.0 | 120.0 | 13.0 | Villages and farmlands | Good |
| 1718 | Baise | Napo County | Moraceae | *Ficus altissima* Blume. | 600 | 25.0 | 288.0 | 36.0 | Villages and farmlands | Good |
| 1719 | Baise | Napo County | Moraceae | *Ficus virens* Aiton. | 500 | 20.0 | 320.0 | 45.0 | Villages and farmlands | Good |
| 1720 | Baise | Napo County | Fagaceae | *Lithocarpus corneus* (Lour.) Rehder | 500 | 36.0 | 150.0 | 24.0 | Villages and farmlands | Good |
| 1721 | Baise | Napo County | Fagaceae | *Lithocarpus corneus* (Lour.) Rehder | 550 | 45.0 | 159.0 | 27.0 | Villages and farmlands | Good |
| 1722 | Baise | Napo County | Lauraceae | *Phoebe calcarea* S. K. Lee & F. N. Wei | 500 | 35.0 | 144.0 | 15.0 | Villages and farmlands | Moderate |
| 1723 | Baise | Napo County | Moraceae | *Ficus altissima* Blume. | 500 | 24.0 | 280.0 | 36.5 | Villages and farmlands | Good |
| 1724 | Baise | Napo County | Lauraceae | *Phoebe calcarea* S. K. Lee & F. N. Wei | 500 | 26.0 | 110.0 | 10.9 | Villages and farmlands | Moderate |
| 1725 | Baise | Napo County | Rosaceae | *Prunus dulcis (M*ill.) D. A. Webb | 500 | 35.0 | 197.0 | 30.0 | Villages and farmlands | Moderate |
| 1726 | Baise | Napo County | Moraceae | *Ficus altissima* Blume. | 500 | 25.0 | 280.0 | 35.0 | Villages and farmlands | Good |
| 1727 | Baise | Napo County | Moraceae | *Ficus altissima* Blume. | 550 | 28.0 | 280.0 | 35.5 | Villages and farmlands | Good |
| 1728 | Baise | Lingyun County | Moraceae | *Ficus microcarpa* L. f. | 500 | 25.0 | 256.9 | 18.0 | Residential districts | Good |
| 1729 | Baise | Lingyun County | Sapindaceae | *Dimocarpus longan* Lour. | 550 | 13.0 | 113.5 | 7.5 | Villages and farmlands | Good |
| 1730 | Baise | Lingyun County | Moraceae | *Ficus virens* Aiton. | 500 | 34.0 | 275.8 | 17.5 | Villages and farmlands | Good |
| 1731 | Baise | Lingyun County | Moraceae | *Ficus virens* Aiton. | 750 | 30.0 | 347.6 | 32.5 | Villages and farmlands | Good |
| 1732 | Baise | Lingyun County | Fagaceae | *Castanopsis chinensis* (Spreng.) Hance | 900 | 20.0 | 190.0 | 7.5 | Villages and farmlands | Good |
| 1733 | Baise | Lingyun County | Juglandaceae | *Carya sinensis* Dode. | 600 | 23.0 | 162.4 | 12.0 | Villages and farmlands | Poor |
| 1734 | Baise | Lingyun County | Fagaceae | *Quercus disciformis* Chun & Tsiang | 900 | 15.0 | 207.0 | 12.0 | Villages and farmlands | Moderate |
| 1735 | Baise | Lingyun County | Fagaceae | *Quercus glauca* Thunb. | 630 | 25.0 | 162.0 | 13.0 | Villages and farmlands | Moderate |
| 1736 | Baise | Lingyun County | Moraceae | *Ficus virens* Aiton. | 550 | 25.0 | 287.0 | 20.0 | Villages and farmlands | Good |
| 1737 | Baise | Lingyun County | Moraceae | *Ficus virens* Aiton. | 650 | 30.0 | 328.0 | 22.5 | Villages and farmlands | Moderate |
| 1738 | Baise | Lingyun County | Lauraceae | *Litsea glutinosa* (Lour.) C. B. Rob. | 500 | 11.0 | 127.0 | 10.0 | Villages and farmlands | Poor |
| 1739 | Baise | Lingyun County | Moraceae | *Ficus virens* Aiton. | 500 | 27.0 | 275.0 | 28.0 | Villages and farmlands | Moderate |
| 1740 | Baise | Lingyun County | Moraceae | *Ficus virens* Aiton. | 520 | 25.0 | 280.0 | 21.0 | Villages and farmlands | Good |
| 1741 | Baise | Lingyun County | Meliaceae | *Aglaia lawii* (Wight) C. J. Saldanha & Ramamorthy | 500 | 20.0 | 115.0 | 9.0 | Villages and farmlands | Poor |
| 1742 | Baise | Lingyun County | Fagaceae | *Quercus glauca* Thunb. | 650 | 20.0 | 169.0 | 14.0 | Villages and farmlands | Moderate |
| 1743 | Baise | Lingyun County | Moraceae | *Ficus virens* Aiton. | 650 | 21.0 | 322.0 | 18.0 | Villages and farmlands | Good |
| 1744 | Baise | Lingyun County | Moraceae | *Ficus virens* Aiton. | 800 | 27.0 | 366.0 | 43.0 | Villages and farmlands | Good |
| 1745 | Baise | Lingyun County | Moraceae | *Ficus virens* Aiton. | 650 | 35.0 | 337.5 | 22.5 | Villages and farmlands | Good |
| 1746 | Baise | Lingyun County | Moraceae | *Ficus virens* Aiton. | 500 | 27.0 | 300.0 | 9.5 | Villages and farmlands | Good |
| 1747 | Baise | Lingyun County | Moraceae | *Ficus virens* Aiton. | 500 | 30.0 | 278.6 | 25.0 | Villages and farmlands | Good |
| 1748 | Baise | Lingyun County | Moraceae | *Ficus virens* Aiton. | 550 | 27.0 | 286.6 | 17.5 | Villages and farmlands | Moderate |
| 1749 | Baise | Lingyun County | Moraceae | *Ficus virens* Aiton. | 550 | 20.0 | 303.0 | 29.0 | Villages and farmlands | Good |
| 1750 | Baise | Lingyun County | Fagaceae | *Quercus acutissima* Carruth. | 700 | 18.0 | 124.0 | 8.5 | Villages and farmlands | Moderate |
| 1751 | Baise | Lingyun County | Altingiaceae | *Liquidambar formosana* Hance | 550 | 30.0 | 207.0 | 17.0 | Villages and farmlands | Good |
| 1752 | Baise | Lingyun County | Moraceae | *Ficus virens* Aiton. | 500 | 20.0 | 350.0 | 22.0 | Villages and farmlands | Good |
| 1753 | Baise | Lingyun County | Fagaceae | *Quercus glauca* Thunb. | 500 | 20.0 | 130.5 | 19.0 | Villages and farmlands | Moderate |
| 1754 | Baise | Lingyun County | Moraceae | *Ficus microcarpa* L. f. | 800 | 33.0 | 300.0 | 23.5 | Villages and farmlands | Good |
| 1755 | Baise | Leye County | Cephalotaxaceae | *Cephalotaxus hainanensis* H. L. Li | 850 | 18.8 | 141.3 | 8.9 | Residential districts | Poor |
| 1756 | Baise | Leye County | Juglandaceae | *Carya sinensis* Dode. | 700 | 35.0 | 129.0 | 17.9 | Villages and farmlands | Good |
| 1757 | Baise | Leye County | Juglandaceae | *Carya sinensis* Dode. | 800 | 36.0 | 216.6 | 16.8 | Villages and farmlands | Good |
| 1758 | Baise | Leye County | Magnoliaceae | *Manglietia aromatica* Dandy | 530 | 25.0 | 182.0 | 23.0 | Villages and farmlands | Moderate |
| 1759 | Baise | Leye County | Fagaceae | *Castanopsis carlesii* (Hemsl.) Hayata | 550 | 32.0 | 147.4 | 9.5 | Villages and farmlands | Moderate |
| 1760 | Baise | Leye County | Fagaceae | *Castanopsis carlesii* (Hemsl.) Hayata | 510 | 26.5 | 132.4 | 12.5 | Villages and farmlands | Good |
| 1761 | Baise | Leye County | Pinaceae | *Keteleeria davidiana* (C. E. Bertrand) Beissn. | 510 | 26.5 | 161.1 | 18.5 | Villages and farmlands | Moderate |
| 1762 | Baise | Leye County | Elaeocarpaceae | *Elaeocarpus sylvestris* (Lour.) Poir. | 500 | 32.0 | 165.8 | 12.5 | Villages and farmlands | Good |
| 1763 | Baise | Leye County | Fagaceae | *Quercus variabilis* Blume | 500 | 28.0 | 105.5 | 8.5 | Villages and farmlands | Poor |
| 1764 | Baise | Leye County | Moraceae | *Ficus virens* Aiton. | 900 | 45.0 | 380.0 | 17.5 | Villages and farmlands | Good |
| 1765 | Baise | Leye County | Moraceae | *Ficus virens* Aiton. | 520 | 40.0 | 286.0 | 18.0 | Villages and farmlands | Good |
| 1766 | Baise | Leye County | Moraceae | *Ficus virens* Aiton. | 600 | 37.3 | 300.0 | 34.5 | Villages and farmlands | Good |
| 1767 | Baise | Leye County | Moraceae | *Ficus virens* Aiton. | 550 | 38.9 | 289.0 | 29.8 | Villages and farmlands | Good |
| 1768 | Baise | Leye County | Moraceae | *Ficus virens* Aiton. | 600 | 35.8 | 296.2 | 41.1 | Villages and farmlands | Good |
| 1769 | Baise | Leye County | Moraceae | *Ficus virens* Aiton. | 550 | 29.5 | 280.2 | 42.2 | Villages and farmlands | Good |
| 1770 | Baise | Leye County | Lauraceae | *Lindera megaphylla* Hemsl. | 700 | 37.8 | 181.5 | 30.5 | Villages and farmlands | Moderate |
| 1771 | Baise | Leye County | Pinaceae | *Keteleeria david*iana (C. E. Bertrand) Beissn. | 700 | 35.0 | 168.0 | 19.5 | Villages and farmlands | Good |
| 1772 | Baise | Leye County | Fagaceae | *Castanopsis hystrix* Hook. f. & Thomson ex A. DC. | 650 | 20.0 | 163.4 | 7.5 | Wooded areas and plant nurseries | Moderate |
| 1773 | Baise | Leye County | Fagaceae | *Quercus fabri* Hance. | 600 | 12.0 | 165.0 | 8.5 | Wooded areas and plant nurseries | Moderate |
| 1774 | Baise | Leye County | Lauraceae | *Machilus wenshanensis* H. W. Li | 500 | 35.0 | 168.0 | 20.0 | Wooded areas and plant nurseries | Good |
| 1775 | Baise | Tianlin County | Fabaceae | *Lysidice rhodostegia* Hance. | 530 | 23.0 | 156.0 | 19.0 | Villages and farmlands | Good |
| 1776 | Baise | Tianlin County | Cannabaceae | *Celtis sinensis* Pers. | 520 | 20.0 | 150.0 | 18.5 | Villages and farmlands | Good |
| 1777 | Baise | Tianlin County | Moraceae | *Ficus microcarpa* L. f. | 530 | 25.6 | 240.0 | 26.5 | Villages and farmlands | Good |
| 1778 | Baise | Tianlin County | Moraceae | *Ficus virens* Aiton. | 700 | 18.4 | 304.0 | 24.5 | Villages and farmlands | Moderate |
| 1779 | Baise | Tianlin County | Moraceae | *Ficus virens* Aiton. | 500 | 30.0 | 159.2 | 33.5 | Residential districts | Good |
| 1780 | Baise | Tianlin County | Moraceae | *Ficus microcarpa* L. f. | 580 | 23.0 | 200.0 | 37.0 | Villages and farmlands | Good |
| 1781 | Baise | Tianlin County | Phyllanthaceae | *Bischofia javanica* Blume | 550 | 24.0 | 215.0 | 29.0 | Villages and farmlands | Good |
| 1782 | Baise | Tianlin County | Moraceae | *Ficus microcarpa* L. f. | 550 | 35.0 | 280.0 | 39.0 | Villages and farmlands | Good |
| 1783 | Baise | Tianlin County | Moraceae | *Ficus virens* Aiton. | 510 | 23.0 | 230.0 | 26.0 | Villages and farmlands | Good |
| 1784 | Baise | Tianlin County | Sapindaceae | *Dimocarpus longan* Lour. | 520 | 15.0 | 103.0 | 14.0 | Villages and farmlands | Good |
| 1785 | Baise | Tianlin County | Sapindaceae | *Dimocarpus longan* Lour. | 550 | 13.0 | 110.0 | 8.0 | Villages and farmlands | Moderate |
| 1786 | Baise | Tianlin County | Altingiaceae | *Liquidambar formosana* Hance | 500 | 38.0 | 160.0 | 18.0 | Villages and farmlands | Poor |
| 1787 | Baise | Tianlin County | Sapindaceae | *Dimocarpus longan* Lour. | 550 | 15.0 | 110.0 | 14.0 | Villages and farmlands | Good |
| 1788 | Baise | Tianlin County | Moraceae | *Ficus virens* Aiton. | 520 | 22.0 | 230.0 | 22.0 | Villages and farmlands | Good |
| 1789 | Baise | Tianlin County | Moraceae | *Ficus virens* Aiton. | 600 | 18.0 | 300.0 | 16.0 | Villages and farmlands | Moderate |
| 1790 | Baise | Tianlin County | Moraceae | *Ficus virens* Aiton. | 600 | 15.0 | 290.0 | 12.0 | Villages and farmlands | Moderate |
| 1791 | Baise | Tianlin County | Moraceae | *Ficus virens* Aiton. | 500 | 30.0 | 250.0 | 16.0 | Villages and farmlands | Good |
| 1792 | Baise | Tianlin County | Moraceae | *Ficus virens* Aiton. | 500 | 28.0 | 365.0 | 15.0 | Villages and farmlands | Good |
| 1793 | Baise | Tianlin County | Moraceae | *Ficus virens* Aiton. | 530 | 35.0 | 320.0 | 25.5 | Villages and farmlands | Good |
| 1794 | Baise | Tianlin County | Moraceae | *Ficus virens* Aiton. | 650 | 28.0 | 300.0 | 26.3 | Villages and farmlands | Good |
| 1795 | Baise | Tianlin County | Moraceae | *Ficus virens* Aiton. | 560 | 26.0 | 260.0 | 29.5 | Villages and farmlands | Good |
| 1796 | Baise | Tianlin County | Moraceae | *Ficus virens* Aiton. | 540 | 25.0 | 248.4 | 29.0 | Villages and farmlands | Good |
| 1797 | Baise | Tianlin County | Moraceae | *Ficus virens* Aiton. | 500 | 38.0 | 465.0 | 32.5 | Villages and farmlands | Good |
| 1798 | Baise | Tianlin County | Moraceae | *Ficus virens* Aiton. | 580 | 32.0 | 260.0 | 24.5 | Villages and farmlands | Good |
| 1799 | Baise | Tianlin County | Anacardiaceae | *Pistacia chinensis* Bunge | 650 | 28.4 | 73.2 | 12.0 | Villages and farmlands | Moderate |
| 1800 | Baise | Tianlin County | Fagaceae | *Castanopsis chinensis (*Spreng.) Hance | 520 | 18.4 | 138.0 | 10.9 | Villages and farmlands | Good |
| 1801 | Baise | Tianlin County | Fagaceae | *Castanopsis chinensis* (Spreng.) Hance | 520 | 16.3 | 134.0 | 8.1 | Villages and farmlands | Good |
| 1802 | Baise | Tianlin County | Fagaceae | *Castanopsis chinensis* (Spreng.) Hance | 550 | 13.6 | 151.0 | 7.2 | Villages and farmlands | Good |
| 1803 | Baise | Tianlin County | Anacardiaceae | *Pistacia chinensis* Bunge | 520 | 22.6 | 107.4 | 10.8 | Villages and farmlands | Moderate |
| 1804 | Baise | Tianlin County | Moraceae | *Ficus virens* Aiton. | 760 | 24.8 | 297.1 | 11.3 | Villages and farmlands | Moderate |
| 1805 | Baise | Tianlin County | Moraceae | *Ficus microcarpa* L. f. | 750 | 24.8 | 297.1 | 11.0 | Villages and farmlands | Good |
| 1806 | Baise | Tianlin County | Ulmaceae | *Zelkova schneideriana* Hand.-Mazz. | 520 | 20.6 | 123.8 | 11.0 | Villages and farmlands | Good |
| 1807 | Baise | Tianlin County | Ulmaceae | *Zelkova schneideriana* Hand.-Mazz. | 520 | 21.6 | 132.4 | 11.1 | Villages and farmlands | Good |
| 1808 | Baise | Tianlin County | Ulmaceae | *Ulmus parvifolia* Jacq. | 520 | 19.8 | 121.6 | 10.4 | Villages and farmlands | Good |
| 1809 | Baise | Tianlin County | Cannabaceae | *Celtis sinensis* Pers. | 550 | 17.3 | 175.0 | 14.5 | Villages and farmlands | Good |
| 1810 | Baise | Tianlin County | Moraceae | *Ficus microcarpa* L. f. | 560 | 20.0 | 257.1 | 26.5 | Villages and farmlands | Good |
| 1811 | Baise | Tianlin County | Moraceae | *Ficus microcarpa* L. f. | 520 | 25.0 | 238.8 | 34.5 | Villages and farmlands | Good |
| 1812 | Baise | Tianlin County | Moraceae | *Ficus microcarpa* L. f. | 510 | 25.0 | 257.5 | 25.0 | Villages and farmlands | Good |
| 1813 | Baise | Tianlin County | Moraceae | *Ficus virens* Aiton. | 550 | 18.0 | 273.8 | 26.0 | Villages and farmlands | Good |
| 1814 | Baise | Tianlin County | Moraceae | *Ficus microcarpa* L. f. | 630 | 20.0 | 277.1 | 26.0 | Villages and farmlands | Good |
| 1815 | Baise | Tianlin County | Ulmaceae | *Ulmus parvifolia* Jacq. | 530 | 18.0 | 114.6 | 12.4 | Villages and farmlands | Good |
| 1816 | Baise | Tianlin County | Moraceae | *Ficus virens* Aiton. | 550 | 22.5 | 360.2 | 35.6 | Villages and farmlands | Good |
| 1817 | Baise | Tianlin County | Moraceae | *Ficus virens* Aiton. | 550 | 25.0 | 261.1 | 18.0 | Villages and farmlands | Good |
| 1818 | Baise | Tianlin County | Moraceae | *Ficus virens* Aiton. | 550 | 18.0 | 266.3 | 15.5 | Villages and farmlands | Good |
| 1819 | Baise | Tianlin County | Moraceae | *Ficus virens* Aiton. | 530 | 18.4 | 288.1 | 27.0 | Villages and farmlands | Good |
| 1820 | Baise | Tianlin County | Moraceae | *Ficus virens* Aiton. | 540 | 23.0 | 362.0 | 19.0 | Villages and farmlands | Good |
| 1821 | Baise | Tianlin County | Moraceae | *Ficus virens* Aiton. | 550 | 22.0 | 296.6 | 22.5 | Villages and farmlands | Good |
| 1822 | Baise | Tianlin County | Moraceae | *Ficus virens* Aiton. | 530 | 24.0 | 245.3 | 18.5 | Villages and farmlands | Good |
| 1823 | Baise | Tianlin County | Moraceae | *Ficus virens* Aiton. | 520 | 30.0 | 243.4 | 20.0 | Villages and farmlands | Good |
| 1824 | Baise | Tianlin County | Moraceae | *Ficus virens* Aiton. | 520 | 23.0 | 245.8 | 23.5 | Villages and farmlands | Good |
| 1825 | Baise | Tianlin County | Moraceae | *Ficus virens* Aiton. | 550 | 25.3 | 321.2 | 22.4 | Villages and farmlands | Good |
| 1826 | Baise | Tianlin County | Moraceae | *Ficus microcarpa* L. f. | 650 | 20.0 | 278.5 | 27.5 | Villages and farmlands | Good |
| 1827 | Baise | Tianlin County | Moraceae | *Ficus virens* Aiton. | 580 | 18.0 | 258.1 | 12.6 | Villages and farmlands | Good |
| 1828 | Baise | Tianlin County | Moraceae | *Ficus virens* Aiton. | 530 | 26.0 | 262.1 | 23.5 | Villages and farmlands | Good |
| 1829 | Baise | Tianlin County | Moraceae | *Ficus virens* Aiton. | 550 | 24.0 | 280.0 | 30.0 | Villages and farmlands | Good |
| 1830 | Baise | Tianlin County | Moraceae | *Ficus virens* Aiton. | 600 | 50.0 | 280.0 | 67.5 | Villages and farmlands | Good |
| 1831 | Baise | Tianlin County | Moraceae | *Ficus virens* Aiton. | 600 | 50.0 | 350.0 | 77.5 | Villages and farmlands | Good |
| 1832 | Baise | Tianlin County | Moraceae | *Ficus virens* Aiton. | 500 | 27.0 | 254.7 | 36.0 | Villages and farmlands | Good |
| 1833 | Baise | Tianlin County | Moraceae | *Ficus virens* Aiton. | 520 | 30.0 | 318.0 | 41.0 | Villages and farmlands | Good |
| 1834 | Baise | Tianlin County | Moraceae | *Ficus microcarpa* L. f. | 510 | 35.0 | 232.0 | 31.5 | Villages and farmlands | Good |
| 1835 | Baise | Tianlin County | Moraceae | *Ficus virens* Aiton. | 530 | 45.0 | 350.0 | 32.5 | Villages and farmlands | Good |
| 1836 | Baise | Tianlin County | Moraceae | *Ficus microcarpa* L. f. | 520 | 18.0 | 260.0 | 25.5 | Villages and farmlands | Good |
| 1837 | Baise | Tianlin County | Fagaceae | *Castanopsis carlesii* (Hemsl.) Hayata | 510 | 21.0 | 137.0 | 17.0 | Villages and farmlands | Good |
| 1838 | Baise | Tianlin County | Sapindaceae | *Handeliodendron bodinieri* (H. Lév.) Rehder | 510 | 18.0 | 78.0 | 6.0 | Villages and farmlands | Good |
| 1839 | Baise | Tianlin County | Olacaceae | *Malania oleifera* Chun & S. K. Lee | 500 | 38.0 | 66.9 | 20.0 | Villages and farmlands | Good |
| 1840 | Baise | Tianlin County | Lauraceae | *Machilus rehder* iC. K. Allen | 510 | 20.0 | 169.4 | 29.0 | Villages and farmlands | Good |
| 1841 | Baise | Tianlin County | Moraceae | *Ficus virens* Aiton. | 750 | 5.0 | 295.0 | 37.0 | Villages and farmlands | Good |
| 1842 | Baise | Tianlin County | Moraceae | *Ficus virens* Aiton. | 510 | 23.0 | 240.0 | 24.0 | Villages and farmlands | Good |
| 1843 | Baise | Tianlin County | Moraceae | *Ficus virens* Aiton. | 510 | 27.0 | 250.0 | 36.0 | Villages and farmlands | Good |
| 1844 | Baise | Xilin County | Moraceae | *Ficus virens* Aiton. | 550 | 21.6 | 267.0 | 25.0 | Villages and farmlands | Good |
| 1845 | Baise | Xilin County | Moraceae | *Ficus virens* Aiton. | 520 | 22.1 | 267.2 | 35.3 | Villages and farmlands | Good |
| 1846 | Baise | Xilin County | Moraceae | *Ficus virens* Aiton. | 550 | 21.3 | 274.0 | 24.2 | Villages and farmlands | Good |
| 1847 | Baise | Xilin County | Moraceae | *Ficus virens* Aiton. | 650 | 25.0 | 281.0 | 35.0 | Villages and farmlands | Good |
| 1848 | Baise | Xilin County | Moraceae | *Ficus virens* Aiton. | 500 | 21.0 | 264.0 | 24.5 | Villages and farmlands | Good |
| 1849 | Baise | Xilin County | Moraceae | *Ficus virens* Aiton. | 600 | 21.0 | 300.0 | 29.0 | Villages and farmlands | Good |
| 1850 | Baise | Xilin County | Moraceae | *Ficus virens* Aiton. | 600 | 25.0 | 300.0 | 23.8 | Villages and farmlands | Good |
| 1851 | Baise | Xilin County | Moraceae | *Ficus microcarpa* L. f. | 650 | 20.0 | 288.0 | 31.5 | Villages and farmlands | Good |
| 1852 | Baise | Xilin County | Moraceae | *Ficus microcarpa* L. f. | 600 | 23.0 | 320.0 | 25.5 | Villages and farmlands | Good |
| 1853 | Baise | Xilin County | Moraceae | *Ficus microcarpa* L. f. | 600 | 25.0 | 286.0 | 34.0 | Villages and farmlands | Good |
| 1854 | Baise | Xilin County | Moraceae | *Ficus virens* Aiton. | 620 | 26.0 | 321.0 | 30.5 | Villages and farmlands | Good |
| 1855 | Baise | Xilin County | Altingiaceae | *Liquidambar formosana* Hance | 500 | 33.0 | 200.0 | 11.0 | Villages and farmlands | Good |
| 1856 | Baise | Xilin County | Ulmaceae | *Zelkova schneideriana* Hand.-Mazz. | 800 | 26.0 | 96.0 | 22.0 | Villages and farmlands | Good |
| 1857 | Baise | Xilin County | Moraceae | *Ficus virens* Aiton. | 620 | 32.0 | 276.0 | 32.0 | Villages and farmlands | Good |
| 1858 | Baise | Longlin County | Moraceae | *Ficus virens* Aiton. | 580 | 17.0 | 302.0 | 27.0 | Villages and farmlands | Good |
| 1859 | Baise | Longlin County | Moraceae | *Ficus virens* Aiton. | 680 | 23.0 | 331.0 | 41.0 | Villages and farmlands | Good |
| 1860 | Baise | Longlin County | Moraceae | *Ficus virens* Aiton. | 800 | 25.0 | 359.0 | 33.5 | Villages and farmlands | Good |
| 1861 | Baise | Longlin County | Moraceae | *Ficus virens* Aiton. | 525 | 20.0 | 286.0 | 28.0 | Villages and farmlands | Good |
| 1862 | Baise | Longlin County | Moraceae | *Ficus virens* Aiton. | 510 | 24.0 | 277.0 | 42.5 | Villages and farmlands | Good |
| 1863 | Baise | Longlin County | Moraceae | *Ficus virens* Aiton. | 510 | 18.0 | 283.0 | 15.0 | Villages and farmlands | Good |
| 1864 | Baise | Longlin County | Moraceae | *Ficus virens* Aiton. | 700 | 28.0 | 334.0 | 33.0 | Villages and farmlands | Good |
| 1865 | Baise | Longlin County | Moraceae | *Ficus virens* Aiton. | 670 | 18.0 | 327.9 | 38.0 | Villages and farmlands | Good |
| 1866 | Baise | Longlin County | Moraceae | *Ficus virens* Aiton. | 530 | 22.0 | 280.1 | 23.0 | Villages and farmlands | Good |
| 1867 | Baise | Longlin County | Moraceae | *Ficus virens* Aiton. | 750 | 23.0 | 350.0 | 21.0 | Villages and farmlands | Good |
| 1868 | Baise | Longlin County | Moraceae | *Ficus virens* Aiton. | 520 | 20.0 | 289.0 | 39.0 | Villages and farmlands | Good |
| 1869 | Baise | Longlin County | Moraceae | *Ficus virens* Aiton. | 720 | 15.0 | 337.0 | 18.0 | Villages and farmlands | Good |
| 1870 | Baise | Longlin County | Moraceae | *Ficus virens* Aiton. | 730 | 20.0 | 337.0 | 23.0 | Villages and farmlands | Good |
| 1871 | Baise | Longlin County | Moraceae | *Ficus virens* Aiton. | 510 | 29.0 | 286.0 | 35.0 | Villages and farmlands | Good |
| 1872 | Baise | Longlin County | Moraceae | *Ficus virens* Aiton. | 736 | 25.0 | 344.0 | 35.0 | Villages and farmlands | Good |
| 1873 | Baise | Longlin County | Moraceae | *Ficus virens* Aiton. | 685 | 25.0 | 331.0 | 36.0 | Villages and farmlands | Good |
| 1874 | Baise | Longlin County | Moraceae | *Ficus virens* Aiton. | 550 | 19.0 | 293.0 | 36.0 | Villages and farmlands | Good |
| 1875 | Baise | Longlin County | Moraceae | *Ficus virens* Aiton. | 900 | 20.0 | 380.0 | 38.0 | Villages and farmlands | Good |
| 1876 | Baise | Longlin County | Moraceae | *Ficus virens* Aiton. | 646 | 23.0 | 318.0 | 22.0 | Villages and farmlands | Good |
| 1877 | Baise | Longlin County | Moraceae | *Ficus virens* Aiton. | 910 | 14.0 | 382.0 | 43.0 | Villages and farmlands | Good |
| 1878 | Baise | Longlin County | Moraceae | *Ficus virens* Aiton. | 762 | 15.0 | 350.0 | 35.0 | Villages and farmlands | Good |
| 1879 | Baise | Longlin County | Cannabaceae | *Celtis sinensis* Pers. | 860 | 25.0 | 207.0 | 16.0 | Villages and farmlands | Good |
| 1880 | Baise | Longlin County | Moraceae | *Ficus virens* Aiton. | 530 | 20.0 | 286.5 | 28.0 | Villages and farmlands | Good |
| 1881 | Baise | Longlin County | Moraceae | *Ficus virens* Aiton. | 500 | 18.0 | 276.9 | 19.0 | Villages and farmlands | Good |
| 1882 | Baise | Longlin County | Moraceae | *Ficus virens* Aiton. | 800 | 25.0 | 360.3 | 45.7 | Villages and farmlands | Good |
| 1883 | Baise | Longlin County | Moraceae | *Ficus virens* Aiton. | 590 | 23.0 | 305.6 | 29.0 | Villages and farmlands | Good |
| 1884 | Baise | Longlin County | Moraceae | *Ficus virens* Aiton. | 850 | 14.0 | 375.6 | 31.0 | Villages and farmlands | Good |
| 1885 | Baise | Longlin County | Moraceae | *Ficus virens* Aiton. | 550 | 18.0 | 289.7 | 48.0 | Villages and farmlands | Good |
| 1886 | Baise | Longlin County | Moraceae | *Ficus virens* Aiton. | 530 | 20.0 | 280.0 | 11.0 | Villages and farmlands | Good |
| 1887 | Baise | Longlin County | Moraceae | *Ficus virens* Aiton. | 760 | 10.0 | 350.2 | 21.0 | Villages and farmlands | Good |
| 1888 | Baise | Longlin County | Moraceae | *Ficus virens* Aiton. | 640 | 15.0 | 318.3 | 28.0 | Villages and farmlands | Good |
| 1889 | Baise | Longlin County | Moraceae | *Ficus virens* Aiton. | 500 | 26.0 | 273.8 | 28.0 | Villages and farmlands | Good |
| 1890 | Baise | Longlin County | Moraceae | *Ficus virens* Aiton. | 540 | 26.0 | 286.5 | 34.0 | Villages and farmlands | Good |
| 1891 | Baise | Longlin County | Moraceae | *Ficus virens* Aiton. | 840 | 23.0 | 366.2 | 40.0 | Villages and farmlands | Good |
| 1892 | Baise | Longlin County | Anacardiaceae | *Pistacia chinensis* Bunge | 580 | 20.0 | 143.2 | 19.0 | Villages and farmlands | Good |
| 1893 | Baise | Longlin County | Moraceae | *Ficus virens* Aiton | 700 | 13.0 | 334.0 | 32.0 | Villages and farmlands | Good |
| 1894 | Baise | Longlin County | Fagaceae | *Castanopsis carlesii* (Hemsl.) Hayata | 500 | 25.0 | 136.8 | 32.0 | Villages and farmlands | Good |
| 1895 | Baise | Longlin County | Moraceae | *Ficus virens* Aiton. | 520 | 17.0 | 283.0 | 16.0 | Villages and farmlands | Good |
| 1896 | Baise | Longlin County | Moraceae | *Ficus virens* Aiton. | 560 | 29.0 | 296.0 | 27.5 | Villages and farmlands | Good |
| 1897 | Baise | Longlin County | Moraceae | *Ficus virens* Aiton. | 610 | 29.6 | 316.0 | 33.0 | Villages and farmlands | Good |
| 1898 | Baise | Longlin County | Moraceae | *Ficus virens* Aiton. | 920 | 28.0 | 385.0 | 28.0 | Villages and farmlands | Good |
| 1899 | Baise | Longlin County | Moraceae | *Ficus virens* Aiton. | 650 | 23.0 | 327.0 | 25.0 | Villages and farmlands | Good |
| 1900 | Baise | Longlin County | Moraceae | *Ficus virens* Aiton. | 550 | 20.0 | 292.9 | 31.0 | Villages and farmlands | Good |
| 1901 | Baise | Longlin County | Moraceae | *Ficus virens* Aiton. | 950 | 22.0 | 391.0 | 23.0 | Villages and farmlands | Good |
| 1902 | Baise | Longlin County | Moraceae | *Ficus virens* Aiton. | 660 | 26.0 | 321.0 | 26.0 | Villages and farmlands | Good |
| 1903 | Baise | Longlin County | Moraceae | *Ficus virens* Aiton. | 600 | 30.0 | 304.1 | 17.5 | Villages and farmlands | Good |
| 1904 | Baise | Longlin County | Moraceae | *Ficus virens* Aiton. | 620 | 17.0 | 315.1 | 17.0 | Villages and farmlands | Good |
| 1905 | Baise | Longlin County | Moraceae | *Ficus virens* Aiton. | 550 | 16.0 | 289.7 | 22.0 | Villages and farmlands | Good |
| 1906 | Baise | Longlin County | Phyllanthaceae | *Bischofia polycarpa* (H. Lév.) Airy Shaw | 640 | 26.0 | 235.6 | 31.0 | Villages and farmlands | Good |
| 1907 | Baise | Longlin County | Moraceae | *Ficus virens* Aiton. | 830 | 23.0 | 366.0 | 44.5 | Villages and farmlands | Good |
| 1908 | Baise | Longlin County | Moraceae | *Ficus virens* Aiton. | 730 | 23.0 | 350.1 | 23.5 | Villages and farmlands | Good |
| 1909 | Baise | Longlin County | Moraceae | *Ficus virens* Aiton. | 530 | 21.0 | 286.4 | 21.5 | Villages and farmlands | Good |
| 1910 | Baise | Longlin County | Moraceae | *Ficus virens* Aiton. | 760 | 27.0 | 350.0 | 34.5 | Villages and farmlands | Good |
| 1911 | Baise | Longlin County | Fagaceae | *Castanopsis hystrix* Hook. f. & Thomson ex A. DC. | 700 | 40.0 | 172.0 | 30.0 | Wooded areas and plant nurseries | Good |
| 1912 | Baise | Longlin County | Moraceae | *Ficus virens* Aiton. | 550 | 35.0 | 280.3 | 32.5 | Wooded areas and plant nurseries | Good |
| 1913 | Hezhou | Babu District | Lauraceae | *Camphora officinarum Nees.* | 1000 | 23.0 | 300.0 | 24.0 | Villages and farmlands | Good |
| 1914 | Hezhou | Babu District | Moraceae | *Ficus concinna* (Miq.) Miq. | 1000 | 13.0 | 315.0 | 22.5 | Villages and farmlands | Good |
| 1915 | Hezhou | Babu District | Moraceae | *Ficus concinna* (Miq.) Miq. | 1100 | 16.0 | 398.0 | 34.9 | Villages and farmlands | Good |
| 1916 | Hezhou | Babu District | Moraceae | *Ficus concinna* (Miq.) Miq. | 1000 | 15.0 | 391.0 | 33.4 | Villages and farmlands | Good |
| 1917 | Hezhou | Babu District | Moraceae | *Ficus concinna* (Miq.) Miq. | 1000 | 12.0 | 290.0 | 30.5 | Villages and farmlands | Good |
| 1918 | Hezhou | Babu District | Moraceae | *Ficus concinna* (Miq.) Miq. | 1000 | 18.0 | 302.0 | 28.5 | Villages and farmlands | Good |
| 1919 | Hezhou | Babu District | Moraceae | *Ficus concinna* (Miq.) Miq. | 1050 | 18.0 | 322.0 | 42.5 | Villages and farmlands | Good |
| 1920 | Hezhou | Babu District | Lauraceae | *Camphora officinarum Nees* | 1000 | 18.0 | 311.0 | 32.0 | Villages and farmlands | Good |
| 1921 | Hezhou | Babu District | Moraceae | *Ficus concinna* (Miq.) Miq. | 1050 | 23.0 | 369.0 | 35.0 | Villages and farmlands | Good |
| 1922 | Hezhou | Babu District | Oleaceae | *Osmanthus fragrans* (Thunb.) Lour. | 1200 | 14.0 | 115.0 | 16.5 | Villages and farmlands | Good |
| 1923 | Hezhou | Babu District | Lauraceae | *Camphora officinarum Nees.* | 1010 | 16.0 | 264.0 | 22.5 | Villages and farmlands | Poor |
| 1924 | Hezhou | Babu District | Moraceae | *Ficus concinna* (Miq.) Miq. | 1200 | 25.0 | 398.0 | 49.0 | Villages and farmlands | Good |
| 1925 | Hezhou | Babu District | Moraceae | *Ficus concinna* (Miq.) Miq. | 1000 | 25.0 | 366.0 | 50.0 | Villages and farmlands | Good |
| 1926 | Hezhou | Babu District | Oleaceae | *Osmanthus fragrans* (Thunb.) Lour. | 1000 | 12.0 | 105.0 | 21.0 | Villages and farmlands | Good |
| 1927 | Hezhou | Babu District | Oleaceae | *Osmanthus fragrans* (Thunb.) Lour. | 1000 | 13.0 | 105.0 | 14.0 | Villages and farmlands | Good |
| 1928 | Hezhou | Babu District | Oleaceae | *Ligustrum lucidum* W. T. Aiton. | 1500 | 20.0 | 162.0 | 15.0 | Villages and farmlands | Moderate |
| 1929 | Hezhou | Babu District | Fagaceae | *Castanopsis ca*r*lesii* (Hemsl.) Hayata. | 1000 | 14.0 | 220.0 | 17.0 | Villages and farmlands | Good |
| 1930 | Hezhou | Babu District | Cycadaceae | *Cycas szechuanensis* W. C. Cheng & L. K. Fu | 1010 | 4.0 | 32.0 | 5.2 | Villages and farmlands | Poor |
| 1931 | Hezhou | Pinggui District | Moraceae | *Ficus concinna* (Miq.) Miq. | 1000 | 14.0 | 299.2 | 37.0 | Residential districts | Moderate |
| 1932 | Hezhou | Pinggui District | Moraceae | *Ficus concinna* (Miq.) Miq. | 1000 | 15.0 | 311.9 | 27.0 | Villages and farmlands | Good |
| 1933 | Hezhou | Pinggui District | Moraceae | *Ficus concinna* (Miq.) Miq. | 1000 | 15.0 | 318.3 | 31.0 | Villages and farmlands | Good |
| 1934 | Hezhou | Pinggui District | Moraceae | *Ficus concinna* (Miq.) Miq. | 1030 | 17.0 | 321.5 | 38.0 | Villages and farmlands | Good |
| 1935 | Hezhou | Pinggui District | Moraceae | *Ficus concinna* (Miq.) Miq. | 1000 | 18.0 | 343.7 | 30.0 | Villages and farmlands | Good |
| 1936 | Hezhou | Pinggui District | Moraceae | *Ficus concinna* (Miq.) Miq. | 1000 | 17.0 | 356.5 | 33.5 | Villages and farmlands | Good |
| 1937 | Hezhou | Zhaoping County | Moraceae | *Ficus concinna* (Miq.) Miq. | 1000 | 20.0 | 324.0 | 30.0 | Villages and farmlands | Good |
| 1938 | Hezhou | Zhaoping County | Moraceae | *Ficus concinna* (Miq.) Miq. | 1100 | 18.0 | 344.0 | 34.5 | Villages and farmlands | Good |
| 1939 | Hezhou | Zhongshan County | Lauraceae | *Camphora officinarum Nees.* | 1000 | 17.0 | 331.2 | 22.0 | Villages and farmlands | Good |
| 1940 | Hezhou | Zhongshan County | Lauraceae | *Camphora officinarum Nees.* | 1000 | 15.0 | 296.2 | 27.0 | Villages and farmlands | Good |
| 1941 | Hezhou | Zhongshan County | Moraceae | *Ficus concinna* (Miq.) Miq. | 1200 | 19.0 | 321.7 | 35.5 | Villages and farmlands | Good |
| 1942 | Hezhou | Zhongshan County | Moraceae | *Ficus concinna* (Miq.) Miq. | 1000 | 16.0 | 312.1 | 31.5 | Villages and farmlands | Good |
| 1943 | Hezhou | Zhongshan County | Moraceae | *Ficus concinna* (Miq.) Miq. | 1000 | 20.0 | 350.3 | 42.5 | Villages and farmlands | Good |
| 1944 | Hezhou | Zhongshan County | Moraceae | *Ficus concinna* (Miq.) Miq. | 1100 | 15.0 | 343.9 | 34.0 | Villages and farmlands | Good |
| 1945 | Hezhou | Zhongshan County | Moraceae | *Ficus concinna* (Miq.) Miq. | 1000 | 28.0 | 337.0 | 46.0 | Villages and farmlands | Good |
| 1946 | Hezhou | Fuchuan County | Lauraceae | *Camphora officinarum Nees.* | 1850 | 22.0 | 435.0 | 21.5 | Villages and farmlands | Moderate |
| 1947 | Hezhou | Fuchuan County | Fabaceae | *Styphnolobium japonicum* (L.) Schott. | 1000 | 12.0 | 184.7 | 12.0 | Villages and farmlands | Good |
| 1948 | Hezhou | Fuchuan County | Sapindaceae | *Acer coriaceifolium* H. Lév. | 1100 | 22.0 | 109.6 | 13.0 | Villages and farmlands | Good |
| 1949 | Hezhou | Fuchuan County | Sapindaceae | *Acer coriaceifolium* H. Lév. | 1100 | 17.0 | 119.4 | 15.0 | Villages and farmlands | Good |
| 1950 | Hezhou | Fuchuan County | Lauraceae | *Camphora officinarum Nees.* | 1400 | 23.0 | 397.8 | 25.0 | Villages and farmlands | Good |
| 1951 | Hezhou | Fuchuan County | Sapindaceae | *Acer coriaceifolium* H. Lév. | 1100 | 20.0 | 111.1 | 16.0 | Villages and farmlands | Good |
| 1952 | Hezhou | Babu District | Lauraceae | *Camphora officinarum Nees.* | 500 | 24.0 | 220.0 | 20.5 | Residential districts | Good |
| 1953 | Hezhou | Babu District | Lauraceae | *Camphora officinarum Nees.* | 500 | 23.0 | 222.0 | 16.5 | Residential districts | Good |
| 1954 | Hezhou | Babu District | Lauraceae | *Camphora officinarum Nees.* | 625 | 20.0 | 276.0 | 51.5 | Residential districts | Good |
| 1955 | Hezhou | Babu District | Moraceae | *Ficus concinna (*Miq.) Miq. | 680 | 15.0 | 220.0 | 29.0 | Villages and farmlands | Good |
| 1956 | Hezhou | Babu District | Moraceae | *Ficus concinna (*Miq.) Miq. | 700 | 9.0 | 338.0 | 17.0 | Villages and farmlands | Moderate |
| 1957 | Hezhou | Babu District | Lauraceae | *Camphora officinarum Nees* | 720 | 23.0 | 282.0 | 23.5 | Villages and farmlands | Good |
| 1958 | Hezhou | Babu District | Moraceae | *Ficus concinna (*Miq.) Miq. | 580 | 18.0 | 266.0 | 24.5 | Villages and farmlands | Good |
| 1959 | Hezhou | Babu District | Fagaceae | *Castanopsis carlesii* (Hemsl.) Hayata | 600 | 22.0 | 160.0 | 18.0 | Villages and farmlands | Good |
| 1960 | Hezhou | Babu District | Moraceae | *Ficus concinna (*Miq.) Miq. | 580 | 22.0 | 270.0 | 41.0 | Villages and farmlands | Good |
| 1961 | Hezhou | Babu District | Oleaceae | *Osmanthus fragrans* (Thunb.) Lour. | 800 | 9.0 | 102.0 | 12.0 | Villages and farmlands | Good |
| 1962 | Hezhou | Babu District | Lauraceae | *Camphora officinarum Nees* | 650 | 18.0 | 265.0 | 19.0 | Villages and farmlands | Moderate |
| 1963 | Hezhou | Babu District | Fagaceae | *Castanopsis carlesii* (Hemsl.) Hayata | 557 | 20.0 | 147.0 | 20.5 | Villages and farmlands | Good |
| 1964 | Hezhou | Babu District | Fagaceae | *Castanopsis carlesii* (Hemsl.) Hayata | 551 | 25.0 | 146.0 | 23.0 | Villages and farmlands | Good |
| 1965 | Hezhou | Babu District | Fagaceae | *Castanopsis carlesii* (Hemsl.) Hayata | 550 | 24.0 | 143.0 | 19.0 | Villages and farmlands | Good |
| 1966 | Hezhou | Babu District | Lauraceae | *Camphora officinarum Nees* | 600 | 25.0 | 244.0 | 35.0 | Villages and farmlands | Moderate |
| 1967 | Hezhou | Babu District | Fagaceae | *Castanopsis hystrix* Hook. f. & Thomson ex A. DC. | 650 | 33.0 | 168.0 | 14.0 | Villages and farmlands | Good |
| 1968 | Hezhou | Babu District | Moraceae | *Ficus concinna (*Miq.) Miq. | 650 | 16.0 | 280.0 | 37.5 | Villages and farmlands | Good |
| 1969 | Hezhou | Babu District | Oleaceae | *Osmanthus fragrans* (Thunb.) Lour. | 700 | 15.0 | 100.0 | 18.0 | Villages and farmlands | Good |
| 1970 | Hezhou | Babu District | Lauraceae | *Camphora officinarum Nees* | 500 | 26.0 | 190.0 | 29.5 | Villages and farmlands | Good |
| 1971 | Hezhou | Babu District | Oleaceae | *Osmanthus fragrans* (Thunb.) Lour. | 850 | 15.0 | 102.0 | 19.0 | Villages and farmlands | Good |
| 1972 | Hezhou | Babu District | Rhamnaceae | *Hovenia acerba* Lindl. | 540 | 16.0 | 105.0 | 17.0 | Villages and farmlands | Good |
| 1973 | Hezhou | Babu District | Lauraceae | *Camphora officinarum Nees* | 520 | 18.0 | 223.0 | 24.5 | Villages and farmlands | Moderate |
| 1974 | Hezhou | Babu District | Lauraceae | *Camphora officinarum Nees* | 610 | 20.0 | 160.0 | 20.0 | Villages and farmlands | Moderate |
| 1975 | Hezhou | Babu District | Moraceae | *Ficus concinna* (Miq.) Miq. | 610 | 18.0 | 360.0 | 31.0 | Villages and farmlands | Moderate |
| 1976 | Hezhou | Babu District | Fagaceae | *Castanopsis hystrix* Hook. f. & Thomson ex A. DC. | 800 | 22.0 | 112.0 | 18.0 | Villages and farmlands | Good |
| 1977 | Hezhou | Babu District | Fagaceae | *Castanopsis carlesii*( Hemsl.) Hayata | 601 | 16.0 | 155.0 | 17.0 | Villages and farmlands | Good |
| 1978 | Hezhou | Babu District | Fagaceae | *Castanopsis hystrix* Hook. f. & Thomson ex A. DC. | 550 | 25.0 | 150.0 | 13.5 | Wooded areas and plant nurseries | Good |
| 1979 | Hezhou | Babu District | Sapindaceae | *Boniodendron minus* (Hemsl.) T. Chen | 600 | 26.0 | 159.0 | 9.5 | Wooded areas and plant nurseries | Good |
| 1980 | Hezhou | Babu District | Fagaceae | *Castanopsis faberi* Hance | 600 | 20.0 | 156.0 | 8.5 | Wooded areas and plant nurseries | Good |
| 1981 | Hezhou | Babu District | Fagaceae | *Castanopsis faberi* Hance | 600 | 16.0 | 162.0 | 6.5 | Wooded areas and plant nurseries | Good |
| 1982 | Hezhou | Babu District | Fagaceae | *Castanopsis hystrix* Hook. f. & Thomson ex A. DC. | 500 | 21.0 | 140.0 | 12.0 | Wooded areas and plant nurseries | Good |
| 1983 | Hezhou | Babu District | Fagaceae | *Castanopsis hystrix* Hook. f. & Thomson ex A. DC. | 750 | 28.0 | 179.9 | 18.0 | Wooded areas and plant nurseries | Good |
| 1984 | Hezhou | Pinggui District | Aquifoliaceae | *Ilex rotunda* Thunb. | 500 | 21.0 | 143.2 | 18.5 | Villages and farmlands | Good |
| 1985 | Hezhou | Pinggui District | Moraceae | *Ficus concinna (*Miq.) Miq. | 500 | 24.0 | 184.6 | 35.0 | Villages and farmlands | Moderate |
| 1986 | Hezhou | Pinggui District | Moraceae | *Ficus concinna (*Miq.) Miq. | 630 | 17.0 | 283.3 | 26.0 | Villages and farmlands | Good |
| 1987 | Hezhou | Pinggui District | Moraceae | *Ficus concinna (*Miq.) Miq. | 630 | 17.0 | 280.1 | 27.0 | Villages and farmlands | Good |
| 1988 | Hezhou | Pinggui District | Moraceae | *Ficus concinna (*Miq.) Miq. | 680 | 18.0 | 286.0 | 40.0 | Villages and farmlands | Good |
| 1989 | Hezhou | Pinggui District | Moraceae | *Ficus concinna (*Miq.) Miq. | 530 | 15.0 | 267.3 | 26.5 | Villages and farmlands | Good |
| 1990 | Hezhou | Pinggui District | Lauraceae | *Camphora officinarum Nees* | 610 | 15.0 | 267.4 | 15.0 | Villages and farmlands | Good |
| 1991 | Hezhou | Pinggui District | Moraceae | *Ficus concinna (*Miq.) Miq. | 530 | 16.0 | 267.3 | 29.0 | Villages and farmlands | Good |
| 1992 | Hezhou | Pinggui District | Moraceae | *Ficus concinna (*Miq.) Miq. | 600 | 17.0 | 254.6 | 30.5 | Villages and farmlands | Good |
| 1993 | Hezhou | Pinggui District | Moraceae | *Ficus concinna (*Miq.) Miq. | 680 | 17.0 | 289.6 | 33.5 | Residential districts | Good |
| 1994 | Hezhou | Pinggui District | Moraceae | *Ficus concinna (*Miq.) Miq. | 750 | 17.0 | 294.4 | 31.0 | Villages and farmlands | Good |
| 1995 | Hezhou | Pinggui District | Moraceae | *Ficus concinna (*Miq.) Miq. | 520 | 16.0 | 270.5 | 25.0 | Villages and farmlands | Good |
| 1996 | Hezhou | Pinggui District | Moraceae | *Ficus concinna (*Miq.) Miq. | 630 | 19.0 | 282.0 | 22.5 | Villages and farmlands | Good |
| 1997 | Hezhou | Pinggui District | Fagaceae | *Castanopsis hystrix* Hook. f. & Thomson ex A. DC. | 580 | 17.0 | 155.3 | 14.0 | Villages and farmlands | Good |
| 1998 | Hezhou | Pinggui District | Lauraceae | *Camphora officinarum Nees* | 600 | 35.0 | 243.8 | 30.0 | Villages and farmlands | Good |
| 1999 | Hezhou | Pinggui District | Lauraceae | *Camphora officinarum Nees* | 580 | 29.0 | 250.8 | 31.5 | Villages and farmlands | Good |
| 2000 | Hezhou | Pinggui District | Moraceae | *Ficus concinna (*Miq.) Miq. | 680 | 21.0 | 288.0 | 31.0 | Villages and farmlands | Good |
| 2001 | Hezhou | Pinggui District | Lauraceae | *Camphora officinarum Nees* | 700 | 21.0 | 281.0 | 23.2 | Villages and farmlands | Good |
| 2002 | Hezhou | Zhaoping County | Moraceae | *Ficus concinna (*Miq.) Miq. | 700 | 18.0 | 303.0 | 41.0 | Residential districts | Good |
| 2003 | Hezhou | Zhaoping County | Podocarpaceae | *Dacrycarpus imbricatus* (Blume) de Laub. | 700 | 25.0 | 130.0 | 15.0 | Villages and farmlands | Good |
| 2004 | Hezhou | Zhaoping County | Moraceae | *Ficus concinna (*Miq.) Miq. | 850 | 18.0 | 245.0 | 29.0 | Villages and farmlands | Good |
| 2005 | Hezhou | Zhaoping County | Moraceae | *Ficus concinna (*Miq.) Miq. | 800 | 17.0 | 178.0 | 26.0 | Villages and farmlands | Good |
| 2006 | Hezhou | Zhaoping County | Moraceae | *Ficus concinna (*Miq.) Miq. | 500 | 14.0 | 135.0 | 26.5 | Villages and farmlands | Good |
| 2007 | Hezhou | Zhaoping County | Lauraceae | *Camphora officinarum Nees* | 500 | 18.0 | 210.0 | 22.5 | Villages and farmlands | Good |
| 2008 | Hezhou | Zhaoping County | Lauraceae | *Camphora officinarum Nees* | 500 | 20.0 | 188.0 | 29.5 | Villages and farmlands | Good |
| 2009 | Hezhou | Zhaoping County | Lauraceae | *Camphora officinarum Nees* | 500 | 18.0 | 146.0 | 26.5 | Villages and farmlands | Good |
| 2010 | Hezhou | Zhaoping County | Lauraceae | *Camphora officinarum Nees* | 500 | 16.0 | 152.0 | 20.0 | Villages and farmlands | Good |
| 2011 | Hezhou | Zhaoping County | Lauraceae | *Camphora officinarum Nees* | 700 | 11.0 | 275.0 | 11.0 | Villages and farmlands | Good |
| 2012 | Hezhou | Zhaoping County | Lauraceae | *Camphora officinarum Nees* | 500 | 15.0 | 225.0 | 29.0 | Villages and farmlands | Good |
| 2013 | Hezhou | Zhaoping County | Fagaceae | *Castanopsis hystrix* Hook. f. & Thomson ex A. DC. | 500 | 17.0 | 140.0 | 11.0 | Villages and farmlands | Good |
| 2014 | Hezhou | Zhaoping County | Podocarpaceae | *Dacrycarpus imbricatus* (Blume) de Laub. | 700 | 21.0 | 134.0 | 15.5 | Villages and farmlands | Good |
| 2015 | Hezhou | Zhaoping County | Theaceae | *Schima superba* Gardner & Champ. | 650 | 22.0 | 132.0 | 18.5 | Villages and farmlands | Good |
| 2016 | Hezhou | Zhaoping County | Theaceae | *Schima superba* Gardner & Champ. | 500 | 25.0 | 124.0 | 19.0 | Villages and farmlands | Good |
| 2017 | Hezhou | Zhaoping County | Theaceae | *Schima superba* Gardner & Champ. | 500 | 17.0 | 124.0 | 15.0 | Villages and farmlands | Good |
| 2018 | Hezhou | Zhaoping County | Theaceae | *Schima superba* Gardner & Champ. | 700 | 20.0 | 135.0 | 20.5 | Villages and farmlands | Good |
| 2019 | Hezhou | Zhaoping County | Podocarpaceae | *Dacrycarpus imbricatus* (Blume) de Laub. | 500 | 25.0 | 108.0 | 23.5 | Villages and farmlands | Good |
| 2020 | Hezhou | Zhaoping County | Aquifoliaceae | *Ilex rotunda* Thunb. | 500 | 10.0 | 91.0 | 16.5 | Villages and farmlands | Good |
| 2021 | Hezhou | Zhaoping County | Lauraceae | *Camphora officinarum Nees* | 500 | 17.0 | 217.0 | 34.5 | Villages and farmlands | Good |
| 2022 | Hezhou | Zhaoping County | Lauraceae | *Camphora officinarum Nees* | 550 | 17.0 | 235.0 | 28.5 | Villages and farmlands | Moderate |
| 2023 | Hezhou | Zhongshan County | Lauraceae | *Camphora officinarum Nees* | 540 | 18.0 | 213.4 | 34.5 | Villages and farmlands | Good |
| 2024 | Hezhou | Zhongshan County | Moraceae | *Ficus concinna (*Miq.) Miq. | 600 | 17.0 | 283.7 | 33.0 | Villages and farmlands | Good |
| 2025 | Hezhou | Zhongshan County | Lauraceae | *Camphora officinarum Nees* | 850 | 15.0 | 170.4 | 15.0 | Villages and farmlands | Moderate |
| 2026 | Hezhou | Zhongshan County | Moraceae | *Ficus concinna (*Miq.) Miq. | 650 | 16.0 | 287.8 | 28.5 | Villages and farmlands | Good |
| 2027 | Hezhou | Zhongshan County | Moraceae | *Ficus concinna (*Miq.) Miq. | 600 | 17.0 | 283.1 | 32.0 | Villages and farmlands | Good |
| 2028 | Hezhou | Zhongshan County | Moraceae | *Ficus concinna (*Miq.) Miq. | 700 | 17.0 | 293.1 | 27.0 | Villages and farmlands | Good |
| 2029 | Hezhou | Zhongshan County | Moraceae | *Ficus concinna (*Miq.) Miq. | 650 | 21.0 | 286.6 | 34.5 | Villages and farmlands | Good |
| 2030 | Hezhou | Zhongshan County | Lauraceae | *Camphora officinarum Nees* | 550 | 17.0 | 248.4 | 22.0 | Villages and farmlands | Moderate |
| 2031 | Hezhou | Zhongshan County | Lauraceae | *Camphora officinarum Nees* | 600 | 18.0 | 255.8 | 36.5 | Villages and farmlands | Moderate |
| 2032 | Hezhou | Zhongshan County | Fagaceae | *Castanopsis sclerophylla* (Lindl.) Schottky | 500 | 12.0 | 151.0 | 17.5 | Villages and farmlands | Good |
| 2033 | Hezhou | Zhongshan County | Moraceae | *Ficus concinna (*Miq.) Miq. | 650 | 18.0 | 246.8 | 29.0 | Villages and farmlands | Good |
| 2034 | Hezhou | Zhongshan County | Lauraceae | *Camphora officinarum Nees* | 620 | 19.0 | 160.8 | 26.0 | Villages and farmlands | Good |
| 2035 | Hezhou | Zhongshan County | Lauraceae | *Camphora officinarum Nees* | 660 | 17.0 | 192.0 | 24.5 | Villages and farmlands | Good |
| 2036 | Hezhou | Zhongshan County | Lauraceae | *Camphora officinarum Nees* | 630 | 17.0 | 160.2 | 22.5 | Villages and farmlands | Good |
| 2037 | Hezhou | Zhongshan County | Moraceae | *Ficus concinna (*Miq.) Miq. | 620 | 17.0 | 145.2 | 22.5 | Villages and farmlands | Good |
| 2038 | Hezhou | Zhongshan County | Lauraceae | *Camphora officinarum Nees.* | 630 | 18.0 | 168.8 | 31.0 | Villages and farmlands | Good |
| 2039 | Hezhou | Zhongshan County | Lauraceae | *Camphora officinarum Nees.* | 625 | 19.0 | 152.9 | 27.5 | Villages and farmlands | Good |
| 2040 | Hezhou | Zhongshan County | Moraceae | *Ficus concinna (*Miq.) Miq. | 500 | 20.0 | 268.5 | 36.0 | Villages and farmlands | Good |
| 2041 | Hezhou | Zhongshan County | Moraceae | *Ficus concinna (*Miq.) Miq. | 650 | 16.0 | 289.9 | 31.0 | Villages and farmlands | Good |
| 2042 | Hezhou | Zhongshan County | Moraceae | *Ficus concinna (*Miq.) Miq. | 500 | 22.0 | 264.3 | 37.0 | Villages and farmlands | Good |
| 2043 | Hezhou | Zhongshan County | Lauraceae | *Camphora officinarum Nees.* | 550 | 18.0 | 251.6 | 33.0 | Villages and farmlands | Good |
| 2044 | Hezhou | Zhongshan County | Lauraceae | *Camphora officinarum Nees.* | 555 | 18.0 | 251.6 | 36.5 | Villages and farmlands | Good |
| 2045 | Hezhou | Zhongshan County | Lauraceae | *Camphora officinarum Nees.* | 770 | 17.0 | 296.2 | 26.5 | Villages and farmlands | Good |
| 2046 | Hezhou | Zhongshan County | Lauraceae | *Camphora officinarum Nees.* | 600 | 22.0 | 260.0 | 15.0 | Villages and farmlands | Moderate |
| 2047 | Hezhou | Zhongshan County | Moraceae | *Ficus concinna (*Miq.) Miq. | 500 | 16.0 | 146.5 | 17.5 | Villages and farmlands | Good |
| 2048 | Hezhou | Zhongshan County | Moraceae | *Ficus concinna (*Miq.) Miq. | 560 | 18.0 | 204.5 | 29.5 | Villages and farmlands | Good |
| 2049 | Hezhou | Zhongshan County | Lauraceae | *Camphora officinarum Nees* | 520 | 16.0 | 131.5 | 29.0 | Villages and farmlands | Good |
| 2050 | Hezhou | Zhongshan County | Lauraceae | *Camphora officinarum Nees* | 600 | 21.0 | 254.3 | 30.5 | Villages and farmlands | Good |
| 2051 | Hezhou | Zhongshan County | Moraceae | *Ficus concinna (*Miq.) Miq. | 700 | 18.0 | 296.7 | 43.5 | Villages and farmlands | Good |
| 2052 | Hezhou | Zhongshan County | Moraceae | *Ficus concinna (*Miq.) Miq. | 800 | 23.0 | 302.5 | 45.0 | Villages and farmlands | Good |
| 2053 | Hezhou | Zhongshan County | Lauraceae | *Camphora officinarum Nees.* | 750 | 15.0 | 293.2 | 26.0 | Villages and farmlands | Good |
| 2054 | Hezhou | Zhongshan County | Lauraceae | *Camphora officinarum Nees.* | 500 | 21.0 | 181.5 | 36.0 | Villages and farmlands | Good |
| 2055 | Hezhou | Zhongshan County | Lauraceae | *Camphora officinarum Nees.* | 500 | 23.0 | 168.8 | 25.5 | Villages and farmlands | Good |
| 2056 | Hezhou | Zhongshan County | Lauraceae | *Camphora officinarum Nees.* | 500 | 19.0 | 162.4 | 21.5 | Villages and farmlands | Good |
| 2057 | Hezhou | Zhongshan County | Oleaceae | *Osmanthus fragrans* (Thunb.) Lour. | 950 | 16.0 | 105.1 | 14.0 | Villages and farmlands | Moderate |
| 2058 | Hezhou | Zhongshan County | Taxaceae | *Taxus wallichiana* var. *mairei* (Lemée & H. Lév.) L. K. Fu & Nan Li | 600 | 14.0 | 120.1 | 7.0 | Villages and farmlands | Moderate |
| 2059 | Hezhou | Zhongshan County | Lauraceae | *Camphora officinarum Nees.* | 500 | 23.0 | 222.9 | 33.5 | Villages and farmlands | Good |
| 2060 | Hezhou | Zhongshan County | Lauraceae | *Camphora officinarum Nees.* | 600 | 25.0 | 245.2 | 30.5 | Villages and farmlands | Good |
| 2061 | Hezhou | Zhongshan County | Fagaceae | *Castanopsis chinensis* (Spreng.) Hance | 500 | 20.0 | 149.0 | 17.5 | Villages and farmlands | Good |
| 2062 | Hezhou | Fuchuan County | Lauraceae | *Camphora officinarum Nees.* | 550 | 18.0 | 238.9 | 16.5 | Villages and farmlands | Good |
| 2063 | Hezhou | Fuchuan County | Lauraceae | *Camphora officinarum Nees.* | 500 | 15.0 | 179.9 | 15.5 | Villages and farmlands | Good |
| 2064 | Hezhou | Fuchuan County | Anacardiaceae | *Pistacia chinensis* Bunge. | 500 | 10.0 | 105.1 | 10.0 | Residential districts | Moderate |
| 2065 | Hezhou | Fuchuan County | Lauraceae | *Camphora officinarum Nees.* | 500 | 22.0 | 202.5 | 27.0 | Villages and farmlands | Good |
| 2066 | Hezhou | Fuchuan County | Phyllanthaceae | *Bischofia polycarpa* (H. Lév.) Airy Shaw | 600 | 10.0 | 241.4 | 15.0 | Villages and farmlands | Good |
| 2067 | Hezhou | Fuchuan County | Aquifoliaceae | *Ilex rotunda* Thunb. | 500 | 10.0 | 103.8 | 16.0 | Villages and farmlands | Good |
| 2068 | Hezhou | Fuchuan County | Lauraceae | *Camphora officinarum Nees.* | 500 | 16.0 | 187.3 | 17.0 | Villages and farmlands | Good |
| 2069 | Hezhou | Fuchuan County | Phyllanthaceae | *Bischofia polycarpa* (H. Lév.) Airy Shaw | 500 | 20.0 | 181.5 | 18.0 | Villages and farmlands | Moderate |
| 2070 | Hezhou | Fuchuan County | Lauraceae | *Camphora officinarum Nees.* | 500 | 15.0 | 200.0 | 14.5 | Villages and farmlands | Moderate |
| 2071 | Hezhou | Fuchuan County | Lauraceae | *Camphora officinarum Nees.* | 500 | 9.0 | 220.0 | 6.5 | Villages and farmlands | Poor |
| 2072 | Hezhou | Fuchuan County | Lauraceae | *Camphora officinarum Nees.* | 500 | 23.0 | 182.2 | 20.0 | Villages and farmlands | Good |
| 2073 | Hezhou | Fuchuan County | Fagaceae | *Castanopsis hystrix* Hook. f. & Thomson ex A. DC. | 720 | 16.0 | 175.2 | 17.0 | Villages and farmlands | Good |
| 2074 | Hezhou | Fuchuan County | Aquifoliaceae | *Ilex rotunda* Thunb. | 600 | 14.0 | 105.1 | 12.0 | Villages and farmlands | Good |
| 2075 | Hezhou | Fuchuan County | Lauraceae | *Camphora officinarum Nees.* | 500 | 18.0 | 219.7 | 18.0 | Villages and farmlands | Good |
| 2076 | Hezhou | Fuchuan County | Anacardiaceae | *Pistacia chinensis* Bunge. | 500 | 20.0 | 156.1 | 12.5 | Villages and farmlands | Moderate |
| 2077 | Hezhou | Fuchuan County | Anacardiaceae | *Pistacia chinensis* Bunge. | 500 | 20.0 | 107.0 | 15.5 | Villages and farmlands | Moderate |
| 2078 | Hezhou | Fuchuan County | Fagaceae | *Castanopsis sclerophylla* (Lindl.) Schottky | 500 | 10.0 | 115.0 | 9.0 | Villages and farmlands | Moderate |
| 2079 | Hezhou | Fuchuan County | Fagaceae | *Castanopsis sclerophylla* (Lindl.) Schottky | 500 | 13.0 | 114.6 | 10.5 | Villages and farmlands | Moderate |
| 2080 | Hezhou | Fuchuan County | Fagaceae | *Quercus fabri* Hance. | 700 | 20.0 | 121.0 | 17.0 | Villages and farmlands | Good |
| 2081 | Hezhou | Fuchuan County | Fabaceae | *Styphnolobium japonicum* (L.) Schott | 650 | 21.0 | 162.4 | 12.0 | Villages and farmlands | Moderate |
| 2082 | Hezhou | Fuchuan County | Fabaceae | *Styphnolobium japonicum* (L.) Schott | 500 | 18.0 | 137.9 | 13.5 | Villages and farmlands | Good |
| 2083 | Hezhou | Fuchuan County | Pinaceae | *Keteleeria davidiana* var. *calcarea* (C. Y. Cheng & L. K. Fu) Silba | 520 | 24.0 | 149.0 | 12.0 | Villages and farmlands | Good |
| 2084 | Hezhou | Fuchuan County | Fabaceae | *Gleditsia sinensis* Lam. | 500 | 22.0 | 140.0 | 19.0 | Villages and farmlands | Good |
| 2085 | Hezhou | Fuchuan County | Fabaceae | *Styphnolobium japonicum* (L.) Schott | 500 | 18.0 | 130.0 | 17.0 | Villages and farmlands | Good |
| 2086 | Hezhou | Fuchuan County | Fabaceae | *Gleditsia sinensis* Lam. | 650 | 17.0 | 157.6 | 14.0 | Villages and farmlands | Good |
| 2087 | Hezhou | Fuchuan County | Fagaceae | *Castanopsis sclerophylla* (Lindl.) Schottky | 500 | 15.0 | 137.6 | 13.0 | Villages and farmlands | Good |
| 2088 | Hezhou | Fuchuan County | Fagaceae | *Castanopsis sclerophylla* (Lindl.) Schottky | 800 | 14.0 | 204.5 | 12.0 | Villages and farmlands | Good |
| 2089 | Hezhou | Fuchuan County | Fagaceae | *Castanopsis sclerophylla* (Lindl.) Schottky | 600 | 16.0 | 162.4 | 8.0 | Villages and farmlands | Good |
| 2090 | Hezhou | Fuchuan County | Fagaceae | *Castanopsis sclerophylla* (Lindl.) Schottky | 500 | 16.0 | 122.6 | 5.0 | Villages and farmlands | Good |
| 2091 | Hezhou | Fuchuan County | Fagaceae | *Castanopsis sclerophylla* (Lindl.) Schottky | 500 | 15.0 | 137.3 | 13.0 | Villages and farmlands | Good |
| 2092 | Hezhou | Fuchuan County | Fagaceae | *Castanopsis sclerophylla* (Lindl.) Schottky | 500 | 10.0 | 138.5 | 12.0 | Villages and farmlands | Good |
| 2093 | Hezhou | Fuchuan County | Fagaceae | *Castanopsis sclerophylla* (Lindl.) Schottky | 520 | 13.0 | 143.6 | 11.0 | Villages and farmlands | Good |
| 2094 | Hezhou | Fuchuan County | Fagaceae | *Castanopsis sclerophylla* (Lindl.) Schottky | 500 | 16.0 | 127.4 | 11.0 | Villages and farmlands | Good |
| 2095 | Hezhou | Fuchuan County | Fagaceae | *Quercus acutissima* Carruth. | 550 | 16.0 | 114.6 | 14.0 | Villages and farmlands | Good |
| 2096 | Hezhou | Fuchuan County | Ulmaceae | *Zelkova schneideriana* Hand.-Mazz. | 550 | 7.0 | 183.1 | 10.0 | Villages and farmlands | Moderate |
| 2097 | Hezhou | Fuchuan County | Aquifoliaceae | *Ilex rotunda* Thunb. | 700 | 10.0 | 121.0 | 8.0 | Villages and farmlands | Good |
| 2098 | Hezhou | Fuchuan County | Lauraceae | *Camphora officinarum Nees* | 500 | 17.0 | 198.4 | 15.0 | Villages and farmlands | Good |
| 2099 | Hezhou | Fuchuan County | Fagaceae | *Castanopsis scle*r*ophylla* (Lindl.) Schottky | 500 | 19.0 | 130.6 | 16.0 | Villages and farmlands | Good |
| 2100 | Hezhou | Fuchuan County | Sapindaceae | *Acer coriaceifolium* H. Lév. | 600 | 21.0 | 97.1 | 11.0 | Villages and farmlands | Good |
| 2101 | Hezhou | Fuchuan County | Fagaceae | *Castanopsis sclerophylla* (Lindl.) Schottky | 500 | 19.0 | 122.9 | 14.0 | Villages and farmlands | Good |
| 2102 | Hezhou | Fuchuan County | Fagaceae | *Castanopsis sclerophylla* (Lindl.) Schottky | 500 | 13.0 | 137.6 | 8.5 | Villages and farmlands | Good |
| 2103 | Hezhou | Fuchuan County | Fagaceae | *Castanopsis sclerophylla* (Lindl.) Schottky | 500 | 17.0 | 117.8 | 16.0 | Villages and farmlands | Good |
| 2104 | Hezhou | Fuchuan County | Lauraceae | *Camphora officinarum Nees* | 500 | 14.0 | 141.4 | 14.0 | Villages and farmlands | Good |
| 2105 | Hezhou | Fuchuan County | Theaceae | *Schima superba* Gardner & Champ. | 500 | 20.0 | 115.9 | 18.0 | Villages and farmlands | Good |
| 2106 | Hezhou | Fuchuan County | Fagaceae | *Castanopsis sclerophylla* (Lindl.) Schottky | 500 | 13.0 | 117.8 | 11.0 | Villages and farmlands | Good |
| 2107 | Hezhou | Fuchuan County | Cupressaceae | *Juniperus chinensis* Roxb. | 550 | 14.0 | 116.2 | 9.0 | Villages and farmlands | Moderate |
| 2108 | Hezhou | Fuchuan County | Elaeocarpaceae | *Elaeocarpus sylvestris* (Lour.) Poir. | 500 | 16.0 | 85.0 | 16.0 | Villages and farmlands | Good |
| 2109 | Hezhou | Fuchuan County | Fagaceae | *Castanopsis sclerophylla* (Lindl.) Schottky | 500 | 20.0 | 128.0 | 17.0 | Villages and farmlands | Good |
| 2110 | Hezhou | Fuchuan County | Lauraceae | *Camphora officinarum Nees* | 550 | 20.0 | 227.1 | 26.5 | Villages and farmlands | Good |
| 2111 | Hezhou | Fuchuan County | Lauraceae | *Camphora officinarum Nees* | 550 | 21.0 | 222.3 | 16.5 | Villages and farmlands | Good |
| 2112 | Hezhou | Fuchuan County | Lauraceae | *Camphora officinarum Nees* | 700 | 13.0 | 276.4 | 19.5 | Villages and farmlands | Good |
| 2113 | Hezhou | Fuchuan County | Lauraceae | *Phoebe bournei (*Hemsl.) Yen C. Yang | 500 | 26.0 | 116.0 | 21.0 | Villages and farmlands | Good |
| 2114 | Hezhou | Fuchuan County | Fagaceae | *Castanopsis sclerophylla* (Lindl.) Schottky | 600 | 25.0 | 165.0 | 14.0 | Villages and farmlands | Good |
| 2115 | Hezhou | Fuchuan County | Fagaceae | *Castanopsis sclerophylla* (Lindl.) Schottky | 600 | 17.0 | 164.0 | 15.0 | Villages and farmlands | Good |
| 2116 | Hezhou | Fuchuan County | Fagaceae | *Castanopsis sclerophylla* (Lindl.) Schottky | 500 | 22.0 | 116.2 | 16.0 | Villages and farmlands | Good |
| 2117 | Hezhou | Fuchuan County | Phyllanthaceae | *Bischofia polycarpa* (H. Lév.) Airy Shaw | 500 | 20.0 | 216.9 | 16.0 | Villages and farmlands | Good |
| 2118 | Hezhou | Fuchuan County | Fagaceae | *Castanopsis sclerophylla* (Lindl.) Schottky | 500 | 14.0 | 131.2 | 8.5 | Villages and farmlands | Good |
| 2119 | Hezhou | Fuchuan County | Fagaceae | *Castanopsis sclerophylla* (Lindl.) Schottky | 500 | 17.0 | 114.6 | 11.0 | Villages and farmlands | Good |
| 2120 | Hezhou | Fuchuan County | Fagaceae | *Castanopsis sclerophylla* (Lindl.) Schottky | 500 | 17.0 | 122.6 | 13.5 | Villages and farmlands | Good |
| 2121 | Hezhou | Fuchuan County | Fagaceae | *Castanopsis sclerophylla* (Lindl.) Schottky | 600 | 17.0 | 168.8 | 14.0 | Villages and farmlands | Good |
| 2122 | Hezhou | Fuchuan County | Lauraceae | *Camphora officinarum Nees* | 500 | 17.0 | 184.1 | 16.0 | Villages and farmlands | Good |
| 2123 | Hezhou | Fuchuan County | Fagaceae | *Castanopsis sclerophylla* (Lindl.) Schottky | 500 | 16.0 | 122.0 | 12.5 | Villages and farmlands | Good |
| 2124 | Hezhou | Fuchuan County | Fagaceae | *Castanopsis sclerophylla* (Lindl.) Schottky | 500 | 16.0 | 108.0 | 13.0 | Villages and farmlands | Good |
| 2125 | Hezhou | Fuchuan County | Fagaceae | *Castanopsis sclerophylla* (Lindl.) Schottky | 500 | 17.0 | 137.0 | 12.5 | Villages and farmlands | Good |
| 2126 | Hezhou | Fuchuan County | Fagaceae | *Castanopsis sclerophylla* (Lindl.) Schottky | 500 | 20.0 | 114.6 | 14.0 | Villages and farmlands | Good |
| 2127 | Hezhou | Fuchuan County | Fagaceae | *Castanopsis sclerophylla* (Lindl.) Schottky | 500 | 19.0 | 115.6 | 14.0 | Villages and farmlands | Good |
| 2128 | Hezhou | Fuchuan County | Fagaceae | *Castanopsis sclerophylla* (Lindl.) Schottky | 600 | 21.0 | 168.8 | 14.0 | Villages and farmlands | Good |
| 2129 | Hezhou | Fuchuan County | Fagaceae | *Castanopsis sclerophylla* (Lindl.) Schottky | 500 | 16.0 | 145.0 | 12.5 | Villages and farmlands | Good |
| 2130 | Hezhou | Fuchuan County | Fagaceae | *Castanopsis sclerophylla* (Lindl.) Schottky | 600 | 13.0 | 162.4 | 12.0 | Villages and farmlands | Good |
| 2131 | Hezhou | Fuchuan County | Fagaceae | *Castanopsis sclerophylla* (Lindl.) Schottky | 500 | 21.0 | 143.3 | 15.5 | Villages and farmlands | Good |
| 2132 | Hezhou | Fuchuan County | Theaceae | *Schima superba* Gardner & Champ. | 500 | 20.0 | 108.3 | 13.0 | Villages and farmlands | Good |
| 2133 | Hezhou | Fuchuan County | Fagaceae | *Castanopsis sclerophylla* (Lindl.) Schottky | 500 | 18.0 | 128.3 | 11.0 | Villages and farmlands | Good |
| 2134 | Hezhou | Fuchuan County | Fagaceae | *Castanopsis sclerophylla* (Lindl.) Schottky | 600 | 9.0 | 163.1 | 8.5 | Villages and farmlands | Good |
| 2135 | Hezhou | Fuchuan County | Fagaceae | *Castanopsis sclerophylla* (Lindl.) Schottky | 500 | 18.0 | 142.4 | 13.0 | Villages and farmlands | Good |
| 2136 | Hezhou | Fuchuan County | Fagaceae | *Castanopsis sclerophylla* (Lindl.) Schottky | 500 | 16.0 | 129.9 | 10.5 | Villages and farmlands | Good |
| 2137 | Hezhou | Fuchuan County | Fagaceae | *Castanopsis sclerophylla* (Lindl.) Schottky | 700 | 16.0 | 190.4 | 13.0 | Villages and farmlands | Good |
| 2138 | Hezhou | Fuchuan County | Fagaceae | *Castanopsis sclerophylla* (Lindl.) Schottky | 550 | 16.0 | 148.1 | 15.0 | Villages and farmlands | Good |
| 2139 | Hezhou | Fuchuan County | Phyllanthaceae | *Bischofia polycarpa* (H. Lév.) Airy Shaw | 500 | 18.0 | 200.6 | 15.0 | Villages and farmlands | Moderate |
| 2140 | Hezhou | Fuchuan County | Sapindaceae | *Acer coriaceifolium* H. Lév. | 700 | 18.0 | 99.4 | 12.0 | Villages and farmlands | Good |
| 2141 | Hezhou | Fuchuan County | Cannabaceae | *Celtis sinensis* Pers. | 500 | 20.0 | 148.4 | 11.5 | Villages and farmlands | Good |
| 2142 | Hezhou | Fuchuan County | Sapindaceae | *Acer coriaceifolium* H. Lév. | 650 | 20.0 | 98.1 | 12.5 | Villages and farmlands | Good |
| 2143 | Hezhou | Fuchuan County | Fagaceae | *Castanopsis sclerophylla* (Lindl.) Schottky | 500 | 20.0 | 122.4 | 13.0 | Villages and farmlands | Good |
| 2144 | Hezhou | Fuchuan County | Fagaceae | *Castanopsis sclerophylla* (Lindl.) Schottky | 500 | 17.0 | 118.8 | 12.0 | Villages and farmlands | Good |
| 2145 | Hezhou | Fuchuan County | Fagaceae | *Castanopsis sclerophylla* (Lindl.) Schottky | 600 | 20.0 | 162.4 | 17.0 | Villages and farmlands | Good |
| 2146 | Hezhou | Fuchuan County | Fagaceae | *Castanopsis sclerophylla* (Lindl.) Schottky | 500 | 26.0 | 144.3 | 16.0 | Villages and farmlands | Good |
| 2147 | Hezhou | Fuchuan County | Sapindaceae | *Acer coriaceifolium* H. Lév. | 600 | 16.0 | 109.2 | 13.0 | Villages and farmlands | Good |
| 2148 | Hezhou | Fuchuan County | Fagaceae | *Castanopsis sclerophylla* (Lindl.) Schottky | 500 | 22.0 | 142.0 | 9.0 | Villages and farmlands | Good |
| 2149 | Hezhou | Fuchuan County | Ulmaceae | *Zelkova schneideriana* Hand.-Mazz. | 600 | 15.0 | 185.0 | 11.5 | Villages and farmlands | Good |
| 2150 | Hezhou | Fuchuan County | Aquifoliaceae | *Ilex rotunda* Thunb. | 600 | 18.0 | 113.4 | 17.5 | Villages and farmlands | Good |
| 2151 | Hezhou | Fuchuan County | Fagaceae | *Castanopsis sclerophylla* (Lindl.) Schottky | 800 | 16.0 | 200.0 | 15.0 | Villages and farmlands | Good |
| 2152 | Hezhou | Fuchuan County | Fagaceae | *Castanopsis sclerophylla* (Lindl.) Schottky | 800 | 17.0 | 203.8 | 12.5 | Villages and farmlands | Good |
| 2153 | Hezhou | Fuchuan County | Fabaceae | *Styphnolobium japonicum* (L.) Schott | 500 | 6.0 | 137.6 | 5.5 | Villages and farmlands | Good |
| 2154 | Hezhou | Fuchuan County | Fagaceae | *Castanopsis hystrix* Hook. f. & Thomson ex A. DC. | 550 | 10.0 | 147.1 | 7.0 | Villages and farmlands | Good |
| 2155 | Hezhou | Fuchuan County | Fagaceae | *Castanopsis sclerophylla* (Lindl.) Schottky | 500 | 13.0 | 122.0 | 14.0 | Villages and farmlands | Good |
| 2156 | Hezhou | Fuchuan County | Fagaceae | *Castanopsis sclerophylla* (Lindl.) Schottky | 500 | 11.0 | 120.0 | 8.0 | Villages and farmlands | Good |
| 2157 | Hezhou | Fuchuan County | Anacardiaceae | *Pistacia chinensis* Bunge | 500 | 25.0 | 124.2 | 13.0 | Villages and farmlands | Moderate |
| 2158 | Hezhou | Fuchuan County | Phyllanthaceae | *Bischofia polycarpa* (H. Lév.) Airy Shaw | 500 | 18.0 | 225.5 | 20.0 | Residential districts | Good |
| 2159 | Hezhou | Fuchuan County | Lauraceae | *Camphora officinarum Nees* | 750 | 21.0 | 285.0 | 22.5 | Villages and farmlands | Good |
| 2160 | Hezhou | Fuchuan County | Theaceae | *Schima superba* Gardner & Champ. | 500 | 26.0 | 116.9 | 12.5 | Villages and farmlands | Good |
| 2161 | Hezhou | Fuchuan County | Fagaceae | *Castanopsis scle*r*ophylla* (Lindl.) Schottky | 750 | 11.0 | 193.6 | 7.5 | Villages and farmlands | Good |
| 2162 | Hezhou | Fuchuan County | Fagaceae | *Castanopsis scle*r*ophylla* (Lindl.) Schottky | 650 | 16.0 | 175.0 | 13.0 | Villages and farmlands | Good |
| 2163 | Hezhou | Fuchuan County | Fagaceae | *Castanopsis scle*r*ophylla* (Lindl.) Schottky | 550 | 16.0 | 149.7 | 7.5 | Villages and farmlands | Good |
| 2164 | Hezhou | Fuchuan County | Fabaceae | *Gleditsia sinensis* Lam. | 500 | 18.0 | 141.7 | 15.0 | Villages and farmlands | Good |
| 2165 | Hezhou | Fuchuan County | Phyllanthaceae | *Bischofia polycarpa* (H. Lév.) Airy Shaw | 500 | 14.0 | 220.4 | 9.0 | Villages and farmlands | Moderate |
| 2166 | Hezhou | Fuchuan County | Fagaceae | *Castanopsis sclerophylla* (Lindl.) Schottky | 500 | 8.0 | 101.9 | 11.0 | Villages and farmlands | Moderate |
| 2167 | Hezhou | Fuchuan County | Fagaceae | *Castanopsis sclerophylla* (Lindl.) Schottky | 500 | 21.0 | 135.7 | 18.0 | Villages and farmlands | Moderate |
| 2168 | Hezhou | Fuchuan County | Fagaceae | *Castanopsis sclerophylla* (Lindl.) Schottky | 550 | 14.0 | 153.5 | 12.0 | Villages and farmlands | Moderate |
| 2169 | Hezhou | Fuchuan County | Fagaceae | *Castanopsis sclerophylla* (Lindl.) Schottky | 500 | 24.0 | 138.5 | 14.0 | Villages and farmlands | Moderate |
| 2170 | Hezhou | Fuchuan County | Fagaceae | *Castanopsis sclerophylla* (Lindl.) Schottky | 650 | 15.0 | 173.6 | 14.0 | Villages and farmlands | Moderate |
| 2171 | Hezhou | Fuchuan County | Aquifoliaceae | *Ilex chinensis* Sims | 600 | 14.0 | 108.0 | 16.0 | Villages and farmlands | Good |
| 2172 | Hezhou | Fuchuan County | Aquifoliaceae | *Ilex rotunda* Thunb. | 600 | 10.0 | 111.5 | 20.0 | Villages and farmlands | Good |
| 2173 | Hechi | Jinchengjiang District | Moraceae | *Ficus virens* Aiton. | 1000 | 32.0 | 315.2 | 37.5 | Villages and farmlands | Good |
| 2174 | Hechi | Jinchengjiang District | Moraceae | *Ficus virens* Aiton. | 1000 | 27.0 | 382.1 | 27.5 | Villages and farmlands | Good |
| 2175 | Hechi | Jinchengjiang District | Moraceae | *Ficus virens* Aiton. | 1000 | 31.0 | 366.2 | 36.0 | Residential districts | Good |
| 2176 | Hechi | Jinchengjiang District | Moraceae | *Ficus virens* Aiton. | 1000 | 30.0 | 369.4 | 29.0 | Residential districts | Good |
| 2177 | Hechi | Jinchengjiang District | Moraceae | *Ficus virens* Aiton. | 1000 | 24.0 | 414.3 | 26.0 | Residential districts | Good |
| 2178 | Hechi | Jinchengjiang District | Moraceae | *Ficus virens* Aiton. | 1000 | 32.0 | 359.8 | 45.0 | Villages and farmlands | Good |
| 2179 | Hechi | Nandan County | Anacardiaceae | *Pistacia chinensis* Bunge | 1000 | 25.0 | 181.0 | 20.0 | Villages and farmlands | Good |
| 2180 | Hechi | Nandan County | Fabaceae | *Styphnolobium japonicum* (L.) Schott. | 1000 | 23.0 | 181.0 | 16.0 | Villages and farmlands | Good |
| 2181 | Hechi | Nandan County | Moraceae | *Ficus virens* Aiton. | 1000 | 32.0 | 394.0 | 26.0 | Villages and farmlands | Good |
| 2182 | Hechi | Tian'e County | Moraceae | *Ficus virens* Aiton. | 1000 | 32.0 | 480.0 | 29.0 | Villages and farmlands | Good |
| 2183 | Hechi | Tian'e County | Moraceae | *Ficus virens* Aiton. | 1000 | 28.0 | 414.0 | 30.5 | Villages and farmlands | Good |
| 2184 | Hechi | Tian'e County | Moraceae | *Ficus virens* Aiton. | 1000 | 27.0 | 318.5 | 24.0 | Villages and farmlands | Good |
| 2185 | Hechi | Tian'e County | Pinaceae | *Keteleeria fortunei* (A. Murray bis) Carrière | 1020 | 27.6 | 197.0 | 22.9 | Villages and farmlands | Good |
| 2186 | Hechi | Fengshan County | Magnoliaceae | *Manglietia fordiana* Oliv. | 1000 | 40.0 | 285.0 | 16.5 | Villages and farmlands | Good |
| 2187 | Hechi | Fengshan County | Fagaceae | *Castanea seguinii* Dode. | 1000 | 26.0 | 121.0 | 16.5 | Villages and farmlands | Good |
| 2188 | Hechi | Donglan County | Moraceae | *Ficus virens* Aiton. | 1200 | 20.0 | 456.0 | 35.5 | Residential districts | Good |
| 2189 | Hechi | Luocheng County | Moraceae | *Ficus microcarpa* L. f. | 1000 | 18.0 | 302.0 | 29.0 | Villages and farmlands | Good |
| 2190 | Hechi | Luocheng County | Moraceae | *Ficus microcarpa* L. f. | 1000 | 18.0 | 452.0 | 37.5 | Villages and farmlands | Good |
| 2191 | Hechi | Luocheng County | Moraceae | *Ficus microcarpa* L. f. | 1000 | 17.0 | 334.0 | 33.0 | Villages and farmlands | Good |
| 2192 | Hechi | Luocheng County | Moraceae | *Ficus microcarpa* L. f. | 1000 | 20.0 | 356.0 | 41.5 | Villages and farmlands | Good |
| 2193 | Hechi | Luocheng County | Moraceae | *Ficus microcarpa* L. f. | 1000 | 19.0 | 363.0 | 38.0 | Villages and farmlands | Good |
| 2194 | Hechi | Luocheng County | Moraceae | *Ficus virens* Aiton. | 1000 | 18.1 | 306.0 | 21.5 | Villages and farmlands | Good |
| 2195 | Hechi | Luocheng County | Moraceae | *Ficus microcarpa* L. f. | 1000 | 16.0 | 407.0 | 27.0 | Villages and farmlands | Good |
| 2196 | Hechi | Luocheng County | Moraceae | *Ficus microcarpa* L. f. | 1200 | 20.0 | 385.0 | 43.0 | Villages and farmlands | Good |
| 2197 | Hechi | Luocheng County | Moraceae | *Ficus microcarpa* L. f. | 1000 | 22.0 | 309.0 | 36.0 | Villages and farmlands | Good |
| 2198 | Hechi | Luocheng County | Moraceae | *Ficus virens* Aiton. | 1200 | 20.0 | 464.0 | 30.0 | Villages and farmlands | Good |
| 2199 | Hechi | Huanjiang County | Moraceae | *Ficus concinna* (Miq.) Miq. | 1000 | 30.2 | 346.6 | 40.8 | Villages and farmlands | Good |
| 2200 | Hechi | Huanjiang County | Moraceae | *Ficus virens* Aiton. | 1000 | 15.0 | 312.0 | 28.4 | Villages and farmlands | Good |
| 2201 | Hechi | Huanjiang County | Moraceae | *Ficus concinna* (Miq.) Miq. | 1000 | 32.1 | 302.0 | 40.6 | Villages and farmlands | Good |
| 2202 | Hechi | Huanjiang County | Moraceae | *Ficus virens* Aiton. | 1000 | 18.5 | 350.0 | 46.3 | Residential districts | Good |
| 2203 | Hechi | Bama County | Juglandaceae | *Carya sinensis* Dode. | 2000 | 30.3 | 210.0 | 24.4 | Villages and farmlands | Good |
| 2204 | Hechi | Bama County | Dipterocarpaceae | *Parashorea chinensis* H. Wang. | 1000 | 58.8 | 138.0 | 12.6 | Villages and farmlands | Good |
| 2205 | Hechi | Du'an County | Meliaceae | *Toona ciliata* M. Roem. | 1191 | 26.0 | 200.6 | 30.5 | Villages and farmlands | Good |
| 2206 | Hechi | Du'an County | Magnoliaceae | *Manglietia aromatica* Dandy. | 1000 | 19.5 | 185.3 | 16.3 | Villages and farmlands | Moderate |
| 2207 | Hechi | Yizhou District | Moraceae | *Ficus virens* Aiton. | 1100 | 17.0 | 430.0 | 19.4 | Residential districts | Good |
| 2208 | Hechi | Yizhou District | Moraceae | *Ficus virens* Aiton. | 1000 | 28.0 | 433.0 | 44.5 | Villages and farmlands | Good |
| 2209 | Hechi | Yizhou District | Juglandaceae | *Carya sinensis* Dode. | 1000 | 21.0 | 265.0 | 24.0 | Villages and farmlands | Good |
| 2210 | Hechi | Jinchengjiang District | Moraceae | *Ficus virens* Aiton. | 750 | 32.0 | 308.9 | 35.0 | Villages and farmlands | Good |
| 2211 | Hechi | Jinchengjiang District | Moraceae | *Ficus virens* Aiton. | 600 | 37.0 | 308.9 | 45.0 | Villages and farmlands | Moderate |
| 2212 | Hechi | Jinchengjiang District | Moraceae | *Ficus virens* Aiton. | 512 | 7.0 | 229.3 | 5.5 | Residential districts | Good |
| 2213 | Hechi | Jinchengjiang District | Moraceae | *Ficus virens* Aiton. | 500 | 25.0 | 274.0 | 27.0 | Residential districts | Good |
| 2214 | Hechi | Jinchengjiang District | Moraceae | *Ficus virens* Aiton. | 500 | 25.0 | 264.3 | 28.0 | Residential districts | Good |
| 2215 | Hechi | Jinchengjiang District | Moraceae | *Ficus virens* Aiton. | 500 | 28.0 | 261.5 | 34.0 | Residential districts | Good |
| 2216 | Hechi | Jinchengjiang District | Moraceae | *Ficus virens* Aiton. | 600 | 20.0 | 302.0 | 22.0 | Wooded areas and plant nurseries | Good |
| 2217 | Hechi | Jinchengjiang District | Moraceae | *Ficus microcarpa* L. f. | 550 | 31.0 | 270.7 | 29.0 | Villages and farmlands | Good |
| 2218 | Hechi | Jinchengjiang District | Moraceae | *Ficus virens* Aiton. | 500 | 29.0 | 251.5 | 29.0 | Villages and farmlands | Good |
| 2219 | Hechi | Jinchengjiang District | Moraceae | *Ficus microcarpa* L. f. | 550 | 25.0 | 280.3 | 24.0 | Villages and farmlands | Good |
| 2220 | Hechi | Jinchengjiang District | Moraceae | *Ficus microcarpa* L. f. | 550 | 18.0 | 280.3 | 19.0 | Villages and farmlands | Good |
| 2221 | Hechi | Jinchengjiang District | Moraceae | *Ficus microcarpa* L. f. | 500 | 16.0 | 318.5 | 18.0 | Villages and farmlands | Good |
| 2222 | Hechi | Jinchengjiang District | Moraceae | *Ficus virens* Aiton. | 800 | 28.0 | 353.5 | 37.5 | Residential districts | Good |
| 2223 | Hechi | Jinchengjiang District | Moraceae | *Ficus virens* Aiton. | 500 | 30.0 | 256.3 | 39.0 | Villages and farmlands | Good |
| 2224 | Hechi | Jinchengjiang District | Moraceae | *Ficus virens* Aiton. | 550 | 18.0 | 296.2 | 30.0 | Villages and farmlands | Good |
| 2225 | Hechi | Jinchengjiang District | Anacardiaceae | *Pistacia chinensis* Bunge | 550 | 33.0 | 197.4 | 24.0 | Villages and farmlands | Good |
| 2226 | Hechi | Jinchengjiang District | Moraceae | *Ficus microcarpa* L. f. | 650 | 31.0 | 286.6 | 27.0 | Villages and farmlands | Good |
| 2227 | Hechi | Jinchengjiang District | Moraceae | *Ficus microcarpa* L. f. | 550 | 30.0 | 277.1 | 22.5 | Villages and farmlands | Good |
| 2228 | Hechi | Jinchengjiang District | Moraceae | *Ficus virens* Aiton. | 700 | 21.0 | 363.0 | 26.0 | Villages and farmlands | Good |
| 2229 | Hechi | Jinchengjiang District | Moraceae | *Ficus virens* Aiton. | 600 | 32.0 | 305.6 | 30.0 | Villages and farmlands | Moderate |
| 2230 | Hechi | Jinchengjiang District | Moraceae | *Ficus virens* Aiton. | 570 | 26.0 | 299.3 | 23.0 | Villages and farmlands | Good |
| 2231 | Hechi | Jinchengjiang District | Moraceae | *Ficus virens* Aiton. | 570 | 31.0 | 299.3 | 30.0 | Villages and farmlands | Good |
| 2232 | Hechi | Jinchengjiang District | Moraceae | *Ficus virens* Aiton. | 500 | 25.0 | 261.1 | 12.0 | Villages and farmlands | Moderate |
| 2233 | Hechi | Jinchengjiang District | Moraceae | *Ficus virens* Aiton. | 500 | 32.0 | 270.7 | 31.5 | Villages and farmlands | Good |
| 2234 | Hechi | Jinchengjiang District | Moraceae | *Ficus virens* Aiton. | 600 | 16.0 | 318.5 | 15.5 | Villages and farmlands | Good |
| 2235 | Hechi | Jinchengjiang District | Altingiaceae | *Liquidambar formosana* Hance | 700 | 37.0 | 240.7 | 20.0 | Villages and farmlands | Moderate |
| 2236 | Hechi | Jinchengjiang District | Altingiaceae | *Liquidambar formosana* Hance | 500 | 25.0 | 197.5 | 32.5 | Villages and farmlands | Good |
| 2237 | Hechi | Nandan County | Aquifoliaceae | *Ilex pentagona S*. K. Chen | 500 | 10.0 | 117.8 | 4.0 | Residential districts | Moderate |
| 2238 | Hechi | Nandan County | Juglandaceae | *Carya cathayensis* Sarg. | 500 | 18.0 | 174.0 | 30.5 | Villages and farmlands | Moderate |
| 2239 | Hechi | Nandan County | Juglandaceae | *Carya cathayensis* Sarg. | 500 | 18.0 | 132.0 | 22.0 | Villages and farmlands | Moderate |
| 2240 | Hechi | Nandan County | Juglandaceae | *Carya cathayensis* Sarg. | 500 | 12.0 | 94.0 | 8.0 | Villages and farmlands | Moderate |
| 2241 | Hechi | Nandan County | Juglandaceae | *Carya cathayensis* Sarg. | 500 | 20.0 | 94.0 | 21.0 | Villages and farmlands | Moderate |
| 2242 | Hechi | Nandan County | Moraceae | *Ficus virens* Aiton. | 500 | 18.0 | 120.0 | 28.0 | Villages and farmlands | Good |
| 2243 | Hechi | Nandan County | Rosaceae | *Photinia bodinieri* H. Lév. | 500 | 18.0 | 210.0 | 7.0 | Villages and farmlands | Good |
| 2244 | Hechi | Nandan County | Rosaceae | *Photinia bodinieri* H. Lév. | 500 | 19.0 | 180.0 | 9.0 | Villages and farmlands | Good |
| 2245 | Hechi | Nandan County | Myricaceae | *Morella rubra* Lour. | 500 | 15.0 | 190.0 | 8.0 | Villages and farmlands | Good |
| 2246 | Hechi | Nandan County | Anacardiaceae | *Pistacia chinensis* Bunge | 600 | 27.0 | 150.0 | 20.0 | Villages and farmlands | Good |
| 2247 | Hechi | Nandan County | Anacardiaceae | *Pistacia chinensis* Bunge | 700 | 34.0 | 230.0 | 18.0 | Villages and farmlands | Good |
| 2248 | Hechi | Nandan County | Pinaceae | *Keteleeria pubescens* W. C. Cheng & L. K. Fu | 500 | 25.0 | 168.0 | 30.0 | Villages and farmlands | Good |
| 2249 | Hechi | Nandan County | Moraceae | *Ficus virens* Aiton. | 590 | 16.0 | 280.0 | 18.0 | Villages and farmlands | Good |
| 2250 | Hechi | Nandan County | Moraceae | *Ficus microcarpa* L. f. | 690 | 28.0 | 286.6 | 21.0 | Villages and farmlands | Good |
| 2251 | Hechi | Nandan County | Moraceae | *Ficus microcarpa* L. f. | 800 | 26.0 | 300.0 | 30.0 | Villages and farmlands | Good |
| 2252 | Hechi | Nandan County | Moraceae | *Ficus microcarpa* L. f. | 810 | 32.0 | 302.6 | 28.0 | Villages and farmlands | Good |
| 2253 | Hechi | Nandan County | Simaroubaceae | *Ailanthus altissima* (Mill.) Swingle | 600 | 40.0 | 150.0 | 36.0 | Villages and farmlands | Good |
| 2254 | Hechi | Nandan County | Fagaceae | *Quercus glauca* Thunb. | 600 | 32.0 | 185.0 | 25.0 | Villages and farmlands | Good |
| 2255 | Hechi | Nandan County | Juglandaceae | *Carya sinensis* Dode. | 600 | 35.0 | 167.2 | 25.0 | Residential districts | Good |
| 2256 | Hechi | Nandan County | Juglandaceae | *Carya sinensis* Dode. | 600 | 25.0 | 172.0 | 31.0 | Residential districts | Moderate |
| 2257 | Hechi | Nandan County | Fagaceae | *Castanopsis indica* (Roxb. ex Lindl.) A. DC. | 500 | 32.0 | 140.2 | 30.0 | Villages and farmlands | Good |
| 2258 | Hechi | Nandan County | Fagaceae | *Castanopsis indica* (Roxb. ex Lindl.) A. DC. | 500 | 19.0 | 170.0 | 35.0 | Villages and farmlands | Moderate |
| 2259 | Hechi | Nandan County | Pinaceae | *Keteleeria davidiana* var. *calcarea* (C. Y. Cheng & L. K. Fu) Silba | 600 | 16.0 | 110.0 | 26.0 | Villages and farmlands | Good |
| 2260 | Hechi | Nandan County | Cephalotaxaceae | *Cephalotaxus fortunei* Hook. | 500 | 21.0 | 98.7 | 12.0 | Villages and farmlands | Good |
| 2261 | Hechi | Tian'e County | Moraceae | *Ficus virens* Aiton. | 610 | 40.0 | 312.1 | 27.5 | Residential districts | Good |
| 2262 | Hechi | Tian'e County | Moraceae | *Ficus virens* Aiton. | 640 | 25.0 | 318.0 | 22.0 | Villages and farmlands | Good |
| 2263 | Hechi | Tian'e County | Moraceae | *Ficus virens* Aiton. | 800 | 27.6 | 360.0 | 23.5 | Villages and farmlands | Good |
| 2264 | Hechi | Tian'e County | Moraceae | *Ficus virens* Aiton. | 580 | 30.0 | 300.0 | 19.0 | Villages and farmlands | Moderate |
| 2265 | Hechi | Tian'e County | Moraceae | *Ficus virens* Aiton. | 800 | 26.0 | 360.0 | 15.5 | Villages and farmlands | Good |
| 2266 | Hechi | Tian'e County | Moraceae | *Ficus virens* Aiton. | 640 | 31.0 | 320.0 | 25.0 | Villages and farmlands | Good |
| 2267 | Hechi | Tian'e County | Moraceae | *Ficus virens* Aiton. | 590 | 29.0 | 300.0 | 16.5 | Villages and farmlands | Good |
| 2268 | Hechi | Tian'e County | Moraceae | *Ficus virens* Aiton. | 680 | 22.0 | 330.0 | 40.5 | Villages and farmlands | Good |
| 2269 | Hechi | Tian'e County | Moraceae | *Ficus virens* Aiton. | 520 | 17.0 | 280.0 | 23.5 | Villages and farmlands | Good |
| 2270 | Hechi | Tian'e County | Moraceae | *Ficus virens* Aiton. | 580 | 30.0 | 300.0 | 38.5 | Villages and farmlands | Good |
| 2271 | Hechi | Tian'e County | Moraceae | *Ficus virens* Aiton. | 580 | 34.0 | 304.9 | 23.0 | Villages and farmlands | Good |
| 2272 | Hechi | Tian'e County | Moraceae | *Ficus microcarpa* L. f. | 560 | 10.0 | 270.0 | 22.5 | Villages and farmlands | Good |
| 2273 | Hechi | Tian'e County | Moraceae | *Ficus virens* Aiton. | 600 | 30.0 | 363.1 | 31.0 | Villages and farmlands | Good |
| 2274 | Hechi | Tian'e County | Moraceae | *Ficus virens* Aiton. | 600 | 20.0 | 310.0 | 32.0 | Villages and farmlands | Good |
| 2275 | Hechi | Tian'e County | Taxaceae | *Taxus wallichiana* var. *mairei* (Lemée & H. Lév.) L. K. Fu & Nan Li | 530 | 20.0 | 107.6 | 14.0 | Wooded areas and plant nurseries | Good |
| 2276 | Hechi | Fengshan County | Moraceae | *Ficus virens* Aiton. | 550 | 30.0 | 268.0 | 26.5 | Villages and farmlands | Good |
| 2277 | Hechi | Fengshan County | Moraceae | *Ficus virens* Aiton. | 750 | 28.0 | 350.0 | 41.0 | Villages and farmlands | Good |
| 2278 | Hechi | Fengshan County | Moraceae | *Ficus virens* Aiton. | 550 | 20.0 | 270.0 | 14.5 | Villages and farmlands | Good |
| 2279 | Hechi | Fengshan County | Moraceae | *Ficus virens* Aiton. | 580 | 23.0 | 301.3 | 29.5 | Villages and farmlands | Good |
| 2280 | Hechi | Fengshan County | Fagaceae | *Quercus glauca* Thunb. | 500 | 30.0 | 152.0 | 15.5 | Villages and farmlands | Good |
| 2281 | Hechi | Fengshan County | Elaeocarpaceae | *Sloanea sinensis* (Hance) Hemsl. | 500 | 25.0 | 85.0 | 9.0 | Villages and farmlands | Good |
| 2282 | Hechi | Fengshan County | Moraceae | *Ficus virens* Aiton. | 600 | 22.0 | 308.0 | 21.5 | Villages and farmlands | Good |
| 2283 | Hechi | Fengshan County | Moraceae | *Ficus virens* Aiton. | 900 | 22.0 | 310.0 | 16.5 | Villages and farmlands | Good |
| 2284 | Hechi | Donglan County | Moraceae | *Ficus microcarpa* L. f. | 560 | 27.0 | 267.5 | 25.0 | Villages and farmlands | Good |
| 2285 | Hechi | Donglan County | Sapindaceae | *Dimocarpus longan* Lour. | 550 | 12.0 | 113.0 | 13.0 | Villages and farmlands | Good |
| 2286 | Hechi | Donglan County | Fagaceae | *Castanopsis hystrix* Hook. f. & Thomson ex A. DC. | 550 | 23.0 | 146.0 | 16.0 | Villages and farmlands | Good |
| 2287 | Hechi | Donglan County | Fagaceae | *Quercus glauca* Thunb. | 520 | 32.0 | 144.0 | 16.0 | Villages and farmlands | Good |
| 2288 | Hechi | Donglan County | Moraceae | *Ficus racemosa* L. | 500 | 25.0 | 201.0 | 23.5 | Villages and farmlands | Good |
| 2289 | Hechi | Donglan County | Moraceae | *Ficus virens* Aiton. | 800 | 20.0 | 354.0 | 32.0 | Residential districts | Good |
| 2290 | Hechi | Donglan County | Moraceae | *Ficus virens* Aiton. | 500 | 33.0 | 278.0 | 32.0 | Villages and farmlands | Good |
| 2291 | Hechi | Donglan County | Moraceae | *Ficus virens* Aiton. | 550 | 31.0 | 282.0 | 26.5 | Villages and farmlands | Good |
| 2292 | Hechi | Luocheng County | Moraceae | *Ficus microcarpa* L. f. | 591 | 19.1 | 267.0 | 39.1 | Residential districts | Good |
| 2293 | Hechi | Luocheng County | Moraceae | *Ficus microcarpa* L. f. | 591 | 19.8 | 192.0 | 27.8 | Residential districts | Good |
| 2294 | Hechi | Luocheng County | Moraceae | *Ficus microcarpa* L. f. | 591 | 18.5 | 143.0 | 18.6 | Residential districts | Good |
| 2295 | Hechi | Luocheng County | Oleaceae | *Ligustrum lucidum* W. T. Aiton. | 591 | 10.6 | 179.0 | 8.0 | Residential districts | Good |
| 2296 | Hechi | Luocheng County | Moraceae | *Ficus microcarpa* L. f. | 600 | 22.0 | 286.0 | 33.0 | Residential districts | Good |
| 2297 | Hechi | Luocheng County | Lauraceae | *Camphora officinarum Nees.* | 615 | 25.0 | 229.0 | 33.0 | Villages and farmlands | Good |
| 2298 | Hechi | Luocheng County | Lauraceae | *Camphora officinarum Nees.* | 610 | 13.0 | 232.0 | 13.5 | Villages and farmlands | Good |
| 2299 | Hechi | Luocheng County | Moraceae | *Ficus microcarpa* L. f. | 615 | 17.8 | 192.0 | 27.6 | Villages and farmlands | Good |
| 2300 | Hechi | Luocheng County | Moraceae | *Ficus microcarpa* L. f. | 615 | 17.6 | 219.0 | 29.5 | Villages and farmlands | Good |
| 2301 | Hechi | Luocheng County | Moraceae | *Ficus microcarpa* L. f. | 615 | 18.7 | 277.0 | 34.3 | Villages and farmlands | Good |
| 2302 | Hechi | Luocheng County | Moraceae | *Ficus microcarpa* L. f. | 615 | 11.0 | 197.0 | 1.9 | Villages and farmlands | Good |
| 2303 | Hechi | Luocheng County | Moraceae | *Ficus microcarpa* L. f. | 615 | 17.2 | 185.0 | 34.2 | Villages and farmlands | Good |
| 2304 | Hechi | Luocheng County | Moraceae | *Ficus microcarpa* L. f. | 615 | 16.0 | 121.0 | 23.4 | Villages and farmlands | Good |
| 2305 | Hechi | Luocheng County | Moraceae | *Ficus microcarpa* L. f. | 650 | 20.0 | 300.0 | 28.0 | Residential districts | Good |
| 2306 | Hechi | Luocheng County | Moraceae | *Ficus microcarpa* L. f. | 615 | 26.0 | 369.0 | 33.0 | Villages and farmlands | Good |
| 2307 | Hechi | Luocheng County | Fagaceae | *Castanopsis carlesii* (Hemsl.) Hayata | 615 | 18.0 | 191.0 | 17.0 | Villages and farmlands | Good |
| 2308 | Hechi | Luocheng County | Moraceae | *Ficus microcarpa* L. f. | 515 | 16.0 | 267.0 | 15.0 | Villages and farmlands | Good |
| 2309 | Hechi | Luocheng County | Moraceae | *Ficus microcarpa* L. f. | 700 | 18.0 | 292.0 | 26.0 | Villages and farmlands | Good |
| 2310 | Hechi | Luocheng County | Moraceae | *Ficus microcarpa* L. f. | 500 | 20.0 | 254.0 | 26.5 | Villages and farmlands | Good |
| 2311 | Hechi | Luocheng County | Moraceae | *Ficus microcarpa* L. f. | 650 | 20.0 | 296.0 | 19.0 | Villages and farmlands | Good |
| 2312 | Hechi | Luocheng County | Moraceae | *Ficus microcarpa* L. f. | 500 | 20.0 | 267.0 | 11.0 | Villages and farmlands | Good |
| 2313 | Hechi | Huanjiang County | Moraceae | *Ficus microcarpa* L. f. | 650 | 35.0 | 280.0 | 40.0 | Villages and farmlands | Good |
| 2314 | Hechi | Huanjiang County | Moraceae | *Ficus microcarpa* L. f. | 750 | 30.0 | 292.0 | 31.6 | Villages and farmlands | Good |
| 2315 | Hechi | Huanjiang County | Pinaceae | *Keteleeria fortunei* (A. Murray bis) Carrière | 500 | 20.1 | 113.6 | 17.2 | Villages and farmlands | Moderate |
| 2316 | Hechi | Huanjiang County | Moraceae | *Ficus virens* Aiton. | 630 | 18.2 | 312.6 | 42.7 | Residential districts | Good |
| 2317 | Hechi | Huanjiang County | Moraceae | *Ficus virens* Aiton. | 600 | 18.5 | 318.0 | 27.7 | Residential districts | Good |
| 2318 | Hechi | Huanjiang County | Moraceae | *Ficus microcarpa* L. f. | 600 | 17.6 | 272.0 | 32.5 | Villages and farmlands | Good |
| 2319 | Hechi | Huanjiang County | Moraceae | *Ficus microcarpa* L. f. | 700 | 13.5 | 230.0 | 20.3 | Villages and farmlands | Good |
| 2320 | Hechi | Huanjiang County | Moraceae | *Ficus virens* Aiton | 530 | 22.2 | 280.0 | 41.9 | Villages and farmlands | Good |
| 2321 | Hechi | Huanjiang County | Moraceae | *Ficus microcarpa* L. f. | 550 | 30.5 | 263.3 | 32.8 | Villages and farmlands | Good |
| 2322 | Hechi | Huanjiang County | Fagaceae | *Quercus glauca* Thunb. | 670 | 19.6 | 117.8 | 13.0 | Wooded areas and plant nurseries | Poor |
| 2323 | Hechi | Huanjiang County | Moraceae | *Ficus virens* Aiton. | 520 | 25.0 | 280.0 | 28.8 | Villages and farmlands | Good |
| 2324 | Hechi | Huanjiang County | Theaceae | *Schima superba* Gardner & Champ. | 500 | 17.1 | 120.1 | 21.2 | Villages and farmlands | Good |
| 2325 | Hechi | Huanjiang County | Moraceae | *Ficus microcarpa* L. f. | 500 | 15.2 | 254.7 | 43.7 | Residential districts | Good |
| 2326 | Hechi | Huanjiang County | Moraceae | *Ficus microcarpa* L. f. | 550 | 9.5 | 220.0 | 20.4 | Residential districts | Good |
| 2327 | Hechi | Huanjiang County | Moraceae | *Ficus microcarpa* L. f. | 550 | 12.3 | 200.0 | 34.8 | Residential districts | Good |
| 2328 | Hechi | Huanjiang County | Taxaceae | *Taxus wallichiana* var. *mairei* (Lemée & H. Lév.) L. K. Fu & Nan Li | 600 | 16.0 | 111.4 | 14.3 | Villages and farmlands | Good |
| 2329 | Hechi | Huanjiang County | Moraceae | *Ficus microcarpa* L. f. | 750 | 22.1 | 260.0 | 54.2 | Villages and farmlands | Good |
| 2330 | Hechi | Huanjiang County | Lauraceae | *Camphora officinarum Nees.* | 750 | 25.5 | 281.2 | 25.2 | Villages and farmlands | Good |
| 2331 | Hechi | Huanjiang County | Altingiaceae | *Liquidambar formosana* Hance. | 750 | 27.5 | 234.0 | 25.1 | Villages and farmlands | Good |
| 2332 | Hechi | Huanjiang County | Anacardiaceae | *Pistacia chinensis* Bunge. | 500 | 14.2 | 187.0 | 17.3 | Villages and farmlands | Good |
| 2333 | Hechi | Huanjiang County | Anacardiaceae | *Pistacia chinensis* Bunge. | 560 | 13.8 | 200.1 | 12.8 | Villages and farmlands | Moderate |
| 2334 | Hechi | Huanjiang County | Moraceae | *Ficus virens* Aiton. | 500 | 28.9 | 396.0 | 42.6 | Villages and farmlands | Good |
| 2335 | Hechi | Huanjiang County | Meliaceae | *Toona sinensis (*Juss.) Roem. | 600 | 14.5 | 195.0 | 15.9 | Villages and farmlands | Moderate |
| 2336 | Hechi | Huanjiang County | Meliaceae | *Toona ciliata* M. Roem. | 800 | 30.0 | 270.6 | 23.0 | Villages and farmlands | Good |
| 2337 | Hechi | Bama County | Moraceae | *Ficus virens* Aiton. | 700 | 33.2 | 350.0 | 41.4 | Villages and farmlands | Good |
| 2338 | Hechi | Bama County | Moraceae | *Ficus virens* Aiton. | 500 | 35.5 | 280.0 | 41.0 | Villages and farmlands | Good |
| 2339 | Hechi | Bama County | Moraceae | *Ficus virens* Aiton. | 500 | 23.3 | 276.0 | 35.3 | Villages and farmlands | Good |
| 2340 | Hechi | Bama County | Moraceae | *Ficus microcarpa* L. f. | 500 | 28.6 | 233.0 | 27.5 | Villages and farmlands | Good |
| 2341 | Hechi | Bama County | Moraceae | *Ficus microcarpa* L. f. | 500 | 30.6 | 274.0 | 26.1 | Villages and farmlands | Good |
| 2342 | Hechi | Bama County | Meliaceae | *Aphanamixis polystachya* (Wall.) R. Parker | 500 | 22.3 | 143.0 | 22.0 | Villages and farmlands | Good |
| 2343 | Hechi | Bama County | Moraceae | *Ficus virens* Aiton. | 500 | 21.6 | 280.0 | 40.1 | Villages and farmlands | Good |
| 2344 | Hechi | Bama County | Moraceae | *Ficus virens* Aiton. | 500 | 29.6 | 274.0 | 28.2 | Villages and farmlands | Good |
| 2345 | Hechi | Bama County | Moraceae | *Ficus virens* Aiton. | 500 | 34.5 | 281.0 | 27.3 | Villages and farmlands | Good |
| 2346 | Hechi | Bama County | Moraceae | *Ficus virens* Aiton. | 550 | 28.3 | 296.0 | 36.4 | Villages and farmlands | Good |
| 2347 | Hechi | Bama County | Moraceae | *Ficus virens* Aiton. | 500 | 40.6 | 293.0 | 35.2 | Villages and farmlands | Good |
| 2348 | Hechi | Bama County | Moraceae | *Ficus virens* Aiton. | 600 | 21.8 | 308.0 | 31.0 | Villages and farmlands | Good |
| 2349 | Hechi | Bama County | Fabaceae | *Lysidice rhodostegia* Hance. | 500 | 28.4 | 178.0 | 24.8 | Villages and farmlands | Good |
| 2350 | Hechi | Bama County | Moraceae | *Ficus microcarpa* L. f. | 500 | 26.4 | 278.0 | 41.7 | Villages and farmlands | Good |
| 2351 | Hechi | Bama County | Juglandaceae | *Carya sinensis* Dode. | 600 | 37.0 | 177.0 | 22.9 | Villages and farmlands | Poor |
| 2352 | Hechi | Bama County | Moraceae | *Ficus virens* Aiton. | 500 | 38.3 | 280.0 | 35.0 | Villages and farmlands | Good |
| 2353 | Hechi | Bama County | Moraceae | *Ficus virens* Aiton. | 500 | 28.8 | 298.0 | 39.1 | Villages and farmlands | Good |
| 2354 | Hechi | Du'an County | Sapotaceae | *Sinosideroxylon pedunculatum* (Hemsl.) H. Chuang | 500 | 23.0 | 184.7 | 10.0 | Villages and farmlands | Poor |
| 2355 | Hechi | Du'an County | Clusiaceae | *Garcinia paucinervis* Chun ex F. C. How | 560 | 16.0 | 89.0 | 11.5 | Villages and farmlands | Moderate |
| 2356 | Hechi | Du'an County | Juglandaceae | *Carya sinensis* Dode. | 600 | 24.0 | 165.0 | 20.0 | Villages and farmlands | Poor |
| 2357 | Hechi | Du'an County | Pinaceae | *Keteleeria davidiana* var. *calcarea* (C. Y. Cheng & L. K. Fu) Silba | 805 | 28.0 | 128.0 | 5.0 | Villages and farmlands | Good |
| 2358 | Hechi | Du'an County | Ebenaceae | *Diospyros japonica* Siebold & Zucc. | 600 | 26.0 | 130.5 | 19.0 | Villages and farmlands | Poor |
| 2359 | Hechi | Dahua County | Sapindaceae | *Dimocarpus longan* Lour. | 550 | 12.0 | 111.4 | 16.0 | Villages and farmlands | Good |
| 2360 | Hechi | Dahua County | Moraceae | *Ficus virens* Aiton. | 550 | 18.5 | 279.6 | 29.5 | Villages and farmlands | Good |
| 2361 | Hechi | Dahua County | Moraceae | *Ficus racemosa* L. | 500 | 20.0 | 207.9 | 26.7 | Villages and farmlands | Good |
| 2362 | Hechi | Dahua County | Juglandaceae | *Carya cathayensis* Sarg. | 600 | 27.0 | 210.1 | 32.0 | Villages and farmlands | Good |
| 2363 | Hechi | Dahua County | Lythraceae | *Lagerstroemia caudata* Chun & F. C. How ex S. K. Lee & L. F. Lau | 500 | 15.0 | 119.4 | 11.0 | Villages and farmlands | Moderate |
| 2364 | Hechi | Dahua County | Fabaceae | *Zenia insignis* Chun. | 560 | 40.0 | 200.0 | 12.5 | Villages and farmlands | Good |
| 2365 | Hechi | Dahua County | Fabaceae | *Zenia insignis* Chun. | 560 | 25.0 | 127.3 | 25.0 | Villages and farmlands | Good |
| 2366 | Hechi | Yizhou District | Moraceae | *Ficus virens* Aiton. | 550 | 25.0 | 289.0 | 14.0 | Villages and farmlands | Good |
| 2367 | Hechi | Yizhou District | Moraceae | *Ficus virens* Aiton. | 850 | 30.0 | 375.0 | 28.0 | Villages and farmlands | Good |
| 2368 | Hechi | Yizhou District | Moraceae | *Ficus virens* Aiton. | 650 | 30.0 | 324.0 | 30.8 | Villages and farmlands | Good |
| 2369 | Hechi | Yizhou District | Moraceae | *Ficus virens* Aiton. | 650 | 20.8 | 330.0 | 21.0 | Residential districts | Good |
| 2370 | Hechi | Yizhou District | Moraceae | *Ficus virens* Aiton. | 500 | 29.0 | 275.0 | 33.0 | Villages and farmlands | Good |
| 2371 | Hechi | Yizhou District | Moraceae | *Ficus virens* Aiton. | 580 | 23.0 | 304.0 | 28.0 | Villages and farmlands | Good |
| 2372 | Hechi | Yizhou District | Moraceae | *Ficus virens* Aiton. | 800 | 22.0 | 391.0 | 21.0 | Villages and farmlands | Good |
| 2373 | Hechi | Yizhou District | Moraceae | *Ficus virens* Aiton. | 550 | 22.0 | 300.0 | 30.0 | Residential districts | Good |
| 2374 | Hechi | Yizhou District | Moraceae | *Ficus virens* Aiton. | 650 | 22.0 | 326.0 | 39.0 | Villages and farmlands | Good |
| 2375 | Hechi | Yizhou District | Moraceae | *Ficus virens* Aiton. | 600 | 23.0 | 315.0 | 21.0 | Villages and farmlands | Good |
| 2376 | Hechi | Yizhou District | Moraceae | *Ficus virens* Aiton. | 550 | 19.0 | 292.0 | 25.5 | Villages and farmlands | Good |
| 2377 | Hechi | Yizhou District | Juglandaceae | *Ficus virens* Aiton. | 500 | 24.0 | 167.0 | 11.0 | Villages and farmlands | Good |
| 2378 | Hechi | Yizhou District | Moraceae | *Ficus virens* Aiton. | 650 | 17.0 | 324.0 | 28.0 | Villages and farmlands | Good |
| 2379 | Hechi | Yizhou District | Moraceae | *Ficus virens* Aiton. | 600 | 17.0 | 302.0 | 29.0 | Villages and farmlands | Good |
| 2380 | Hechi | Yizhou District | Moraceae | *Ficus virens* Aiton. | 750 | 25.0 | 350.0 | 23.5 | Villages and farmlands | Good |
| 2381 | Hechi | Yizhou District | Moraceae | *Ficus virens* Aiton. | 500 | 23.0 | 269.0 | 43.5 | Villages and farmlands | Good |
| 2382 | Hechi | Yizhou District | Moraceae | *Ficus virens* Aiton. | 580 | 23.0 | 283.0 | 29.8 | Villages and farmlands | Good |
| 2383 | Hechi | Yizhou District | Moraceae | *Ficus virens* Aiton. | 650 | 24.0 | 321.0 | 28.0 | Villages and farmlands | Good |
| 2384 | Hechi | Yizhou District | Moraceae | *Ficus virens* Aiton. | 600 | 20.0 | 312.0 | 19.5 | Villages and farmlands | Good |
| 2385 | Laibin | Xiangzhou County | Lauraceae | *Camphora officinarum Nees.* | 1000 | 27.0 | 330.0 | 20.0 | Villages and farmlands | Good |
| 2386 | Laibin | Xiangzhou County | Moraceae | *Ficus virens* Aiton. | 1000 | 39.0 | 436.0 | 31.0 | Villages and farmlands | Good |
| 2387 | Laibin | Xiangzhou County | Moraceae | *Ficus virens* Aiton. | 1000 | 24.0 | 382.0 | 35.0 | Residential districts | Good |
| 2388 | Laibin | Xiangzhou County | Moraceae | *Ficus concinna* (Miq.) Miq. | 1000 | 12.0 | 367.0 | 31.5 | Villages and farmlands | Good |
| 2389 | Laibin | Xiangzhou County | Moraceae | *Ficus concinna* (Miq.) Miq. | 1000 | 18.0 | 310.0 | 48.0 | Villages and farmlands | Good |
| 2390 | Laibin | Xiangzhou County | Moraceae | *Ficus virens* Aiton. | 1000 | 33.0 | 420.0 | 33.5 | Residential districts | Good |
| 2391 | Laibin | Xiangzhou County | Moraceae | *Ficus virens* Aiton. | 1200 | 27.0 | 430.0 | 32.0 | Villages and farmlands | Good |
| 2392 | Laibin | Xiangzhou County | Moraceae | *Ficus concinna* (Miq.) Miq. | 1000 | 12.0 | 315.0 | 28.0 | Villages and farmlands | Good |
| 2393 | Laibin | Xiangzhou County | Moraceae | *Ficus concinna* (Miq.) Miq. | 1000 | 20.0 | 490.0 | 38.5 | Villages and farmlands | Good |
| 2394 | Laibin | Xiangzhou County | Moraceae | *Ficus concinna* (Miq.) Miq. | 1000 | 12.0 | 310.0 | 39.5 | Villages and farmlands | Good |
| 2395 | Laibin | Xiangzhou County | Moraceae | *Ficus concinna* (Miq.) Miq. | 1000 | 12.0 | 340.0 | 26.0 | Villages and farmlands | Good |
| 2396 | Laibin | Xiangzhou County | Moraceae | *Ficus concinna* (Miq.) Miq. | 1000 | 25.0 | 320.0 | 31.0 | Villages and farmlands | Good |
| 2397 | Laibin | Xiangzhou County | Lauraceae | *Camphora officinarum Nees.* | 1000 | 27.0 | 330.0 | 20.0 | Villages and farmlands | Good |
| 2398 | Laibin | Xiangzhou County | Moraceae | *Ficus concinna* (Miq.) Miq. | 1000 | 17.0 | 310.0 | 21.0 | Villages and farmlands | Good |
| 2399 | Laibin | Jinxiu County | Taxaceae | *Taxus wallichiana* var. *mairei* (Lemée & H. Lév.) L. K. Fu & Nan Li | 1000 | 31.2 | 116.0 | 15.0 | Villages and farmlands | Good |
| 2400 | Laibin | Jinxiu County | Taxaceae | *Taxus wallichiana* var. *mairei* (Lemée & H. Lév.) L. K. Fu & Nan Li | 1000 | 25.0 | 79.0 | 13.0 | Villages and farmlands | Good |
| 2401 | Laibin | Jinxiu County | Moraceae | *Ficus concinna* (Miq.) Miq. | 1200 | 31.0 | 423.0 | 49.0 | Villages and farmlands | Good |
| 2402 | Laibin | Jinxiu County | Cupressaceae | *Cunninghamia lanceolata* (Lamb.) Hook. | 1010 | 51.8 | 175.1 | 15.8 | Villages and farmlands | Good |
| 2403 | Laibin | Jinxiu County | Pinaceae | *Nothotsuga longibracteata* (W. C. Cheng) Hu ex C. N. Page | 1200 | 8.1 | 101.0 | 6.0 | Wooded areas and plant nurseries | Poor |
| 2404 | Laibin | Xingbin District | Malvaceae | *Bombax ceiba* L. | 600 | 16.0 | 254.8 | 17.8 | Villages and farmlands | Good |
| 2405 | Laibin | Xingbin District | Moraceae | *Ficus racemosa* L. | 500 | 16.8 | 203.7 | 22.0 | Villages and farmlands | Good |
| 2406 | Laibin | Xingbin District | Moraceae | *Ficus microcarpa* L. f. | 600 | 14.0 | 207.0 | 12.8 | Villages and farmlands | Good |
| 2407 | Laibin | Xincheng County | Moraceae | *Ficus virens* Aiton | 650 | 22.0 | 255.0 | 23.0 | Residential districts | Good |
| 2408 | Laibin | Xincheng County | Oxalidaceae | *Averrhoa carambola* L. | 500 | 13.0 | 94.0 | 9.2 | Villages and farmlands | Good |
| 2409 | Laibin | Xincheng County | Oxalidaceae | *Averrhoa carambola* L. | 500 | 14.0 | 96.0 | 12.0 | Villages and farmlands | Moderate |
| 2410 | Laibin | Xincheng County | Lauraceae | *Camphora officinarum Nees.* | 550 | 23.0 | 239.0 | 28.5 | Villages and farmlands | Good |
| 2411 | Laibin | Xincheng County | Lauraceae | *Camphora officinarum Nees.* | 500 | 27.0 | 207.0 | 37.0 | Villages and farmlands | Good |
| 2412 | Laibin | Xincheng County | Moraceae | *Ficus virens* Aiton. | 500 | 16.0 | 258.0 | 19.0 | Villages and farmlands | Poor |
| 2413 | Laibin | Xiangzhou County | Phyllanthaceae | *Bischofia polycarpa* (H. Lév.) Airy Shaw | 550 | 35.0 | 245.0 | 17.5 | Villages and farmlands | Moderate |
| 2414 | Laibin | Xiangzhou County | Moraceae | *Ficus virens* Aiton. | 900 | 24.0 | 338.0 | 17.5 | Villages and farmlands | Good |
| 2415 | Laibin | Xiangzhou County | Lauraceae | *Camphora officinarum Nees.* | 540 | 32.0 | 245.2 | 21.5 | Residential districts | Good |
| 2416 | Laibin | Xiangzhou County | Moraceae | *Ficus virens* Aiton. | 900 | 35.0 | 318.5 | 33.0 | Villages and farmlands | Good |
| 2417 | Laibin | Xiangzhou County | Moraceae | *Ficus virens* Aiton. | 725 | 18.0 | 310.0 | 40.0 | Villages and farmlands | Good |
| 2418 | Laibin | Xiangzhou County | Fagaceae | *Quercus acutissima* Carruth. | 700 | 17.0 | 127.0 | 18.5 | Villages and farmlands | Moderate |
| 2419 | Laibin | Xiangzhou County | Moraceae | *Ficus virens* Aiton. | 590 | 35.0 | 289.8 | 33.0 | Villages and farmlands | Good |
| 2420 | Laibin | Xiangzhou County | Moraceae | *Ficus concinna (*Miq.) Miq. | 700 | 20.0 | 300.0 | 35.5 | Villages and farmlands | Good |
| 2421 | Laibin | Xiangzhou County | Moraceae | *Ficus virens* Aiton. | 600 | 25.0 | 312.1 | 32.0 | Villages and farmlands | Good |
| 2422 | Laibin | Xiangzhou County | Moraceae | *Ficus virens* Aiton. | 600 | 24.0 | 232.5 | 31.0 | Villages and farmlands | Good |
| 2423 | Laibin | Xiangzhou County | Moraceae | *Ficus virens* Aiton. | 600 | 24.5 | 277.1 | 31.5 | Villages and farmlands | Good |
| 2424 | Laibin | Xiangzhou County | Moraceae | *Ficus virens* Aiton. | 600 | 24.6 | 273.9 | 33.0 | Villages and farmlands | Good |
| 2425 | Laibin | Xiangzhou County | Moraceae | *Ficus virens* Aiton. | 600 | 25.5 | 310.5 | 32.5 | Villages and farmlands | Good |
| 2426 | Laibin | Xiangzhou County | Moraceae | *Ficus virens* Aiton. | 600 | 26.0 | 337.6 | 33.5 | Villages and farmlands | Good |
| 2427 | Laibin | Xiangzhou County | Moraceae | *Ficus virens* Aiton. | 600 | 23.6 | 312.1 | 31.0 | Villages and farmlands | Good |
| 2428 | Laibin | Xiangzhou County | Moraceae | *Ficus virens* Aiton. | 750 | 18.0 | 300.0 | 25.0 | Residential districts | Good |
| 2429 | Laibin | Xiangzhou County | Moraceae | *Ficus virens* Aiton. | 575 | 34.0 | 288.0 | 38.0 | Villages and farmlands | Good |
| 2430 | Laibin | Xiangzhou County | Moraceae | *Ficus virens* Aiton. | 550 | 20.0 | 267.0 | 23.0 | Villages and farmlands | Good |
| 2431 | Laibin | Xiangzhou County | Lauraceae | *Camphora officinarum Nees* | 555 | 25.0 | 250.0 | 18.5 | Villages and farmlands | Good |
| 2432 | Laibin | Xiangzhou County | Moraceae | *Ficus virens* Aiton. | 900 | 25.0 | 378.0 | 30.0 | Villages and farmlands | Good |
| 2433 | Laibin | Xiangzhou County | Moraceae | *Ficus concinna (*Miq.) Miq. | 525 | 16.0 | 270.0 | 23.0 | Villages and farmlands | Good |
| 2434 | Laibin | Xiangzhou County | Moraceae | *Ficus concinna (*Miq.) Miq. | 800 | 17.0 | 310.0 | 22.0 | Villages and farmlands | Good |
| 2435 | Laibin | Xiangzhou County | Moraceae | *Ficus concinna (*Miq.) Miq. | 530 | 18.0 | 270.0 | 30.0 | Villages and farmlands | Good |
| 2436 | Laibin | Xiangzhou County | Moraceae | *Ficus concinna (*Miq.) Miq. | 530 | 18.0 | 270.0 | 20.0 | Villages and farmlands | Good |
| 2437 | Laibin | Wuxuan County | Moraceae | *Ficus microcarpa* L. f. | 550 | 15.0 | 260.0 | 19.0 | Villages and farmlands | Good |
| 2438 | Laibin | Jinxiu County | Cupressaceae | *Cunninghamia lanceolata* (Lamb.) Hook. | 750 | 33.2 | 150.0 | 9.0 | Villages and farmlands | Good |
| 2439 | Laibin | Jinxiu County | Taxaceae | *Taxus wallichiana* var. *mairei* (Lemée & H. Lév.) L. K. Fu & Nan Li | 500 | 25.0 | 96.0 | 12.5 | Villages and farmlands | Good |
| 2440 | Laibin | Jinxiu County | Taxaceae | *Taxus wallichiana* var. *mairei* (Lemée & H. Lév.) L. K. Fu & Nan Li | 500 | 25.0 | 108.0 | 14.0 | Villages and farmlands | Good |
| 2441 | Laibin | Jinxiu County | Taxaceae | *Taxus wallichiana* var. *mairei* (Lemée & H. Lév.) L. K. Fu & Nan Li | 500 | 25.0 | 80.2 | 12.5 | Villages and farmlands | Good |
| 2442 | Laibin | Jinxiu County | Taxaceae | *Taxus wallichiana* var. *mairei* (Lemée & H. Lév.) L. K. Fu & Nan Li | 640 | 31.0 | 118.0 | 10.0 | Villages and farmlands | Good |
| 2443 | Laibin | Jinxiu County | Cupressaceae | *Cunninghamia lanceolata* (Lamb.) Hook. | 710 | 33.0 | 148.0 | 12.3 | Villages and farmlands | Good |
| 2444 | Laibin | Jinxiu County | Lauraceae | *Camphora officinarum Nees.* | 530 | 35.0 | 229.2 | 31.5 | Villages and farmlands | Good |
| 2445 | Laibin | Jinxiu County | Lauraceae | *Camphora officinarum Nees.* | 520 | 24.8 | 224.0 | 33.0 | Villages and farmlands | Good |
| 2446 | Laibin | Jinxiu County | Lauraceae | *Camphora officinarum Nees.* | 700 | 30.0 | 270.0 | 27.8 | Villages and farmlands | Good |
| 2447 | Laibin | Jinxiu County | Lauraceae | *Camphora officinarum Nees.* | 500 | 25.0 | 220.8 | 15.0 | Villages and farmlands | Good |
| 2448 | Laibin | Jinxiu County | Pinaceae | *Keteleeria fortunei* var. *cyclolepis* (Flous) Silba | 550 | 24.0 | 164.2 | 27.9 | Villages and farmlands | Poor |
| 2449 | Laibin | Jinxiu County | Podocarpaceae | *Dacrycarpus imbricatus* (Blume) de Laub. | 600 | 32.0 | 119.0 | 25.5 | Villages and farmlands | Good |
| 2450 | Laibin | Jinxiu County | Moraceae | *Ficus microcarpa* L. f. | 580 | 16.2 | 268.0 | 27.5 | Villages and farmlands | Good |
| 2451 | Laibin | Jinxiu County | Moraceae | *Ficus microcarpa* L. f. | 680 | 23.0 | 284.0 | 33.0 | Villages and farmlands | Good |
| 2452 | Laibin | Jinxiu County | Moraceae | *Ficus microcarpa* L. f. | 540 | 18.0 | 261.0 | 24.3 | Villages and farmlands | Good |
| 2453 | Laibin | Jinxiu County | Moraceae | *Ficus microcarpa* L. f. | 615 | 28.0 | 277.0 | 34.8 | Villages and farmlands | Good |
| 2454 | Laibin | Jinxiu County | Moraceae | *Ficus microcarpa* L. f. | 520 | 21.0 | 260.0 | 23.9 | Villages and farmlands | Good |
| 2455 | Laibin | Jinxiu County | Moraceae | *Ficus microcarpa* L. f. | 550 | 18.4 | 265.0 | 25.2 | Villages and farmlands | Good |
| 2456 | Laibin | Jinxiu County | Lauraceae | *Camphora officinarum Nees* | 720 | 28.0 | 273.0 | 30.5 | Villages and farmlands | Good |
| 2457 | Laibin | Jinxiu County | Pinaceae | *Nothotsuga longibracteata* (W. C. Cheng) Hu ex C. N. Page | 930 | 20.0 | 91.2 | 11.0 | Wooded areas and plant nurseries | Moderate |
| 2458 | Laibin | Jinxiu County | Pinaceae | *Cathaya argyrophylla* Chun & Kuang | 549 | 29.3 | 79.2 | 9.5 | Wooded areas and plant nurseries | Moderate |
| 2459 | Laibin | Jinxiu County | Pinaceae | *Tsuga chinensis* (Franch.) E. Pritz. | 576 | 14.0 | 57.0 | 10.1 | Wooded areas and plant nurseries | Moderate |
| 2460 | Laibin | Jinxiu County | Pinaceae | *Tsuga chinensis* (Franch.) E. Pritz. | 568 | 7.5 | 56.5 | 7.4 | Wooded areas and plant nurseries | Moderate |
| 2461 | Laibin | Jinxiu County | Pinaceae | *Tsuga chinensis* (Franch.) E. Pritz. | 650 | 7.3 | 56.6 | 7.8 | Wooded areas and plant nurseries | Moderate |
| 2462 | Laibin | Jinxiu County | Podocarpaceae | *Dacrycarpus imbricatus* (Blume) de Laub. | 650 | 35.0 | 120.0 | 14.5 | Wooded areas and plant nurseries | Good |
| 2463 | Chongzuo | Jiangzhou District | Moraceae | *Antiaris toxicaria* Lesch. | 1000 | 33.0 | 245.0 | 32.0 | Villages and farmlands | Moderate |
| 2464 | Chongzuo | Jiangzhou District | Moraceae | *Ficus altissima* Blume. | 1100 | 24.0 | 372.6 | 35.0 | Villages and farmlands | Poor |
| 2465 | Chongzuo | Jiangzhou District | Moraceae | *Ficus altissima* Blume. | 1000 | 31.0 | 381.5 | 35.3 | Villages and farmlands | Poor |
| 2466 | Chongzuo | Fusui County | Moraceae | *Ficus altissima* Blume. | 1005 | 32.6 | 162.1 | 35.0 | Villages and farmlands | Poor |
| 2467 | Chongzuo | Ningming County | Moraceae | *Ficus altissima* Blume. | 1700 | 34.0 | 162.4 | 60.8 | Villages and farmlands | Poor |
| 2468 | Chongzuo | Longzhou County | Malvaceae | *Excentrodendron tonkinense* (A. Chev.) H. T. Chang & R. H. Miao | 1000 | 30.0 | 222.9 | 30.0 | Villages and farmlands | Moderate |
| 2469 | Chongzuo | Longzhou County | Sapindaceae | *Litchi chinensis* Sonn. | 1000 | 18.0 | 239.0 | 29.0 | Villages and farmlands | Good |
| 2470 | Chongzuo | Longzhou County | Malvaceae | *Excentrodendron* *tonkinense* (A. Chev.) H. T. Chang & R. H. Miao | 1500 | 28.0 | 215.0 | 18.5 | Villages and farmlands | Good |
| 2471 | Chongzuo | Longzhou County | Malvaceae | *Excentrodendron* *tonkinense* (A. Chev.) H. T. Chang & R. H. Miao | 1300 | 22.0 | 232.2 | 23.5 | Villages and farmlands | Good |
| 2472 | Chongzuo | Longzhou County | Malvaceae | *Excentrodendron* *tonkinense* (A. Chev.) H. T. Chang & R. H. Miao | 1000 | 28.0 | 210.2 | 31.0 | Villages and farmlands | Moderate |
| 2473 | Chongzuo | Longzhou County | Malvaceae | *Excentrodendron* *tonkinense* (A. Chev.) H. T. Chang & R. H. Miao | 1000 | 20.0 | 157.0 | 10.0 | Villages and farmlands | Good |
| 2474 | Chongzuo | Longzhou County | Malvaceae | *Excentrodendron* *tonkinense* (A. Chev.) H. T. Chang & R. H. Miao | 1000 | 32.0 | 181.5 | 16.5 | Villages and farmlands | Good |
| 2475 | Chongzuo | Longzhou County | Malvaceae | *Excentrodendron* *tonkinense* (A. Chev.) H. T. Chang & R. H. Miao | 1100 | 20.0 | 161.1 | 10.0 | Wooded areas and plant nurseries | Good |
| 2476 | Chongzuo | Longzhou County | Malvaceae | *Excentrodendron* *tonkinense* (A. Chev.) H. T. Chang & R. H. Miao | 1000 | 22.0 | 152.8 | 15.0 | Wooded areas and plant nurseries | Good |
| 2477 | Chongzuo | Longzhou County | Malvaceae | *Excentrodendron* *tonkinense* (A. Chev.) H. T. Chang & R. H. Miao | 2300 | 48.0 | 299.0 | 16.0 | Wooded areas and plant nurseries | Moderate |
| 2478 | Chongzuo | Longzhou County | Malvaceae | *Excentrodendron* *tonkinense* (A. Chev.) H. T. Chang & R. H. Miao | 1000 | 32.0 | 185.0 | 10.0 | Wooded areas and plant nurseries | Good |
| 2479 | Chongzuo | Daxin County | Malvaceae | *Excentrodendron* *tonkinense* (A. Chev.) H. T. Chang & R. H. Miao | 1510 | 58.0 | 246.0 | 25.4 | Villages and farmlands | Good |
| 2480 | Chongzuo | Daxin County | Malvaceae | *Excentrodendron* *tonkinense* (A. Chev.) H. T. Chang & R. H. Miao | 1510 | 41.0 | 245.0 | 38.6 | Villages and farmlands | Good |
| 2481 | Chongzuo | Daxin County | Malvaceae | *Excentrodendron* *tonkinense* (A. Chev.) H. T. Chang & R. H. Miao | 1200 | 38.2 | 218.7 | 24.7 | Villages and farmlands | Good |
| 2482 | Chongzuo | Daxin County | Clusiaceae | *Garcinia paucinervis* Chun ex F. C. How | 1000 | 37.0 | 112.0 | 23.0 | Villages and farmlands | Good |
| 2483 | Chongzuo | Daxin County | Malvaceae | *Excentrodendron tonkinense* (A. Chev.) H. T. Chang & R. H. Miao | 1000 | 40.8 | 178.3 | 24.6 | Villages and farmlands | Good |
| 2484 | Chongzuo | Daxin County | Malvaceae | *Excentrodendron tonkinense* (A. Chev.) H. T. Chang & R. H. Miao | 1200 | 18.2 | 206.9 | 14.9 | Villages and farmlands | Good |
| 2485 | Chongzuo | Daxin County | Malvaceae | *Excentrodendron tonkinense* (A. Chev.) H. T. Chang & R. H. Miao | 1000 | 36.3 | 104.1 | 28.5 | Villages and farmlands | Good |
| 2486 | Chongzuo | Daxin County | Clusiaceae | *Garcinia paucinervis* Chun ex F. C. How | 1000 | 34.2 | 99.0 | 16.4 | Villages and farmlands | Good |
| 2487 | Chongzuo | Daxin County | Malvaceae | *Excentrodendron tonkinense* (A. Chev.) H. T. Chang & R. H. Miao | 1000 | 38.0 | 103.0 | 27.9 | Villages and farmlands | Good |
| 2488 | Chongzuo | Tiandeng County | Malvaceae | *Excentrodendron tonkinense* (A. Chev.) H. T. Chang & R. H. Miao | 1920 | 46.0 | 279.0 | 25.0 | Villages and farmlands | Good |
| 2489 | Chongzuo | Tiandeng County | Malvaceae | *Excentrodendron tonkinense* (A. Chev.) H. T. Chang & R. H. Miao | 1000 | 20.0 | 192.7 | 9.0 | Villages and farmlands | Good |
| 2490 | Chongzuo | Tiandeng County | Moraceae | *Ficus racemosa* L. | 1000 | 16.0 | 365.0 | 15.5 | Villages and farmlands | Good |
| 2491 | Chongzuo | Tiandeng County | Malvaceae | *Excentrodendron tonkinense* (A. Chev.) H. T. Chang & R. H. Miao | 1000 | 33.0 | 175.2 | 15.0 | Villages and farmlands | Good |
| 2492 | Chongzuo | Tiandeng County | Malvaceae | *Excentrodendron tonkinense* (A. Chev.) H. T. Chang & R. H. Miao | 1000 | 19.0 | 165.6 | 17.5 | Villages and farmlands | Good |
| 2493 | Chongzuo | Tiandeng County | Moraceae | *Ficus virens* Aiton | 1000 | 22.0 | 324.8 | 22.0 | Villages and farmlands | Moderate |
| 2494 | Chongzuo | Jiangzhou District | Cycadaceae | *Cycas pectinata* Griff. | 500 | 6.0 | 45.0 | 5.0 | Residential districts | Good |
| 2495 | Chongzuo | Jiangzhou District | Cycadaceae | *Cycas pectinata* Griff. | 500 | 6.0 | 40.0 | 5.5 | Residential districts | Good |
| 2496 | Chongzuo | Jiangzhou District | Sapindaceae | *Dimocarpus longan* Lour. | 550 | 12.0 | 111.5 | 14.0 | Villages and farmlands | Moderate |
| 2497 | Chongzuo | Jiangzhou District | Moraceae | *Ficus altissima* Blume. | 650 | 24.0 | 300.7 | 44.5 | Villages and farmlands | Moderate |
| 2498 | Chongzuo | Jiangzhou District | Moraceae | *Ficus altissima* Blume. | 550 | 24.0 | 280.0 | 35.0 | Villages and farmlands | Moderate |
| 2499 | Chongzuo | Jiangzhou District | Moraceae | *Ficus altissima* Blume. | 550 | 28.0 | 280.0 | 41.5 | Villages and farmlands | Moderate |
| 2500 | Chongzuo | Jiangzhou District | Moraceae | *Ficus altissima* Blume. | 650 | 23.0 | 300.0 | 43.5 | Villages and farmlands | Moderate |
| 2501 | Chongzuo | Jiangzhou District | Moraceae | *Ficus altissima* Blume. | 550 | 19.0 | 292.9 | 51.5 | Villages and farmlands | Moderate |
| 2502 | Chongzuo | Jiangzhou District | Moraceae | *Ficus altissima* Blume. | 550 | 24.0 | 370.0 | 30.5 | Residential districts | Moderate |
| 2503 | Chongzuo | Jiangzhou District | Moraceae | *Ficus altissima* Blume. | 800 | 25.0 | 318.4 | 37.5 | Villages and farmlands | Moderate |
| 2504 | Chongzuo | Jiangzhou District | Moraceae | *Ficus altissima* Blume. | 500 | 19.0 | 270.7 | 34.0 | Villages and farmlands | Moderate |
| 2505 | Chongzuo | Jiangzhou District | Moraceae | *Ficus altissima* Blume. | 750 | 24.0 | 318.0 | 34.5 | Villages and farmlands | Moderate |
| 2506 | Chongzuo | Jiangzhou District | Moraceae | *Ficus altissima* Blume. | 600 | 25.0 | 287.0 | 24.0 | Villages and farmlands | Moderate |
| 2507 | Chongzuo | Jiangzhou District | Moraceae | *Ficus altissima* Blume. | 750 | 21.0 | 312.1 | 31.5 | Residential districts | Moderate |
| 2508 | Chongzuo | Fusui County | Moraceae | *Ficus altissima* Blume. | 500 | 38.6 | 263.5 | 43.5 | Villages and farmlands | Moderate |
| 2509 | Chongzuo | Fusui County | Sapindaceae | *Dimocarpus longan* Lour. | 700 | 13.2 | 125.0 | 13.4 | Villages and farmlands | Good |
| 2510 | Chongzuo | Fusui County | Phyllanthaceae | *Bischofia javanica* Blume. | 500 | 23.2 | 246.0 | 21.8 | Villages and farmlands | Good |
| 2511 | Chongzuo | Fusui County | Moraceae | *Ficus altissima* Blume. | 660 | 37.3 | 300.5 | 61.8 | Villages and farmlands | Moderate |
| 2512 | Chongzuo | Fusui County | Fagaceae | *Castanopsis hystrix* Hook. f. & Thomson ex A. DC. | 670 | 23.3 | 165.0 | 23.7 | Villages and farmlands | Good |
| 2513 | Chongzuo | Fusui County | Myrtaceae | *Syzygium levinei* (Merr.) Merr. & L. M. Perry | 650 | 22.3 | 119.7 | 17.9 | Villages and farmlands | Good |
| 2514 | Chongzuo | Fusui County | Sapindaceae | *Dimocarpus longan* Lour. | 505 | 13.6 | 111.5 | 18.0 | Villages and farmlands | Moderate |
| 2515 | Chongzuo | Fusui County | Moraceae | *Ficus altissima* Blume. | 815 | 26.2 | 321.6 | 32.2 | Villages and farmlands | Moderate |
| 2516 | Chongzuo | Fusui County | Sapindaceae | *Dimocarpus longan* Lour. | 630 | 5.8 | 120.7 | 3.1 | Villages and farmlands | Moderate |
| 2517 | Chongzuo | Fusui County | Moraceae | *Ficus altissima* Blume. | 600 | 5.2 | 186.0 | 2.4 | Villages and farmlands | Moderate |
| 2518 | Chongzuo | Ningming County | Moraceae | *Ficus altissima* Blume. | 610 | 16.5 | 286.6 | 17.0 | Residential districts | Moderate |
| 2519 | Chongzuo | Ningming County | Moraceae | *Ficus altissima* Blume. | 500 | 18.5 | 267.5 | 47.5 | Villages and farmlands | Moderate |
| 2520 | Chongzuo | Ningming County | Anacardiaceae | *Dracontomelon duperreanum* Pierre | 500 | 50.5 | 223.0 | 24.0 | Villages and farmlands | Good |
| 2521 | Chongzuo | Ningming County | Anacardiaceae | *Dracontomelon duperreanum* Pierre | 550 | 55.0 | 255.0 | 42.0 | Villages and farmlands | Moderate |
| 2522 | Chongzuo | Ningming County | Anacardiaceae | *Dracontomelon duperreanum* Pierre | 800 | 45.0 | 430.0 | 37.0 | Villages and farmlands | Moderate |
| 2523 | Chongzuo | Ningming County | Anacardiaceae | *Dracontomelon duperreanum* Pierre | 750 | 58.6 | 450.0 | 41.5 | Villages and farmlands | Moderate |
| 2524 | Chongzuo | Ningming County | Moraceae | *Ficus altissima* Blume. | 700 | 27.3 | 300.2 | 35.7 | Villages and farmlands | Good |
| 2525 | Chongzuo | Ningming County | Moraceae | *Ficus altissima* Blume. | 550 | 36.5 | 267.5 | 36.9 | Villages and farmlands | Good |
| 2526 | Chongzuo | Ningming County | Moraceae | *Ficus altissima* Blume. | 510 | 38.7 | 175.2 | 64.0 | Villages and farmlands | Good |
| 2527 | Chongzuo | Ningming County | Moraceae | *Ficus altissima* Blume. | 580 | 43.0 | 302.0 | 31.4 | Villages and farmlands | Good |
| 2528 | Chongzuo | Ningming County | Moraceae | *Ficus altissima* Blume. | 590 | 16.2 | 293.0 | 23.3 | Villages and farmlands | Good |
| 2529 | Chongzuo | Ningming County | Moraceae | *Ficus altissima* Blume. | 500 | 22.4 | 267.0 | 23.3 | Residential districts | Good |
| 2530 | Chongzuo | Ningming County | Moraceae | *Ficus altissima* Blume. | 560 | 45.0 | 280.0 | 45.0 | Wooded areas and plant nurseries | Good |
| 2531 | Chongzuo | Longzhou County | Moraceae | *Ficus altissima* Blume. | 580 | 24.0 | 286.6 | 16.0 | Villages and farmlands | Good |
| 2532 | Chongzuo | Longzhou County | Moraceae | *Ficus altissima* Blume. | 500 | 30.0 | 270.0 | 35.0 | Villages and farmlands | Good |
| 2533 | Chongzuo | Longzhou County | Malvaceae | *Excentrodendron tonkinense* (A. Chev.) H. T. Chang & R. H. Miao | 650 | 32.0 | 143.3 | 8.0 | Villages and farmlands | Good |
| 2534 | Chongzuo | Longzhou County | Moraceae | *Ficus altissima* Blume. | 900 | 27.0 | 382.2 | 36.5 | Villages and farmlands | Good |
| 2535 | Chongzuo | Longzhou County | Moraceae | *Ficus virens* Aiton. | 650 | 23.0 | 289.8 | 21.0 | Villages and farmlands | Good |
| 2536 | Chongzuo | Longzhou County | Moraceae | *Antiaris toxicaria* Lesch. | 900 | 29.0 | 248.4 | 11.0 | Villages and farmlands | Good |
| 2537 | Chongzuo | Longzhou County | Anacardiaceae | *Dracontomelon duperreanum* Pierre | 500 | 24.0 | 242.0 | 19.0 | Villages and farmlands | Good |
| 2538 | Chongzuo | Longzhou County | Oxalidaceae | *Averrhoa carambola* L. | 500 | 15.0 | 108.3 | 13.0 | Villages and farmlands | Good |
| 2539 | Chongzuo | Longzhou County | Fabaceae | *Zenia insignis* Chun. | 500 | 30.0 | 180.0 | 42.5 | Villages and farmlands | Good |
| 2540 | Chongzuo | Longzhou County | Moraceae | *Ficus altissima* Blume. | 600 | 27.0 | 285.4 | 30.5 | Villages and farmlands | Good |
| 2541 | Chongzuo | Longzhou County | Moraceae | *Ficus altissima* Blume. | 700 | 19.0 | 302.6 | 38.0 | Villages and farmlands | Good |
| 2542 | Chongzuo | Longzhou County | Anacardiaceae | *Dracontomelon duperreanum* Pierre | 600 | 16.0 | 254.8 | 11.5 | Villages and farmlands | Good |
| 2543 | Chongzuo | Longzhou County | Sapindaceae | *Dimocarpus longan* Lour. | 500 | 18.0 | 119.4 | 16.5 | Villages and farmlands | Good |
| 2544 | Chongzuo | Longzhou County | Moraceae | *Ficus altissima* Blume. | 700 | 25.0 | 302.0 | 35.0 | Villages and farmlands | Good |
| 2545 | Chongzuo | Longzhou County | Sapindaceae | *Dimocarpus longan* Lour. | 750 | 22.0 | 127.7 | 18.0 | Villages and farmlands | Good |
| 2546 | Chongzuo | Longzhou County | Moraceae | *Ficus altissima* Blume | 500 | 36.0 | 120.1 | 44.0 | Villages and farmlands | Good |
| 2547 | Chongzuo | Longzhou County | Anacardiaceae | *Dracontomelon duperreanum* Pierre | 700 | 30.0 | 296.0 | 15.5 | Wooded areas and plant nurseries | Good |
| 2548 | Chongzuo | Longzhou County | Malvaceae | *Excentrodendron tonkinense* (A. Chev.) H. T. Chang & R. H. Miao | 800 | 17.0 | 124.2 | 9.0 | Wooded areas and plant nurseries | Good |
| 2549 | Chongzuo | Longzhou County | Malvaceae | *Excentrodendron tonkinense* (A. Chev.) H. T. Chang & R. H. Miao | 585 | 19.0 | 131.0 | 10.0 | Wooded areas and plant nurseries | Good |
| 2550 | Chongzuo | Longzhou County | Malvaceae | *Excentrodendron tonkinense* (A. Chev.) H. T. Chang & R. H. Miao | 759 | 32.0 | 158.0 | 14.5 | Wooded areas and plant nurseries | Good |
| 2551 | Chongzuo | Daxin County | Moraceae | *Ficus altissima* Blume. | 500 | 28.0 | 428.0 | 36.1 | Villages and farmlands | Good |
| 2552 | Chongzuo | Daxin County | Moraceae | *Ficus altissima* Blume. | 500 | 38.0 | 95.5 | 45.5 | Villages and farmlands | Good |
| 2553 | Chongzuo | Daxin County | Moraceae | *Ficus altissima* Blume. | 800 | 39.0 | 318.3 | 54.5 | Villages and farmlands | Good |
| 2554 | Chongzuo | Daxin County | Moraceae | *Ficus altissima* Blume. | 500 | 26.3 | 264.2 | 47.6 | Villages and farmlands | Good |
| 2555 | Chongzuo | Daxin County | Moraceae | *Ficus altissima* Blume. | 500 | 31.1 | 287.0 | 49.4 | Residential districts | Good |
| 2556 | Chongzuo | Daxin County | Sapindaceae | *Dimocarpus longan* Lour. | 550 | 6.3 | 114.6 | 6.5 | Villages and farmlands | Good |
| 2557 | Chongzuo | Daxin County | Sapindaceae | *Dimocarpus longan* Lour. | 750 | 15.3 | 128.6 | 13.9 | Villages and farmlands | Good |
| 2558 | Chongzuo | Daxin County | Moraceae | *Ficus altissima* Blume. | 650 | 29.0 | 299.0 | 25.7 | Villages and farmlands | Good |
| 2559 | Chongzuo | Daxin County | Moraceae | *Ficus altissima* Blume. | 500 | 27.1 | 263.0 | 33.2 | Villages and farmlands | Good |
| 2560 | Chongzuo | Daxin County | Sapindaceae | *Dimocarpus longan* Lour. | 500 | 14.8 | 111.4 | 11.5 | Villages and farmlands | Good |
| 2561 | Chongzuo | Daxin County | Sapindaceae | *Dimocarpus longan* Lour. | 650 | 10.8 | 120.9 | 9.6 | Villages and farmlands | Good |
| 2562 | Chongzuo | Daxin County | Apocynaceae | *Alstonia scholaris* (L.) R. Br. | 500 | 32.6 | 195.1 | 14.4 | Villages and farmlands | Good |
| 2563 | Chongzuo | Daxin County | Moraceae | *Ficus altissima* Blume. | 500 | 30.3 | 315.2 | 54.1 | Villages and farmlands | Good |
| 2564 | Chongzuo | Daxin County | Moraceae | *Ficus altissima* Blume. | 550 | 33.1 | 283.0 | 40.4 | Villages and farmlands | Good |
| 2565 | Chongzuo | Daxin County | Malvaceae | *Excentrodendron tonkinense* (A. Chev.) H. T. Chang & R. H. Miao | 550 | 42.0 | 127.3 | 26.5 | Villages and farmlands | Good |
| 2566 | Chongzuo | Daxin County | Malvaceae | *Excentrodendron tonkinense* (A. Chev.) H. T. Chang & R. H. Miao | 690 | 22.0 | 81.1 | 15.8 | Villages and farmlands | Good |
| 2567 | Chongzuo | Daxin County | Malvaceae | *Excentrodendron tonkinense* (A. Chev.) H. T. Chang & R. H. Miao | 750 | 47.0 | 149.0 | 30.1 | Villages and farmlands | Good |
| 2568 | Chongzuo | Daxin County | Malvaceae | *Excentrodendron tonkinense* (A. Chev.) H. T. Chang & R. H. Miao | 700 | 55.0 | 146.0 | 26.1 | Villages and farmlands | Good |
| 2569 | Chongzuo | Daxin County | Malvaceae | *Excentrodendron tonkinense* (A. Chev.) H. T. Chang & R. H. Miao | 600 | 32.8 | 133.1 | 21.1 | Villages and farmlands | Good |
| 2570 | Chongzuo | Daxin County | Malvaceae | *Excentrodendron tonkinense* (A. Chev.) H. T. Chang & R. H. Miao | 600 | 28.1 | 129.2 | 18.4 | Villages and farmlands | Poor |
| 2571 | Chongzuo | Daxin County | Malvaceae | *Excentrodendron tonkinense* (A. Chev.) H. T. Chang & R. H. Miao | 750 | 30.8 | 156.0 | 18.3 | Villages and farmlands | Good |
| 2572 | Chongzuo | Daxin County | Malvaceae | *Excentrodendron tonkinense* (A. Chev.) H. T. Chang & R. H. Miao | 700 | 32.3 | 149.6 | 23.4 | Villages and farmlands | Good |
| 2573 | Chongzuo | Daxin County | Sapindaceae | *Litchi chinensis* Sonn. | 500 | 23.1 | 126.1 | 44.3 | Villages and farmlands | Good |
| 2574 | Chongzuo | Daxin County | Malvaceae | *Excentrodendron tonkinense* (A. Chev.) H. T. Chang & R. H. Miao | 500 | 44.5 | 114.0 | 13.9 | Villages and farmlands | Good |
| 2575 | Chongzuo | Daxin County | Moraceae | *Ficus altissima* Blume. | 580 | 43.6 | 281.1 | 53.9 | Villages and farmlands | Moderate |
| 2576 | Chongzuo | Daxin County | Malvaceae | *Excentrodendron tonkinense* (A. Chev.) H. T. Chang & R. H. Miao | 500 | 25.4 | 115.7 | 18.4 | Villages and farmlands | Good |
| 2577 | Chongzuo | Daxin County | Sapindaceae | *Dimocarpus longan* Lour. | 700 | 21.2 | 125.1 | 19.1 | Villages and farmlands | Good |
| 2578 | Chongzuo | Daxin County | Malvaceae | *Excentrodendron tonkinense* (A. Chev.) H. T. Chang & R. H. Miao | 650 | 35.2 | 137.7 | 14.4 | Villages and farmlands | Good |
| 2579 | Chongzuo | Daxin County | Malvaceae | *Excentrodendron tonkinense* (A. Chev.) H. T. Chang & R. H. Miao | 500 | 42.3 | 107.9 | 15.8 | Villages and farmlands | Good |
| 2580 | Chongzuo | Daxin County | Malvaceae | *Bombax ceiba* L. | 500 | 36.0 | 169.0 | 33.0 | Villages and farmlands | Good |
| 2581 | Chongzuo | Daxin County | Sapindaceae | *Dimocarpus longan* Lour. | 500 | 15.0 | 101.9 | 13.5 | Villages and farmlands | Good |
| 2582 | Chongzuo | Daxin County | Malvaceae | *Excentrodendron* tonkinense (A. Chev.) H. T. Chang & R. H. Miao | 500 | 25.4 | 77.0 | 14.0 | Villages and farmlands | Good |
| 2583 | Chongzuo | Daxin County | Sapindaceae | *Dimocarpus longan* Lour. | 550 | 13.0 | 111.4 | 13.5 | Villages and farmlands | Good |
| 2584 | Chongzuo | Daxin County | Sapindaceae | *Dimocarpus longan* Lour. | 550 | 12.0 | 111.4 | 14.0 | Villages and farmlands | Good |
| 2585 | Chongzuo | Daxin County | Sapindaceae | *Dimocarpus longan* Lour. | 700 | 25.6 | 124.0 | 15.6 | Residential districts | Good |
| 2586 | Chongzuo | Daxin County | Sapindaceae | *Dimocarpus longan* Lour. | 550 | 12.0 | 113.0 | 12.3 | Residential districts | Good |
| 2587 | Chongzuo | Daxin County | Moraceae | *Ficus altissima* Blume. | 520 | 28.0 | 268.9 | 28.0 | Villages and farmlands | Good |
| 2588 | Chongzuo | Daxin County | Sapindaceae | *Dimocarpus longan* Lour. | 600 | 15.0 | 119.6 | 10.5 | Villages and farmlands | Good |
| 2589 | Chongzuo | Daxin County | Sapindaceae | *Dimocarpus longan* Lour. | 600 | 15.0 | 118.0 | 15.3 | Villages and farmlands | Good |
| 2590 | Chongzuo | Daxin County | Sapindaceae | *Dimocarpus longan* Lour. | 650 | 17.0 | 122.5 | 15.8 | Villages and farmlands | Good |
| 2591 | Chongzuo | Daxin County | Sapindaceae | *Dimocarpus longan* Lour. | 700 | 20.1 | 124.1 | 10.1 | Villages and farmlands | Good |
| 2592 | Chongzuo | Daxin County | Sapindaceae | *Dimocarpus longan* Lour. | 800 | 20.5 | 128.9 | 17.9 | Villages and farmlands | Good |
| 2593 | Chongzuo | Daxin County | Sapindaceae | *Dimocarpus longan* Lour. | 750 | 12.0 | 125.0 | 11.5 | Villages and farmlands | Good |
| 2594 | Chongzuo | Daxin County | Sapindaceae | *Dimocarpus longan* Lour. | 500 | 14.8 | 110.4 | 12.8 | Villages and farmlands | Good |
| 2595 | Chongzuo | Daxin County | Sapindaceae | *Dimocarpus longan* Lour. | 500 | 17.0 | 111.0 | 26.0 | Villages and farmlands | Good |
| 2596 | Chongzuo | Daxin County | Sapindaceae | *Dimocarpus longan* Lour. | 520 | 16.0 | 111.0 | 12.4 | Villages and farmlands | Good |
| 2597 | Chongzuo | Daxin County | Sapindaceae | *Dimocarpus longan* Lour. | 550 | 16.0 | 100.0 | 13.5 | Villages and farmlands | Good |
| 2598 | Chongzuo | Daxin County | Sapindaceae | *Dimocarpus longan* Lour. | 500 | 15.0 | 100.0 | 12.8 | Villages and farmlands | Good |
| 2599 | Chongzuo | Daxin County | Sapindaceae | *Dimocarpus longan* Lour. | 600 | 15.0 | 117.0 | 13.0 | Villages and farmlands | Good |
| 2600 | Chongzuo | Daxin County | Moraceae | *Ficus altissima* Blume. | 500 | 24.7 | 264.2 | 26.1 | Villages and farmlands | Good |
| 2601 | Chongzuo | Daxin County | Moraceae | *Ficus virens* Aiton. | 800 | 32.8 | 368.6 | 29.2 | Villages and farmlands | Good |
| 2602 | Chongzuo | Daxin County | Sapindaceae | *Dimocarpus longan* Lour. | 500 | 16.9 | 113.0 | 14.3 | Villages and farmlands | Good |
| 2603 | Chongzuo | Daxin County | Moraceae | *Ficus altissima* Blume. | 600 | 28.3 | 471.1 | 30.6 | Villages and farmlands | Good |
| 2604 | Chongzuo | Daxin County | Rubiaceae | *Tarennoidea wallichii* (Hook. f.) Tirveng. & Sastre | 500 | 35.5 | 143.2 | 9.3 | Villages and farmlands | Good |
| 2605 | Chongzuo | Daxin County | Fabaceae | *Adenanthera microsperma* Teijsm. & Binn. | 500 | 20.1 | 190.9 | 15.0 | Villages and farmlands | Poor |
| 2606 | Chongzuo | Tiandeng County | Malvaceae | *Excentrodendron tonkinense* (A. Chev.) H. T. Chang & R. H. Miao | 660 | 48.0 | 143.0 | 14.5 | Villages and farmlands | Good |
| 2607 | Chongzuo | Tiandeng County | Moraceae | *Ficus virens* Aiton. | 550 | 36.0 | 296.0 | 36.0 | Villages and farmlands | Good |
| 2608 | Chongzuo | Tiandeng County | Malvaceae | *Excentrodendron tonkinense* (A. Chev.) H. T. Chang & R. H. Miao | 550 | 22.0 | 125.0 | 15.0 | Villages and farmlands | Good |
| 2609 | Chongzuo | Tiandeng County | Moraceae | *Ficus virens* Aiton. | 550 | 32.0 | 299.0 | 26.0 | Villages and farmlands | Moderate |
| 2610 | Chongzuo | Tiandeng County | Moraceae | *Ficus virens* Aiton. | 900 | 35.0 | 386.0 | 30.5 | Villages and farmlands | Good |
| 2611 | Chongzuo | Tiandeng County | Moraceae | *Ficus virens* Aiton. | 500 | 27.0 | 152.0 | 22.0 | Villages and farmlands | Good |
| 2612 | Chongzuo | Tiandeng County | Malvaceae | *Excentrodendron tonkinense* (A. Chev.) H. T. Chang & R. H. Miao | 540 | 36.0 | 124.5 | 15.0 | Villages and farmlands | Good |
| 2613 | Chongzuo | Tiandeng County | Malvaceae | *Excentrodendron tonkinense* (A. Chev.) H. T. Chang & R. H. Miao | 550 | 28.0 | 125.0 | 8.5 | Villages and farmlands | Good |
| 2614 | Chongzuo | Tiandeng County | Moraceae | *Ficus virens* Aiton. | 750 | 28.0 | 340.0 | 28.5 | Villages and farmlands | Good |
| 2615 | Chongzuo | Tiandeng County | Moraceae | *Ficus virens* Aiton. | 800 | 38.0 | 356.0 | 53.0 | Villages and farmlands | Good |
| 2616 | Chongzuo | Tiandeng County | Malvaceae | *Excentrodendron tonkinense* (A. Chev.) H. T. Chang & R. H. Miao | 540 | 25.0 | 122.0 | 11.5 | Villages and farmlands | Good |
| 2617 | Chongzuo | Tiandeng County | Malvaceae | *Excentrodendron tonkinense* (A. Chev.) H. T. Chang & R. H. Miao | 650 | 28.0 | 141.1 | 8.5 | Villages and farmlands | Good |
| 2618 | Chongzuo | Tiandeng County | Malvaceae | *Excentrodendron tonkinense* (A. Chev.) H. T. Chang & R. H. Miao | 670 | 35.0 | 143.6 | 8.0 | Villages and farmlands | Good |
| 2619 | Chongzuo | Tiandeng County | Malvaceae | *Excentrodendron tonkinense* (A. Chev.) H. T. Chang & R. H. Miao | 520 | 26.0 | 121.0 | 8.5 | Villages and farmlands | Good |
| 2620 | Chongzuo | Tiandeng County | Malvaceae | *Excentrodendron tonkinense* (A. Chev.) H. T. Chang & R. H. Miao | 570 | 36.0 | 128.0 | 8.5 | Villages and farmlands | Good |
| 2621 | Chongzuo | Tiandeng County | Malvaceae | *Excentrodendron tonkinense* (A. Chev.) H. T. Chang & R. H. Miao | 560 | 39.0 | 127.4 | 8.5 | Villages and farmlands | Good |
| 2622 | Chongzuo | Tiandeng County | Malvaceae | *Excentrodendron tonkinense* (A. Chev.) H. T. Chang & R. H. Miao | 760 | 29.0 | 162.0 | 36.0 | Villages and farmlands | Good |
| 2623 | Chongzuo | Tiandeng County | Malvaceae | *Excentrodendron tonkinense* (A. Chev.) H. T. Chang & R. H. Miao | 660 | 23.0 | 145.0 | 12.5 | Villages and farmlands | Good |
| 2624 | Chongzuo | Tiandeng County | Moraceae | *Ficus virens* Aiton. | 750 | 34.0 | 343.0 | 39.0 | Villages and farmlands | Good |
| 2625 | Chongzuo | Tiandeng County | Moraceae | *Ficus virens* Aiton. | 800 | 31.0 | 350.0 | 31.0 | Villages and farmlands | Good |
| 2626 | Chongzuo | Tiandeng County | Moraceae | *Ficus virens* Aiton. | 600 | 28.0 | 275.0 | 36.0 | Villages and farmlands | Good |
| 2627 | Chongzuo | Tiandeng County | Moraceae | *Ficus virens* Aiton. | 690 | 26.0 | 308.0 | 29.5 | Villages and farmlands | Good |
| 2628 | Chongzuo | Tiandeng County | Moraceae | *Ficus virens* Aiton. | 700 | 26.0 | 337.0 | 36.5 | Villages and farmlands | Good |
| 2629 | Chongzuo | Tiandeng County | Malvaceae | *Excentrodendron tonkinense* (A. Chev.) H. T. Chang & R. H. Miao | 600 | 15.0 | 140.2 | 8.0 | Villages and farmlands | Good |
| 2630 | Chongzuo | Pingxiang City | Moraceae | *Ficus altissima* Blume. | 550 | 34.5 | 129.5 | 50.4 | Wooded areas and plant nurseries | Moderate |
