# Supplementary material for: Conserving Large Old Trees in Guangxi, South China: Diversity, Distribution, and Preservation Strategies
Source: Ecol Evol. 2026 Feb 12;16(2):e73043. doi: 10.1002/ece3.73043 (PMC12900623; doi:10.1002/ece3.73043)
Supplement: Supplementary file 3 — Data S3: ece373043‐sup‐0003‐Supinfo03.docx. [file ECE3-16-e73043-s003.docx]

**Appendix**

**Table S1** Families and species with many large old trees in Guangxi. RF represents the relative frequency. RA represents the relative abundance. RD represents relative dominance. IV represents the importance value index.

| **Species** | **Family** | BA(m^2^) | **Number** | **RF** | **RA** | **RD** | **IV** |
| --- | --- | --- | --- | --- | --- | --- | --- |
| *Ficus virens* | Moraceae | 2617.16 | 421 | 0.79 | 0.16 | 0.30 | 41.39 |
| *Camphora officinarum* | Lauraceae | 1139.30 | 337 | 0.79 | 0.13 | 0.13 | 34.76 |
| *Ficus microcarpa* | Moraceae | 1111.17 | 211 | 0.79 | 0.08 | 0.13 | 33.05 |
| *Ficus concinna* | Moraceae | 1391.50 | 210 | 0.57 | 0.08 | 0.16 | 26.96 |
| *Ficus altissima* | Moraceae | 799.03 | 128 | 0.5 | 0.05 | 0.09 | 21.3 |
| *Cycas pectinata* | Cycadaceae | 23.30 | 119 | 0.14 | 0.05 | 0.00 | 6.36 |
| *Litchi chinensis* | Sapindaceae | 157.68 | 111 | 0.29 | 0.04 | 0.02 | 11.53 |
| *Dimocarpus longan* | Sapindaceae | 80.80 | 88 | 0.57 | 0.03 | 0.01 | 20.47 |
| *Tsuga chinensis* | Pinaceae | 22.61 | 86 | 0.14 | 0.03 | 0.00 | 5.94 |
| *Excentrodendron tonkinense* | Malvaceae | 151.20 | 78 | 0.21 | 0.03 | 0.02 | 8.7 |
| *Castanopsis hystrix* | Fagaceae | 106.33 | 74 | 0.57 | 0.03 | 0.01 | 20.39 |
| *Castanopsis sclerophylla* | Fagaceae | 82.84 | 58 | 0.14 | 0.02 | 0.01 | 5.81 |
| *Taxus wallichiana var.mairei* | Taxaceae | 39.23 | 57 | 0.36 | 0.02 | 0.00 | 12.78 |
| *Schima superba* | Theaceae | 54.64 | 46 | 0.5 | 0.02 | 0.01 | 17.46 |
| *Nothotsuga longibracteata* | Pinaceae | 17.61 | 38 | 0.21 | 0.01 | 0.00 | 7.69 |
| *Erythrophleum fordii* | Fabaceae | 30.99 | 24 | 0.5 | 0.01 | 0.00 | 17.09 |
| *Osmanthus fragrans* | Oleaceae | 17.21 | 24 | 0.29 | 0.01 | 0.00 | 9.89 |
| *Dacrycarpus imbricatus* | Podocarpaceae | 26.00 | 24 | 0.57 | 0.01 | 0.00 | 19.45 |
| *Cathaya argyrophylla* | Pinaceae | 4.31 | 22 | 0.14 | 0.01 | 0.00 | 5.06 |
| *Ginkgo biloba* | Ginkgoaceae | 48.45 | 20 | 0.07 | 0.01 | 0.01 | 2.82 |
| *Castanopsis chinensis* | Fagaceae | 37.61 | 19 | 0.29 | 0.01 | 0.00 | 9.91 |
| *Castanopsis carlesii* | Fagaceae | 31.39 | 18 | 0.29 | 0.01 | 0.00 | 9.87 |
| *Bischofia polycarpa* | Phyllanthaceae | 67.73 | 17 | 0.36 | 0.01 | 0.01 | 12.38 |
| *Carya sinensis* | Juglandaceae | 51.55 | 15 | 0.14 | 0.01 | 0.01 | 5.15 |
| *Castanopsis eyrei* | Fagaceae | 8.95 | 15 | 0.07 | 0.01 | 0.00 | 2.6 |
| *Averrhoa carambola* | Oxalidaceae | 11.18 | 14 | 0.5 | 0.01 | 0.00 | 16.89 |
| *Castanea henryi* | Fagaceae | 20.49 | 14 | 0.14 | 0.01 | 0.00 | 5.02 |
| *Liquidambar formosana* | Altingiaceae | 29.57 | 11 | 0.29 | 0 | 0.00 | 9.77 |
| *Ilex rotunda* | Aquifoliaceae | 10.21 | 11 | 0.29 | 0 | 0.00 | 9.7 |
| *Pistacia chinensis* | Anacardiaceae | 34.02 | 10 | 0.21 | 0 | 0.00 | 7.4 |
| *Cunninghamia lanceolata* | Cupressaceae | 15.62 | 10 | 0.21 | 0 | 0.00 | 7.33 |
| *Ficus racemosa* | Moraceae | 54.68 | 9 | 0.43 | 0 | 0.01 | 14.61 |
| *Quercus acutissima* | Fagaceae | 11.56 | 9 | 0.43 | 0 | 0.00 | 14.44 |
| *Quercus glauca* | Fagaceae | 14.69 | 9 | 0.21 | 0 | 0.00 | 7.31 |
| *Zelkova schneideriana* | Ulmaceae | 13.72 | 8 | 0.21 | 0 | 0.00 | 7.3 |
| *Garcinia paucinervis* | Clusiaceae | 6.82 | 8 | 0.21 | 0 | 0.00 | 7.27 |
| *Dracontomelon duperreanum* | Anacardiaceae | 60.25 | 8 | 0.14 | 0 | 0.01 | 5.09 |
| *Pinus kwangtungensis* | Pinaceae | 1.56 | 7 | 0.07 | 0 | 0.00 | 2.48 |
| *Styphnolobium japonicum* | Fabaceae | 13.32 | 7 | 0.21 | 0 | 0.00 | 7.28 |
| *Boniodendron minus* | Sapindaceae | 34.02 | 7 | 0.14 | 0 | 0.00 | 4.86 |
| *Castanopsis fargesii* | Fagaceae | 3.64 | 7 | 0.14 | 0 | 0.00 | 4.86 |
| *Acer coriaceifolium* | Sapindaceae | 6.24 | 7 | 0.07 | 0 | 0.00 | 2.49 |
| *Castanopsis tibetana* | Fagaceae | 6.77 | 6 | 0.14 | 0 | 0.00 | 4.86 |
| *Antiaris toxicaria* | Moraceae | 24.39 | 6 | 0.29 | 0 | 0.00 | 9.69 |
| *Pinus massoniana* | Pinaceae | 7.38 | 6 | 0.14 | 0 | 0.00 | 4.87 |
| *Fagus longipetiolata* | Fagaceae | 3.32 | 6 | 0.07 | 0 | 0.00 | 2.47 |
| *Pterocarya stenoptera* | Juglandaceae | 18.91 | 5 | 0.07 | 0 | 0.00 | 2.52 |
| *Pseudotsuga sinensis* | Pinaceae | 4.26 | 5 | 0.07 | 0 | 0.00 | 2.46 |
| *Phoebe bournei* | Lauraceae | 8.03 | 5 | 0.21 | 0 | 0.00 | 7.24 |
| *Bischofia javanica* | Phyllanthaceae | 22.35 | 5 | 0.29 | 0 | 0.00 | 9.67 |
| *Carya cathayensis* | Juglandaceae | 7.91 | 5 | 0.07 | 0 | 0.00 | 2.47 |
| *Quercus variabilis* | Fagaceae | 6.25 | 5 | 0.14 | 0 | 0.00 | 4.85 |
| *Diospyros decandra* | Ebenaceae | 4.09 | 5 | 0.07 | 0 | 0.00 | 2.46 |
| *Toona ciliata* | Meliaceae | 15.68 | 4 | 0.14 | 0 | 0.00 | 4.87 |
| *Castanopsis faberi* | Fagaceae | 9.49 | 4 | 0.21 | 0 | 0.00 | 7.23 |
| *Bombax ceiba* | Malvaceae | 19.15 | 4 | 0.21 | 0 | 0.00 | 7.27 |
| *Celtis sinensis* | Cannabaceae | 9.27 | 4 | 0.14 | 0 | 0.00 | 4.85 |
| *Vitex quinata* | Lamiaceae | 4.84 | 4 | 0.21 | 0 | 0.00 | 7.21 |
| *Gleditsia sinensis* | Fabaceae | 6.74 | 4 | 0.14 | 0 | 0.00 | 4.84 |
| *Liriodendron chinense* | Magnoliaceae | 2.16 | 3 | 0.14 | 0 | 0.00 | 4.81 |
| *Keteleeria davidiana var.calcarea* | Pinaceae | 3.98 | 3 | 0.14 | 0 | 0.00 | 4.81 |
| *Choerospondias axillaris* | Anacardiaceae | 14.21 | 3 | 0.21 | 0 | 0.00 | 7.23 |
| *Zenia insignis* | Fabaceae | 6.96 | 3 | 0.14 | 0 | 0.00 | 4.83 |
| *Keteleeria pubescens* | Pinaceae | 8.05 | 3 | 0.14 | 0 | 0.00 | 4.83 |
| *Elaeocarpus sylvestris* | Elaeocarpaceae | 3.68 | 3 | 0.21 | 0 | 0.00 | 7.19 |
| *Phoebe calcarea* | Lauraceae | 4.59 | 3 | 0.07 | 0 | 0.00 | 2.44 |
| *Keteleeria davidiana* | Pinaceae | 6.73 | 3 | 0.14 | 0 | 0.00 | 4.83 |
| *Lysidice rhodostegia* | Fabaceae | 7.08 | 3 | 0.21 | 0 | 0.00 | 7.21 |
| *Schima argentea* | Theaceae | 2.15 | 3 | 0.07 | 0 | 0.00 | 2.43 |
| *Hovenia acerba* | Rhamnaceae | 2.27 | 3 | 0.21 | 0 | 0.00 | 7.19 |
| *Quercus fabri* | Fagaceae | 3.29 | 2 | 0.14 | 0 | 0.00 | 4.8 |
| *Prunus dulcis* | Rosaceae | 7.57 | 2 | 0.07 | 0 | 0.00 | 2.43 |
| *Aphananthe aspera* | Cannabaceae | 1.87 | 2 | 0.14 | 0 | 0.00 | 4.79 |
| *Ilex chinensis* | Aquifoliaceae | 1.74 | 2 | 0.14 | 0 | 0.00 | 4.79 |
| *Photinia bodinieri* | Rosaceae | 6.01 | 2 | 0.07 | 0 | 0.00 | 2.43 |
| *Artocarpus parvus* | Moraceae | 2.05 | 2 | 0.14 | 0 | 0.00 | 4.79 |
| *Adenanthera microsperma* | Fabaceae | 3.44 | 2 | 0.14 | 0 | 0.00 | 4.8 |
| *Cephalotaxus hainanensis* | Cephalotaxaceae | 2.80 | 2 | 0.14 | 0 | 0.00 | 4.8 |
| *Ulmus parvifolia* | Ulmaceae | 2.19 | 2 | 0.07 | 0 | 0.00 | 2.41 |
| *Podocarpus macrophyllus* | Podocarpaceae | 1.98 | 2 | 0.14 | 0 | 0.00 | 4.79 |
| *Ligustrum lucidum* | Oleaceae | 4.58 | 2 | 0.14 | 0 | 0.00 | 4.8 |
| *Syzygium levinei* | Myrtaceae | 2.01 | 2 | 0.14 | 0 | 0.00 | 4.79 |
| *Alstonia scholaris* | Apocynaceae | 6.65 | 2 | 0.14 | 0 | 0.00 | 4.81 |
| *Sinosideroxylon pedunculatum* | Sapotaceae | 3.95 | 2 | 0.14 | 0 | 0.00 | 4.8 |
| *Manglietia aromatica* | Magnoliaceae | 5.30 | 2 | 0.14 | 0 | 0.00 | 4.81 |
| *Lithocarpus corneus* | Fagaceae | 3.75 | 2 | 0.07 | 0 | 0.00 | 2.42 |
| *Castanopsis indica* | Fagaceae | 3.81 | 2 | 0.07 | 0 | 0.00 | 2.42 |
| *Keteleeria fortunei* | Pinaceae | 4.06 | 2 | 0.07 | 0 | 0.00 | 2.42 |
| *Saraca dives* | Fabaceae | 4.42 | 2 | 0.07 | 0 | 0.00 | 2.42 |
| *Nageia nagi* | Podocarpaceae | 2.84 | 2 | 0.07 | 0 | 0.00 | 2.42 |
| *Cupressus funebris* | Cupressaceae | 1.99 | 1 | 0.07 | 0 | 0.00 | 2.4 |
| *Machilus leptophylla* | Lauraceae | 1.45 | 1 | 0.07 | 0 | 0.00 | 2.4 |
| *Platycladus orientalis* | Cupressaceae | 2.06 | 1 | 0.07 | 0 | 0.00 | 2.4 |
| *Sassafras tzumu* | Lauraceae | 0.52 | 1 | 0.07 | 0 | 0.00 | 2.4 |
| *Litsea glutinosa* | Lauraceae | 1.27 | 1 | 0.07 | 0 | 0.00 | 2.4 |
| *Platyosprion platycarpum* | Fabaceae | 1.79 | 1 | 0.07 | 0 | 0.00 | 2.4 |
| *Ailanthus altissima* | Simaroubaceae | 1.77 | 1 | 0.07 | 0 | 0.00 | 2.4 |
| *Calocedrus macrolepis* | Cupressaceae | 3.77 | 1 | 0.07 | 0 | 0.00 | 2.41 |
| *Ficus hookeriana* | Moraceae | 9.98 | 1 | 0.07 | 0 | 0.00 | 2.43 |
| *Aphananthe cuspidata* | Cannabaceae | 1.84 | 1 | 0.07 | 0 | 0.00 | 2.4 |
| *Quercus disciformis* | Fagaceae | 3.37 | 1 | 0.07 | 0 | 0.00 | 2.41 |
| *Artocarpus styracifolius* | Moraceae | 1.84 | 1 | 0.07 | 0 | 0.00 | 2.4 |
| *Fraxinus griffithii* | Oleaceae | 0.77 | 1 | 0.07 | 0 | 0.00 | 2.4 |
| *Lindera megaphylla* | Lauraceae | 2.59 | 1 | 0.07 | 0 | 0.00 | 2.4 |
| *Sloanea sinensis* | Elaeocarpaceae | 0.57 | 1 | 0.07 | 0 | 0.00 | 2.4 |
| *Morus cathayana* | Moraceae | 1.04 | 1 | 0.07 | 0 | 0.00 | 2.4 |
| *Dalbergia hupeana* | Fabaceae | 1.13 | 1 | 0.07 | 0 | 0.00 | 2.4 |
| *Xanthophyllum hainanense* | Polygalaceae | 0.46 | 1 | 0.07 | 0 | 0.00 | 2.4 |
| *Elaeocarpus limitaneus* | Elaeocarpaceae | 0.38 | 1 | 0.07 | 0 | 0.00 | 2.4 |
| *Loropetalum chinense* | Hamamelidaceae | 0.44 | 1 | 0.07 | 0 | 0.00 | 2.4 |
| *Keteleeria fortunei var.cyclolepis* | Pinaceae | 2.12 | 1 | 0.07 | 0 | 0.00 | 2.4 |
| *Craibiodendron stellatum* | Ericaceae | 1.00 | 1 | 0.07 | 0 | 0.00 | 2.4 |
| *Corylopsis sinensis* | Hamamelidaceae | 0.54 | 1 | 0.07 | 0 | 0.00 | 2.4 |
| *Tarennoidea wallichii* | Rubiaceae | 1.61 | 1 | 0.07 | 0 | 0.00 | 2.4 |
| *Cryptomeria japonica var.sinensis* | Cupressaceae | 1.13 | 1 | 0.07 | 0 | 0.00 | 2.4 |
| *Chukrasia tabularis* | Meliaceae | 2.94 | 1 | 0.07 | 0 | 0.00 | 2.4 |
| *Castanea seguinii* | Fagaceae | 1.15 | 1 | 0.07 | 0 | 0.00 | 2.4 |
| *Manglietia fordiana* | Magnoliaceae | 6.38 | 1 | 0.07 | 0 | 0.00 | 2.42 |
| *Ormosia pubescens* | Fabaceae | 1.77 | 1 | 0.07 | 0 | 0.00 | 2.4 |
| *Cephalotaxus fortunei* | Cephalotaxaceae | 0.77 | 1 | 0.07 | 0 | 0.00 | 2.4 |
| *Aphanamixis polystachya* | Meliaceae | 1.61 | 1 | 0.07 | 0 | 0.00 | 2.4 |
| *Diospyros japonica* | Ebenaceae | 1.34 | 1 | 0.07 | 0 | 0.00 | 2.4 |
| *Alphonsea mollis* | Annonaceae | 1.21 | 1 | 0.07 | 0 | 0.00 | 2.4 |
| *Mallotus repandus* | Euphorbiaceae | 0.83 | 1 | 0.07 | 0 | 0.00 | 2.4 |
| *Glyptostrobus pensilis* | Cupressaceae | 1.09 | 1 | 0.07 | 0 | 0.00 | 2.4 |
| *Syzygium nervosum* | Myrtaceae | 2.50 | 1 | 0.07 | 0 | 0.00 | 2.4 |
| *Cycas szechuanensis* | Cycadaceae | 0.08 | 1 | 0.07 | 0 | 0.00 | 2.39 |
| *Malania oleifera* | Olacaceae | 0.35 | 1 | 0.07 | 0 | 0.00 | 2.39 |
| *Pouteria annamensis* | Sapotaceae | 0.77 | 1 | 0.07 | 0 | 0.00 | 2.4 |
| *Manilkara hexandra* | Sapotaceae | 1.21 | 1 | 0.07 | 0 | 0.00 | 2.4 |
| *Aglaia lawii* | Meliaceae | 1.04 | 1 | 0.07 | 0 | 0.00 | 2.4 |
| *Parashorea chinensis* | Dipterocarpaceae | 1.50 | 1 | 0.07 | 0 | 0.00 | 2.4 |
| *Lagerstroemia caudata* | Lythraceae | 1.12 | 1 | 0.07 | 0 | 0.00 | 2.4 |
| *Syzygium euonymifolium* | Myrtaceae | 1.67 | 1 | 0.07 | 0 | 0.00 | 2.4 |
| *Machilus wenshanensis* | Lauraceae | 2.22 | 1 | 0.07 | 0 | 0.00 | 2.4 |
| *Diospyros eriantha* | Ebenaceae | 0.82 | 1 | 0.07 | 0 | 0.00 | 2.4 |
| *Ilex pentagona* | Aquifoliaceae | 1.09 | 1 | 0.07 | 0 | 0.00 | 2.4 |
| *Machilus rehderi* | Lauraceae | 2.25 | 1 | 0.07 | 0 | 0.00 | 2.4 |
| *Toona sinensis* | Meliaceae | 2.99 | 1 | 0.07 | 0 | 0.00 | 2.4 |
| *Lindera communis* | Lauraceae | 0.79 | 1 | 0.07 | 0 | 0.00 | 2.4 |
| *Helicia cochinchinensis* | Proteaceae | 0.94 | 1 | 0.07 | 0 | 0.00 | 2.4 |
| *Morella rubra* | Myricaceae | 2.84 | 1 | 0.07 | 0 | 0.00 | 2.4 |
| *Crateva religiosa* | Capparaceae | 0.92 | 1 | 0.07 | 0 | 0.00 | 2.4 |
| *Juniperus chinensis* | Cupressaceae | 1.06 | 1 | 0.07 | 0 | 0.00 | 2.4 |
| *Aesculus assamica* | Sapindaceae | 1.89 | 1 | 0.07 | 0 | 0.00 | 2.4 |
| *Handeliodendron bodinieri* | Sapindaceae | 0.48 | 1 | 0.07 | 0 | 0.00 | 2.4 |
| *Celtis biondii* | Cannabaceae | 1.91 | 1 | 0.07 | 0 | 0.00 | 2.4 |
| *Madhuca pasquieri* | Sapotaceae | 0.98 | 1 | 0.07 | 0 | 0.00 | 2.4 |
| *Phoebe sheareri* | Lauraceae | 1.33 | 1 | 0.07 | 0 | 0.00 | 2.4 |

**Table S2.** Distribution and abundance of large old trees across 14 prefecture-level cities in Guangxi.

| **Species** | **Nanning** | **Liuzhou** | **Guilin** | **Wuzhou** | **Beihai** | **Fangchenggang** | **Qinzhou** | **Guigang** | **Yulin** | **Baise** | **Hezhou** | **Hechi** | **Laibin** | **Chongzuo** |
| --- | --- | --- | --- | --- | --- | --- | --- | --- | --- | --- | --- | --- | --- | --- |
| *Ficus virens* | 6 | 20 | 1 | 5 |  |  | 6 | 16 | 5 | 233 |  | 94 | 21 | 14 |
| *Camphora officinarum* | 2 | 31 | 198 | 18 | 1 | 1 |  | 5 | 4 |  | 64 | 3 | 10 |  |
| *Ficus microcarpa* | 8 | 20 | 50 | 16 | 1 |  | 18 | 10 | 8 | 24 |  | 48 | 8 |  |
| *Ficus concinna* |  | 76 | 32 | 26 |  |  | 1 |  | 1 |  | 58 | 2 | 14 |  |
| *Ficus altissima* | 28 |  |  |  | 1 | 4 | 11 |  | 2 | 33 |  |  |  | 49 |
| *Cycas pectinata* | 117 |  |  |  |  |  |  |  |  |  |  |  |  | 2 |
| *Litchi chinensis* |  |  |  |  |  |  | 95 |  | 10 | 4 |  |  |  | 2 |
| *Dimocarpus longan* | 10 | 3 |  | 12 |  |  | 8 |  | 15 | 9 |  | 2 |  | 29 |
| *Tsuga chinensis* |  |  | 83 |  |  |  |  |  |  |  |  |  | 3 |  |
| *Excentrodendron tonkinense* | 10 |  |  |  |  |  |  |  |  | 17 |  |  |  | 51 |
| *Castanopsis hystrix* |  | 27 | 14 | 19 |  |  |  |  | 1 | 2 | 9 | 1 |  | 1 |
| *Castanopsis sclerophylla* |  |  | 2 |  |  |  |  |  |  |  | 56 |  |  |  |
| *Taxus wallichiana var.mairei* |  | 3 | 45 |  |  |  |  |  |  |  | 1 | 2 | 6 |  |
| *Schima superba* |  | 20 | 12 | 3 |  |  | 2 | 1 |  |  | 7 | 1 |  |  |
| *Nothotsuga longibracteata* | 3 |  | 33 |  |  |  |  |  |  |  |  |  | 2 |  |
| *Erythrophleum fordii* | 1 |  |  | 9 | 1 | 1 | 5 | 1 | 6 |  |  |  |  |  |
| *Osmanthus fragrans* |  |  | 15 | 1 |  |  |  |  | 1 |  | 7 |  |  |  |
| *Dacrycarpus imbricatus* |  | 8 | 2 | 3 |  | 1 | 1 | 4 |  |  | 3 |  | 2 |  |
| *Cathaya argyrophylla* |  |  | 21 |  |  |  |  |  |  |  |  |  | 1 |  |
| *Ginkgo biloba* |  |  | 20 |  |  |  |  |  |  |  |  |  |  |  |
| *Castanopsis chinensis* |  |  | 13 | 1 |  |  |  |  |  | 4 | 1 |  |  |  |
| *Castanopsis carlesii* |  |  | 7 |  |  |  |  |  |  | 4 | 6 | 1 |  |  |
| *Bischofia polycarpa* |  | 1 | 8 |  |  |  |  |  |  | 1 | 6 |  | 1 |  |
| *Carya sinensis* |  |  |  |  |  |  |  |  |  | 8 |  | 7 |  |  |
| *Castanopsis eyrei* | 15 |  |  |  |  |  |  |  |  |  |  |  |  |  |
| *Averrhoa carambola* | 5 |  |  | 3 |  | 1 |  | 1 |  | 1 |  |  | 2 | 1 |
| *Castanea henryi* |  | 1 | 13 |  |  |  |  |  |  |  |  |  |  |  |
| *Liquidambar formosana* |  | 2 | 3 |  |  |  |  |  |  | 3 |  | 3 |  |  |
| *Ilex rotunda* | 1 |  |  |  |  |  | 2 |  | 1 |  | 7 |  |  |  |
| *Pistacia chinensis* |  |  | 2 |  |  |  |  |  |  | 4 | 4 |  |  |  |
| *Cunninghamia lanceolata* |  | 2 | 5 |  |  |  |  |  |  |  |  |  | 3 |  |
| *Ficus racemosa* | 1 |  |  | 1 |  |  |  |  |  | 3 |  | 2 | 1 | 1 |
| *Quercus acutissima* |  | 1 | 4 |  |  |  |  | 1 |  | 1 | 1 |  | 1 |  |
| *Quercus glauca* |  |  | 2 |  |  |  |  |  |  | 3 | 0 | 4 |  |  |
| *Zelkova schneideriana* |  |  | 2 |  |  |  |  |  |  | 4 | 2 |  |  |  |
| *Garcinia paucinervis* | 5 |  |  |  |  |  |  |  |  |  |  | 1 |  | 2 |
| *Dracontomelon duperreanum* |  |  |  |  |  |  |  |  | 1 |  |  |  |  | 7 |
| *Pinus kwangtungensis* |  |  | 7 |  |  |  |  |  |  |  |  |  |  |  |
| *Styphnolobium japonicum* |  |  | 1 |  |  |  |  |  |  |  | 5 | 1 |  |  |
| *Boniodendron minus* |  |  |  |  |  |  |  |  |  |  | 1 | 6 |  |  |
| *Castanopsis fargesii* | 1 |  | 6 |  |  |  |  |  |  |  |  |  |  |  |
| *Acer coriaceifolium* |  |  |  |  |  |  |  |  |  |  | 7 |  |  |  |
| *Castanopsis tibetana* |  | 1 | 5 |  |  |  |  |  |  |  |  |  |  |  |
| *Antiaris toxicaria* |  |  |  |  | 2 |  | 1 |  | 1 |  |  |  |  | 2 |
| *Pinus massoniana* |  | 3 |  |  |  |  |  | 3 |  |  |  |  |  |  |
| *Fagus longipetiolata* |  |  | 6 |  |  |  |  |  |  |  |  |  |  |  |
| *Pterocarya stenoptera* |  |  | 5 |  |  |  |  |  |  |  |  |  |  |  |
| *Pseudotsuga sinensis* |  | 5 |  |  |  |  |  |  |  |  |  |  |  |  |
| *Phoebe bournei* |  | 1 | 3 |  |  |  |  |  |  |  | 1 |  |  |  |
| *Bischofia javanica* |  |  |  | 1 |  |  |  |  | 1 | 2 |  |  |  | 1 |
| *Carya cathayensis* |  |  |  |  |  |  |  |  |  |  |  | 5 |  |  |
| *Quercus variabilis* |  |  | 4 |  |  |  |  |  |  | 1 |  |  |  |  |
| *Diospyros decandra* |  |  |  |  |  | 5 |  |  |  |  |  |  |  |  |
| *Toona ciliata* |  |  | 2 |  |  |  |  |  |  |  |  | 2 |  |  |
| *Castanopsis faberi* |  | 1 | 1 |  |  |  |  |  |  |  | 2 |  |  |  |
| *Bombax ceiba* | 2 |  |  |  |  |  |  |  |  |  |  |  | 1 | 1 |
| *Celtis sinensis* |  |  |  |  |  |  |  |  |  | 3 | 1 |  |  |  |
| *Vitex quinata* |  | 1 | 2 |  |  |  |  | 1 |  |  |  |  |  |  |
| *Gleditsia sinensis* |  |  | 1 |  |  |  |  |  |  |  | 3 |  |  |  |
| *Liriodendron chinense* |  | 1 | 2 |  |  |  |  |  |  |  |  |  |  |  |
| *Keteleeria davidiana var.calcarea* |  |  |  |  |  |  |  |  |  |  | 1 | 2 |  |  |
| *Choerospondias axillaris* |  | 1 | 1 |  |  |  |  | 1 |  |  |  |  |  |  |
| *Zenia insignis* |  |  |  |  |  |  |  |  |  |  |  | 2 |  | 1 |
| *Keteleeria pubescens* |  |  | 2 |  |  |  |  |  |  |  |  | 1 |  |  |
| *Elaeocarpus sylvestris* |  |  |  |  |  |  | 1 |  |  | 1 | 1 |  |  |  |
| *Phoebe calcarea* |  |  |  |  |  |  |  |  |  | 3 |  |  |  |  |
| *Keteleeria davidiana* |  | 1 |  |  |  |  |  |  |  | 2 |  |  |  |  |
| *Lysidice rhodostegia* |  |  |  |  |  |  |  |  | 1 | 1 |  | 1 |  |  |
| *Schima argentea* |  |  | 3 |  |  |  |  |  |  |  |  |  |  |  |
| *Hovenia acerba* |  | 1 |  |  |  |  | 1 |  |  |  | 1 |  |  |  |
| *Quercus fabri* |  |  |  |  |  |  |  |  |  | 1 | 1 |  |  |  |
| *Prunus dulcis* |  |  |  |  |  |  |  |  |  | 2 |  |  |  |  |
| *Aphananthe aspera* |  | 1 | 1 |  |  |  |  |  |  |  |  |  |  |  |
| *Ilex chinensis* |  |  | 1 |  |  |  |  |  |  |  | 1 |  |  |  |
| *Photinia bodinieri* |  |  |  |  |  |  |  |  |  |  |  | 2 |  |  |
| *Artocarpus parvus* |  |  |  |  |  |  | 1 |  | 1 |  |  |  |  |  |
| *Adenanthera microsperma* |  |  |  |  |  |  |  |  | 1 |  |  |  |  | 1 |
| *Cephalotaxus hainanensis* |  |  |  |  |  |  |  |  | 1 | 1 |  |  |  |  |
| *Ulmus parvifolia* |  |  |  |  |  |  |  |  |  | 2 |  |  |  |  |
| *Podocarpus macrophyllus* |  | 1 | 1 |  |  |  |  |  |  |  |  |  |  |  |
| *Ligustrum lucidum* |  |  |  |  |  |  |  |  |  |  | 1 | 1 |  |  |
| *Syzygium levinei* |  |  |  |  |  |  | 1 |  |  |  |  |  |  | 1 |
| *Alstonia scholaris* |  |  |  |  |  |  |  |  | 1 |  |  |  |  | 1 |
| *Sinosideroxylon pedunculatum* |  |  |  |  |  |  |  |  |  | 1 |  | 1 |  |  |
| *Manglietia aromatica* |  |  |  |  |  |  |  |  |  | 1 |  | 1 |  |  |
| *Lithocarpus corneus* |  |  |  |  |  |  |  |  |  | 2 |  |  |  |  |
| *Castanopsis indica* |  |  |  |  |  |  |  |  |  |  |  | 2 |  |  |
| *Keteleeria fortunei* |  |  |  |  |  |  |  |  |  |  |  | 2 |  |  |
| *Saraca dives* |  |  |  |  |  |  |  |  |  | 2 |  |  |  |  |
| *Nageia nagi* |  |  | 2 |  |  |  |  |  |  |  |  |  |  |  |
| *Cupressus funebris* |  |  | 1 |  |  |  |  |  |  |  |  |  |  |  |
| *Machilus leptophylla* |  |  | 1 |  |  |  |  |  |  |  |  |  |  |  |
| *Platycladus orientalis* |  |  | 1 |  |  |  |  |  |  |  |  |  |  |  |
| *Sassafras tzumu* |  |  | 1 |  |  |  |  |  |  |  |  |  |  |  |
| *Litsea glutinosa* |  |  |  |  |  |  |  |  |  | 1 |  |  |  |  |
| *Platyosprion platycarpum* |  |  | 1 |  |  |  |  |  |  |  |  |  |  |  |
| *Ailanthus altissima* |  |  |  |  |  |  |  |  |  |  |  | 1 |  |  |
| *Calocedrus macrolepis* |  |  |  |  |  |  |  |  |  | 1 |  |  |  |  |
| *Ficus hookeriana* |  |  |  |  |  |  |  |  |  | 1 |  |  |  |  |
| *Aphananthe cuspidata* | 1 |  |  |  |  |  |  |  |  |  |  |  |  |  |
| *Quercus disciformis* |  |  |  |  |  |  |  |  |  | 1 |  |  |  |  |
| *Artocarpus styracifolius* |  |  | 1 |  |  |  |  |  |  |  |  |  |  |  |
| *Fraxinus griffithii* |  |  | 1 |  |  |  |  |  |  |  |  |  |  |  |
| *Lindera megaphylla* |  |  |  |  |  |  |  |  |  | 1 |  |  |  |  |
| *Sloanea sinensis* |  |  |  |  |  |  |  |  |  |  |  | 1 |  |  |
| *Morus cathayana* |  |  | 1 |  |  |  |  |  |  |  |  |  |  |  |
| *Dalbergia hupeana* |  | 1 |  |  |  |  |  |  |  |  |  |  |  |  |
| *Xanthophyllum hainanense* |  |  |  |  |  |  | 1 |  |  |  |  |  |  |  |
| *Elaeocarpus limitaneus* |  |  | 1 |  |  |  |  |  |  |  |  |  |  |  |
| *Loropetalum chinense* |  |  | 1 |  |  |  |  |  |  |  |  |  |  |  |
| *Keteleeria fortunei var.cyclolepis* |  |  |  |  |  |  |  |  |  |  |  |  | 1 |  |
| *Craibiodendron stellatum* |  |  |  | 1 |  |  |  |  |  |  |  |  |  |  |
| *Corylopsis sinensis* |  | 1 |  |  |  |  |  |  |  |  |  |  |  |  |
| *Tarennoidea wallichii* |  |  |  |  |  |  |  |  |  |  |  |  |  | 1 |
| *Cryptomeria japonica var.sinensis* |  |  | 1 |  |  |  |  |  |  |  |  |  |  |  |
| *Chukrasia tabularis* |  |  |  |  |  |  |  |  |  | 1 |  |  |  |  |
| *Castanea seguinii* |  |  |  |  |  |  |  |  |  |  |  | 1 |  |  |
| *Manglietia fordiana* |  |  |  |  |  |  |  |  |  |  |  | 1 |  |  |
| *Ormosia pubescens* |  |  |  |  |  | 1 |  |  |  |  |  |  |  |  |
| *Cephalotaxus fortunei* |  |  |  |  |  |  |  |  |  |  |  | 1 |  |  |
| *Aphanamixis polystachya* |  |  |  |  |  |  |  |  |  |  |  | 1 |  |  |
| *Diospyros japonica* |  |  |  |  |  |  |  |  |  |  |  | 1 |  |  |
| *Alphonsea mollis* |  |  |  |  |  |  |  |  |  | 1 |  |  |  |  |
| *Mallotus repandus* |  |  |  |  |  |  |  |  | 1 |  |  |  |  |  |
| *Glyptostrobus pensilis* | 1 |  |  |  |  |  |  |  |  |  |  |  |  |  |
| *Syzygium nervosum* |  |  |  |  |  |  | 1 |  |  |  |  |  |  |  |
| *Cycas szechuanensis* |  |  |  |  |  |  |  |  |  |  | 1 |  |  |  |
| *Malania oleifera* |  |  |  |  |  |  |  |  |  | 1 |  |  |  |  |
| *Pouteria annamensis* |  |  |  |  | 1 |  |  |  |  |  |  |  |  |  |
| *Manilkara hexandra* |  |  |  |  | 1 |  |  |  |  |  |  |  |  |  |
| *Aglaia lawii* |  |  |  |  |  |  |  |  |  | 1 |  |  |  |  |
| *Parashorea chinensis* |  |  |  |  |  |  |  |  |  |  |  | 1 |  |  |
| *Lagerstroemia caudata* |  |  |  |  |  |  |  |  |  |  |  | 1 |  |  |
| *Syzygium euonymifolium* |  |  |  | 1 |  |  |  |  |  |  |  |  |  |  |
| *Machilus wenshanensis* |  |  |  |  |  |  |  |  |  | 1 |  |  |  |  |
| *Diospyros eriantha* |  |  |  | 1 |  |  |  |  |  |  |  |  |  |  |
| *Ilex pentagona* |  |  |  |  |  |  |  |  |  |  |  | 1 |  |  |
| *Machilus rehderi* |  |  |  |  |  |  |  |  |  | 1 |  |  |  |  |
| *Toona sinensis* |  |  |  |  |  |  |  |  |  |  |  | 1 |  |  |
| *Lindera communis* |  | 1 |  |  |  |  |  |  |  |  |  |  |  |  |
| *Helicia cochinchinensis* |  |  | 1 |  |  |  |  |  |  |  |  |  |  |  |
| *Morella rubra* |  |  |  |  |  |  |  |  |  |  |  | 1 |  |  |
| *Crateva religiosa* |  |  | 1 |  |  |  |  |  |  |  |  |  |  |  |
| *Juniperus chinensis* |  |  |  |  |  |  |  |  |  |  | 1 |  |  |  |
| *Aesculus assamica* |  |  |  |  |  |  |  |  |  | 1 |  |  |  |  |
| *Handeliodendron bodinieri* |  |  |  |  |  |  |  |  |  | 1 |  |  |  |  |
| *Celtis biondii* |  |  | 1 |  |  |  |  |  |  |  |  |  |  |  |
| *Madhuca pasquieri* |  |  |  |  |  |  |  |  | 1 |  |  |  |  |  |
| *Phoebe sheareri* |  |  |  |  |  |  |  |  |  | 1 |  |  |  |  |
